# Supplementary material for: Challenging an old paradigm by demonstrating transition metal-like chemistry at a neutral nonmetal center
Source: Nat Commun. 2023 Oct 13;14:6456. doi: 10.1038/s41467-023-42127-3 (PMC10575908; doi:10.1038/s41467-023-42127-3)
Supplement: Supplementary file 1 — Supplemenatry informations [file 41467_2023_42127_MOESM1_ESM.pdf]

Supplementary Information

Challenging an old paradigm by demonstrating transition metal-like chemistry at a neutral nonmetal center

David Biskup,[1] Gregor Schnakenburg,[1] René T. Boeré,[2] Arturo Espinosa Ferao,*[3] Rainer K. Streubel*[1]

1 Institut für Anorganische Chemie, Rheinische Friedrich-Wilhelms-Universität Bonn, Gerhard-Domagk-Str. 1, 53121 Bonn, Germany.

2 Department of Chemistry and Biochemistry, University of Lethbridge, Lethbridge, AB T1K3M4, Canada.

3 Departamento de Química Orgánica, Facultad de Química, Campus Espinardo, Universidad de Murcia, 30100 Murcia, Spain.

Table of Contents

1 General methods S2

2 Experimental procedures and characterization S5

3 NMR spectra S9

4 NMR spectra of thermally treated complexes S62

5 Electrochemical experiments S66

6 X-ray diffraction studies S82

7 Theoretical Investigations S96

1 General methods

All reactions were performed under dried and deoxygenated argon atmosphere using Schlenk or glovebox techniques. The used argon (>99.998%) was purified by a system of three columns (deoxygenation by a BTS copper catalyst (BASF PuriStar® R3-15S) at ca. 100 °C, removing moisture with silica gel, phosphorus pentoxide desiccant with indicator (Sicapent®) and calcium chloride). Glassware, spatulae, cannulae as well as filter papers were dried in a compartment dryer at 110 °C for at least one hour. Additionally, the glassware was heated with a heat gun (up to 550 °C) under active vacuum (<0.02 mbar) and filled with argon three times. Sterile syringes were purged with argon three times before use. The solvents were dried by standard procedures[1] by refluxing over proper desiccants under an argon atmosphere (*n*-pentane, petroleum ether 40/65 and toluene over sodium wire (Ø = 2 mm); diethyl ether stabilized with 3,5-di-*tert*-butyl-4-hydroxytoluene (BHT) and tetrahydrofuran over benzophenone and sodium wire) for several days and distilled before use. Alternatively, diethyl ether and toluene were dried using a MBraun SPS-800 solvent purification system. For filtration Schlenk frits or stainless steel cannulae (Ø = 1 mm and 2 mm) with Whatman® glass microfiber filters (grade GF/B) were used. After use, devices made of stainless steel were cleaned with acetone, water and diluted hydrochloric acid and glassware by storage in a concentrated solution of potassium hydroxide in isopropanol for at least two days and in diluted hydrochloric acid for one day. Afterwards, the glassware was washed with water and soap, acetone and petroleum ether 40/65. All joints were greased with OKS 1112 grease or with PTFE paste (Carl Roth).

NMR spectra were recorded on a Bruker Avance I 300 MHz, Bruker Avance I 400 MHz, Bruker Avance I 500 MHz or Bruker Avance III HD Ascend 500 MHz spectrometer at the NMR department of the University of Bonn and subsequently analyzed by the program Mestrenova 14.2. The calibration of the ^{1}H and ^{13}C NMR spectra was done via the solvent residual signals relative to tetramethylsilane (<1% in $CDCl_3$) ($CDCl_3$: δ(^{1}H) = 7.26 ppm and δ(^{13}C) = 77.16 ppm, CD_2Cl_2: δ(^{1}H) = 5.32 ppm and δ(^{13}C) = 53.84 ppm, C_6D_6: δ(^{1}H) = 7.16 ppm and δ(^{13}C) = 128.06 ppm, toluene-d_8: δ(^{1}H) = 2.08 ppm, 6.97 ppm, 7.01 ppm or 7.09 ppm and δ(^{13}C) = 20.43 ppm, 125.13 ppm, 127.96 ppm, 128.87 ppm or 137.48 ppm, CD_3CN: δ(^{1}H) = 1.94 ppm or 3.58 ppm and δ(^{13}C) = 1.32 ppm or 118.26 ppm, THF-d_8: δ(^{1}H) = 1.72 ppm or 3.58 ppm and δ(^{13}C) = 25.31 ppm or 67.21 ppm).[2] ^{31}P NMR spectra were measured relative to 85% H_3PO_4(aq) as external reference by using the ^{2}H frequency of the deuterated solvent (lock frequency) and the frequency ratio ratio value Ξ(^{31}P) = 40.480742% as recommended by IUPAC, and ^{15}N NMR spectra via ge-2D NMR ^{1}H,^{15}N HMBC experiments relative to liquid ammonia by using the ^{2}H frequency of the deuterated solvent (lock frequency) and the

frequency ratio value $\Xi(^{15}N)$ = 10.132912%.[3] To obtain the ^{15}N NMR chemical shifts relative to CH_3NO_2, 380.5 ppm were subtracted.[4] All lock frequencies were calibrated internally against the ^1H signals of solutions of tetramethylsilane with a volume fraction of $\Phi \leq 1\%$ in the corresponding deuterated solvent. The used deuterated solvents were purified via distillation over proper desiccants ($CDCl_3$ and CD_2Cl_2 over CaH_2, and C_6D_6 and THF-d_8 over a potassium mirror, CD_3CN over molecular sieves (3 Å)), trap-to-trap recondensation and degassing by three freeze-pump-thaw cycles. The purified solvents were stored over 3 Å or 4 Å molecular sieves. The chemical shift (δ) is given in parts per million (ppm) and the coupling constant ($^nJ_{X,Y}$) in Hertz (Hz) as absolute values neglecting the sign where *n* is the number of bonds between the coupling nuclei X and Y. For assigning the multiplicity following abbreviations were used: s = singlet, d = doublet, dd = doublet of doublets, t = triplet, sept = septet, m = multiplet, sat = satellites and br = broad. For ^1H NMR spectra additionally the number of nuclei is given according which is determined via integration. The ^1H and ^{13}C NMR signals of compounds were assigned by a combination of COSY, NOESY, HMQC and HMBC experiments to unequivocally assign protons and carbon resonances of diastereotopic substituents. All measurements were performed at ambient temperature (298 K) if not stated otherwise.

Mass spectra using liquid injection field desorption ionization (LIFDI) were recorded on a Thermo Finnigan MAT 90 sector field instrument equipped with a LIFDI ion source (Linden CMS). The samples were dissolved in toluene or tetrahydrofuran. Electron impact ionization (EI) measurements were performed on a Thermo Finnigan MAT 95 XL sector field instrument using an ionization energy of 70 eV. The calibration and referencing was done using perfluorokerosene (PFK). Electrospray ionization (ESI) and atmospheric pressure chemical ionization (APCI) measurements were performed on a Thermo Fisher Scientific Orbitrap XL spectrometer with an HPLC autosampler using acetonitrile or dichloromethane as solvents. Solutions of highly air sensitive compounds for LIFDI and ESI measurements were prepared in a glovebox using dried, recondensed and degassed solvents. Only selected data are given for detected ions. The peaks are given in mass-to-charge ratio (m/z) while only the isotopomer with the highest relative abundance is represented. Additionally, the relative intensities of the peaks are given in parentheses and the proposed molecule fragments in square brackets. High resolution mass spectra (HRMS) that were obtained using ESI or APCI were recorded in a single measurement and, hence, no standard deviations for ESI/APCI HRMS were obtained.

ATR-IR spectra of solids were recorded in the spectral range of 4000–400 cm^{-1} on a Bruker Alpha FTIR spectrometer with a single-reflection ATR measurement attachment (Platinum-ATR Diamond) or a Shimadzu IRSpirit FTIR spectrometer with a single-reflection ATR

measurement attachment (QATR-S) in a glovebox at ambient temperature. The FT-IR spectra of solutions were recorded in the spectral range of 4000–400 cm^{-1} on a Shimdazu IRSpirit FTIR spectrometer using a stainless-steel cell with KBr windows (Omni-Cell SPECAC) separated by a 6 μm PTFE spacer (OMNI). For apodization the Happ-Genzel function was used. All analyses were performed using the programs EZ OMNIC 7.3 of Fisher Scientific, OPUS of Bruker and LabSolutions IR 2.26 of Shimadzu. Only selected wavenumbers of the absorption bands are given using reciprocal centimeters (cm^{-1}).

Elemental analyses were performed on a Elementar Vario Micro analysis device in quadruplicate or triplicate for each sample. All samples were prepared and weighed up in tin or silver sample containers using a micro-analytical balance in a glovebox. The mean C, H, N and S values are given for each compound.

Melting points were measured using an SRS DigiMelt device or a Büchi melting point determination device according to Dr. Tottoli. The samples were flame-sealed in a glass capillary (Ø = 0.1 mm) in vacuo (<0.02 mbar) and heated quickly (ca. 5 K min^{-1}) for a rough determination of the melting point or decomposition temperature. Afterwards, a heating rate of approximately 2 K min^{-1} was used until the sample melted or decomposed. The thermally treated samples were cooled to ambient temperature and studied by ^{1}H and/or ^{31}P NMR spectroscopy to confirm whether decomposition had occurred. No internal or external temperature corrections were performed.

Single crystal X-ray diffraction analyses were performed on a Bruker X8-KappaApex II diffractometer, a Bruker D8 Venture diffractometer, a STOE IPDS-2T diffractometer or a STOE STADIVARI diffractometer, equipped with a low-temperature device (Bruker Kryoflex, Oxford Cryostream 700 series or Oxford Cryostream 800 series) at 100(2) K, 123(2) K or 180(2) K by using graphite monochromated Mo-Kα radiation (λ = 0.71073 Å) or Cu-Kα radiation (λ = 1.54186 Å). Intensities were measured by fine-slicing Φ and ω scans and corrected background, polarization and Lorentz effects. A semi-empirical absorption correction was applied for the data sets following Blessing's method.[5] The structure was solved by direct methods and refined anisotropically by the least-squares procedure implemented in ShelX program system.[6] All non-hydrogen atoms were refined anisotropically. The hydrogen atoms were included isotropically refined using a riding model at the bound carbon atoms. The program Olex2 1.5[7] of OlexSys was used for analyses and the ellipsoid representations of the molecular structures with the probability level set to 50%. Crystallographic data for the structures reported in this paper have been deposited with the Cambridge Crystallographic Data Centre as supplementary publication no. CCDC 2250849 (**3b**), CCDC 2250850 (**3^{Cr}b**),

CCDC 2250851 (**3c**), CCDC 2250852 (**3^{Cr}c**), CCDC 2250853 (**3^{Cr}d**), CCDC 2250854 (**11a**), CCDC 2250855 (**11^{Cr}a**), CCDC 2250856 (**11b**) and CCDC 2250857 (**11^{Cr}b**) which can be obtained free of charge via www.ccdc.cam.ac.uk/data_request/cif.

2 Experimental procedures and characterization

Synthesis of complex 3^{Cr}b

A solution of a Li/Cl phosphinidenoid chromium(0) complex **1^{Cr}** was prepared using 0.564 g (1.05 mmol, 1.0 eq.) of [Cr(CO)$_5${P(CPh$_3$)Cl$_2$}], 0.16 mL (0.99 mmol, 0.9 eq.) of 12-crown-4 and 0.66 mL (*c* = 1.6 M in *n*-pentane, 1.06 mmol, 1.0 eq.) of a *tert*-butyllithium solution in 20 mL of THF at −80 °C. Afterwards, 0.237 g (1.94 mmol, 1.8 eq.) of 4-dimethylaminopyridine (DMAP) (**2b**) was added at −50 °C. The reaction mixture was stirred for 17 h while it was allowed to slowly warm up to ambient temperature. All volatiles were removed in vacuo (<0.02 mbar) at ambient temperature. The product was extracted five times with 20 mL of diethyl ether using a filter cannula (*Ø* = 2 mm) with a glass microfiber filter paper (Whatman® GF/B) and a Schlenk frit (filled with dry SiO$_2$, *Ø* = 3 cm, *h* = 3 cm). Residual product was extracted from the SiO$_2$ using three times 10 mL of diethyl ether at ambient temperature. Afterwards, 120 mL of *n*-pentane were added to the solution. The obtained yellow suspension was stirred for 1 h at ambient temperature. The supernatant was filtered off using a filter cannula (*Ø* = 2 mm) with a Whatman® 595 filter paper at ambient temperature and the yellow solid residue was washed once with 15 mL and twice with 5 mL of *n*-pentane at ambient temperature. The product was obtained as yellow solid after drying for 2 h in vacuo (<0.02 mbar) at ambient temperature. Yield: 0.199 g (0.34 mmol, 32%); yellow solid; mp: 145 °C (dec.); ^{1}H NMR (500.04 MHz, THF-*d*$_8$, 298 K): δ 7.67 (d, *J* = 6.1 Hz, 2H), 7.55–7.53 (m, 6H), 7.19–7.15 (m, 6H), 7.09–7.06 (m, 3H), 6.48–6.46 (m, 2H), 3.06 (s$_{sat}$, *J* = 67.29 Hz, 6H); ^{13}C{^{1}H} NMR (125.75 MHz, THF-*d*$_8$, 298 K): δ 225.7 (d, *J* = 2.8 Hz), 219.8 (d, *J* = 4.2 Hz), 156.8, 151.0 (d, *J* = 11.7 Hz), 147.6, 130.8 (d, *J* = 8.5 Hz), 128.3, 126.0, 106.4, 64.5 (d, *J* = 60.8 Hz), 39.4; ^{15}N{^{1}H} NMR (50.68 MHz, THF-*d*$_8$, 298 K): δ −192.1, −297.6; ^{31}P NMR (121.51 MHz, THF-*d*$_8$, 298 K): δ 281.1 (br s); IR (ATR Diamond): 1920 (ν_{CO}), 1970 (ν_{CO}), 2042 (ν_{CO}); MS (LIFDI): *m/z* (%): 588.1 (100) [*M*]$^+$, 314.0 (9) [Cr(CO)$_5$(dmap)]$^+$, 243.1 (22) [CPh$_3$]$^+$; elemental analysis calcd (%) for $C_{31}H_{25}N_2O_5PCr$: C 63.27, H 4.28, N 4.76; found: C 62.90, H 4.62, N 4.64.

Synthesis of complex 3^{Cr}c

A solution of a Li/Cl phosphinidenoid chromium(0) complex **1^{Cr}** was prepared using 4.721 g (8.79 mmol, 1.0 eq.) of [Cr(CO)$_5${P(CPh$_3$)Cl$_2$}], 1.42 mL (8.78 mmol, 1.0 eq.) of 12-crown-4 and 5.50 mL (*c* = 1.6 M in *n*-pentane, 8.80 mmol, 1.0 eq.) of a *tert*-butyllithium solution in 50 mL of

THF at −80 °C. Afterwards, 1.06 mL (13.30 mmol, 1.5 eq.) of *N*-methylimidazole (**2c**) were added dropwise at −50 °C. The reaction mixture was stirred for 17 h while it was allowed to slowly warm up to ambient temperature. All volatiles were removed in vacuo (<0.02 mbar) at ambient temperature. The product was extracted five times with 60 mL of diethyl ether using a filter cannula (Ø = 2 mm) with a glass microfiber filter paper (Whatman® GF/B) and a P3 Schlenk frit (filled with dry SiO_2, Ø = 3 cm, *h* = 3 cm). Residual product was extracted from the SiO_2 using five times 60 mL of diethyl ether. Afterwards, 420 mL of *n*-pentane were added to the solution. The obtained yellow suspension was stirred for 30 minutes at ambient temperature and stored stationary at −40 °C for 40 h. The supernatant was filtered off using a filter cannula (Ø = 2 mm) with a Whatman® 595 filter paper at ambient temperature and the yellow solid residue was washed three times using 44 mL of a 10:1 *n*-pentane/diethyl ether mixture at ambient temperature. The product was obtained as yellow solid after drying for 3 h in vacuo (<0.02 mbar) at ambient temperature. Yield: 2.62 g (4.78 mmol, 54%); yellow solid; mp: 141 °C (dec.); ^{1}H NMR (300.13 MHz, C_6D_6, 300 K): δ 7.78–7.69 (m, 6H), 7.15–7.11 (m, 6H), 6.99–6.96 (m, 3H), 6.45–6.44 (m, 1H), 6.33 (m, 1H), 5.11–5.10 (m, 1H), 1.81 (s, 3H); $^{13}C\{^{1}H\}$ NMR (125.75 MHz, C_6D_6, 298 K): δ 225.3, 219.8 (d, *J* = 4.5 Hz), 147.5 (d, *J* = 9.9 Hz), 141.4 (d, *J* = 3.1 Hz), 130.5 (br s), 130.3 (d, *J* = 8.0 Hz), 128.2, 125.9, 119.5, 63.5 (d, *J* = 56.6 Hz), 33.8; $^{15}N\{^{1}H\}$ NMR (50.68 MHz, C_6D_6, 298 K): δ −177.7, −215.2; ^{31}P NMR (121.51 MHz, C_6D_6, 299 K): δ 248.0 (br s); IR (solution in THF, c = 1.5 mM): 1920 (ν_{CO}), 1963 (ν_{CO}), 2043 (ν_{CO}); MS (LIFDI): *m/z* (%) = 548.1 (100) $[M]^+$, 274.0 (25) $[Cr(CO)_5(N\text{-MeIm})]^+$, 243.1 (98) $[CPh_3]^+$; elemental analysis calcd (%) for $C_{28}H_{21}N_2O_5PCr$: C 61.32, H 3.86, N 5.11; found: C 61.34, H 4.12, N 5.10.

Synthesis of complex 3^{Cr}d

A solution of a Li/Cl phosphinidenoid chromium(0) complex $\mathbf{1}^{Cr}$ was prepared using 0.54 g (1.00 mmol, 1.0 eq.) of $[Cr(CO)_5\{P(CPh_3)Cl_2\}]$, 0.15 mL (0.93 mmol, 0.9 eq.) of 12-crown-4 and 0.65 mL (*c* = 1.7 M in *n*-pentane, 1.10 mmol, 1.1 eq.) of a *tert*-butyllithium solution in 30 mL of THF at −80 °C. Afterwards, 0.17 mL (1.50 mmol, 1.5 eq.) of *tert*-butyl isocyanide (**2d**) was added dropwise at −50 °C. The reaction mixture was stirred for 19 h while it was allowed to slowly warm up to ambient temperature. All volatiles were removed in vacuo (<0.02 mbar) at ambient temperature. The product was extracted six times with 20 mL of *n*-pentane. The solvent was removed in vacuo (<0.02 mbar) at ambient temperature and the product was obtained as yellow solid after drying under the same conditions for 10 minutes. Yield: 0.52 g (0.94 mmol, 94%); yellow solid; mp: 93 °C (dec.); ^{1}H NMR (300.13 MHz, C_6D_6, 299 K): δ 7.44–6.96 (m, 15H), 0.72 (s, 9H); $^{13}C\{^{1}H\}$ NMR (75.48 MHz, C_6D_6, 300 K): δ 224.1 (d, *J* = 3.6 Hz),

217.4, 147.3 (d, J = 6.8 Hz), 143.6, 130.6 (d, J = 8.1 Hz), 128.3, 127.0 (d, J = 1.6 Hz), 61.3, 60.0 (d, J = 28.4 Hz), 29.0 (d, J = 1.6 Hz); $^{15}N\{^1H\}$ NMR (50.69 MHz, C_6D_6, 298 K): δ −170.0; ^{31}P NMR (121.51 MHz, $CDCl_3$, 300 K): δ −11.4; IR (ATR diamond): 1894 (ν_{CO}), 1925 (ν_{CO}), 2049 (m) (ν_{CO}), 2132 (w) (ν_{CN}); MS (LIFDI): *m/z* (%) = 549.1 (100) [*M*]$^+$, 243.1 (24) $[CPh_3]^+$; elemental analysis calcd (%) for $C_{29}H_{24}NO_5PCr$: C 63.39, H 4.40, N 2.55; found: C 63.76, H 4.64, N 2.45.

Synthesis of complex 11^{Cr}a

2.2 mL (c = 2 M in THF, 4.40 mmol, 10.2 eq.) of a methylamine solution were added to a solution of 0.238 g (0.43 mmol, 1.0 eq.) of complex **3^{Cr}d** in 10 mL of THF. The solution was stirred for 18 h at ambient temperature. Afterwards, all volatiles were removed in vacuo (<0.02 mbar) at ambient temperature and the obtained yellow solid was further dried under the same conditions for 35 minutes. Yield: 0.19 g (0.32 mmol, 75%); yellow solid; mp: 129–130 °C (dec.); ^1H NMR (300.13 MHz, C_6D_6, 299 K): δ 7.42–7.02 (m, 15H), 6.33 (d, J = 338.87 Hz, 1H), 3.30 (s, 1H), 2.98 (s, 3H), 1.18 (s, 9H); $^{13}C\{^1H\}$ NMR (125.78 MHz, C_6D_6, 297 K): δ 220.7 (d, J = 4.1 Hz), 215.7 (d, J = 10.6 Hz), 151.9 (d, J = 3.0 Hz), 143.1, 129.4, 127.5, 127.2, 60.4 (d, J = 6.8 Hz), 52.3 (d, J = 5.7 Hz), 37.8 (d, J = 13.4 Hz), 28.2; $^{15}N\{^1H\}$ NMR (50.69 MHz, C_6D_6, 298 K): δ −144.8, −258.7; ^{31}P NMR (121.51 MHz, C_6D_6, 299 K): δ 14.4 (d, J = 339.5 Hz); IR (ATR diamond): 1919 (ν_{CO}), 2000 (ν_{CO}), 2064 (ν_{CO}), 2371 (ν_{PH}), 3413 (ν_{NH}); MS (LIFDI): *m/z* (%) = 580.0 (100) [*M*]$^+$, 243.1 (12) $[CPh_3]^+$; elemental analysis calcd (%) for $C_{30}H_{29}N_2O_5PCr$: C 62.07, H 5.04, N 4.83; found: C 62.04, H 5.32, N 4.71.

Synthesis of complex 11^{Cr}b

0.215 g (0.39 mmol, 1.0 eq.) of complex **3^{Cr}d** was dissolved in 10 mL (117 mmol, 299 eq.) of isopropylamine at ambient temperature. The reaction mixture was stirred for 11 h at ambient temperature. All volatiles were removed in vacuo (<0.02 mbar) at ambient temperature. Yield: 0.23 g (0.37 mmol, 95%); pale-yellow solid; mp: 130–131 °C (dec.); ^1H NMR (300.13 MHz, C_6D_6, 298 K): δ 7.59–7.00 (m, 15H), 6.20 (dd, J = 336.73 Hz, J = 1.22 Hz, 1H), 3.36 (s, 1H), 3.27 (sept, J = 6.10 Hz, 1H), 1.21 (d, J = 6.10 Hz, 3H), 1.21 (s, 9H), 0.91 (d, J = 6.10 Hz, 3H); $^{13}C\{^1H\}$ NMR (75.48 MHz, C_6D_6, 298 K): δ 221.0 (d, J = 4.0 Hz), 216.2 (d, J = 10.5 Hz), 147.5 (d, J = 4.4 Hz), 144.4, 129.9, 128.6, 127.9, 60.9 (d, J = 5.8 Hz), 52.6 (d, J = 6.1 Hz), 51.4 (d, J = 10.5 Hz), 28.4, 25.0, 24.8; $^{15}N\{^1H\}$ NMR (50.69 MHz, C_6D_6, 298 K): δ −112.6, −259.3; ^{31}P NMR (121.51 MHz, C_6D_6, 298 K): δ 17.6 (d, J = 339.9 Hz); IR (ATR diamond): 1918 (ν_{CO}), 1989 (ν_{CO}), 2062 (ν_{CO}), 2377 (ν_{PH}), 3410 (ν_{NH}); MS (EI, 70 eV): *m/z* (%) = 608.0 (<0.1) [*M*]$^+$, 468.0

(3) [M−5CO]$^+$, 243.0 (100) [CPh_3]$^+$, 165.0 (83) [$HCPh_2$]$^+$, 141.1 (17) [C(N^{i}Pr)N(H)^{t}Bu]$^+$, 77.0 (3) [Ph]$^+$, 57.0 (12) [^{t}Bu]$^+$; MS (LIFDI): *m/z* (%) = 608.2 (100) [M]$^+$, 580.4 (73) [M−CO]$^+$, 417.5 (9) [M−Cr(CO)$_5$+H]$^+$, 243.3 (20) [CPh_3]$^+$; elemental analysis calcd (%) for $C_{32}H_{33}N_2O_5PCr$: C 63.15, H 5.47, N 4.60; found: C 62.79, H 5.67, N 4.50.

Generation of complex 3a

A solution of a Li/Cl phosphinidenoid tungsten(0) complex **1** was prepared using 0.114 g (0.17 mmol, 1.0 eq.) of [W(CO)$_5${P(CPh$_3$)Cl$_2$}], 27 µL (0.17 mmol, 1.0 eq.) of 12-crown-4 and 0.11 mL (c = 1.6 M in *n*-pentane, 0.18 mmol, 1.0 eq.) of a *tert*-butyllithium solution in 2 mL of THF using a 10 mL Schlenk tube with cooling mantle attached to a cryostat (Thermo Haake PhoenixII CT80L with ethanol as refrigerant) at −77 °C. The obtained red solution was stirred for 40 minutes at −77 °C. Afterwards, 0.28 mL (3.47 mmol, 20 eq.) of pyridine (**2a**) was added dropwise at −77 °C. The reaction mixture was stirred for 5 h while it was allowed to constantly warm up to −20 °C using the ramp function of the cryostat to obtain a dark reddish-brown suspension. All volatiles were removed in vacuo (<0.02 mbar) at −20 °C within 2 h. The product fully decomposed when extracted with diethyl ether using a P3 Schlenk frit (filled with dried Al_2O_3, *Ø* = 1 cm, *h* = 1 cm) at −40 °C. Content in reaction solution: 68% (via ^{31}P NMR integration); ^{31}P{^{1}H} NMR (121.51 MHz, THF, 298 K): δ 284.6 (br s$_{sat}$, J < 100 Hz). The coupling constant cannot be determined exactly due to the broadness of the signal.

Generation of complex 3^{Cr}a

A solution of a Li/Cl phosphinidenoid chromium(0) complex **1^{Cr}** was prepared using 0.606 g (1.13 mmol, 1.0 eq.) of [Cr(CO)$_5${P(CPh$_3$)Cl$_2$}], 0.18 mL (1.11 mmol, 1.0 eq.) of 12-crown-4 and 0.71 mL (c = 1.6 M in *n*-pentane, 1.14 mmol, 1.0 eq.) of a *tert*-butyllithium solution in 15 mL of THF at −80 °C. Afterwards, 1.82 mL (22.6 mmol, 20 eq.) of pyridine (**2a**) were added dropwise at −50 °C. The reaction mixture was stirred for 7 h while it was allowed to slowly warm up to ambient temperature to obtain a dark brownish-violet suspension. All volatiles were removed in vacuo (<0.02 mbar) at ambient temperature. The product fully decomposed when extracted with diethyl ether using a filter cannula (*Ø* = 2 mm) with a glass microfiber filter (Whatman® GF/B) at ambient temperature. Content in reaction solution: 74% (via ^{31}P NMR integration); ^{31}P{^{1}H} NMR (121.51 MHz, THF, 300 K): δ 335.3 (br s).

Generation of complex 12

0.05 mL (0.48 mmol, 4.4 eq.) of *tert*-butylamine was added to a solution of 0.073 g (0.11 mmol, 1.0 eq.) of complex **3d** in 3 mL of THF. The reaction mixture was stirred for 12 days at 40 °C.

Content in reactions solution: 74% (via ^{31}P NMR integration); ^{31}P NMR (202.48 MHz, THF, 298 K): δ −33.8 (s_{sat}, J = 103.8 Hz).

3 NMR spectra

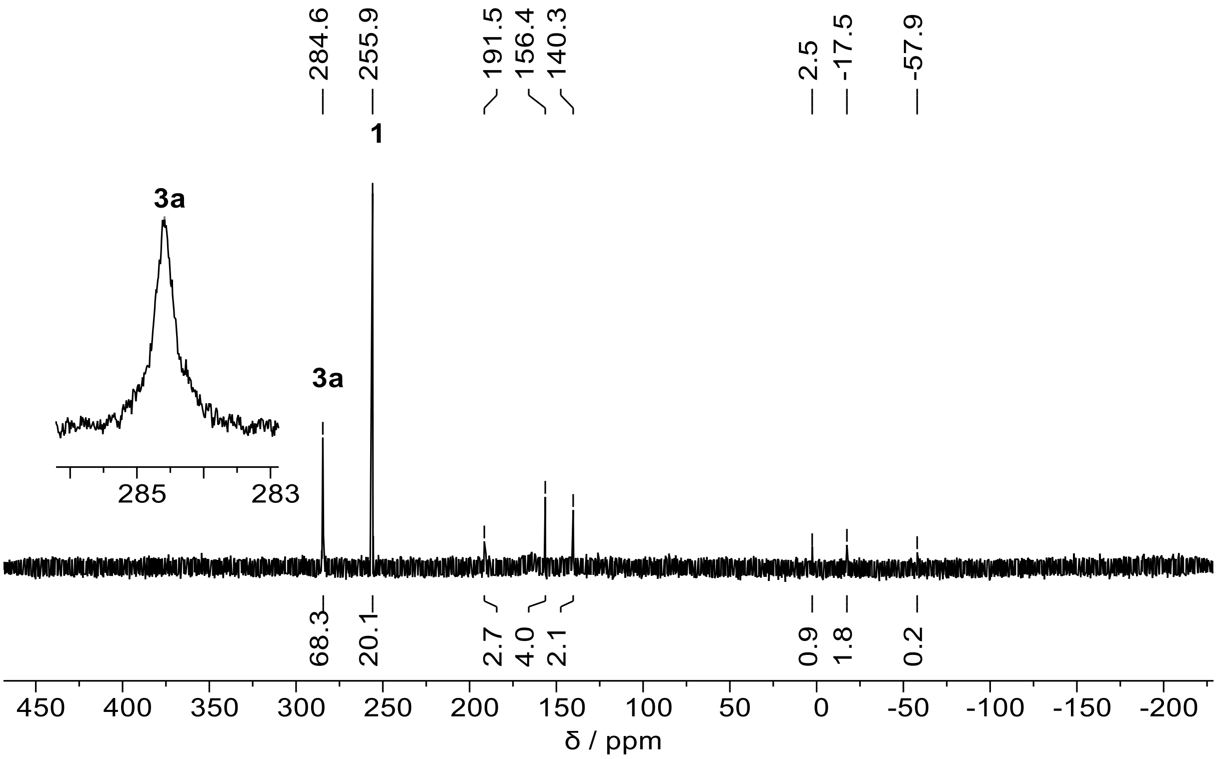

Figure 1: ^{31}P{^{1}H} NMR spectrum (121.51 MHz, THF, 298 K) of the reaction mixture of **1** with pyridine (**2a**) under formation of pyridine-to-phosphinidene complex adduct **3a**.

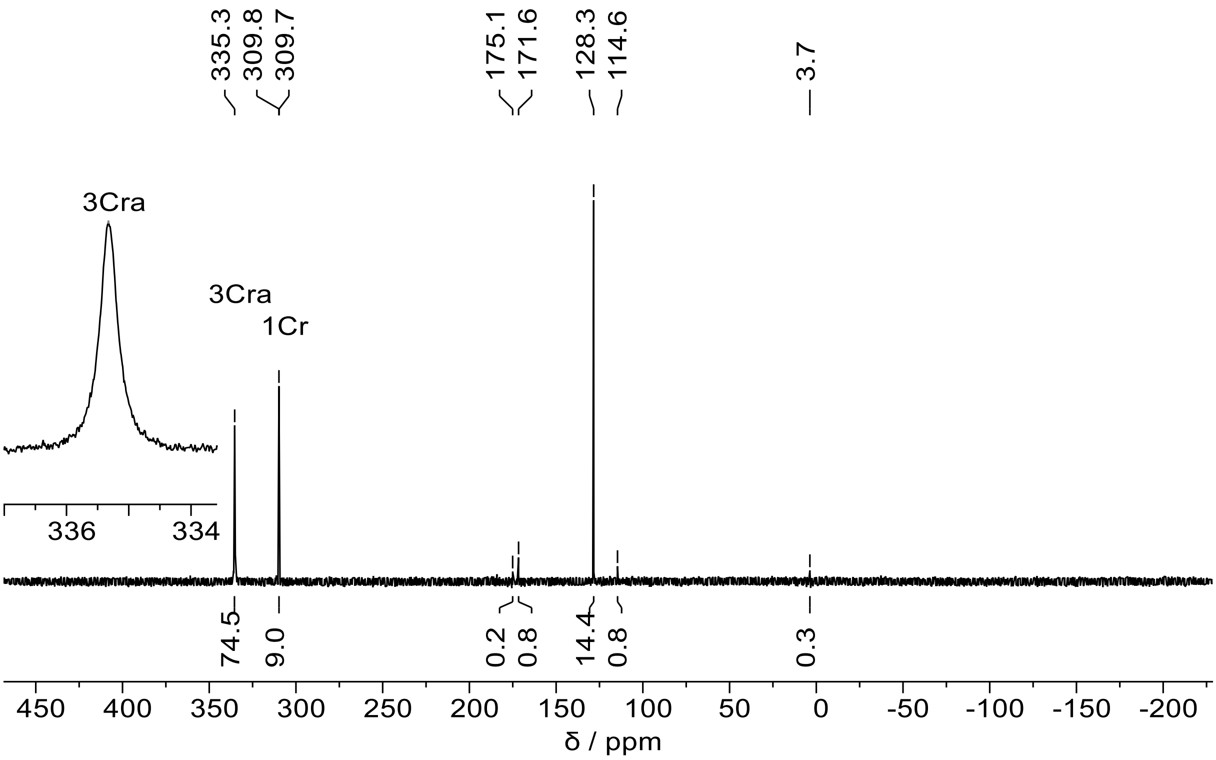

Figure 2: $^{31}P\{^1H\}$ NMR spectrum (121.51 MHz, THF, 300 K) of the reaction mixture of **1^{Cr}** with pyridine (**2a**) under formation of pyridine-to-phosphinidene complex adduct **3^{Cr}a**.

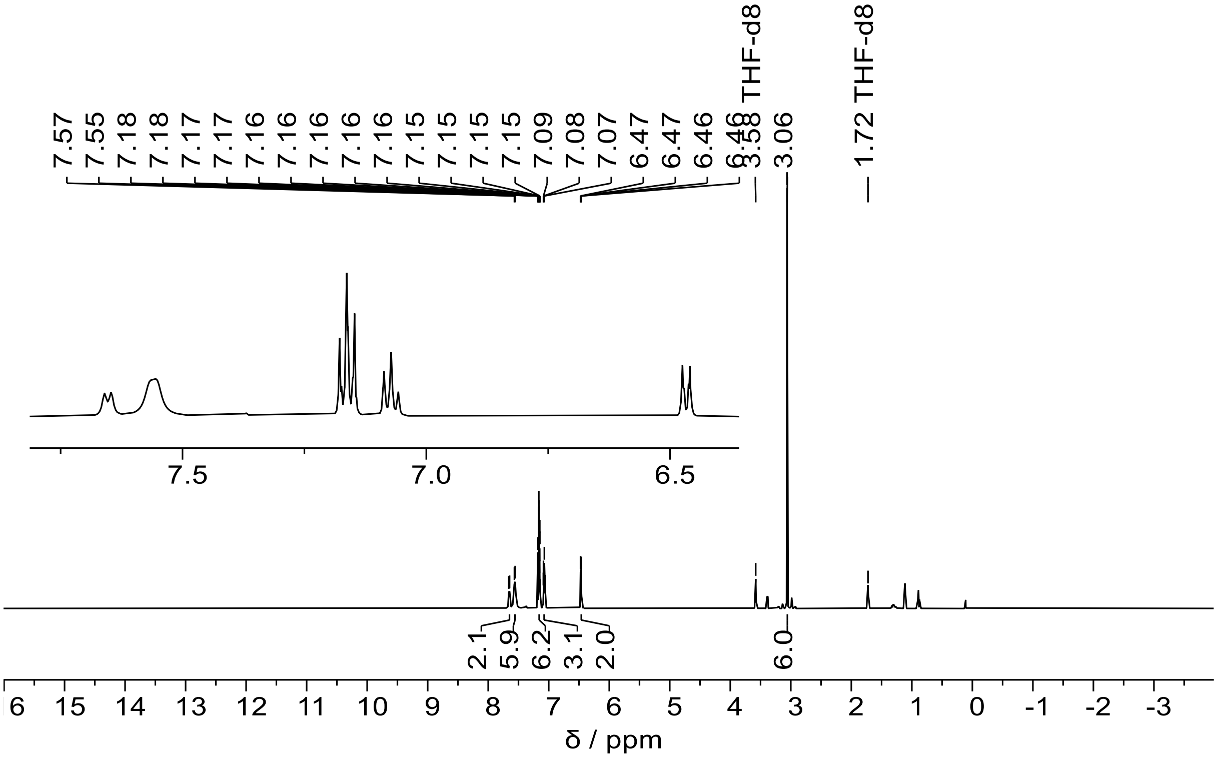

Figure 3: ^1H NMR spectrum (500.04 MHz, THF-d_8, 298 K) of complex **3b**.

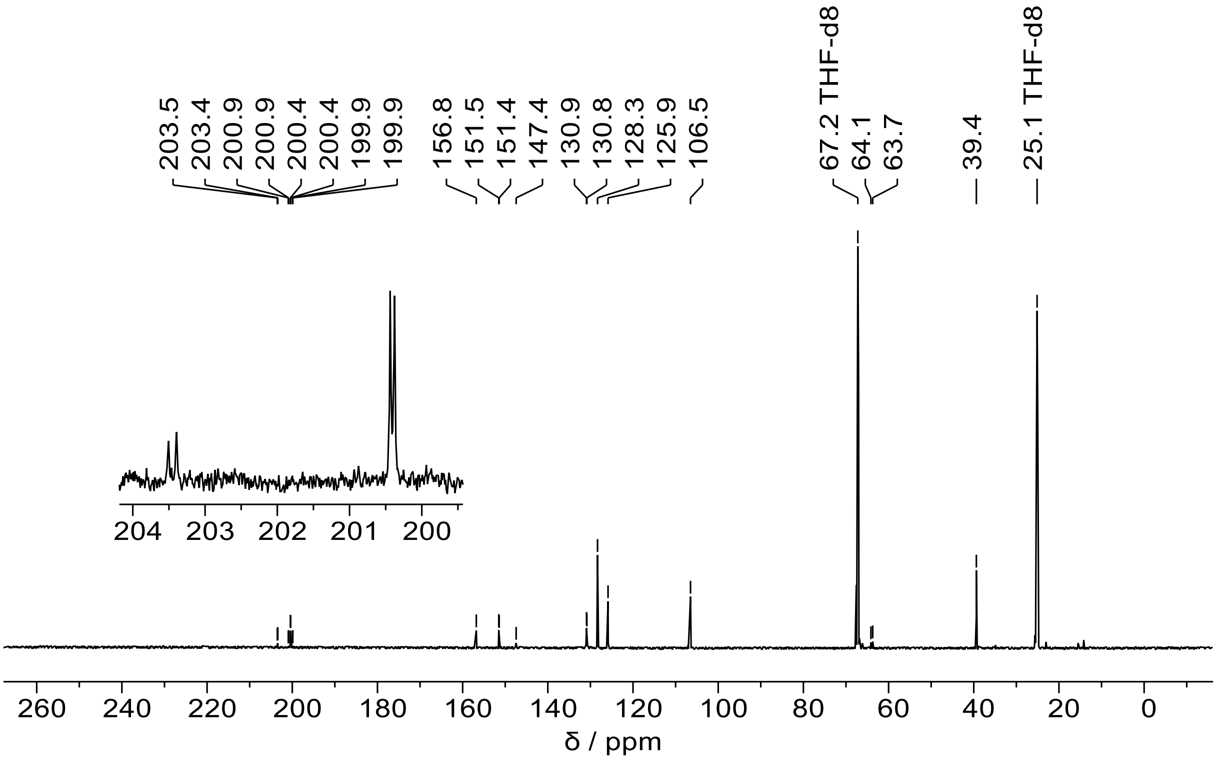

Figure 4: $^{13}C\{^{1}H\}$ NMR spectrum (125.75 MHz, THF-d_8, 298 K) of complex **3b**.

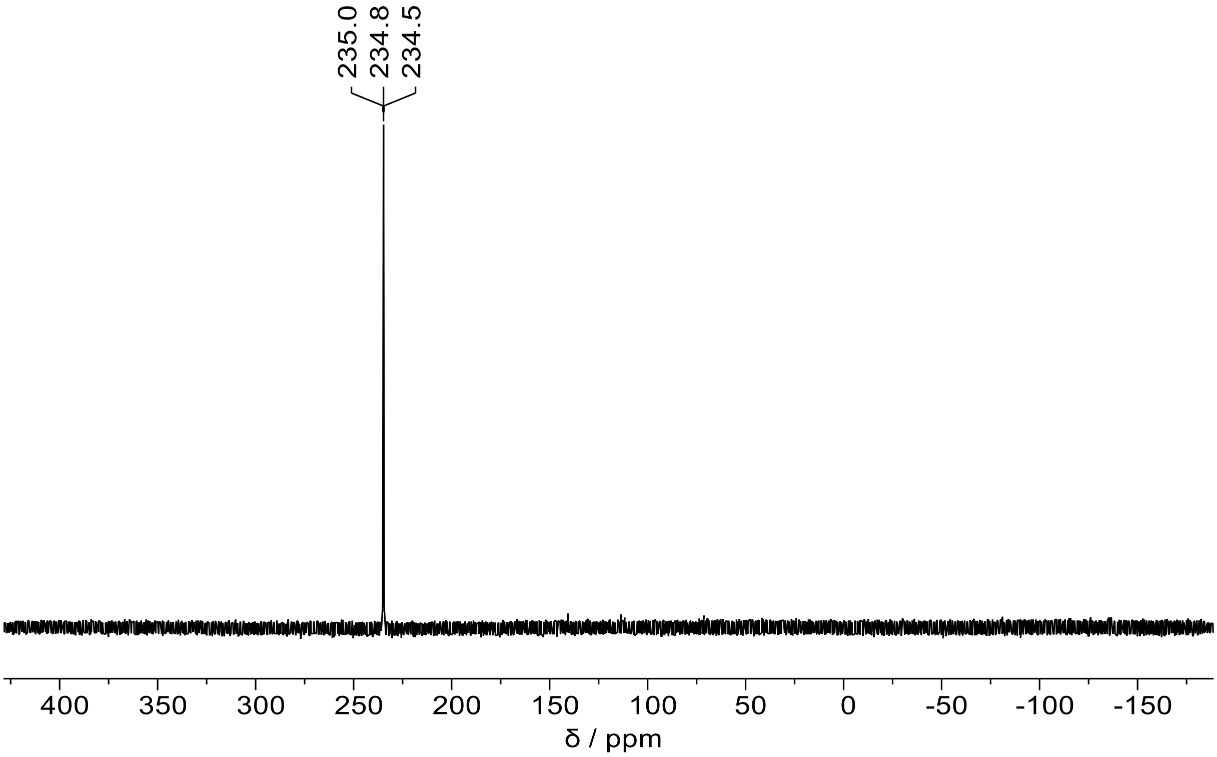

Figure 5: ^{31}P NMR spectrum (125.75 MHz, THF-d_8, 298 K) of complex **3b**.

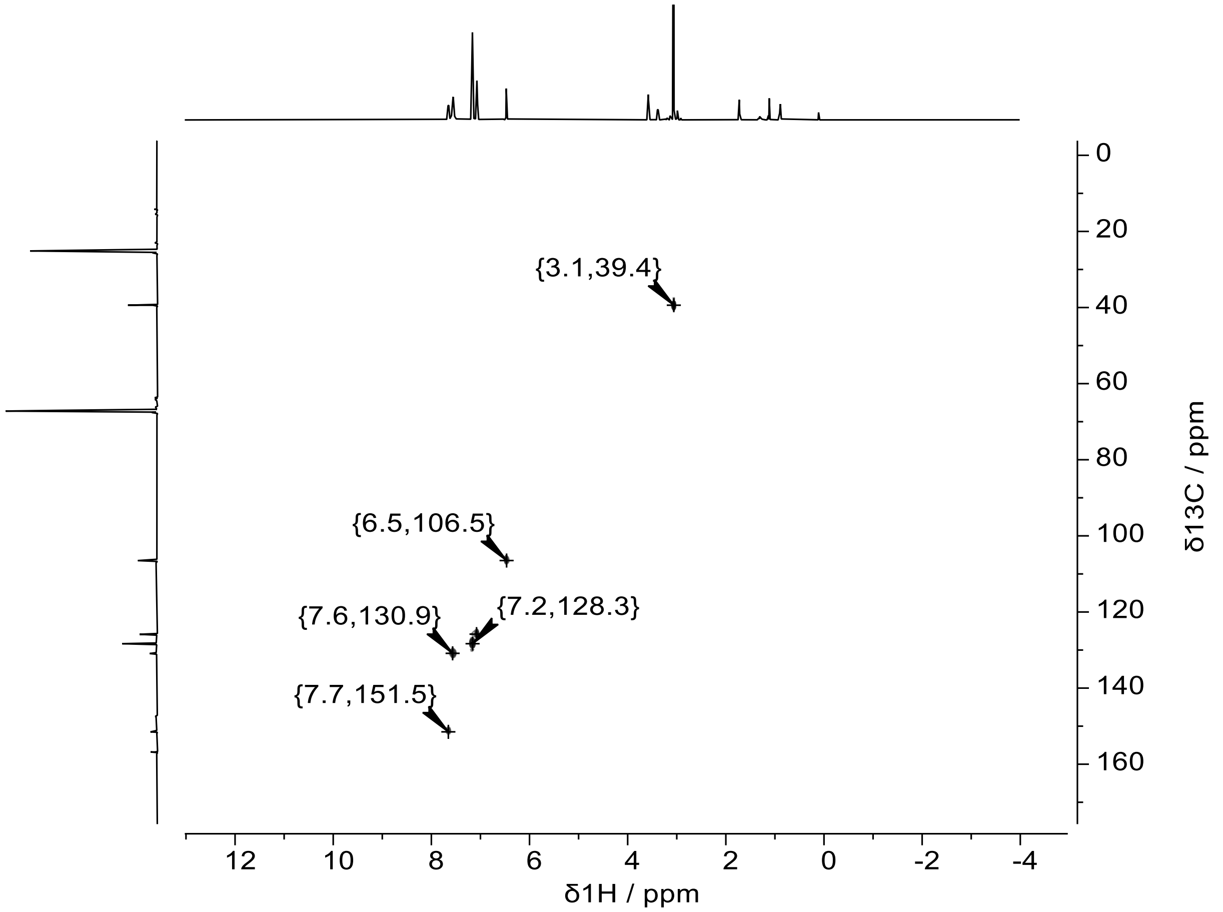

Figure 6: ^{1}H,^{13}C HSQC NMR spectrum (500.04 MHz, 125.75 MHz, THF-d_8, 298 K) of complex **3b**.

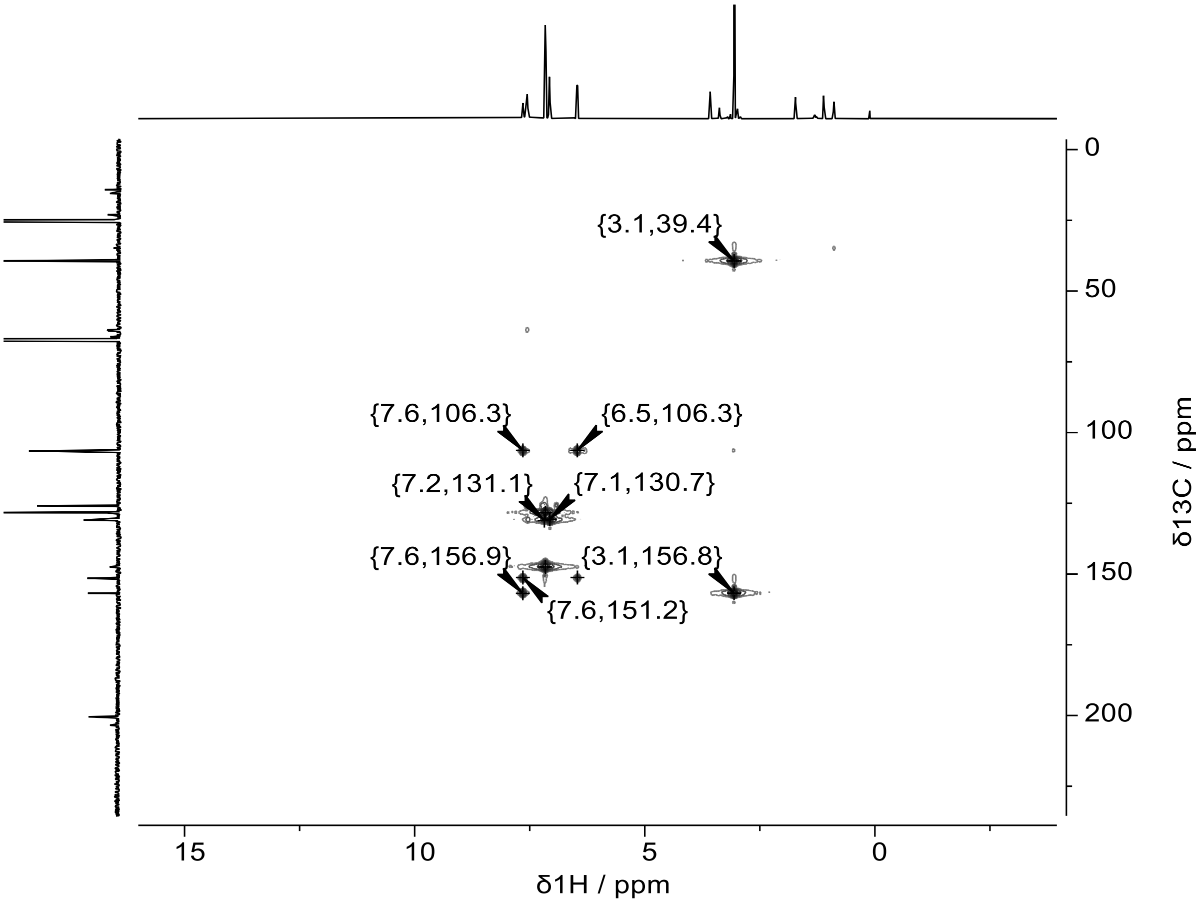

Figure 7: $^{1}H,^{13}C$ HMBC NMR spectrum (500.04 MHz, 125.75 MHz, THF-d_8, 298 K) of complex **3b**.

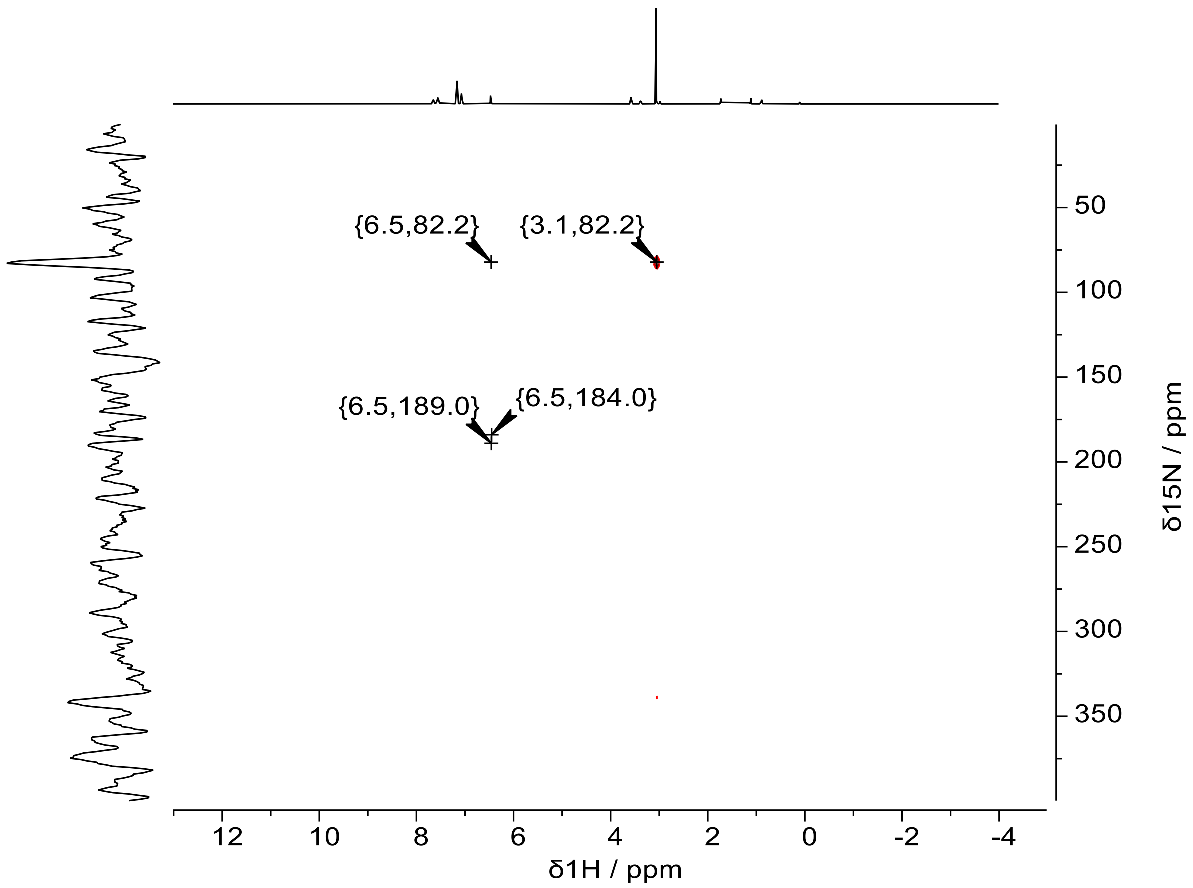

Figure 8: $^{1}H,^{15}N$ HMBC NMR spectrum (500.04 MHz, 50.68 MHz, THF-*d*$_8$, 298 K) of complex **3b**.

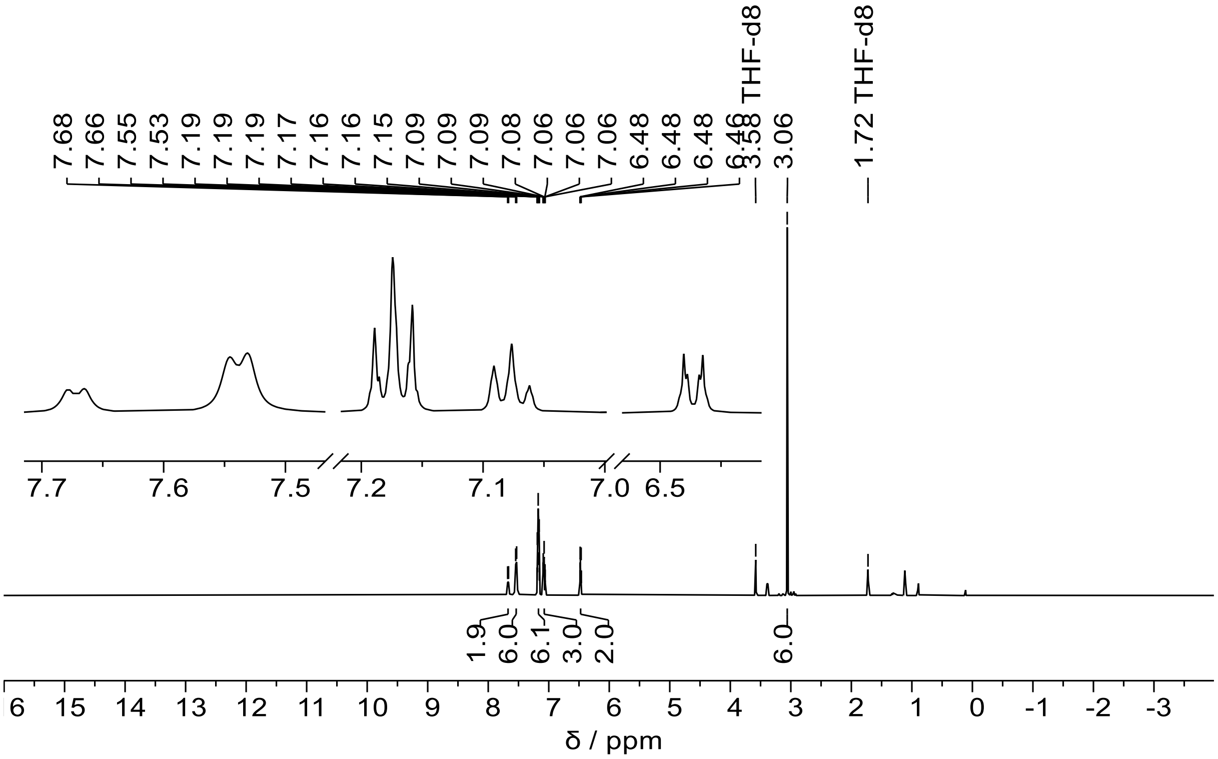

Figure 9: ^{1}H NMR spectrum (500.04 MHz, THF-*d*$_8$, 298 K) of complex **3^{Cr}b**.

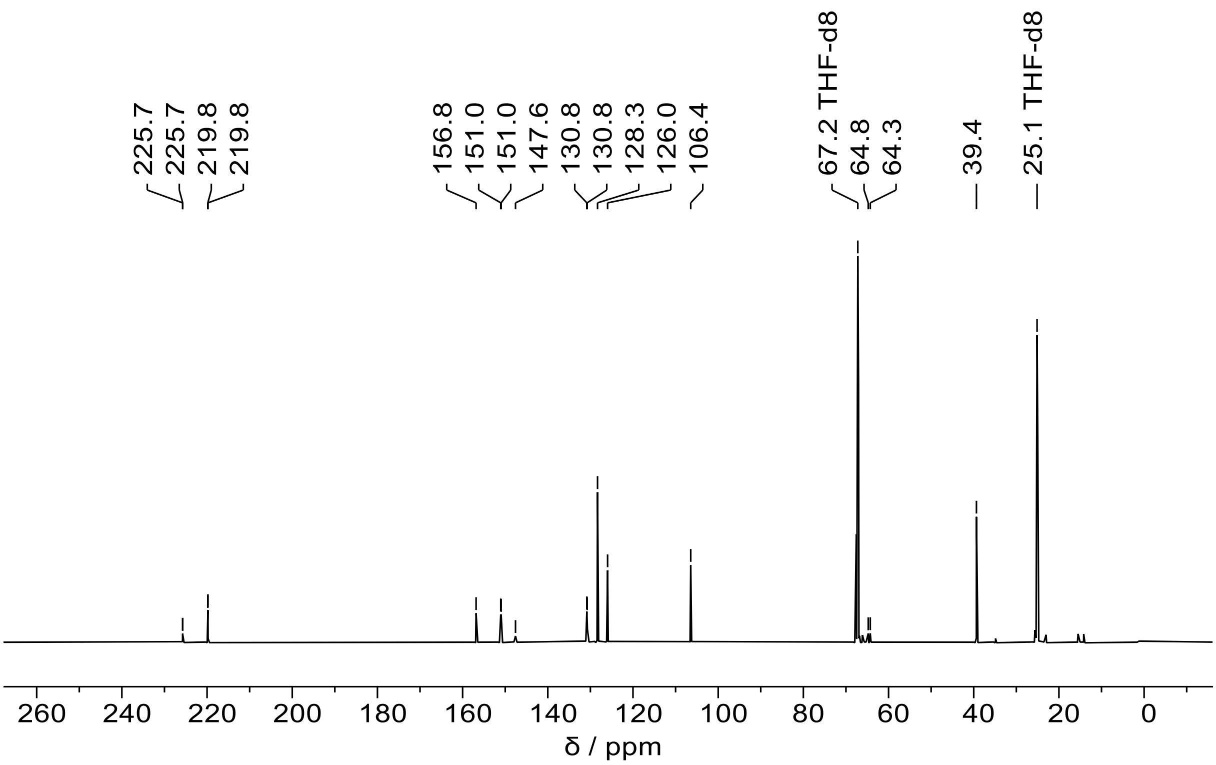

Figure 10: $^{13}C\{^{1}H\}$ NMR spectrum (125.75 MHz, THF-d_8, 298 K) of complex $\mathbf{3^{Cr}b}$.

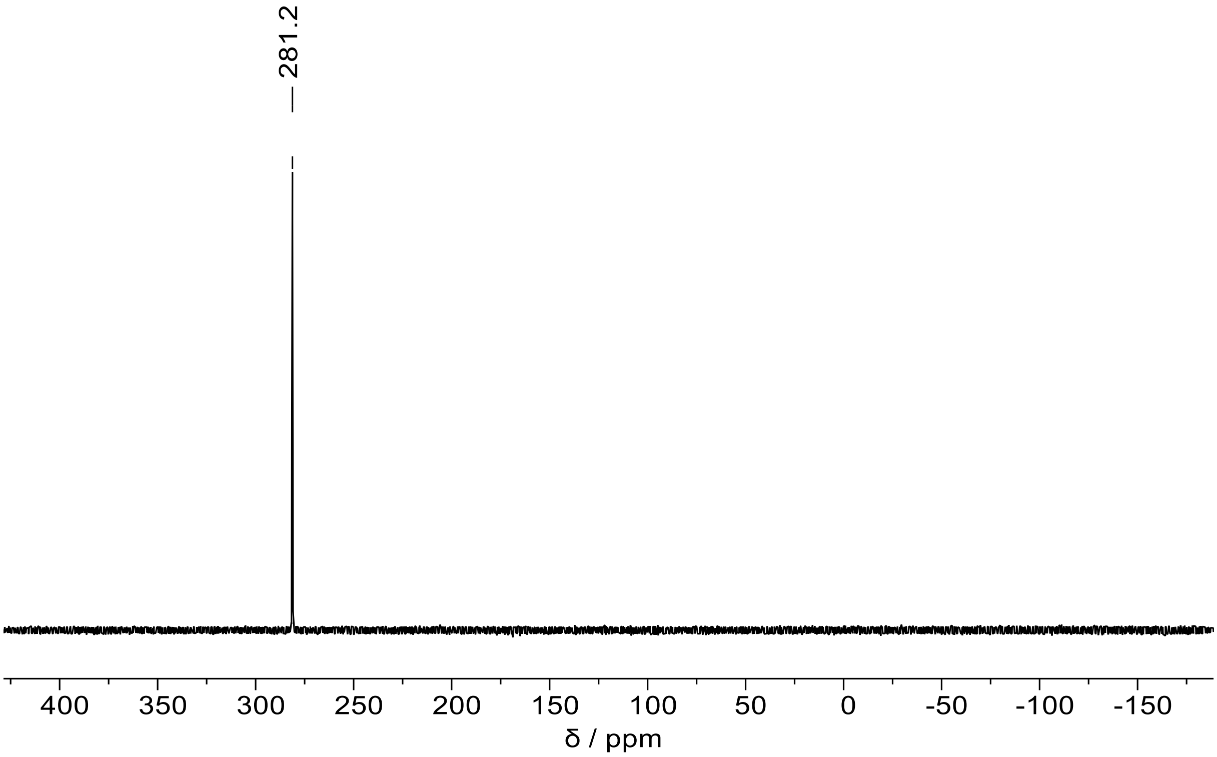

Figure 11: $^{31}P\{^{1}H\}$ NMR spectrum (202.44 MHz, THF-d_8, 298 K) of complex $\mathbf{3^{Cr}b}$.

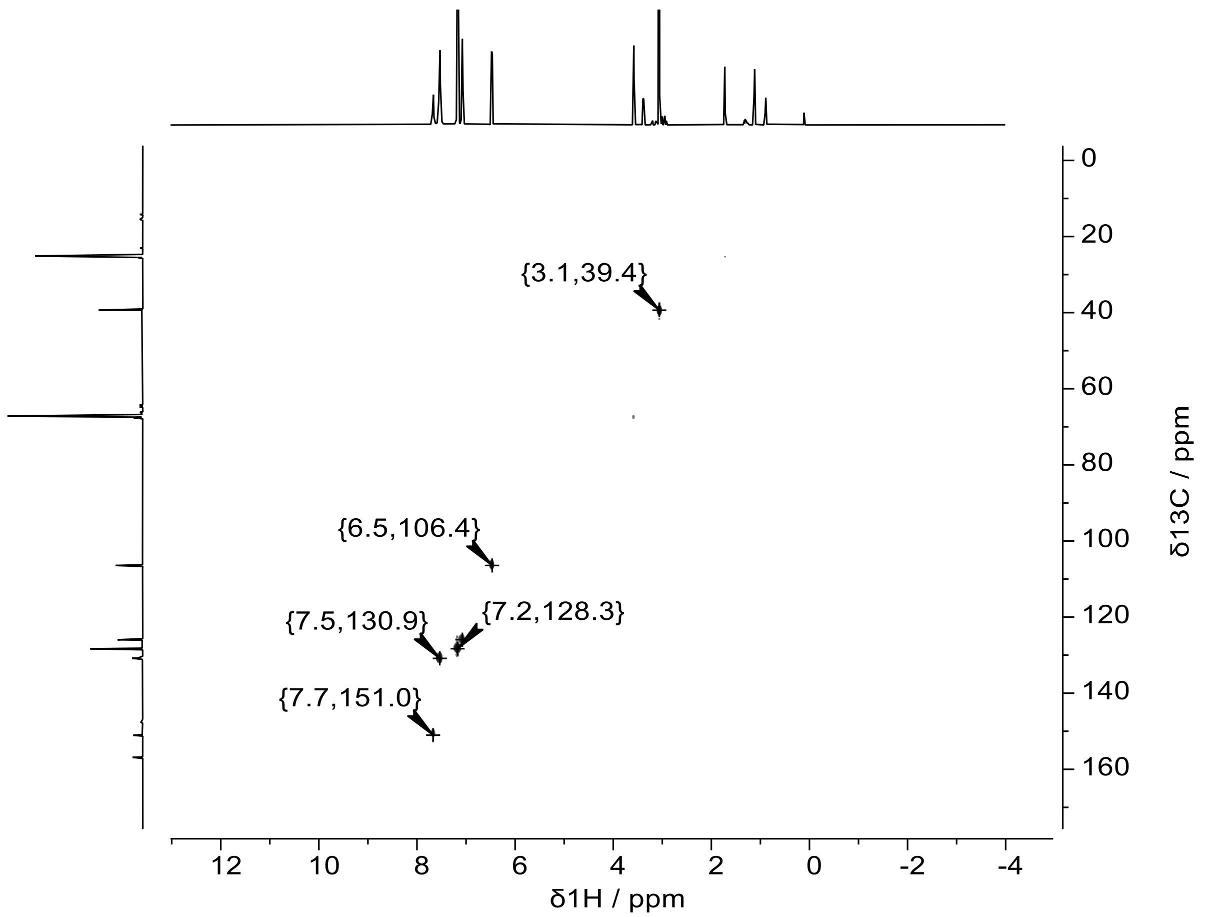

Figure 12: $^{1}H,^{13}C$ HSQC NMR spectrum (500.04 MHz, 125.75 MHz, THF-d_8, 298 K) of complex $\mathbf{3^{Cr}b}$.

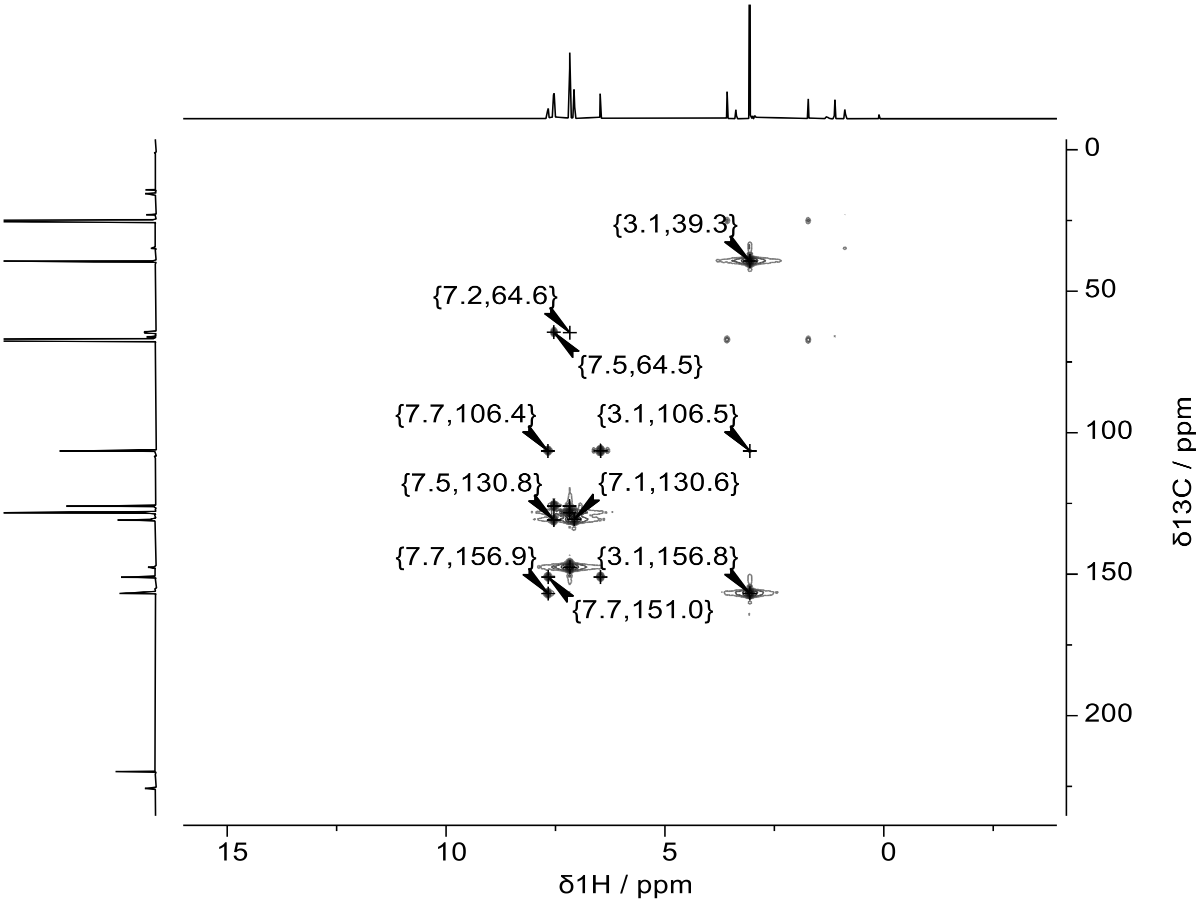

Figure 13: $^{1}H,^{13}C$ HMBC NMR spectrum (500.04 MHz, 125.75 MHz, THF-d_8, 298 K) of complex $\mathbf{3^{Cr}b}$.

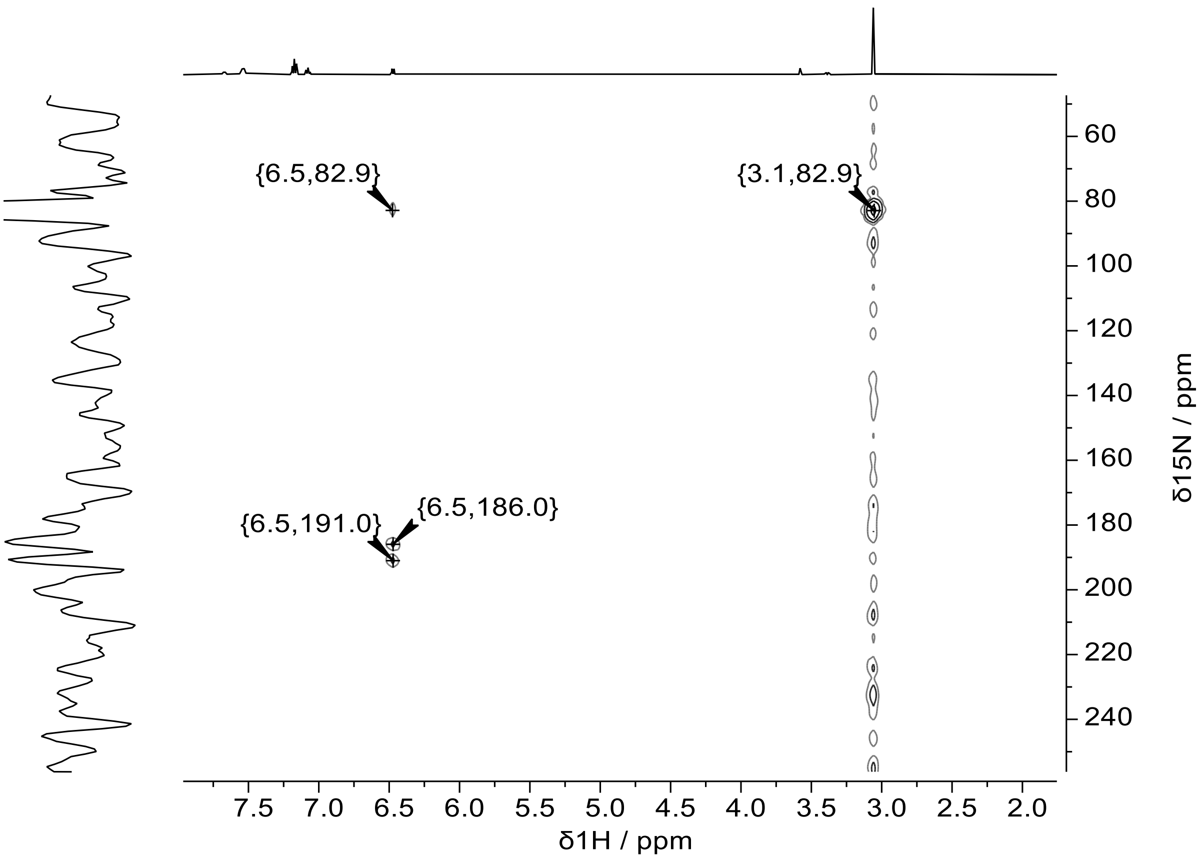

Figure 14: $^{1}H,^{15}N$ HMBC NMR spectrum (500.04 MHz, 50.68 MHz, THF-d_8, 298 K) of complex **$3^{Cr}b$**.

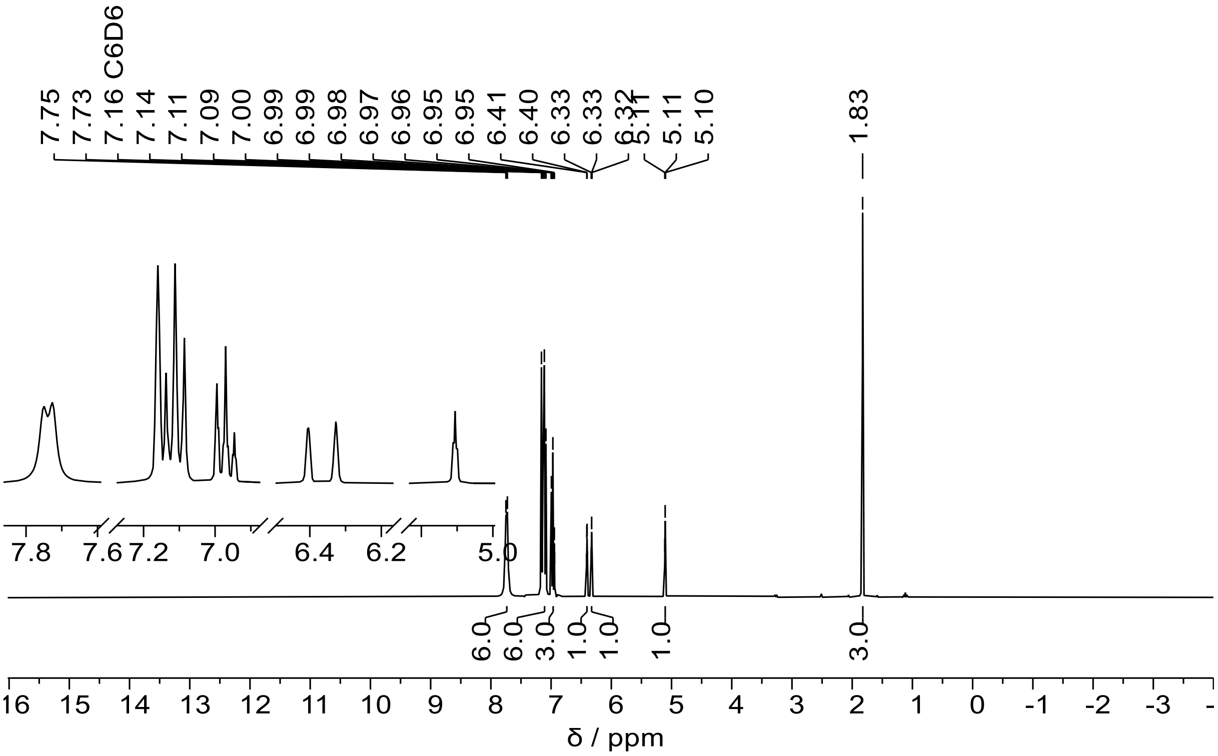

Figure 15: ^{1}H NMR spectrum (300.13 MHz, C_6D_6, 298 K) of complex **3c**.

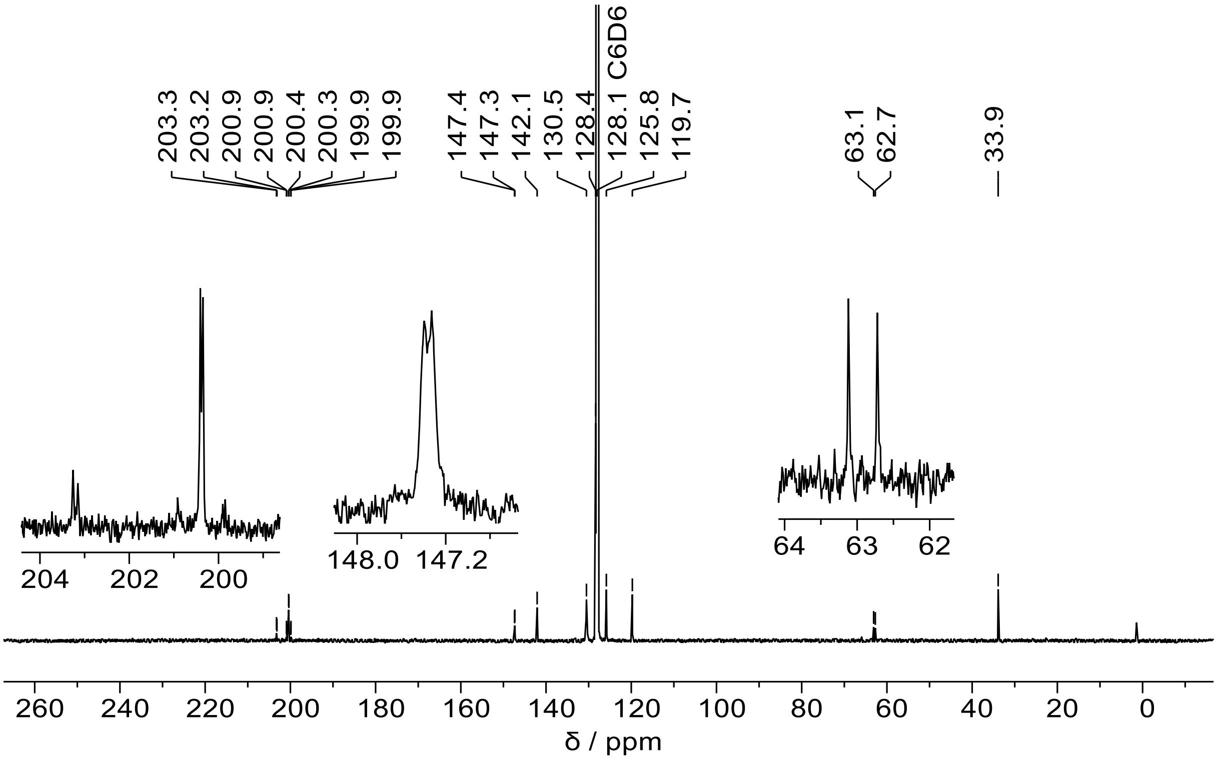

Figure 16: $^{13}C\{^{1}H\}$ NMR spectrum (125.75 MHz, C_6D_6, 298 K) of complex **3c**.

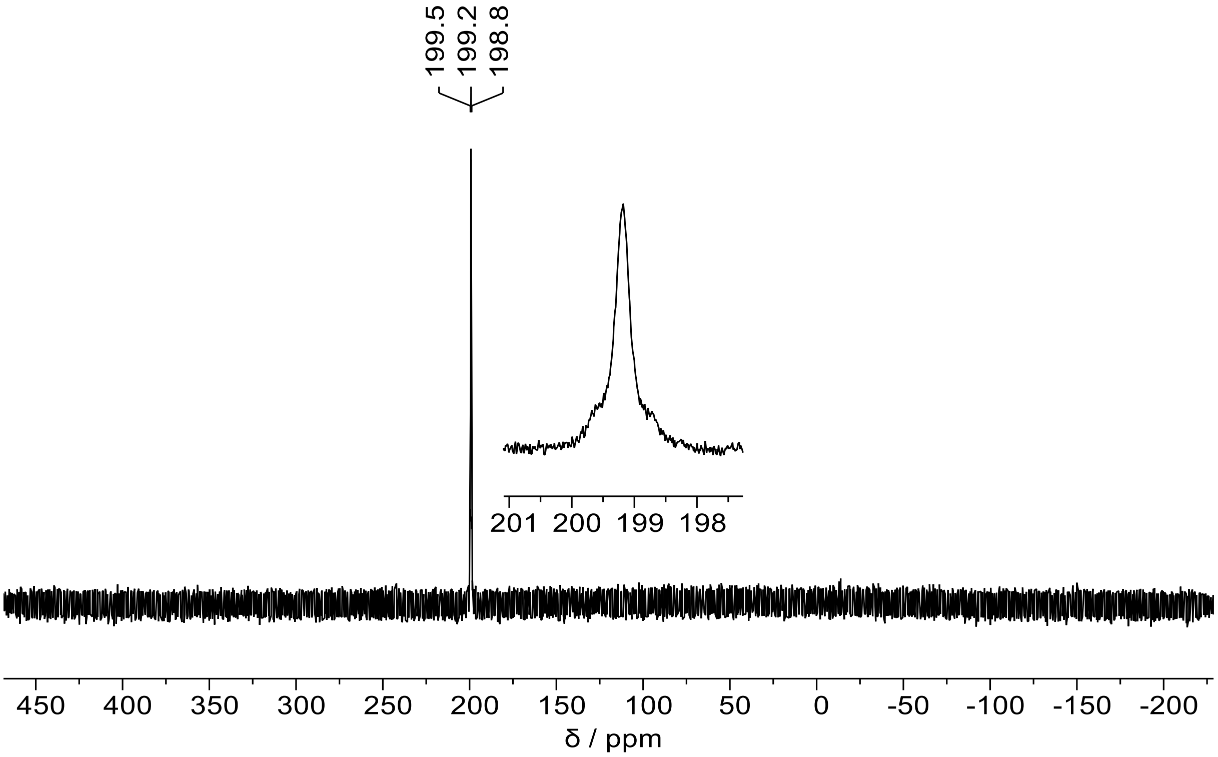

Figure 17: ^{31}P NMR spectrum (121.51 MHz, C_6D_6, 298 K) of complex **3c**.

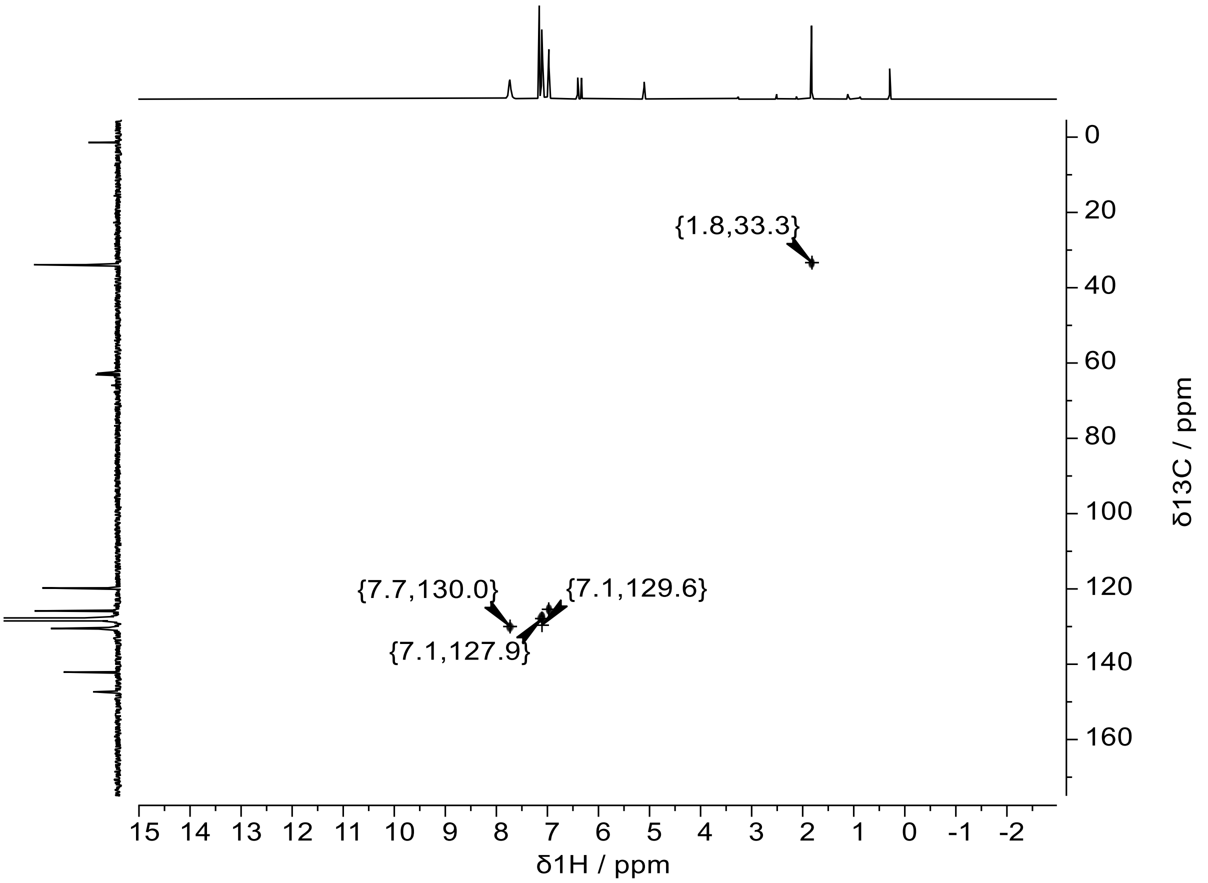

Figure 18: $^{1}H,^{13}C$ HSQC NMR spectrum (500.04 MHz, 125.75 MHz, C_6D_6, 298 K) of complex **3c**.

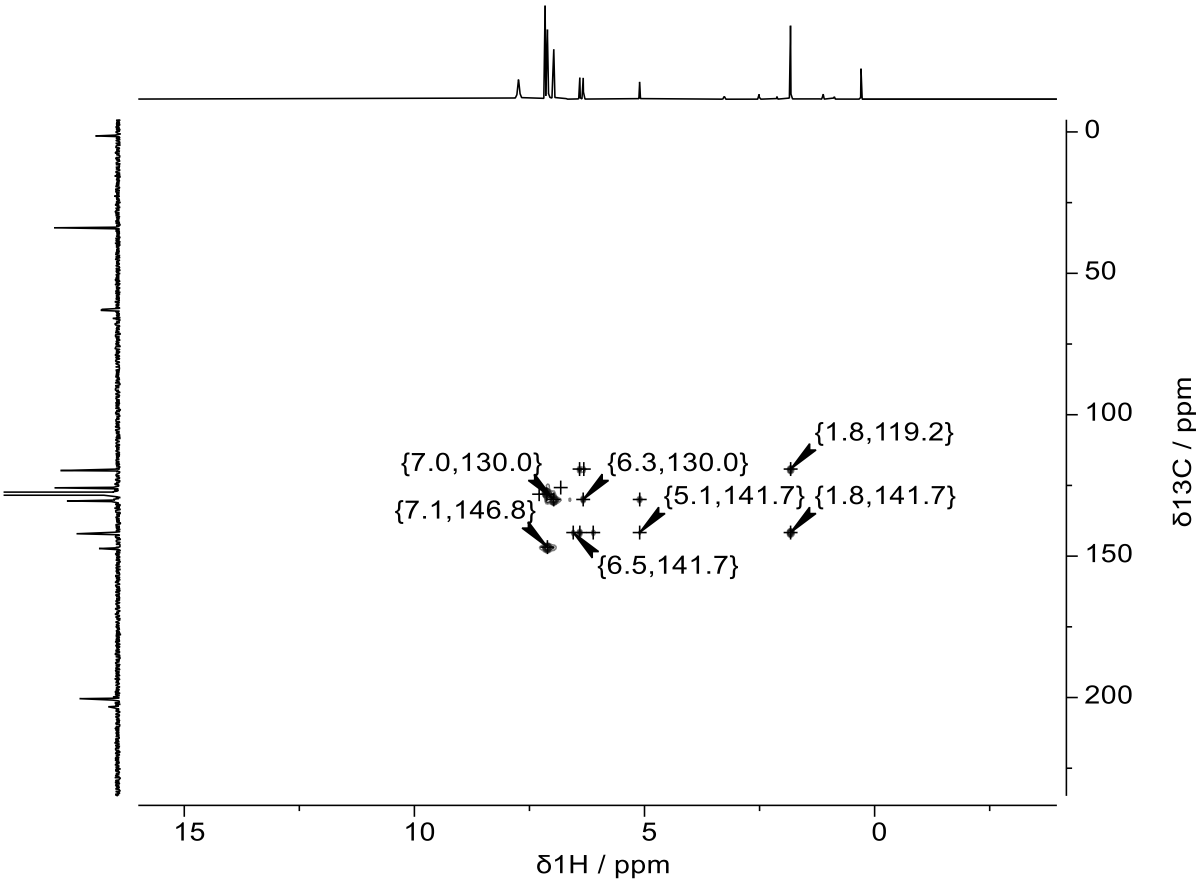

Figure 19: $^{1}H,^{13}C$ HMBC NMR spectrum (500.04 MHz, 125.75 MHz, C_6D_6, 298 K) of complex **3c**.

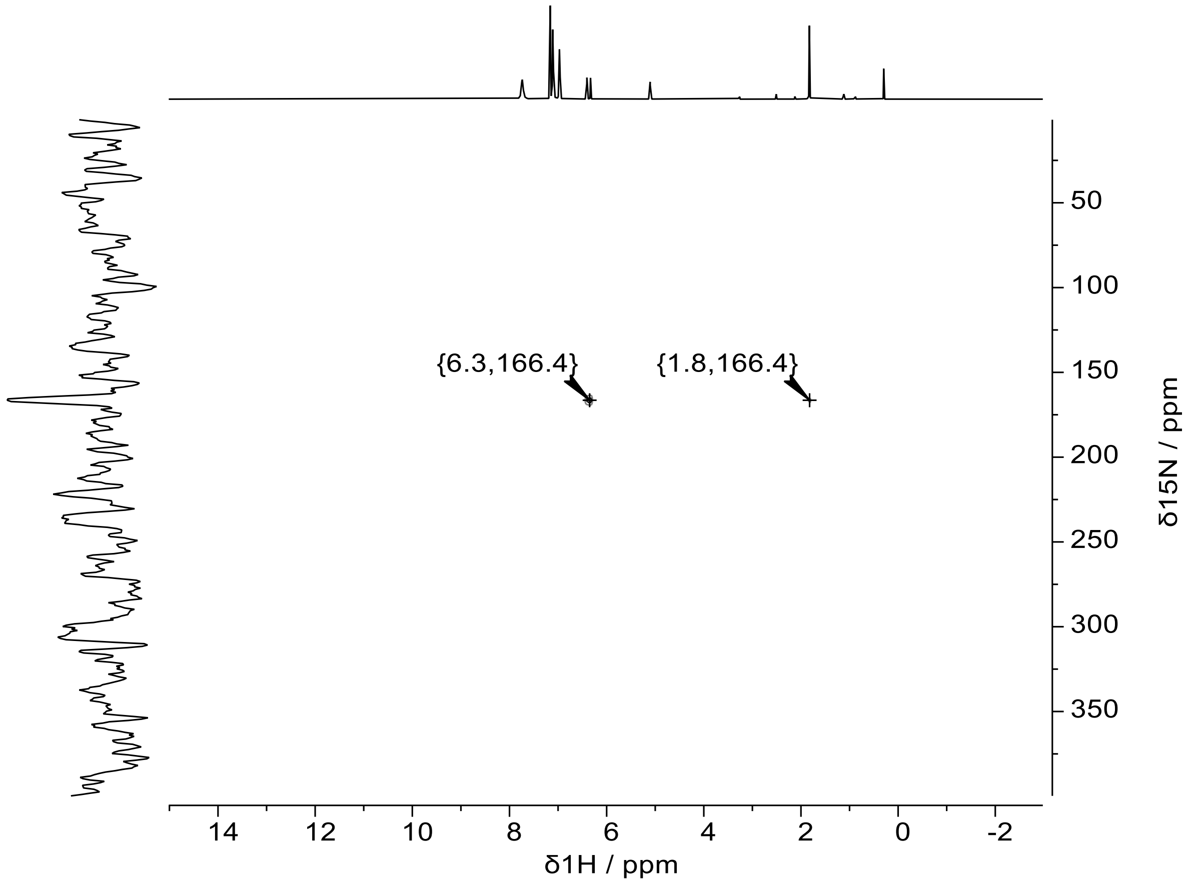

Figure 20: $^{1}H,^{15}N$ HMBC NMR spectrum (500.04 MHz, 50.68 MHz, C_6D_6, 298 K) of complex **3c**.

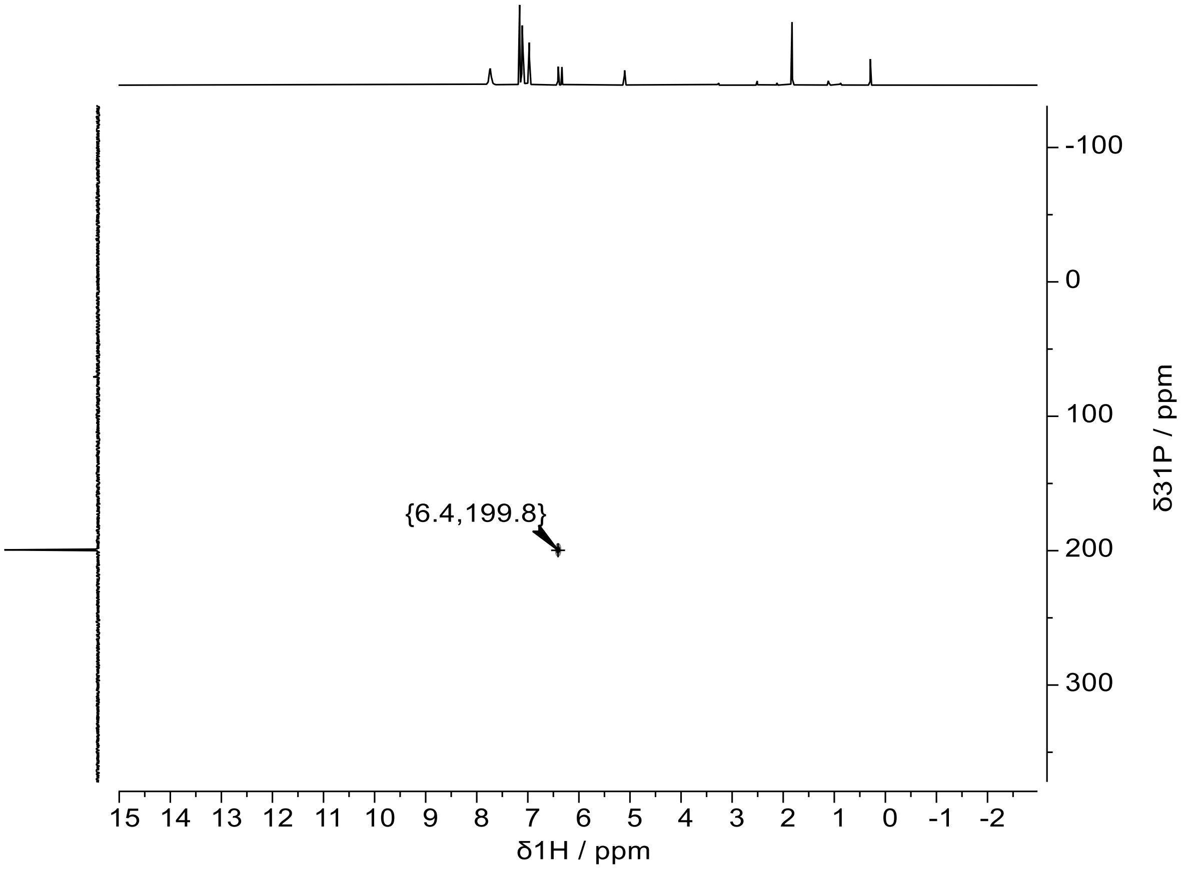

Figure 21: ^{1}H,^{31}P NMR spectrum (500.04 MHz, 202.44 MHz, C_6D_6, 298 K) of complex **3c**.

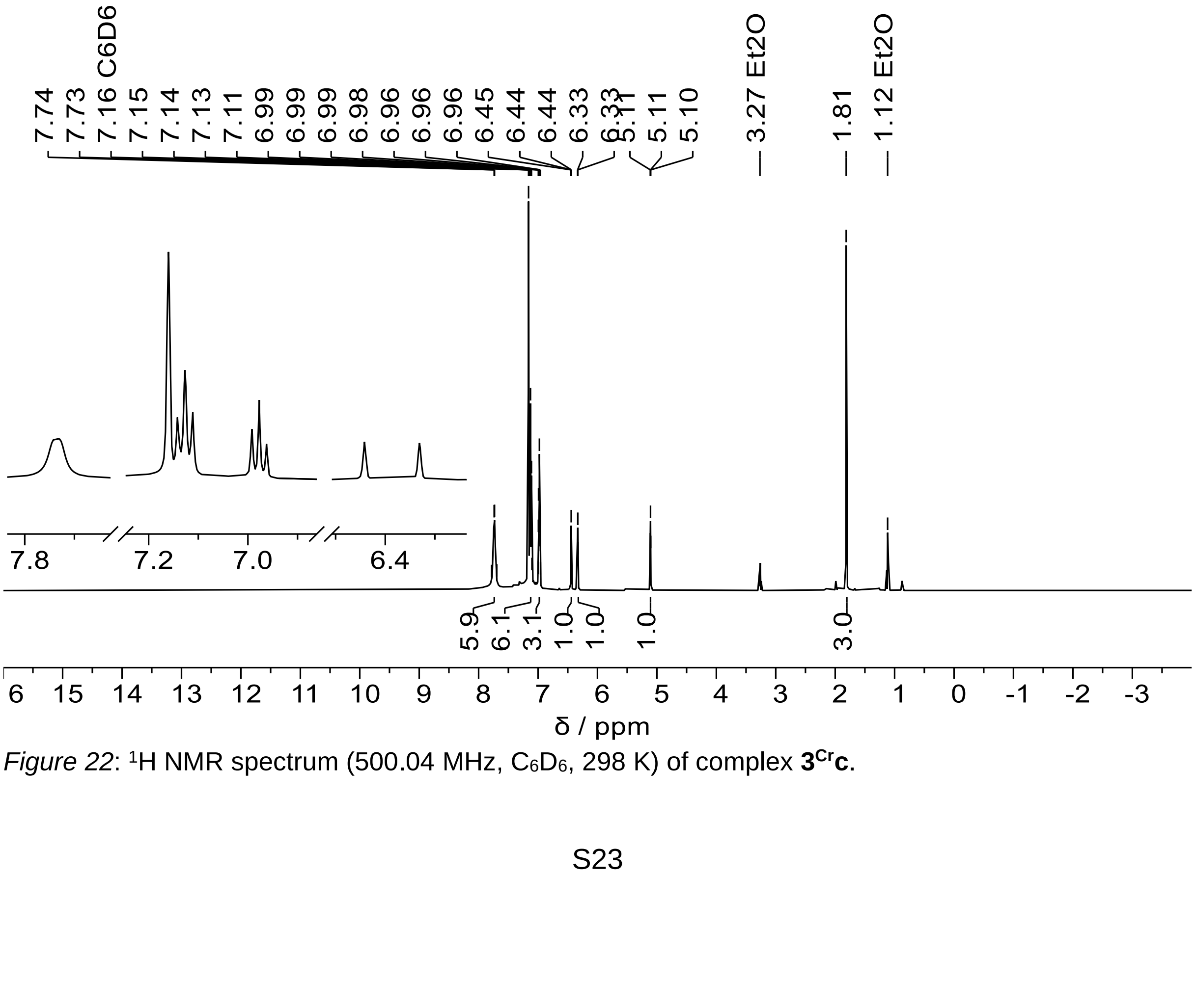

Figure 22: ^{1}H NMR spectrum (500.04 MHz, C_6D_6, 298 K) of complex **3^{Cr}c**.

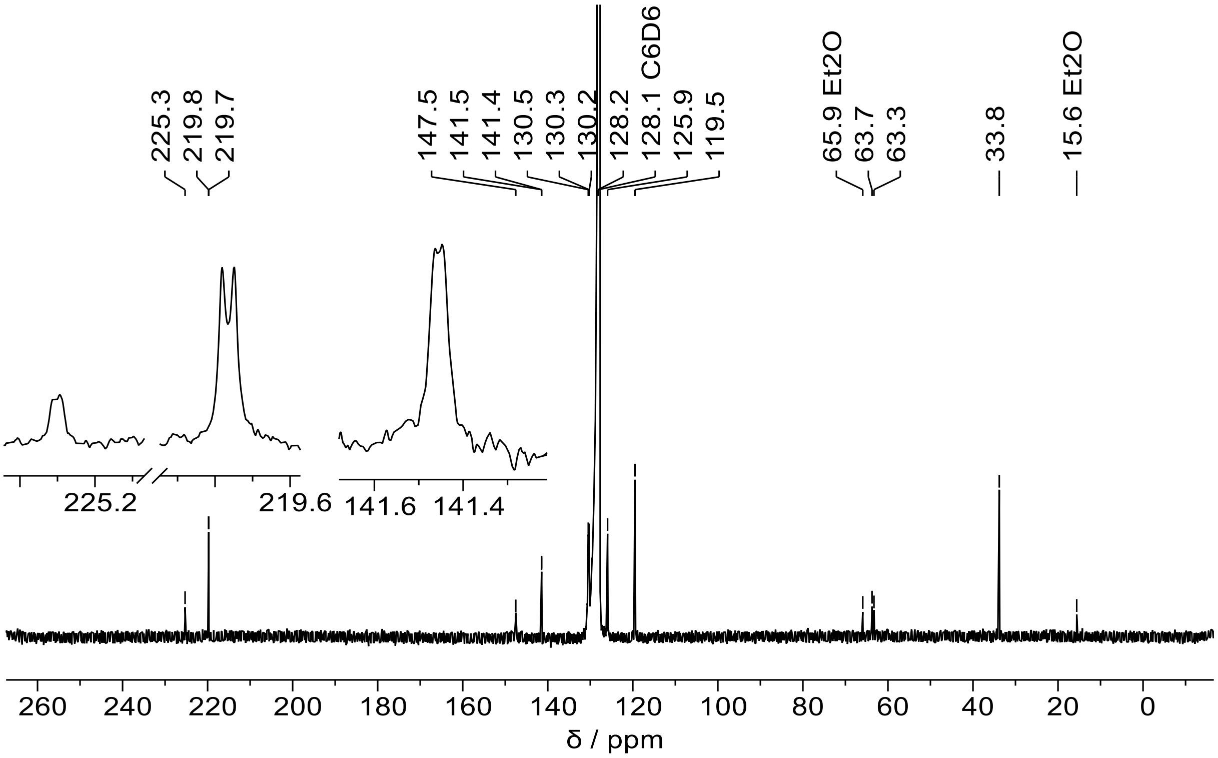

Figure 23: $^{13}C\{^1H\}$ NMR spectrum (125.75 MHz, C_6D_6, 298 K) of complex $\mathbf{3^{Cr}c}$.

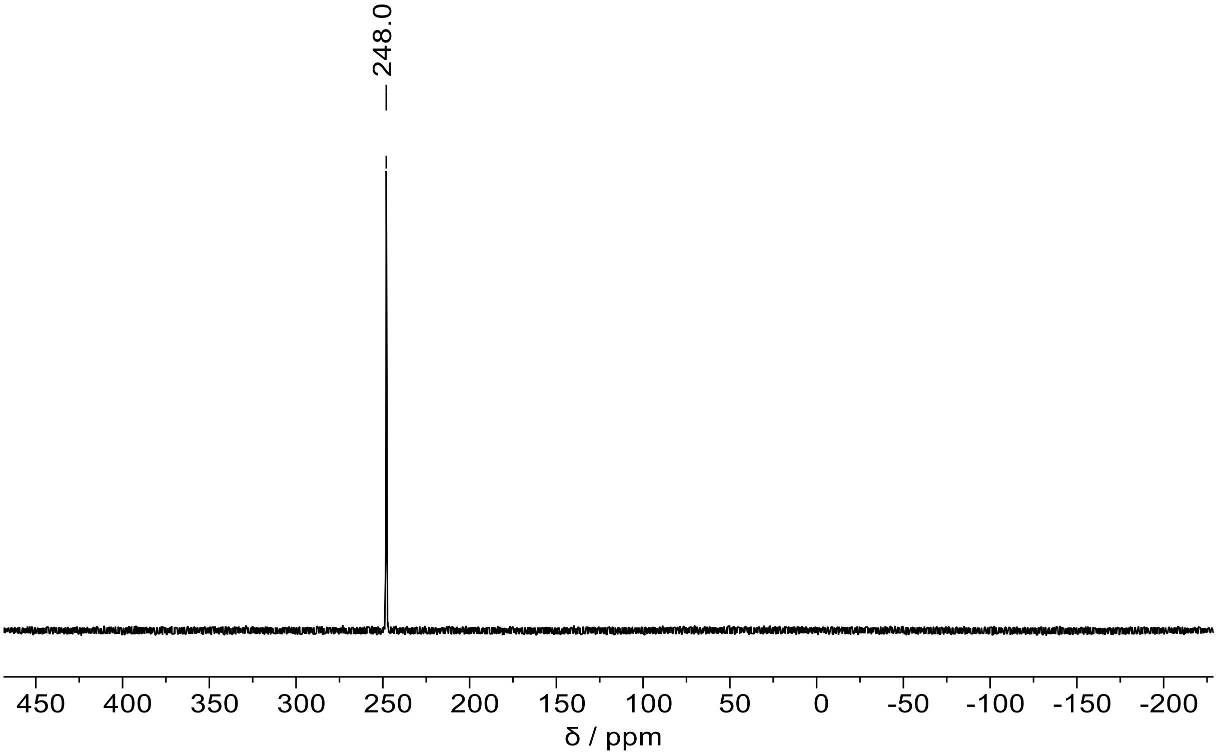

Figure 24: ^{31}P NMR spectrum (121.51 MHz, C_6D_6, 299 K) of complex $\mathbf{3^{Cr}c}$.

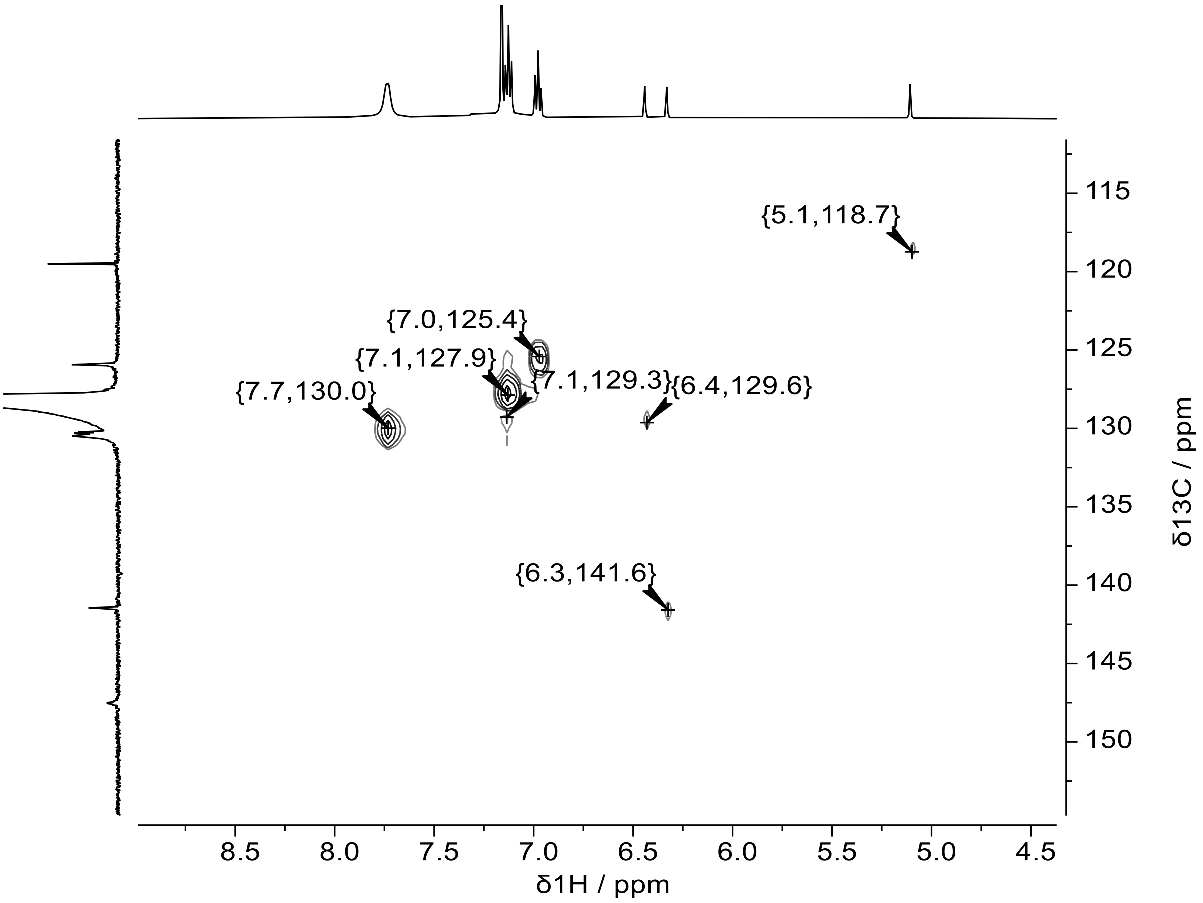

Figure 25: $^{1}H,^{13}C$ HSQC NMR spectrum (500.04 MHz, 125.75 MHz, C_6D_6, 298 K) of complex $\mathbf{3^{Cr}c}$.

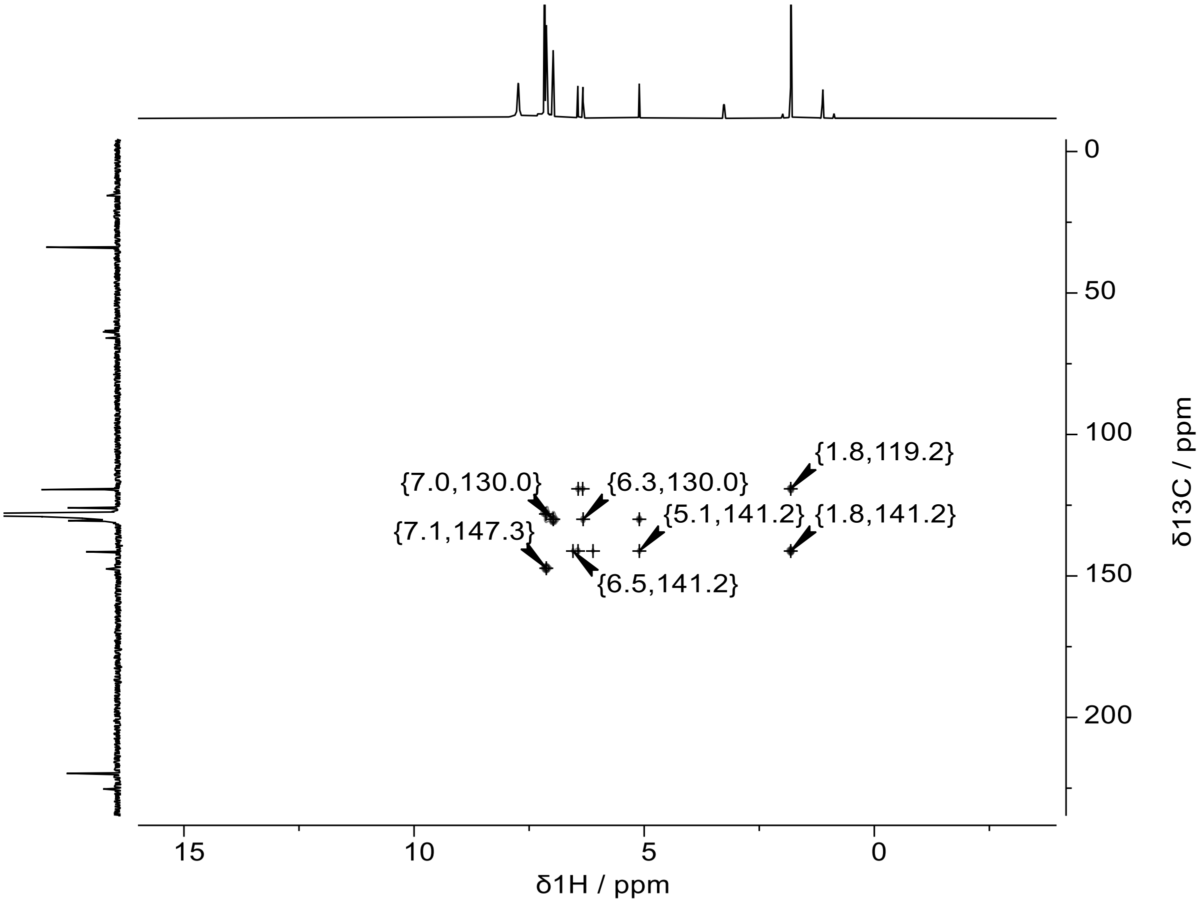

Figure 26: $^{1}H,^{13}C$ HMBC NMR spectrum (500.04 MHz, 125.75 MHz, C_6D_6, 298 K) of complex **3^{Cr}c**.

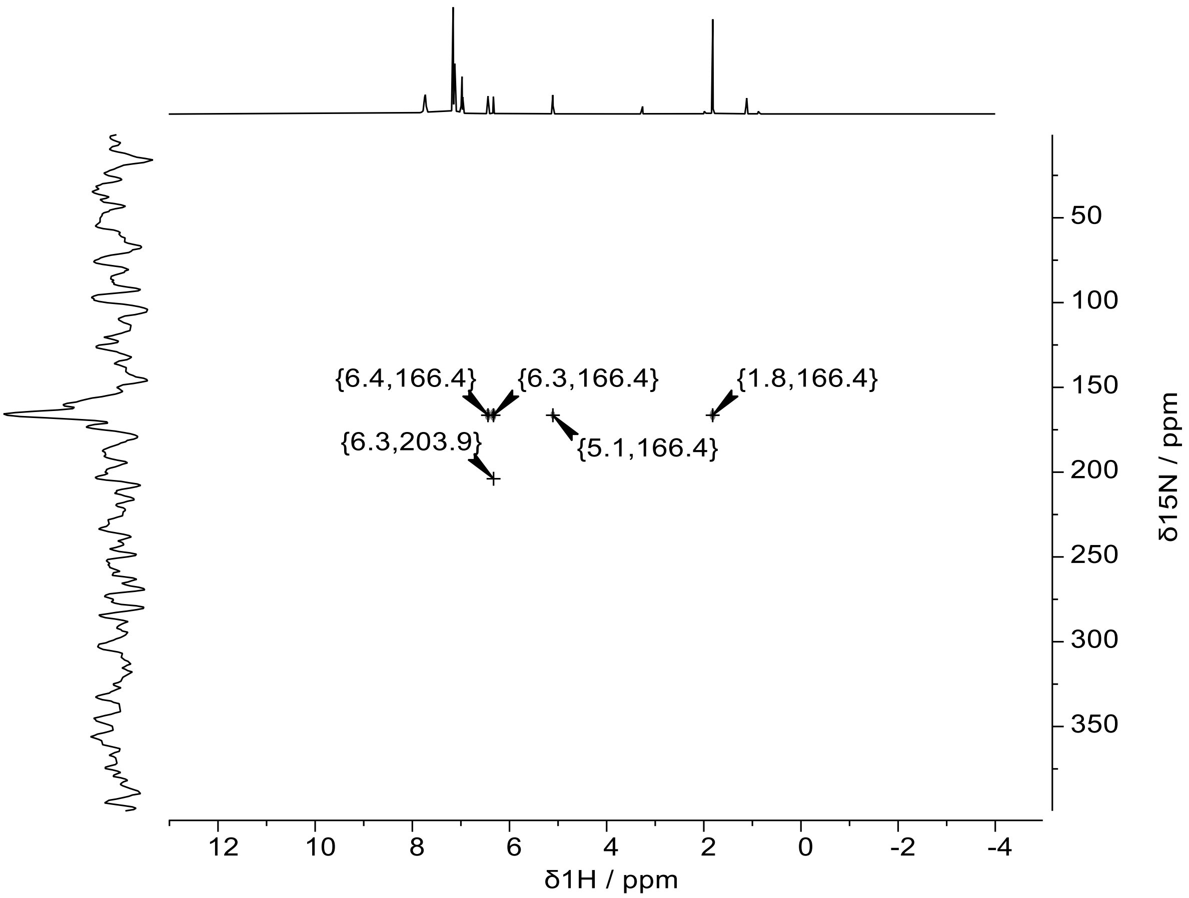

Figure 27: $^{1}H,^{15}N$ HMBC NMR spectrum (500.04 MHz, 50.68 MHz, C_6D_6, 298 K) of complex $\mathbf{3^{Cr}c}$.

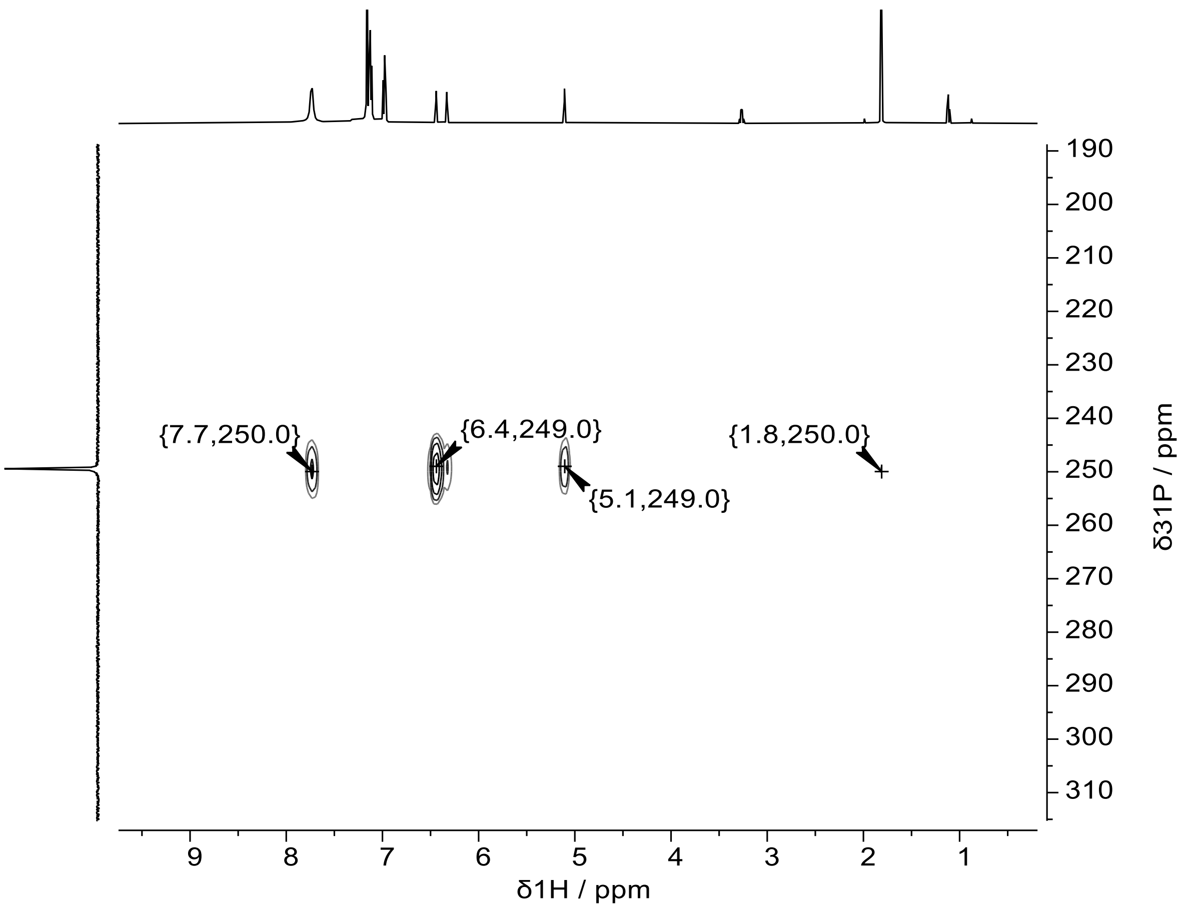

Figure 28: ^{1}H,^{31}P HMBC NMR spectrum (500.04 MHz, 202.44 MHz, C_6D_6, 298 K) of complex $\mathbf{3^{Cr}c}$.

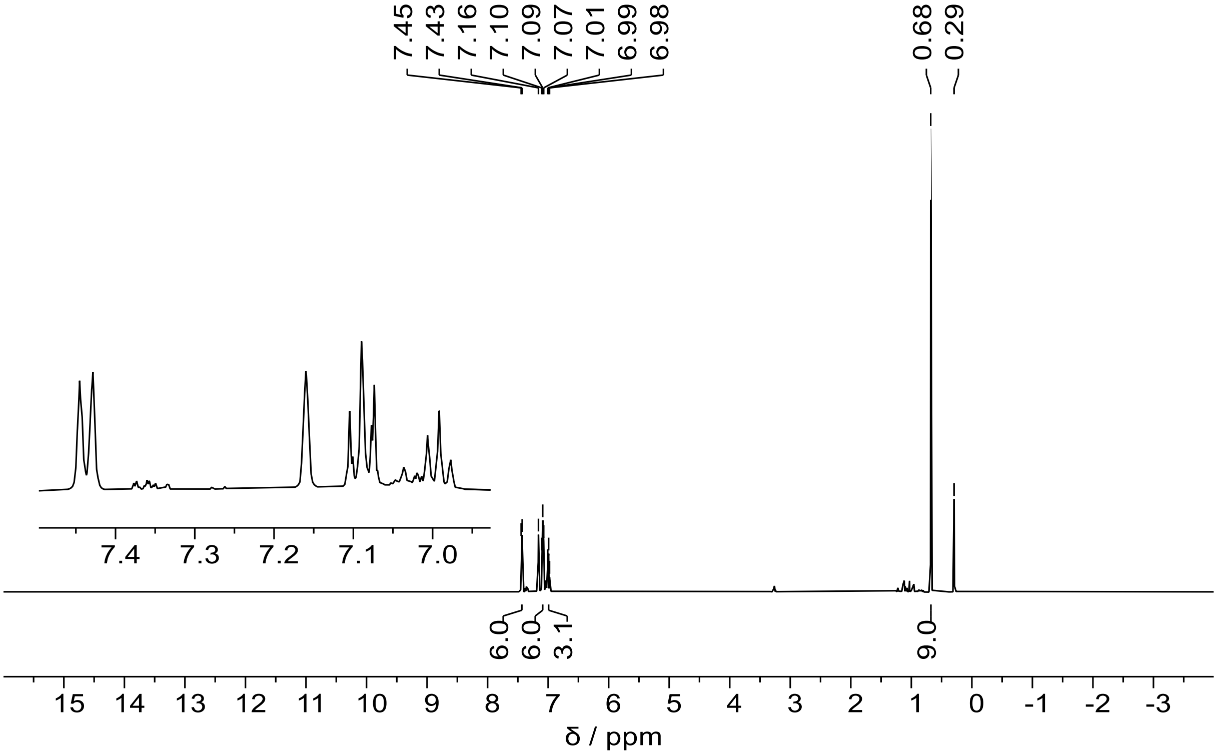

Figure 29: ^{1}H NMR (500.14 MHz, C_6D_6, 298 K) of complex **3d**.

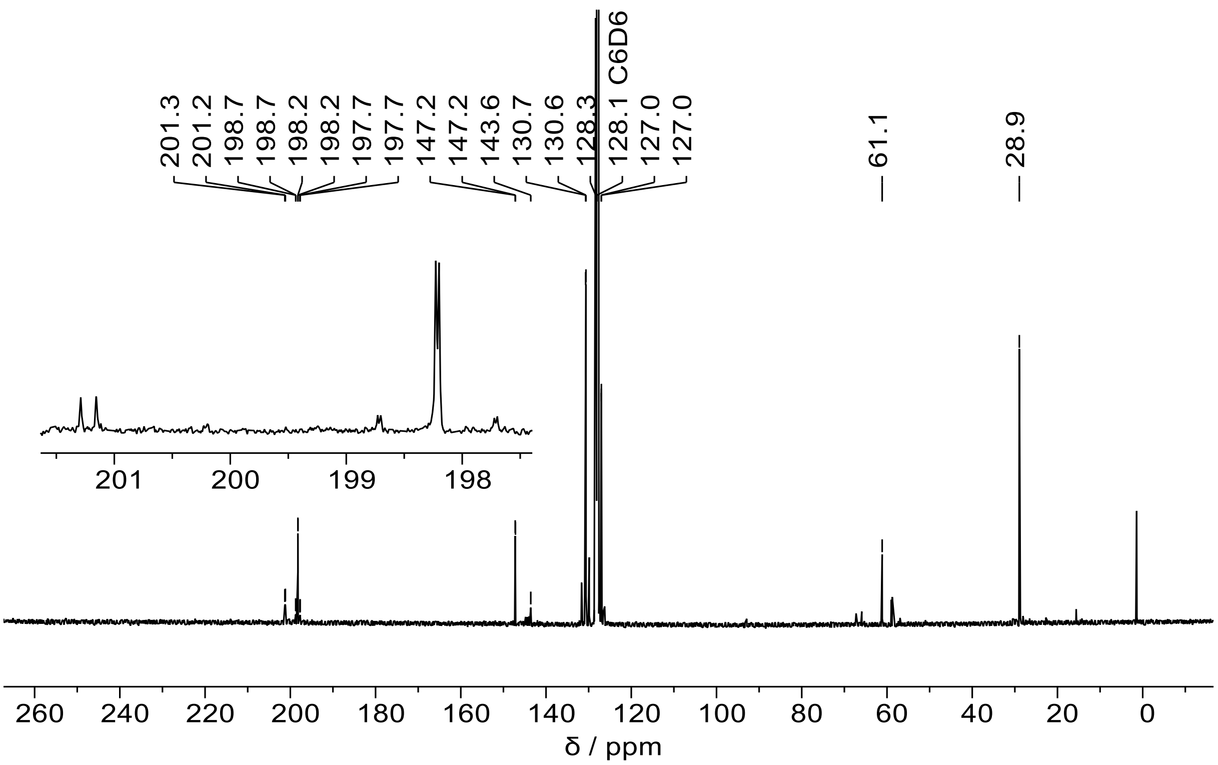

Figure 30: $^{13}C\{^{1}H\}$ NMR spectrum (125.78 MHz, C_6D_6, 298 K) of complex **3d**.

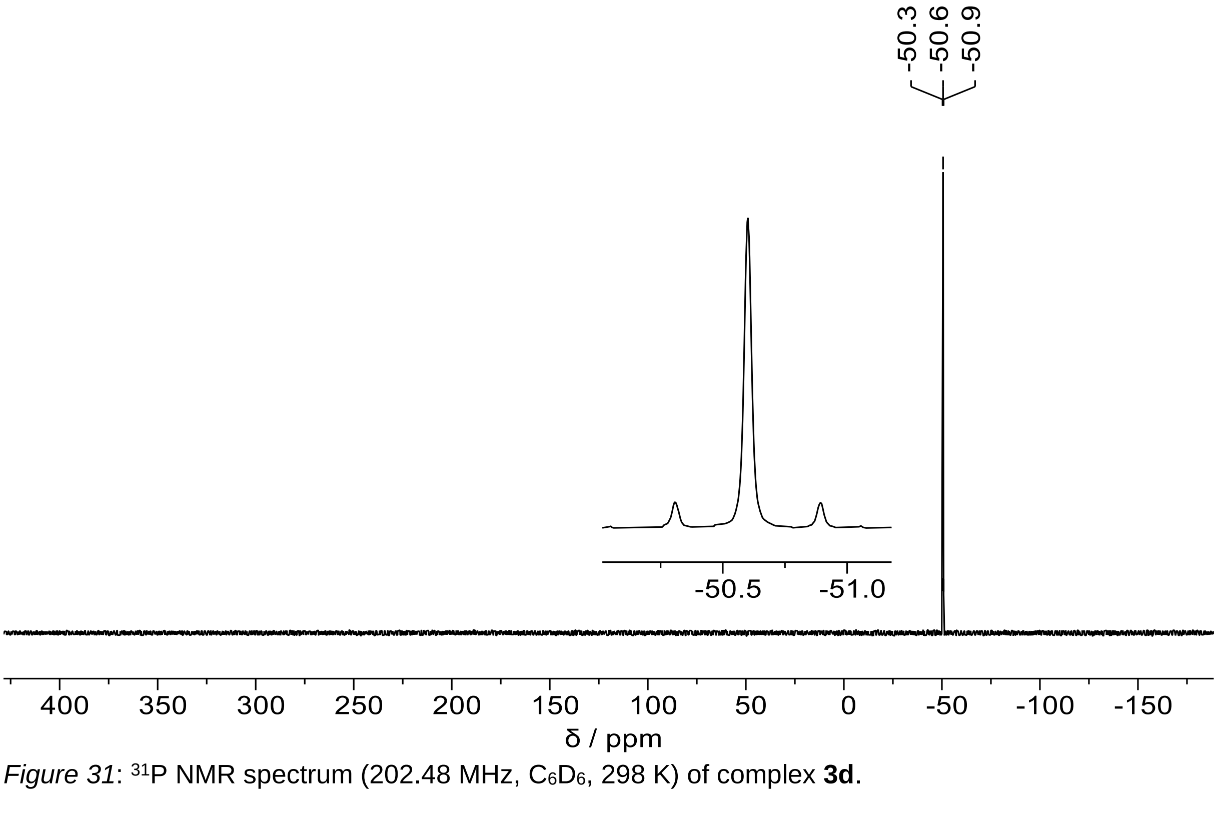

Figure 31: ^{31}P NMR spectrum (202.48 MHz, C_6D_6, 298 K) of complex **3d**.

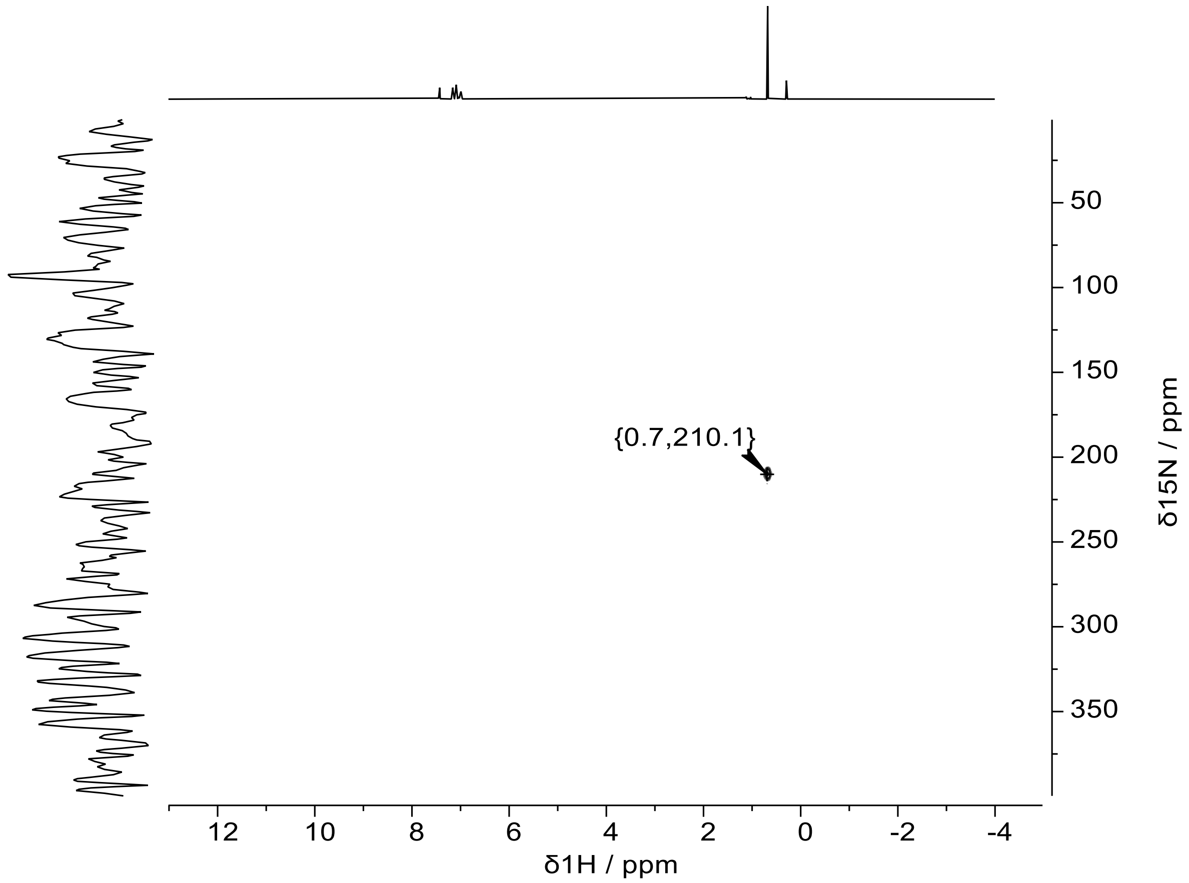

Figure 32: $^{1}H,^{15}N$ HMBC NMR spectrum (500.14 MHz, 50.69 MHz, C_6D_6, 298 K) of spectrum **3d**.

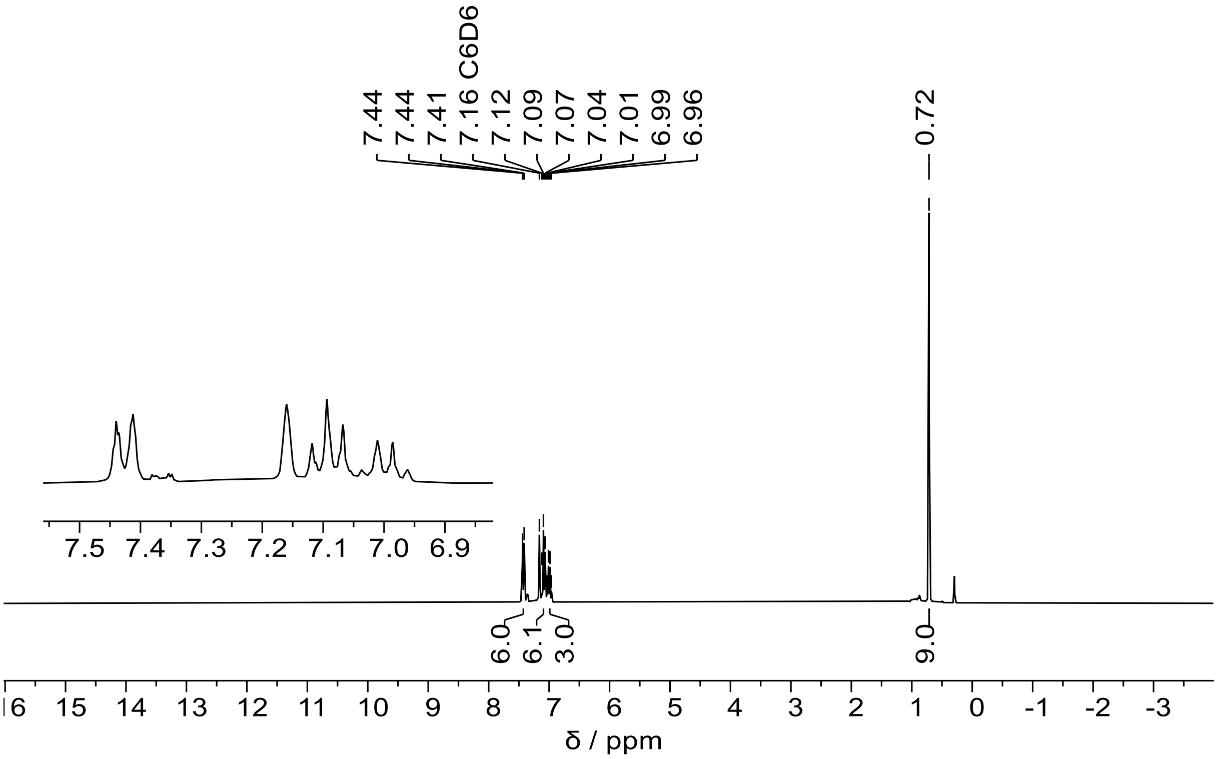

Figure 33: ^{1}H NMR spectrum (300.13 MHz, C_6D_6, 300 K) of complex **3^{Cr}d**.

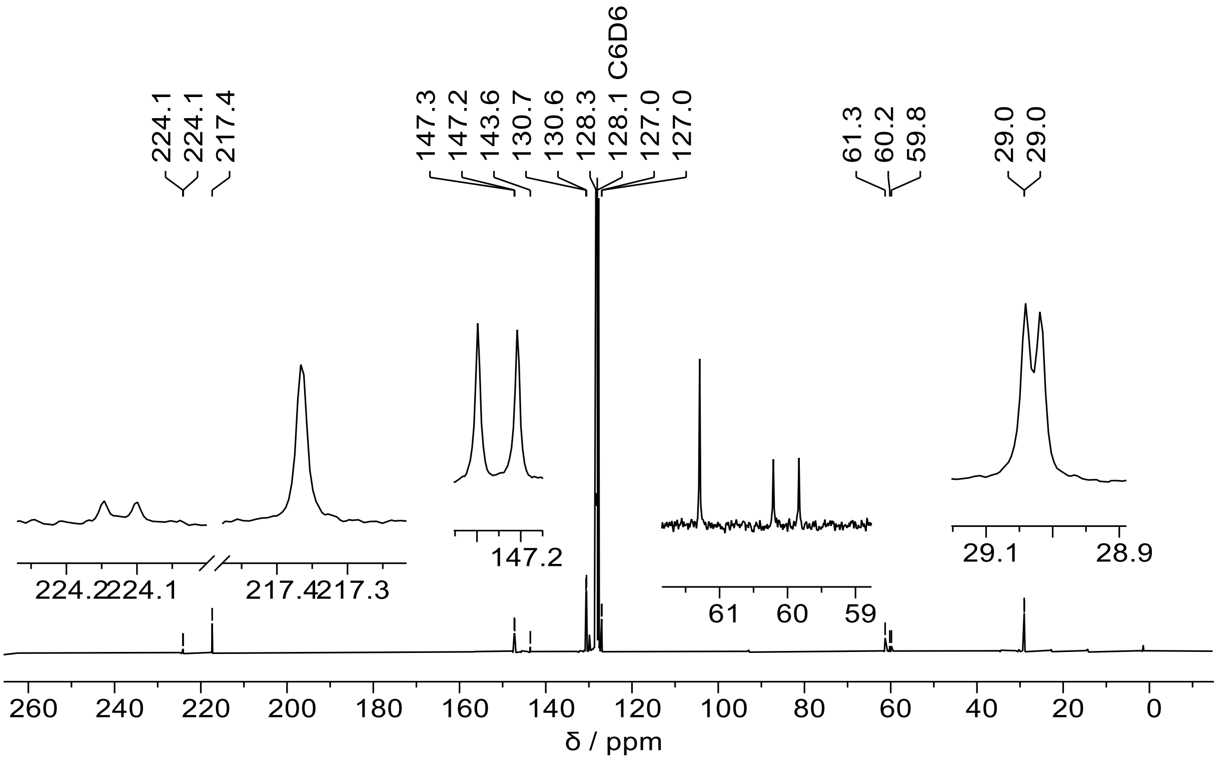

Figure 34: $^{13}C\{^{1}H\}$ NMR (75.48 MHz, C_6D_6, 300 K) of complex $\mathbf{3^{Cr}d}$.

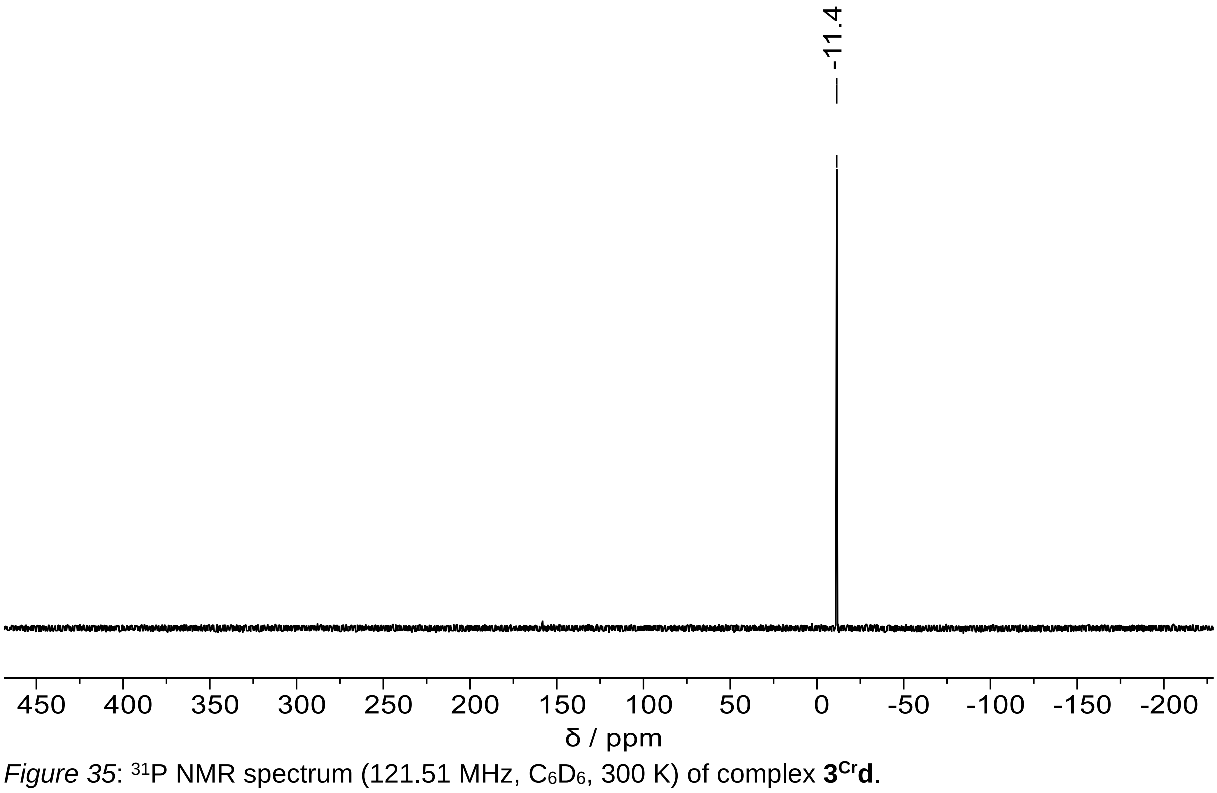

Figure 35: ^{31}P NMR spectrum (121.51 MHz, C_6D_6, 300 K) of complex $\mathbf{3^{Cr}d}$.

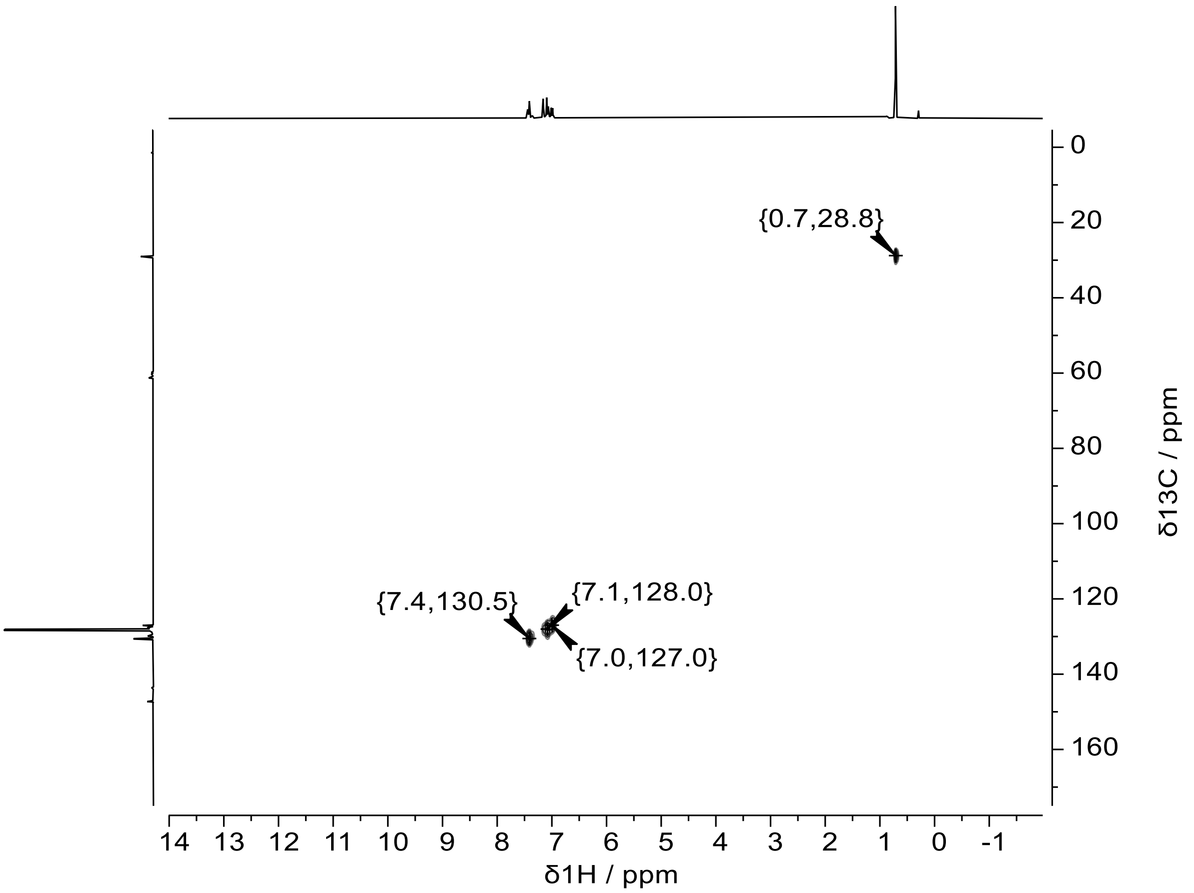

Figure 36: ^{1}H,^{13}C HSQC NMR spectrum (300.13 MHz, 75.47 MHz, C_6D_6, 300 K) of complex **3^{Cr}d**.

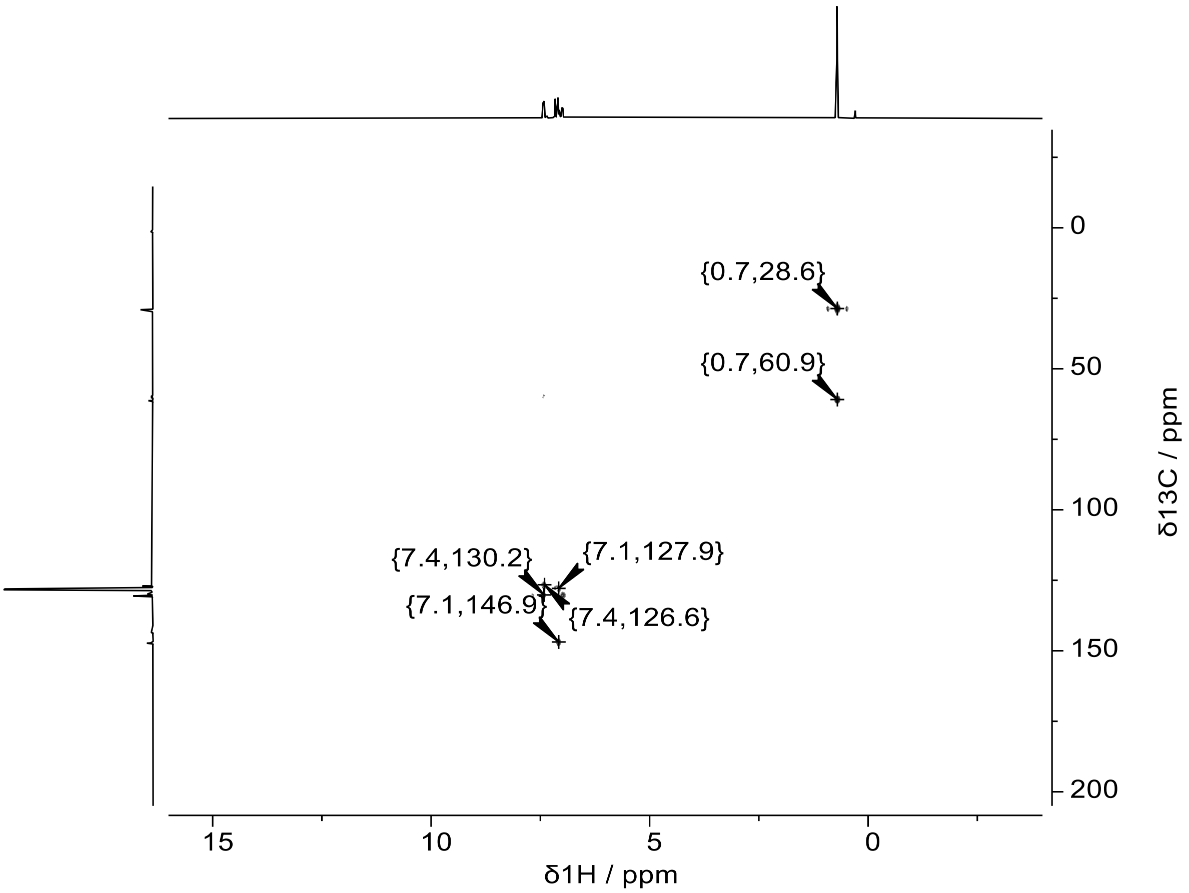

Figure 37: $^{1}H,^{13}C$ HMBC NMR spectrum (300.13 MHz, 75.47 MHz, C_6D_6, 299 K) of complex **3^{Cr}d**.

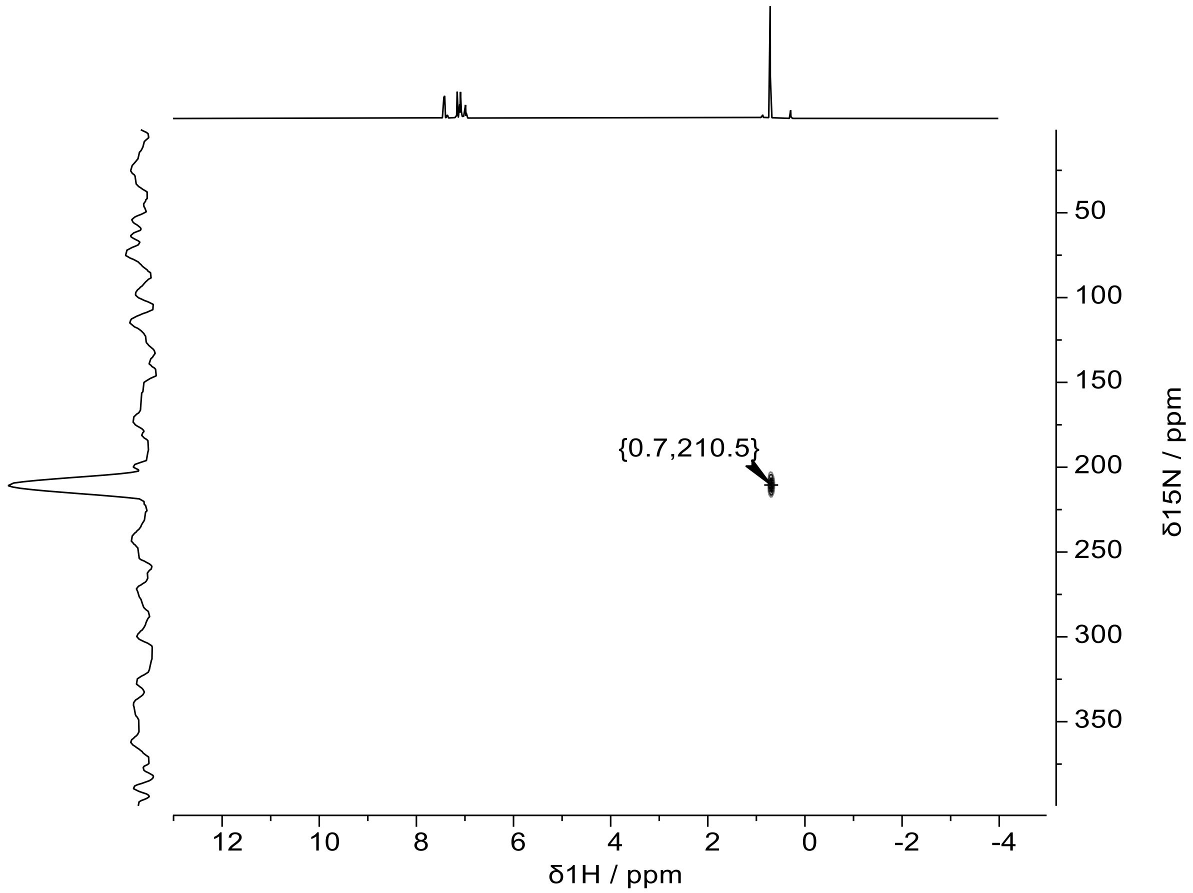

Figure 38: $^{1}H,^{15}N$ HMBC NMR spectrum (500.14 MHz, 50.69 MHz, C_6D_6, 298 K) of complex $\mathbf{3^{Cr}d}$.

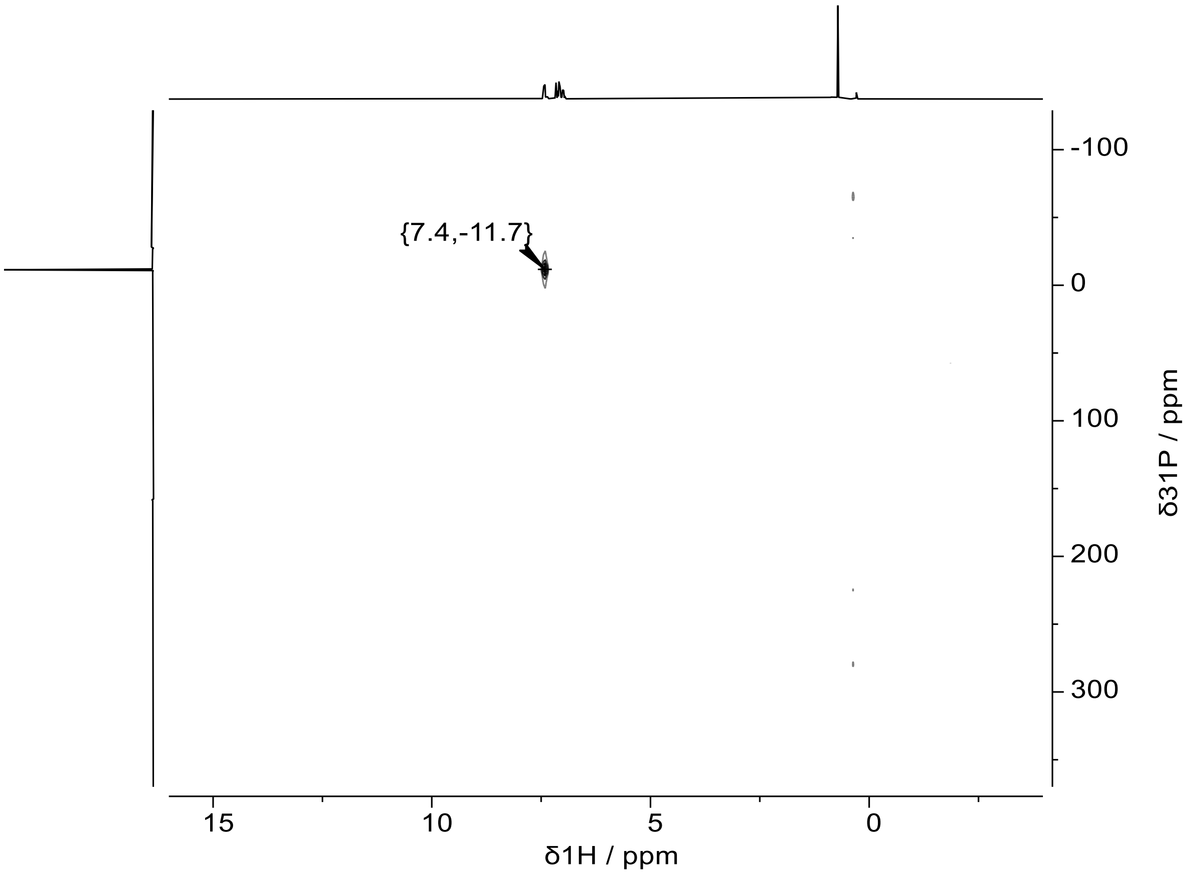

Figure 39: $^{1}H,^{31}P$ HMBC NMR spectrum (300.13 MHz, 121.51 MHz, C_6D_6, 300 K) of complex $\mathbf{3^{Cr}d}$.

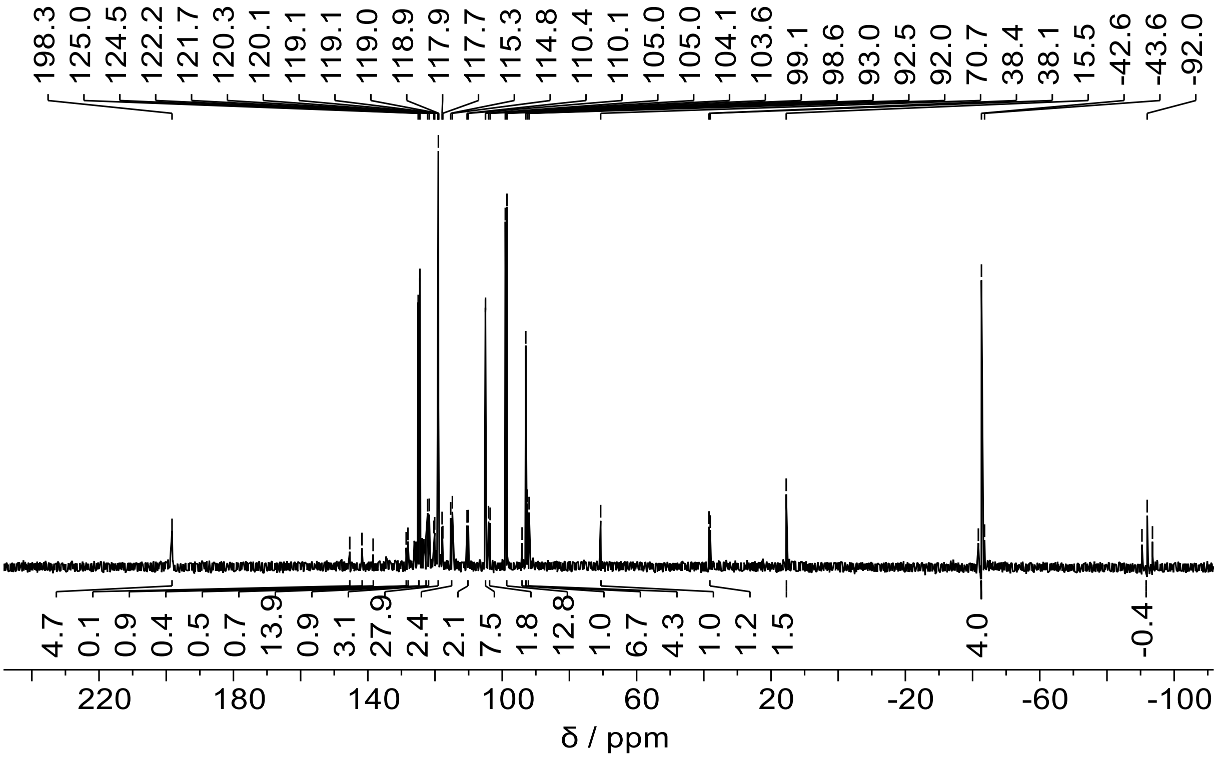

Figure 40: In situ $^{31}P\{^{1}H\}$ NMR spectrum (121.51 MHz, C_6D_6, 298 K) of the reaction mixture of complex **3c** with 10 bars of CO after heating at 70 °C for 21 hours. Signal at 198.3 ppm is assigned to the starting

material **3c**, at 43.6 ppm to [W(CO)$_5${P(CPh$_3$)H$_2$}] and at −92.0 ppm to the terminal P-nucleus of butterfly compound **7** as thermal decomposition product of **3c**.

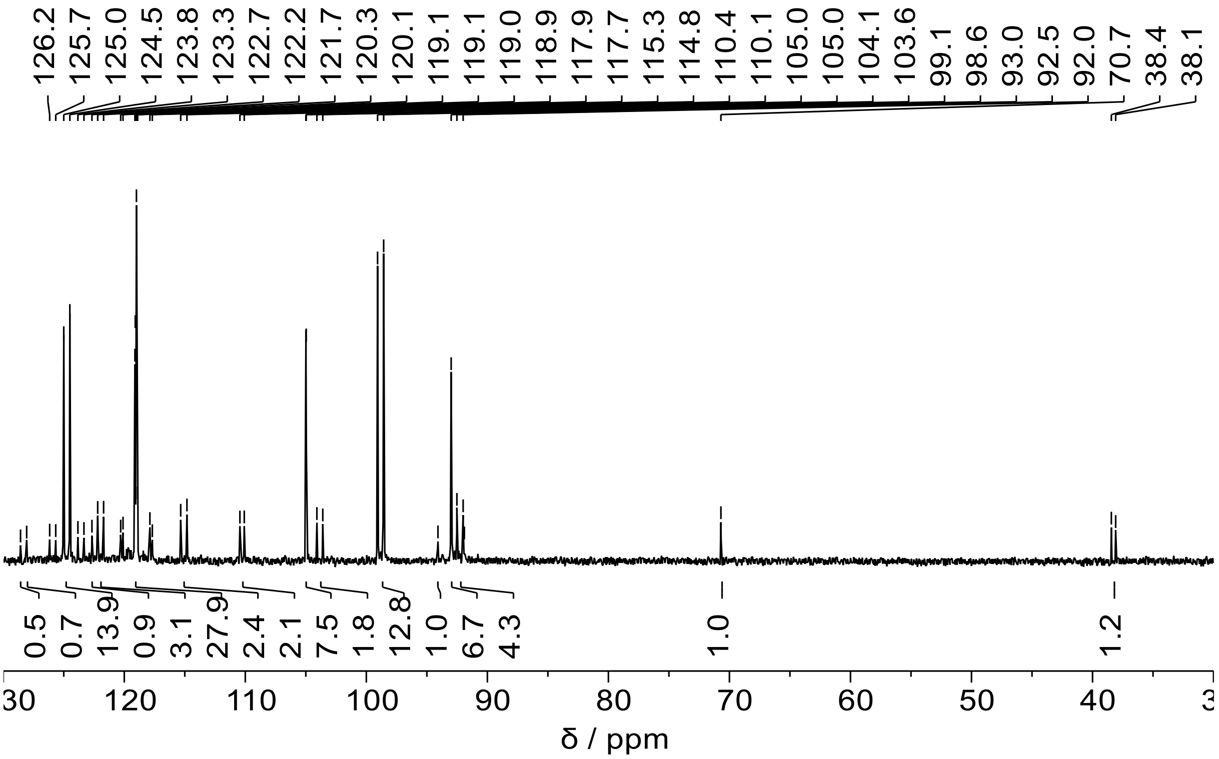

Figure 41: Zoom (130 to 30 ppm) into the in situ $^{31}P\{^1H\}$ NMR spectrum (121.51 MHz, C_6D_6, 298 K) of the reaction mixture of complex **3c** with 10 bars of CO after heating at 70 °C for 21 hours.

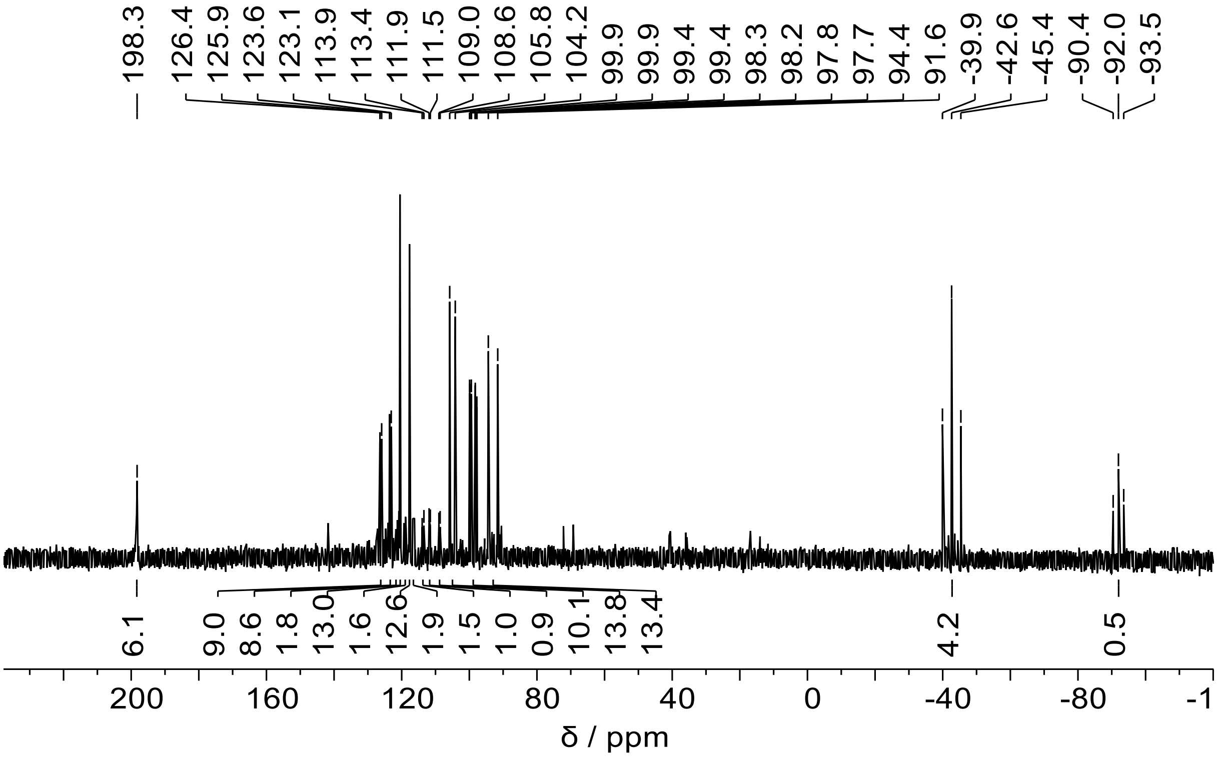

Figure 42: In situ ^{31}P NMR spectrum (121.51 MHz, C_6D_6, 298 K) of the reaction mixture of complex **3c** with 10 bars of CO after heating at 70 °C for 21 hours.

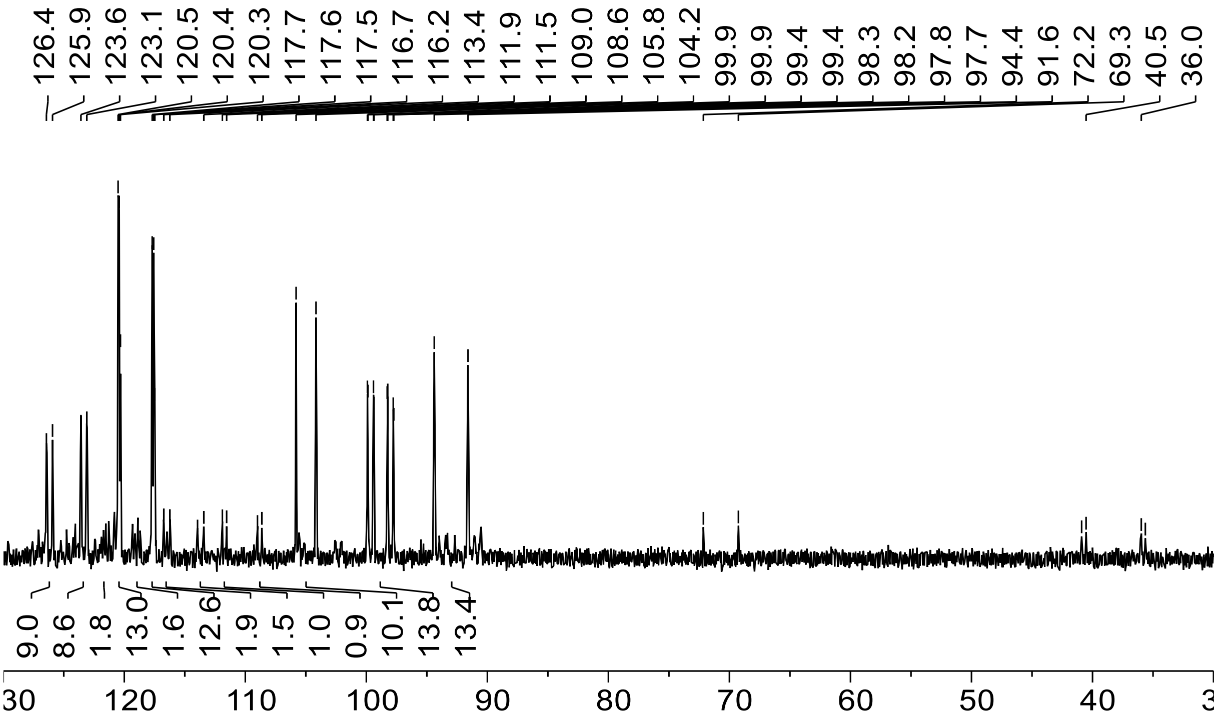

Figure 43: Zoom (130 to 30 ppm) into the in situ ^{31}P NMR spectrum (121.51 MHz, C_6D_6, 298 K) of the reaction mixture of complex **3c** with 10 bars of CO after heating at 70 °C for 21 hours.

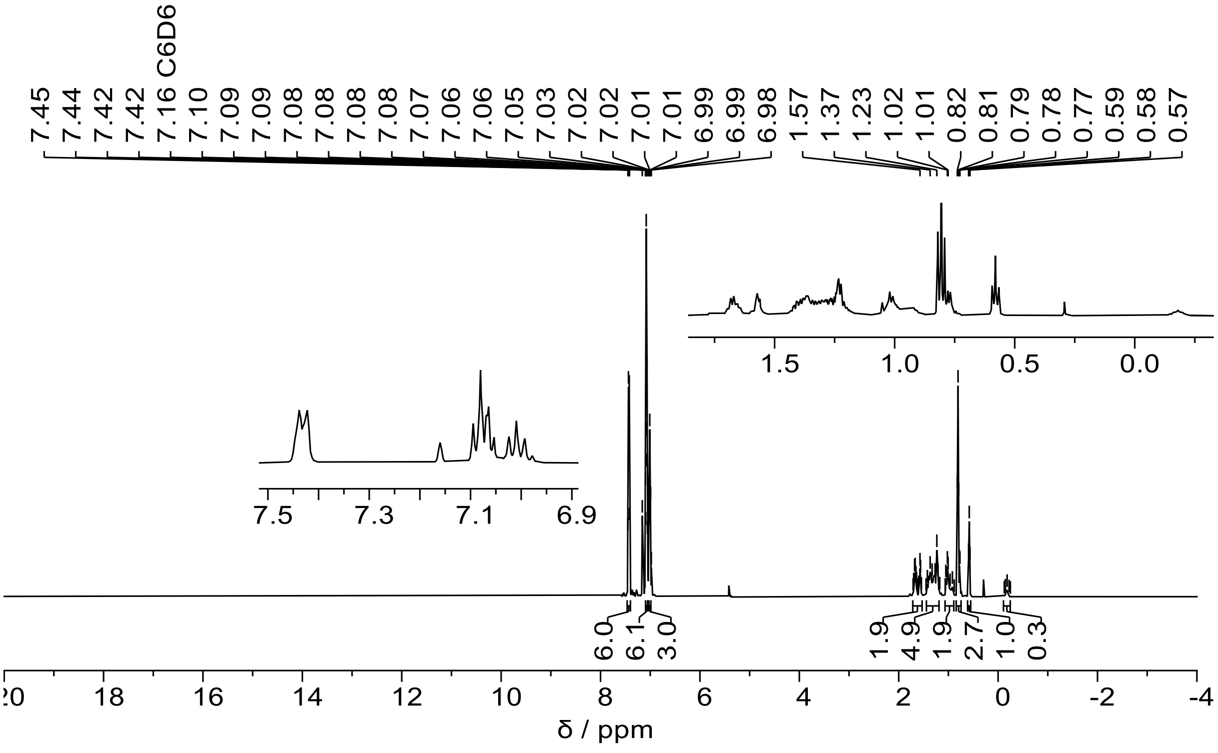

Figure 44: ^1H NMR spectrum (500.04 MHz, C_6D_6, 298 K) of complex **10a**.

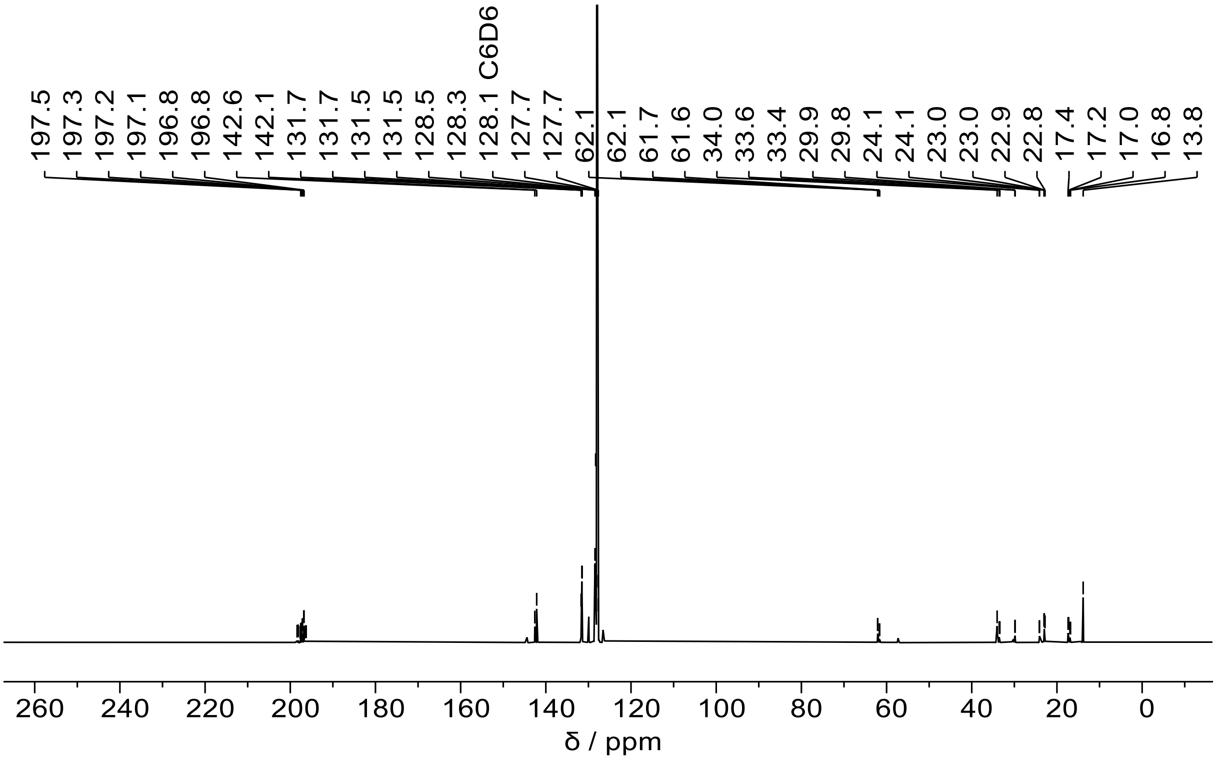

Figure 45: $^{13}C\{^{1}H\}$ NMR spectrum (125.75 MHz, C_6D_6, 298 K) of complex **10a**.

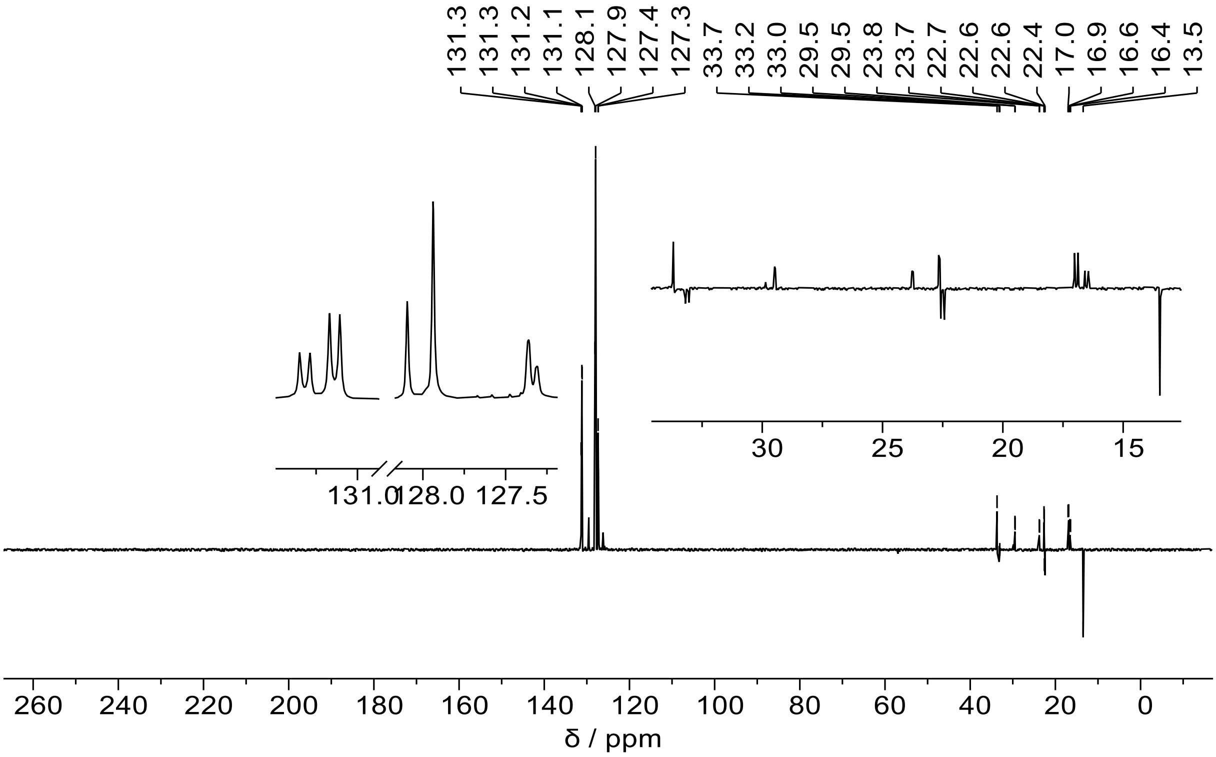

Figure 46: $^{13}C\{^{1}H\}$ DEPT135 NMR spectrum (125.75 MHz, C_6D_6, 298 K) of complex **10a**.

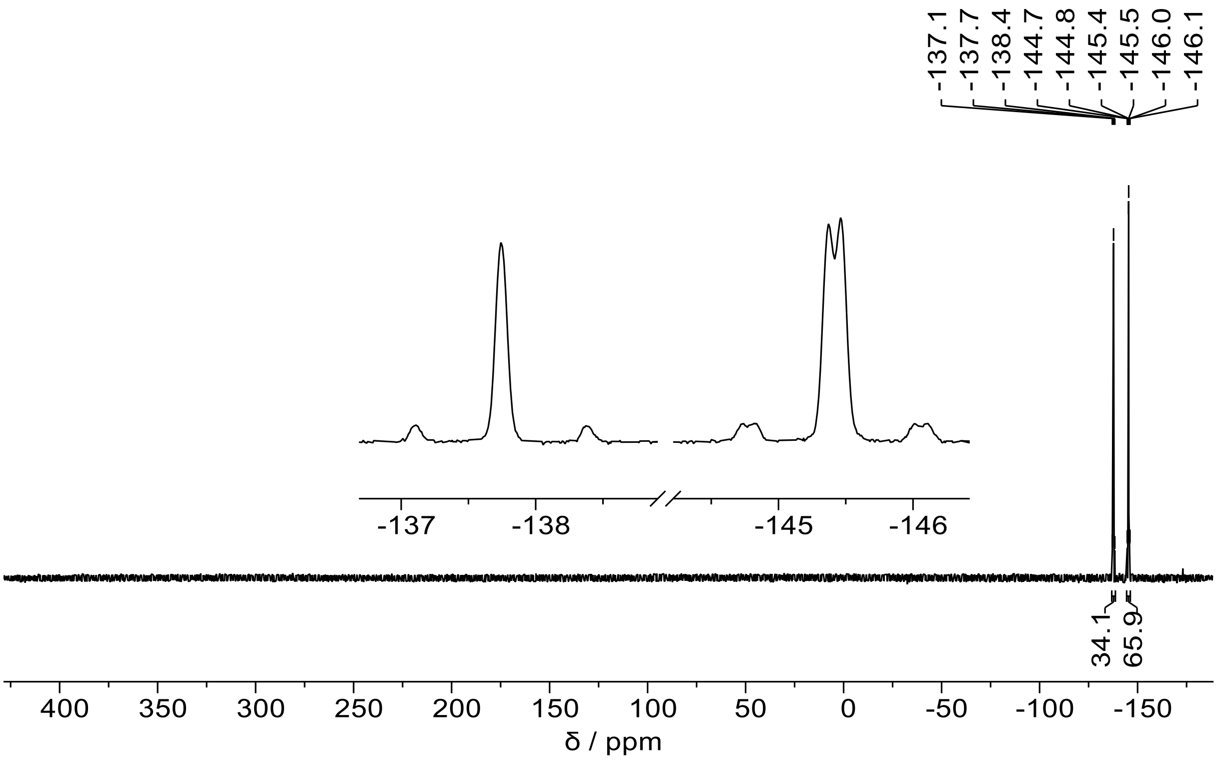

Figure 47: ^{31}P NMR spectrum (202.44 MHz, C_6D_6, 298 K) of complex **10a**.

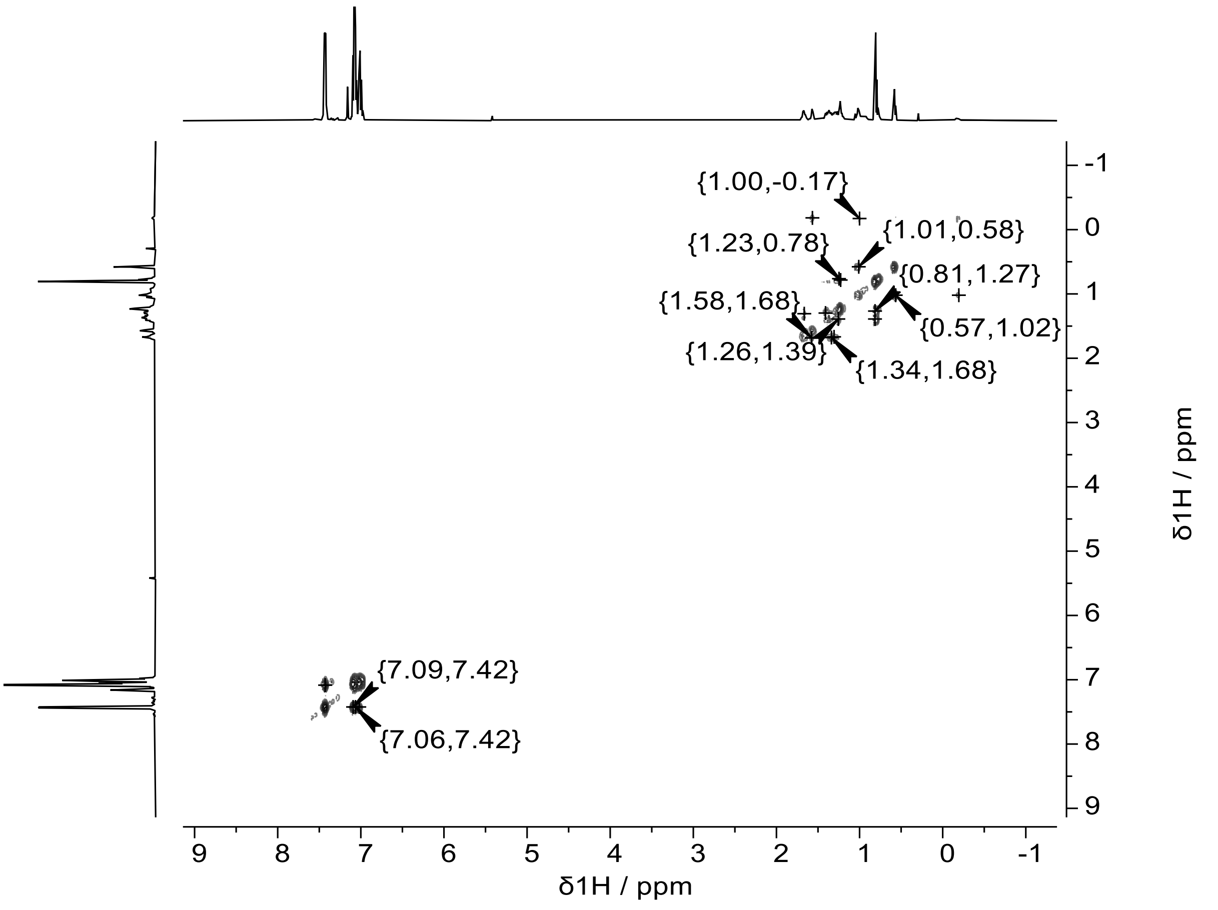

Figure 48: ^{1}H,^{1}H COSY NMR spectrum (500.04 MHz, 500.04 MHz, C_6D_6, 298 K) of complex **10a**.

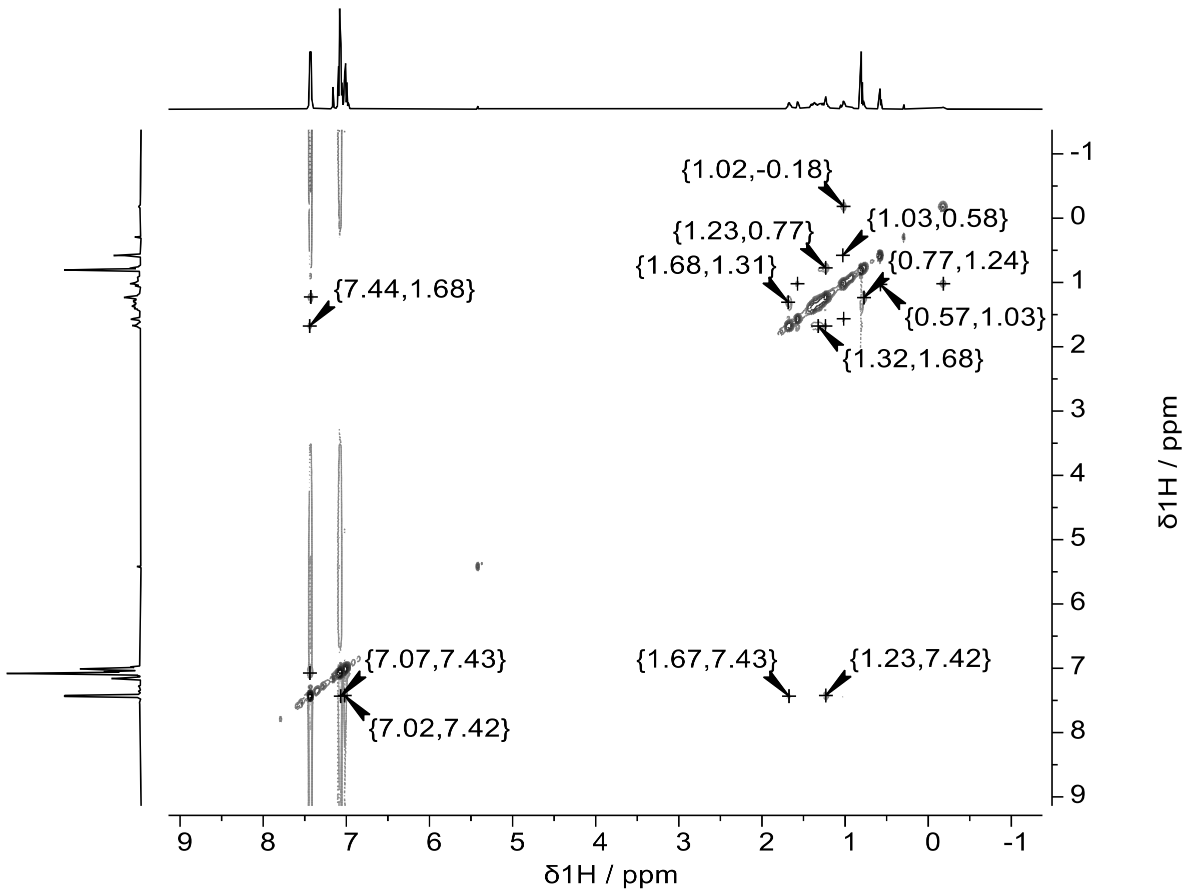

Figure 49: ^{1}H,^{1}H NOESY NMR spectrum (500.04 MHz, 500.04 MHz, C_6D_6, 298 K) of complex **10a**.

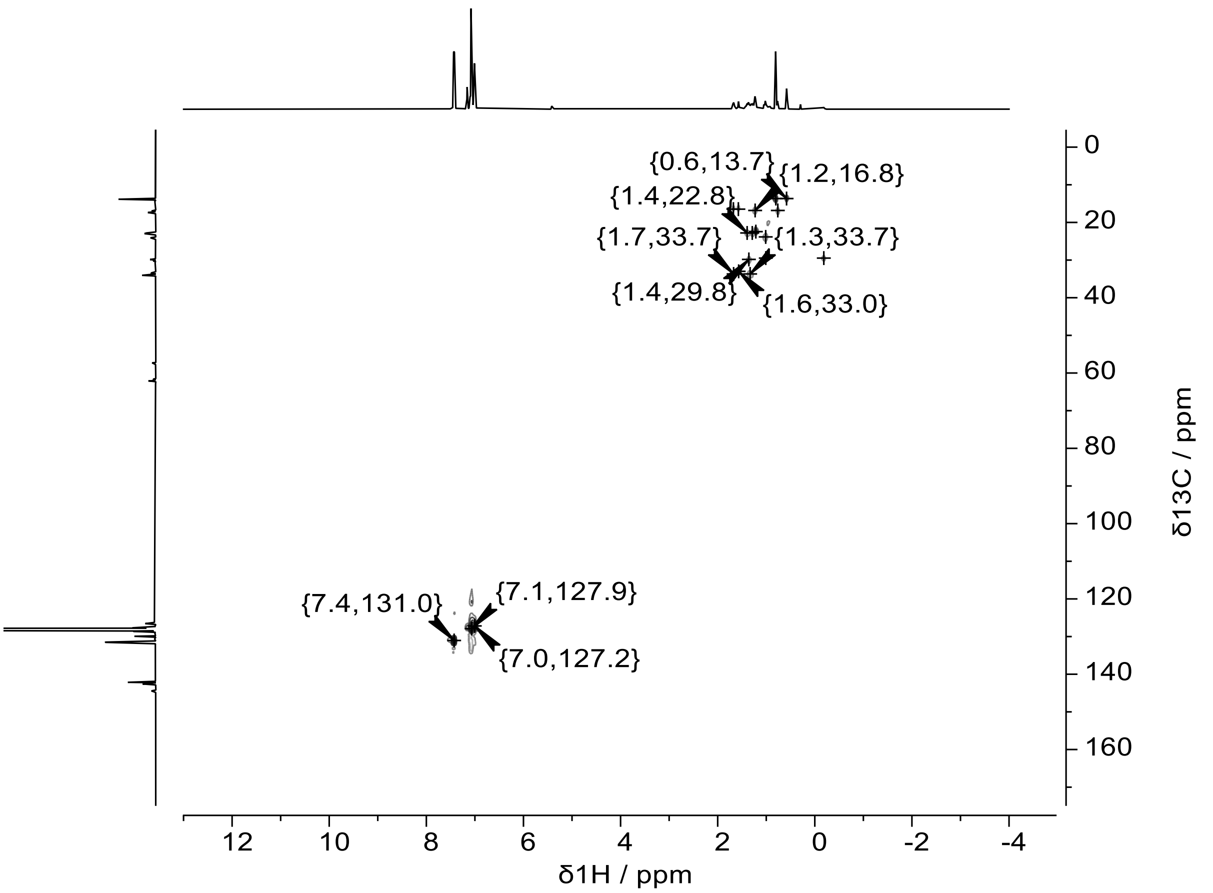

Figure 50: $^{1}H,^{13}C$ HSQC NMR spectrum (500.04 MHz, 125.75 MHz, C_6D_6, 298 K) of complex **10a**.

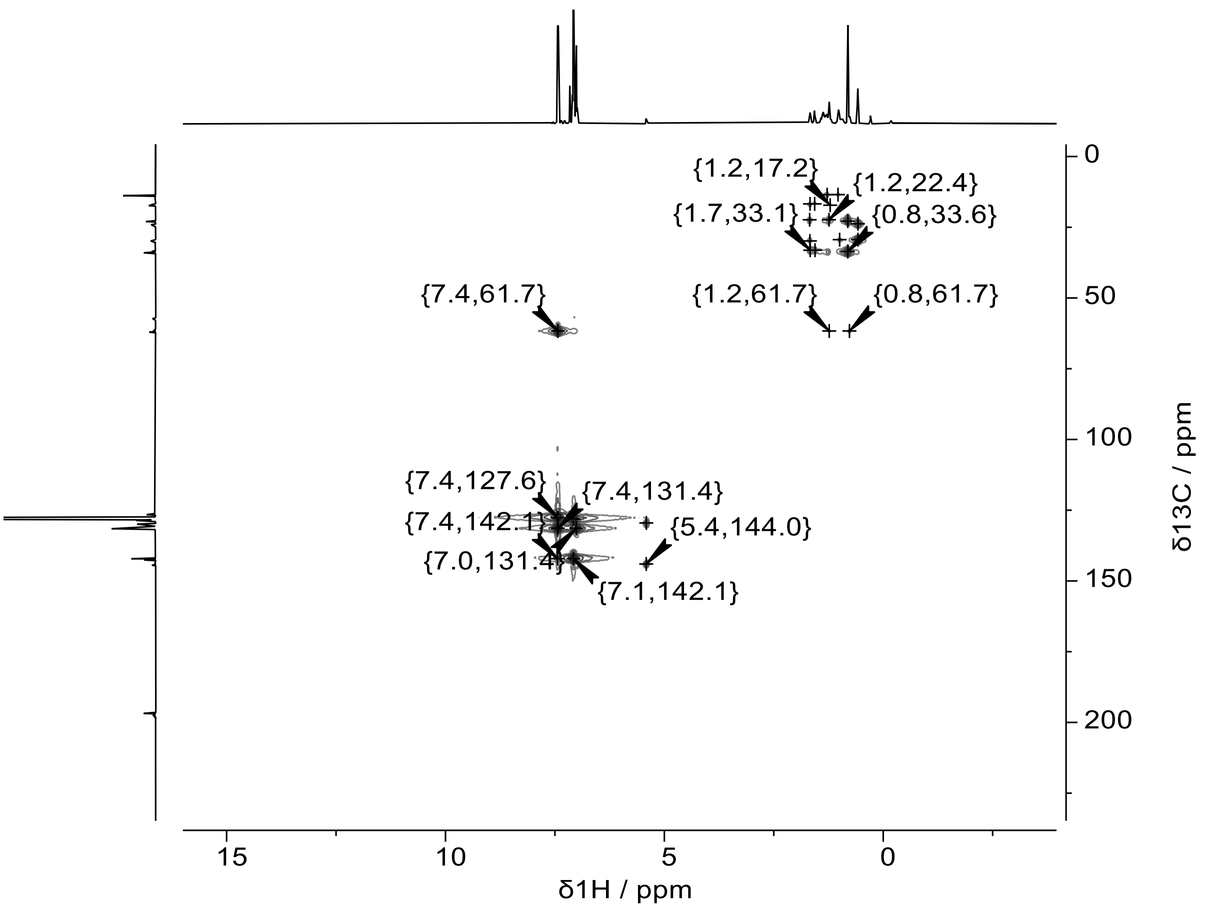

Figure 51: $^{1}H,^{13}C$ HMBC NMR spectrum (500.04 MHz, 125.75 MHz, C_6D_6, 298 K) of complex **10a**.

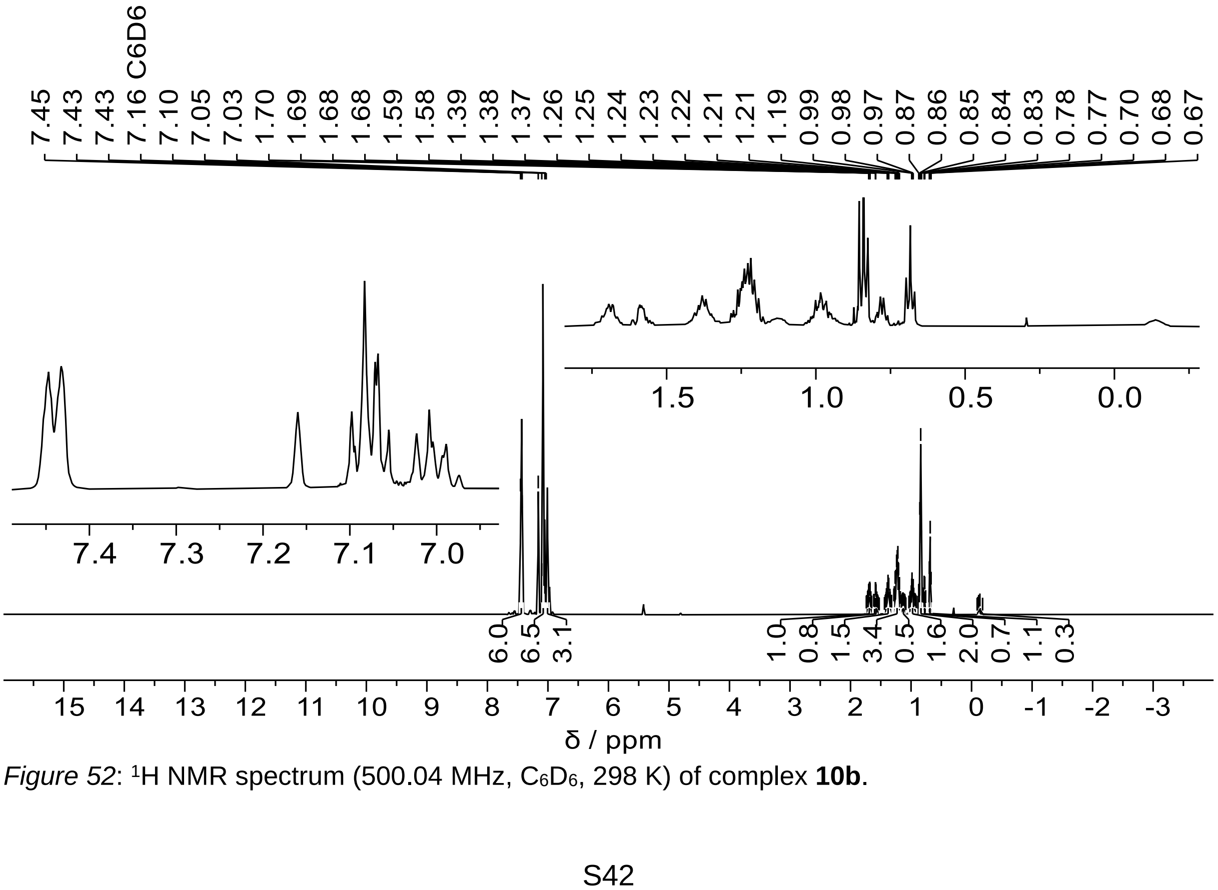

Figure 52: ^{1}H NMR spectrum (500.04 MHz, C_6D_6, 298 K) of complex **10b**.

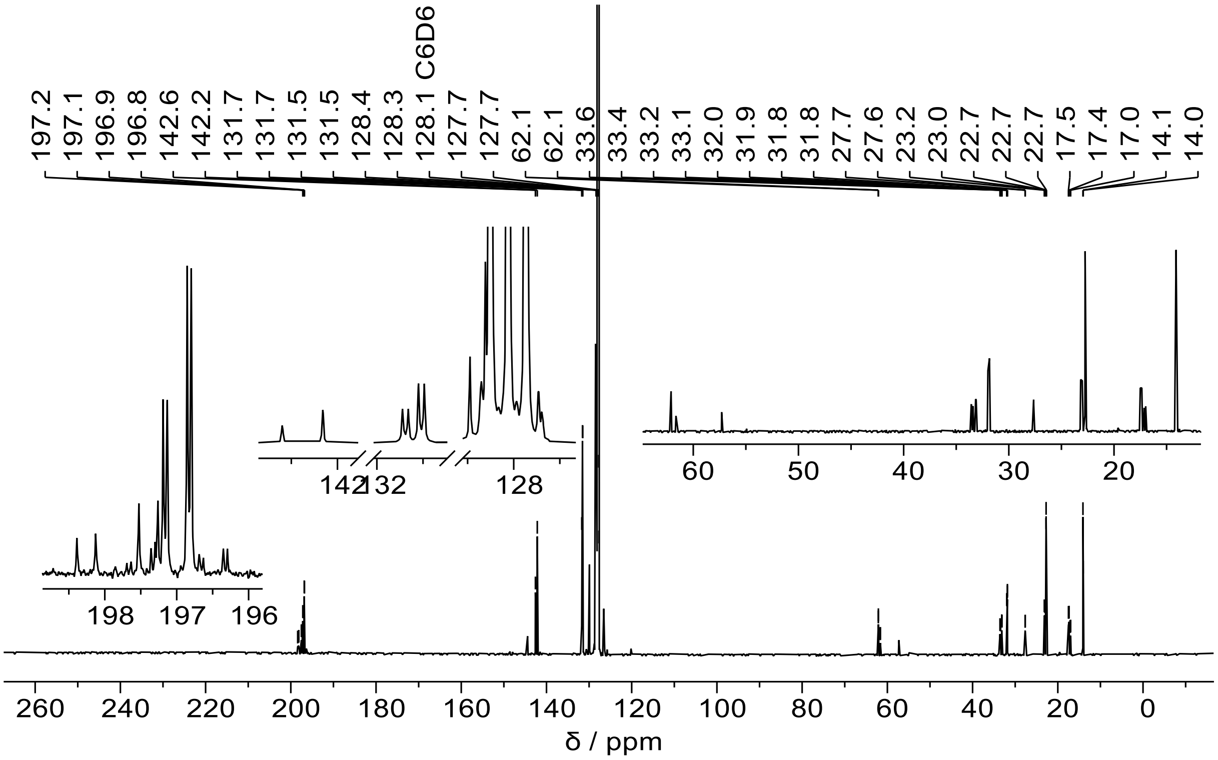

Figure 53: $^{13}C\{^{1}H\}$ NMR spectrum (125.75 MHz, C_6D_6, 298 K) of complex **10b**.

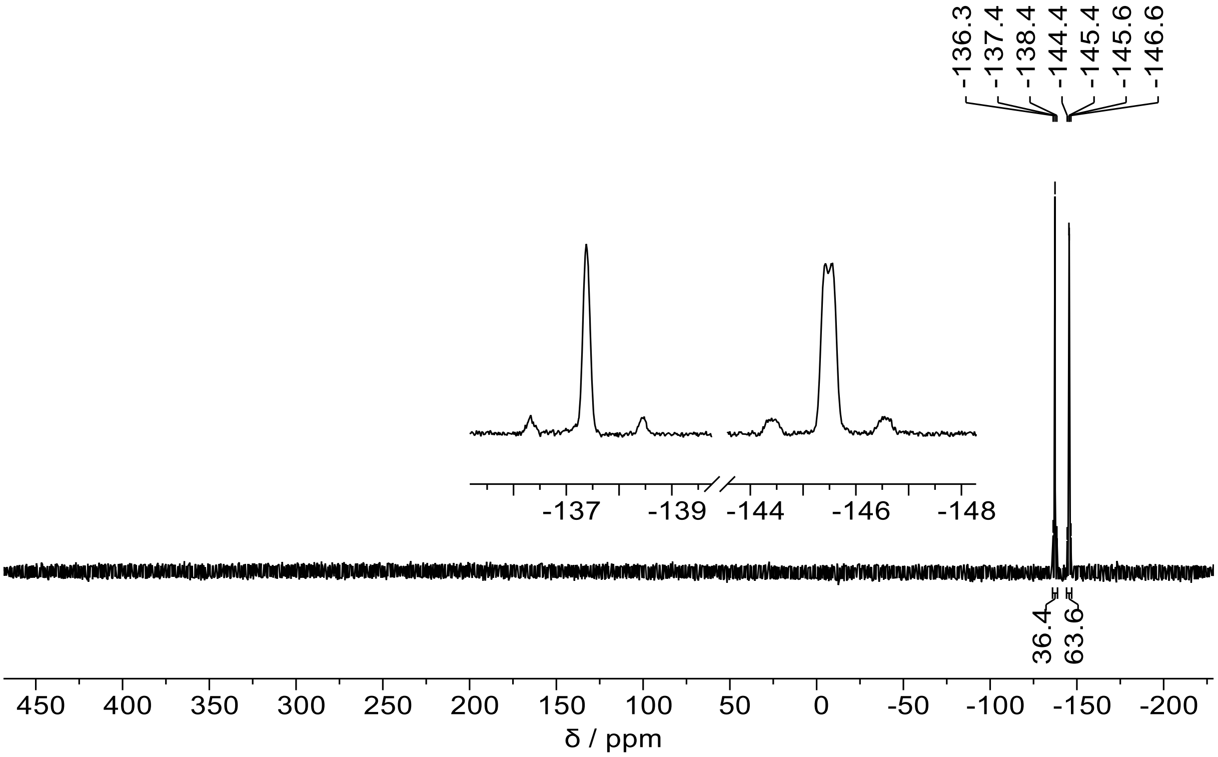

Figure 54: ^{31}P NMR spectrum (121.51 MHz, C_6D_6, 298 K) of complex **10b**.

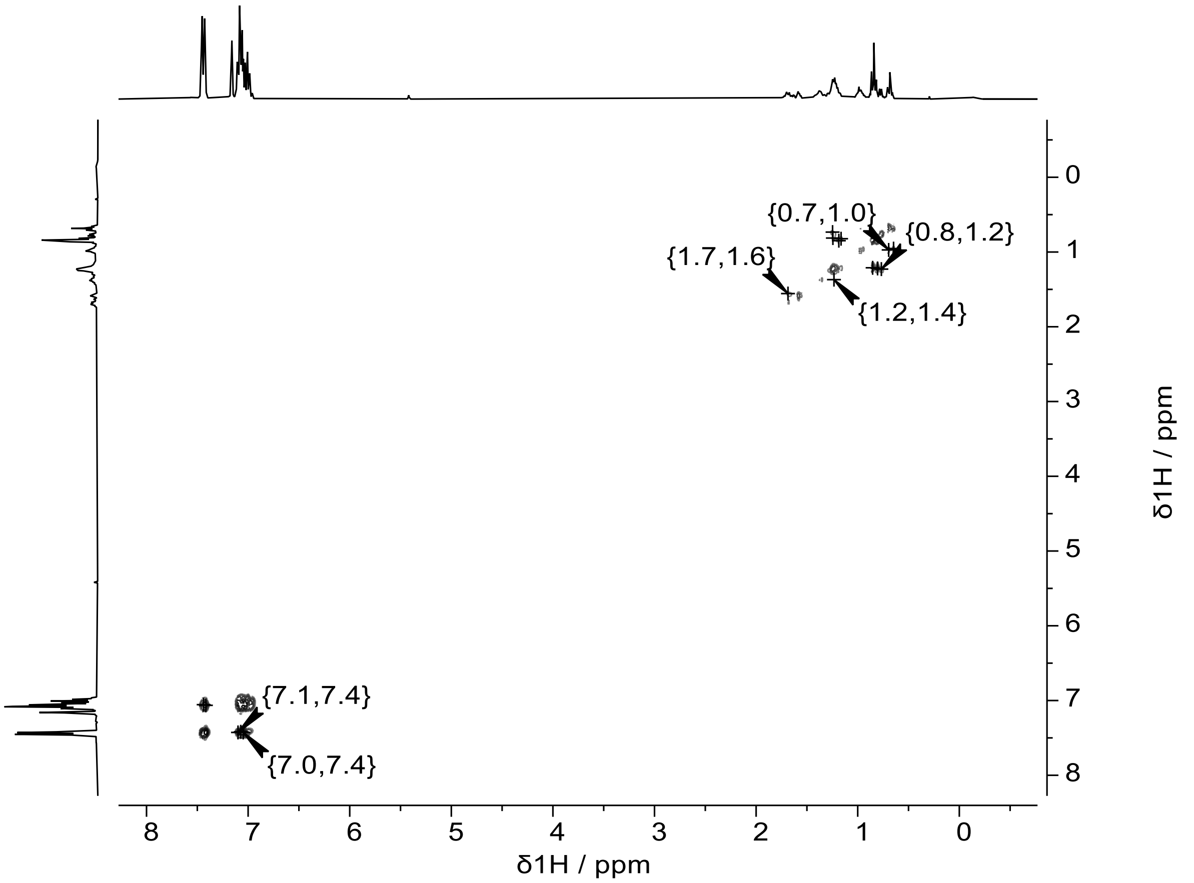

Figure 55: ^{1}H,^{1}H COSY NMR spectrum (300.13 MHz, 300.13 MHz, C_6D_6, 298 K) of complex **10b**.

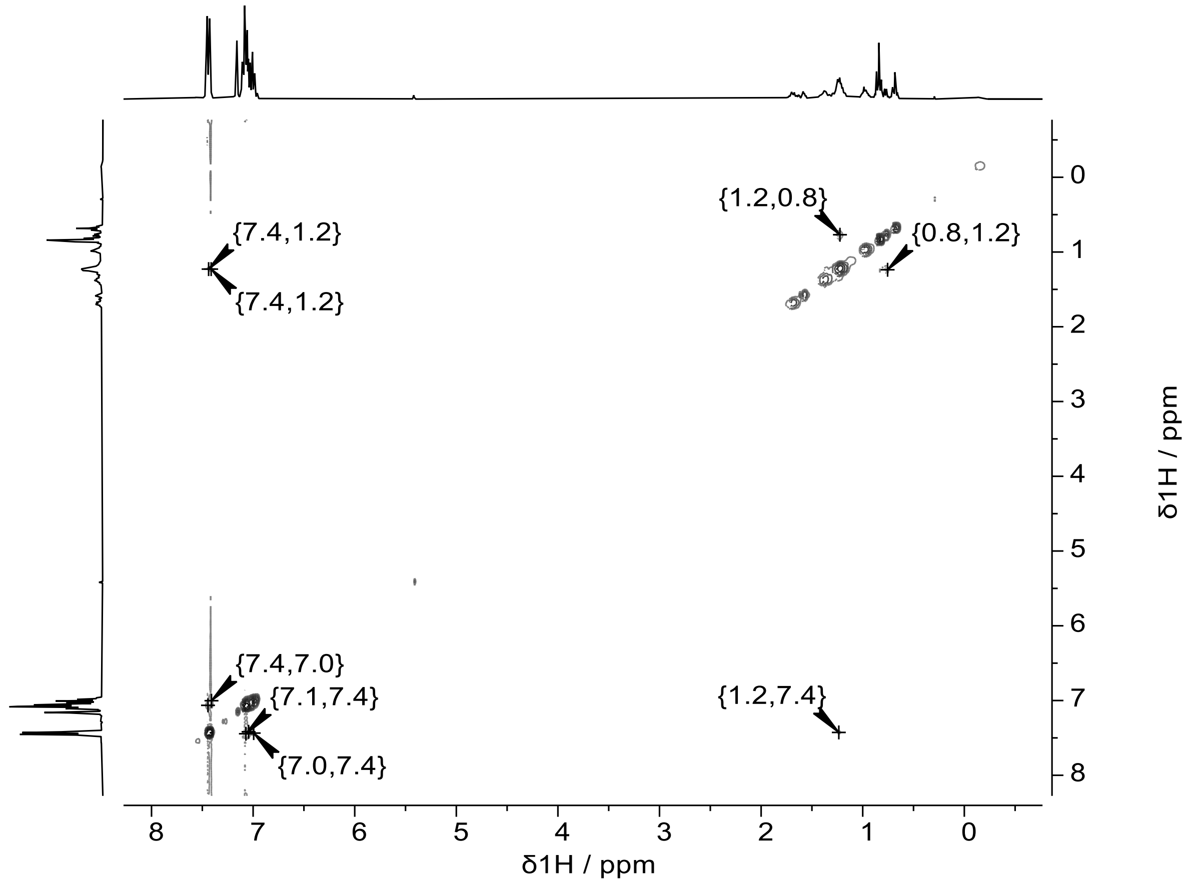

Figure 56: ^{1}H,^{1}H NOESY NMR spectrum (300.13 MHz, 300.13 MHz, C_6D_6, 298 K) of complex **10b**.

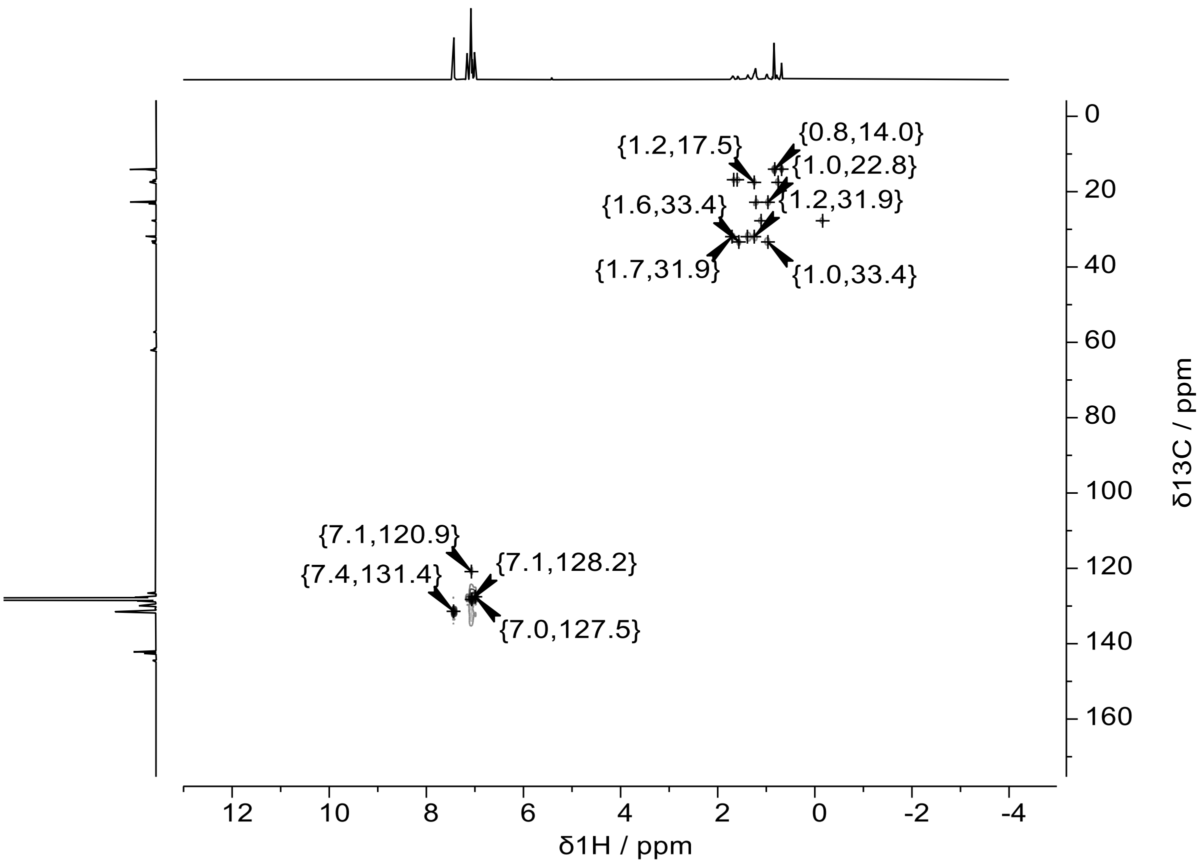

Figure 57: ^{1}H,^{13}C HSQC NMR spectrum (500.04 MHz, 125.75 MHz, C_6D_6, 298 K) of complex **10b**.

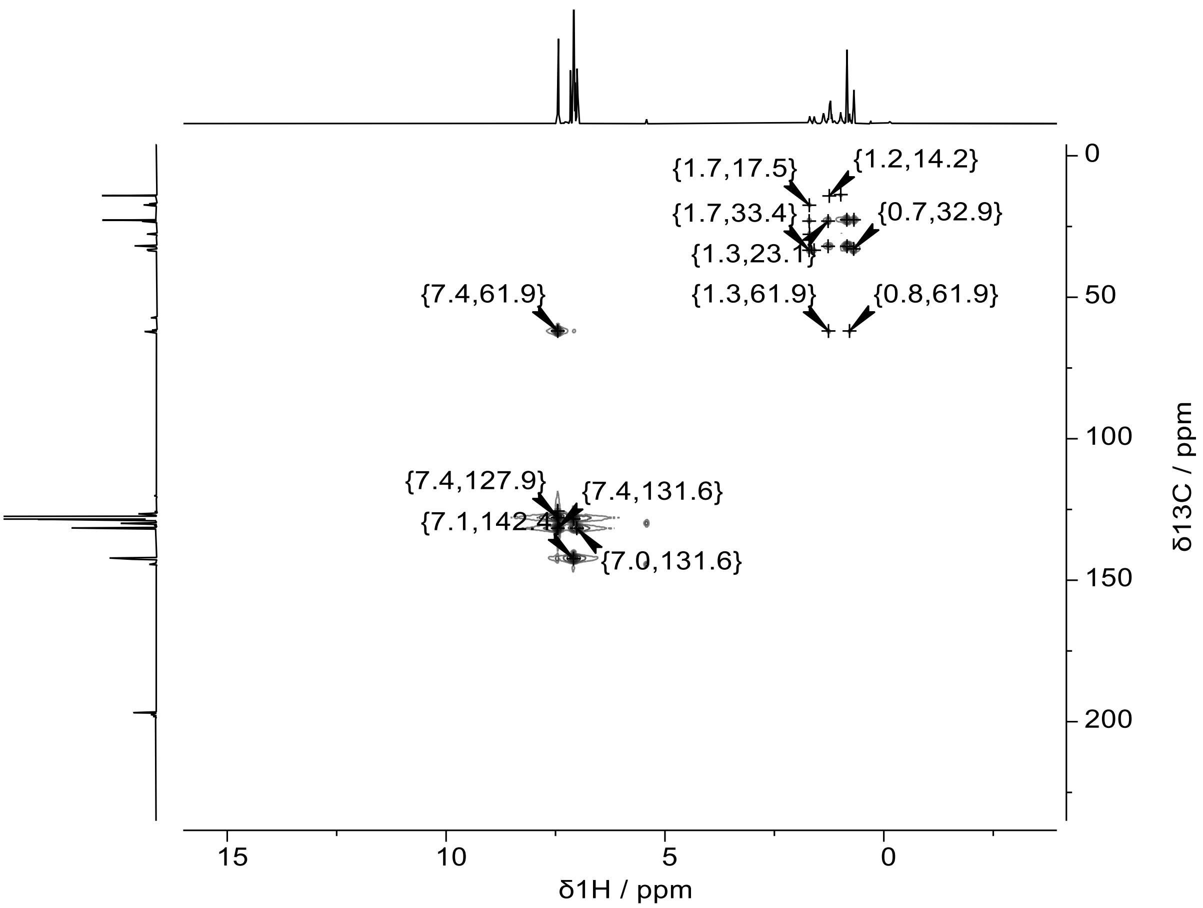

Figure 58: ^{1}H,^{13}C HMBC NMR spectrum (500.04 MHz, 125.75 MHz, C_6D_6, 298 K) of complex **10b**.

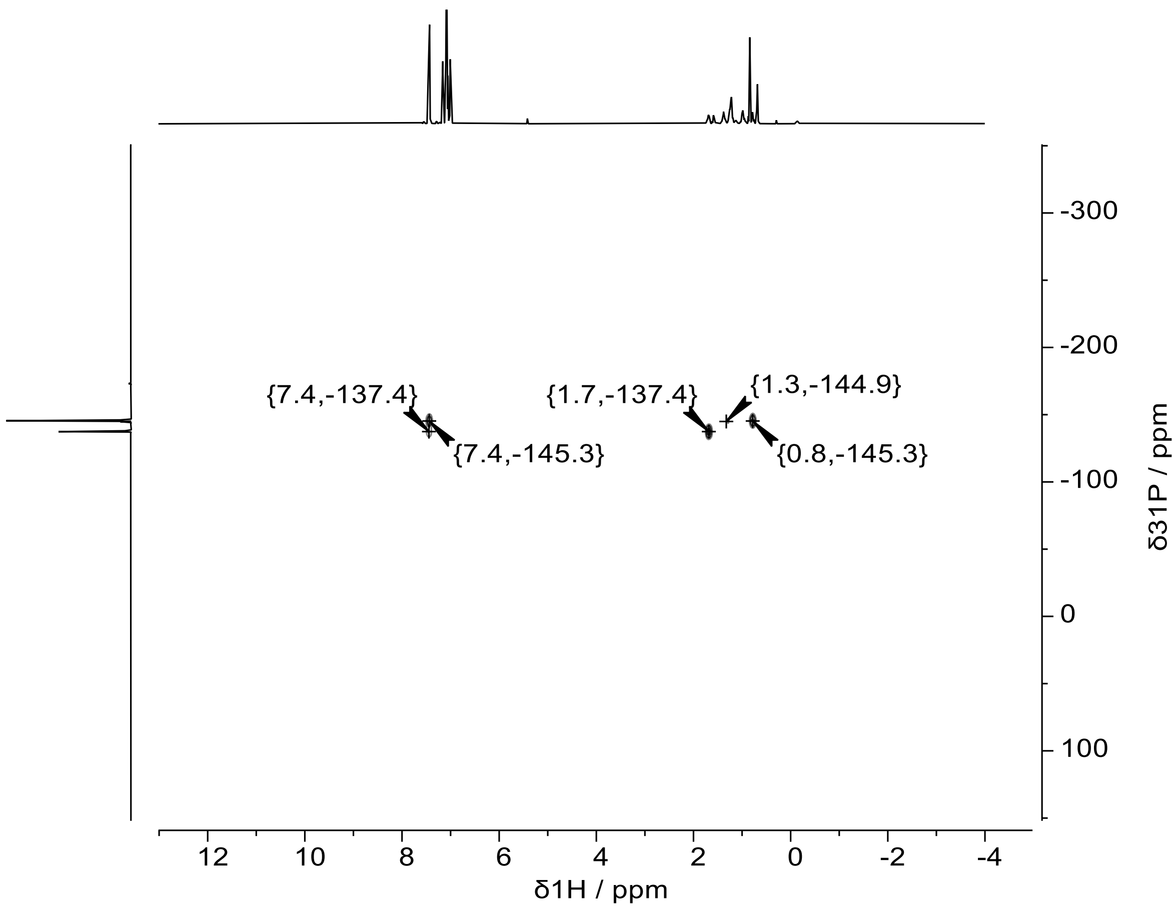

Figure 59: $^{1}H,^{31}P$ HMBC NMR spectrum (500.04 MHz, 202.40 MHz, C_6D_6, 298 K) of complex **10b**.

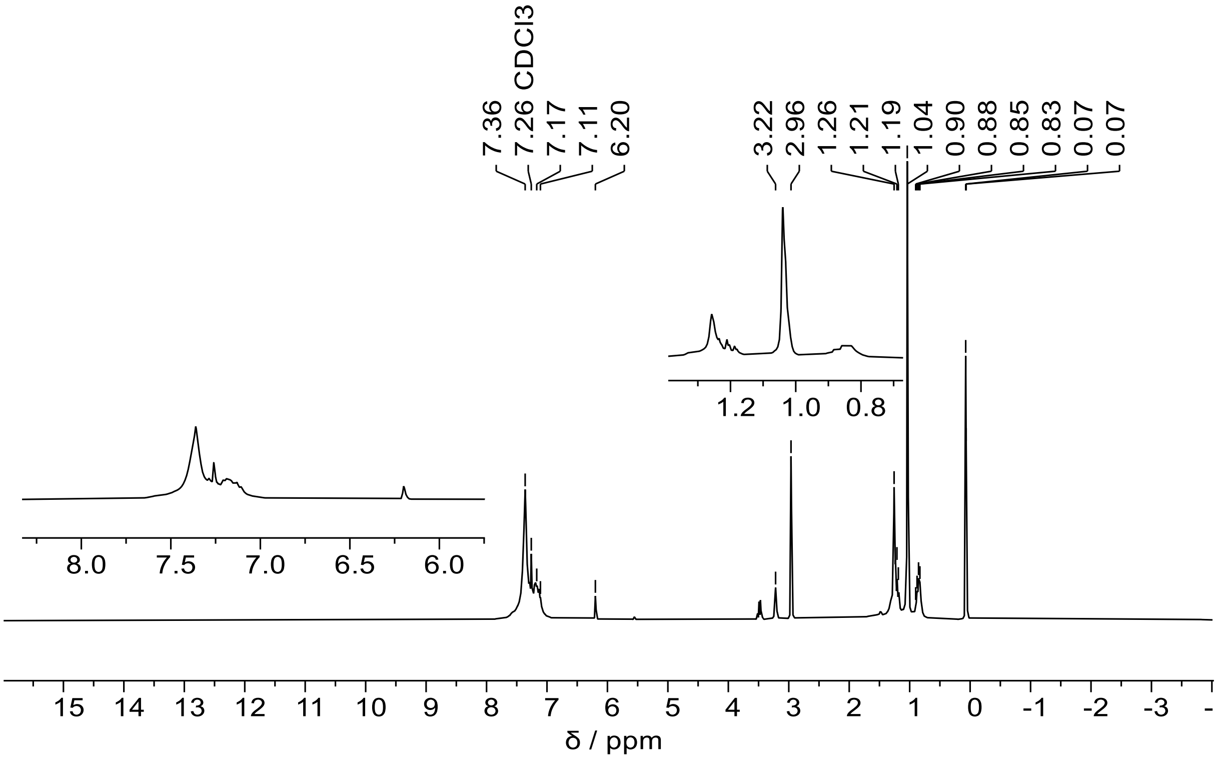

Figure 60: ^{1}H NMR spectrum (300.13 MHz, $CDCl_3$, 298 K) of complex **11a**.

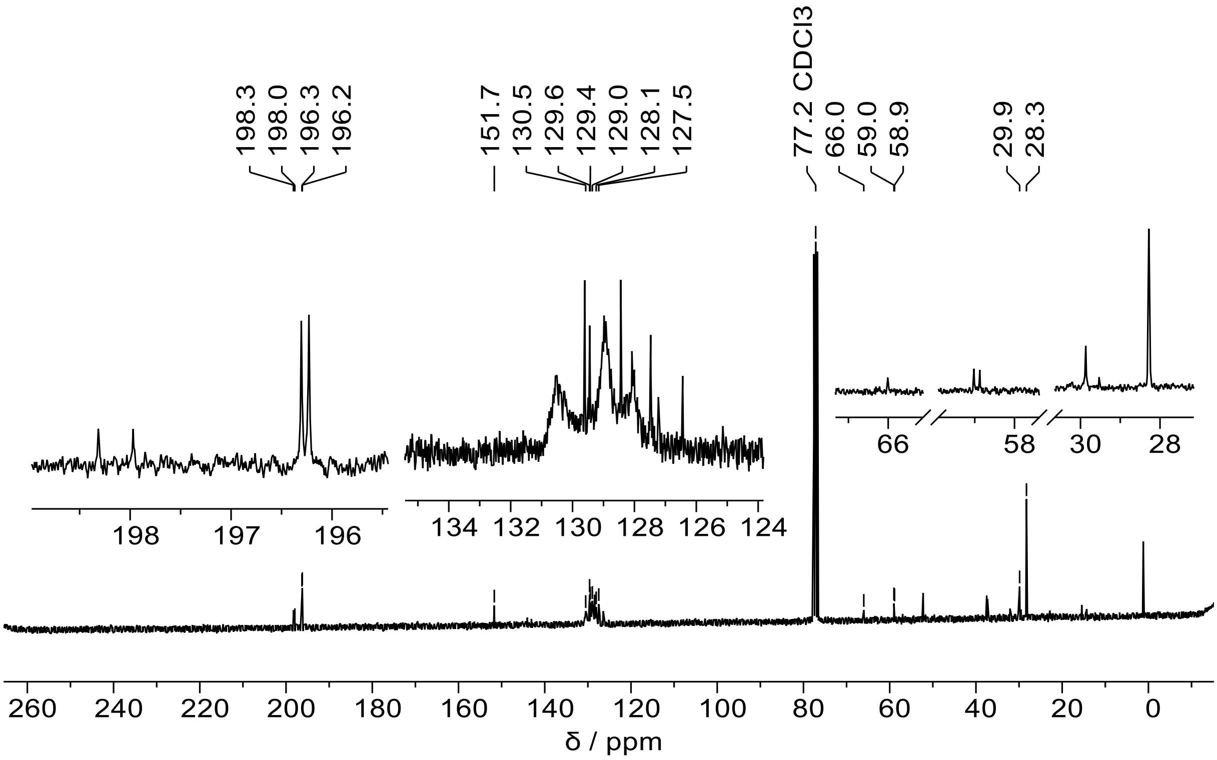

Figure 61: $^{13}C\{^1H\}$ NMR spectrum (75.48 MHz, $CDCl_3$, 298 K) of complex **11a**.

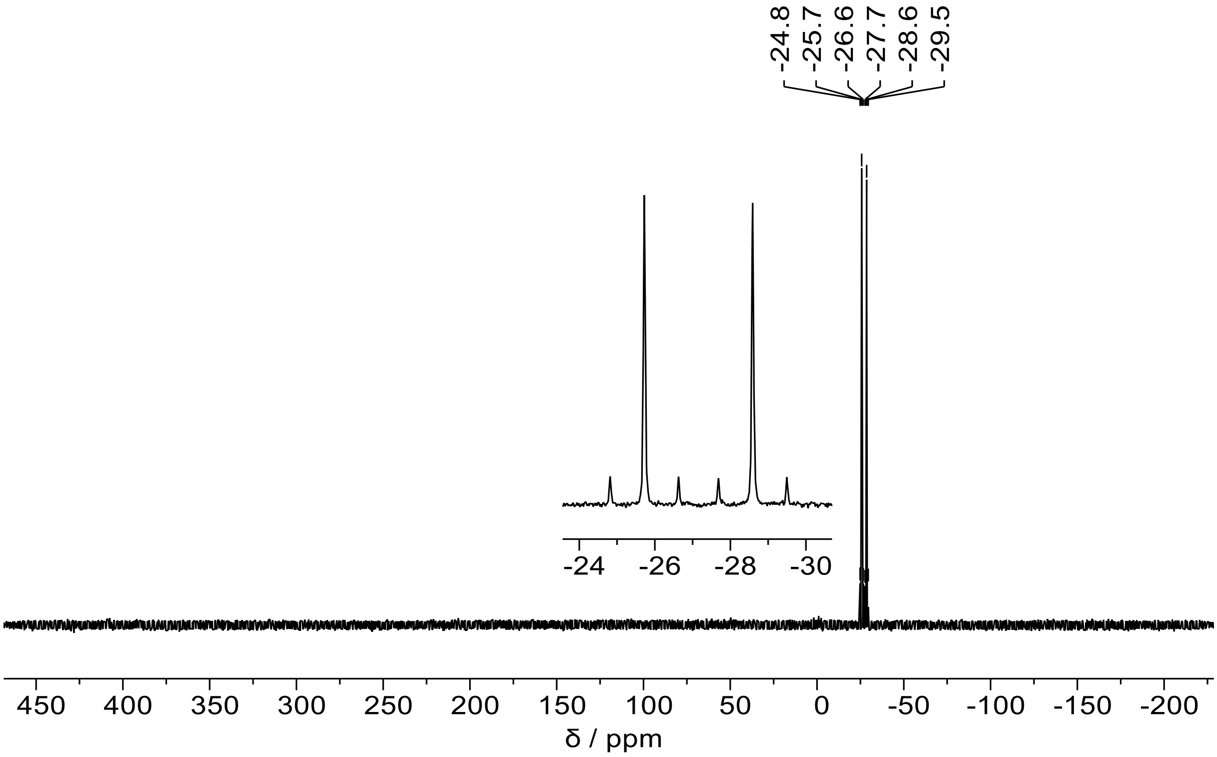

Figure 62: ^{31}P NMR spectrum (121.51 MHz, $CDCl_3$, 298 K) of complex **11a**.

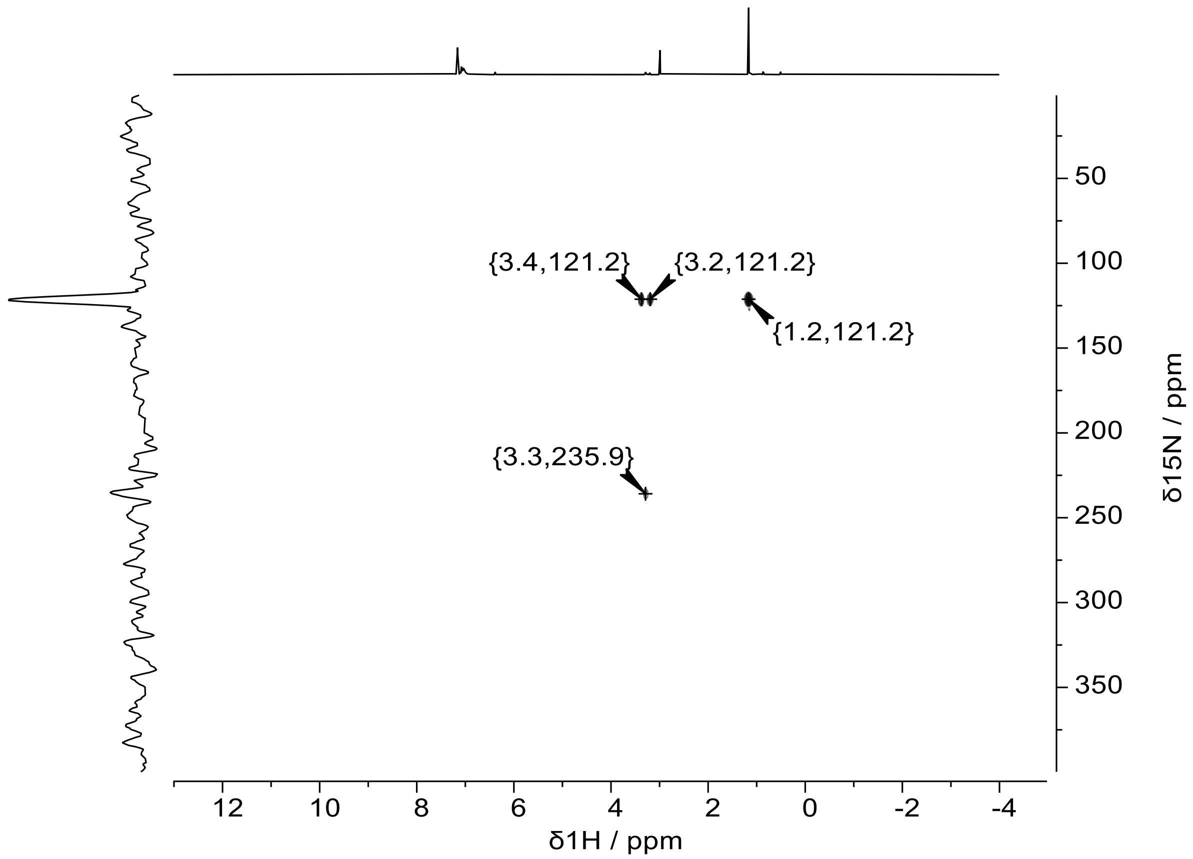

Figure 63: $^{1}H,^{15}N$ HMBC NMR spectrum (500.04 MHz, 50.68 MHz, C_6D_6, 298 K) of complex **11a**.

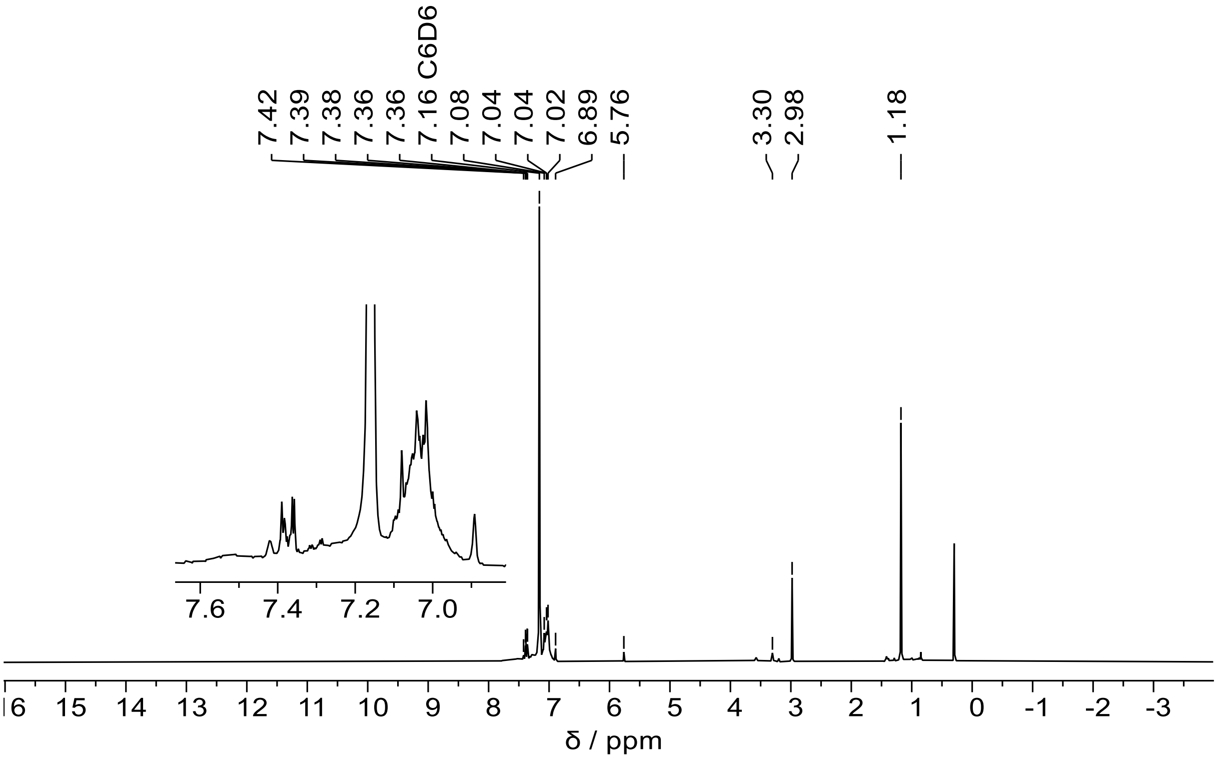

Figure 64: ^{1}H NMR spectrum (300.13 MHz, C_6D_6, 299 K) of complex **11^{Cr}a**.

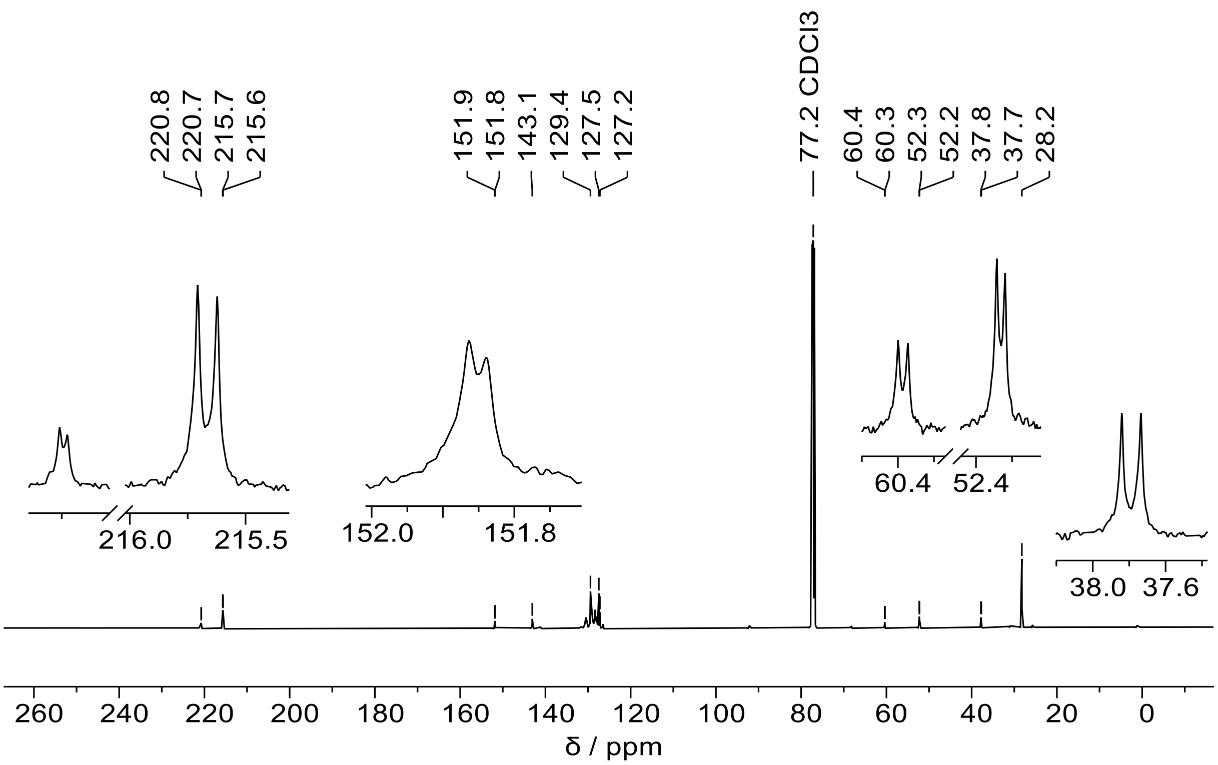

Figure 65: $^{13}C\{^{1}H\}$ NMR spectrum (125.78 MHz, $CDCl_3$, 297 K) of complex **11^{Cr}a**.

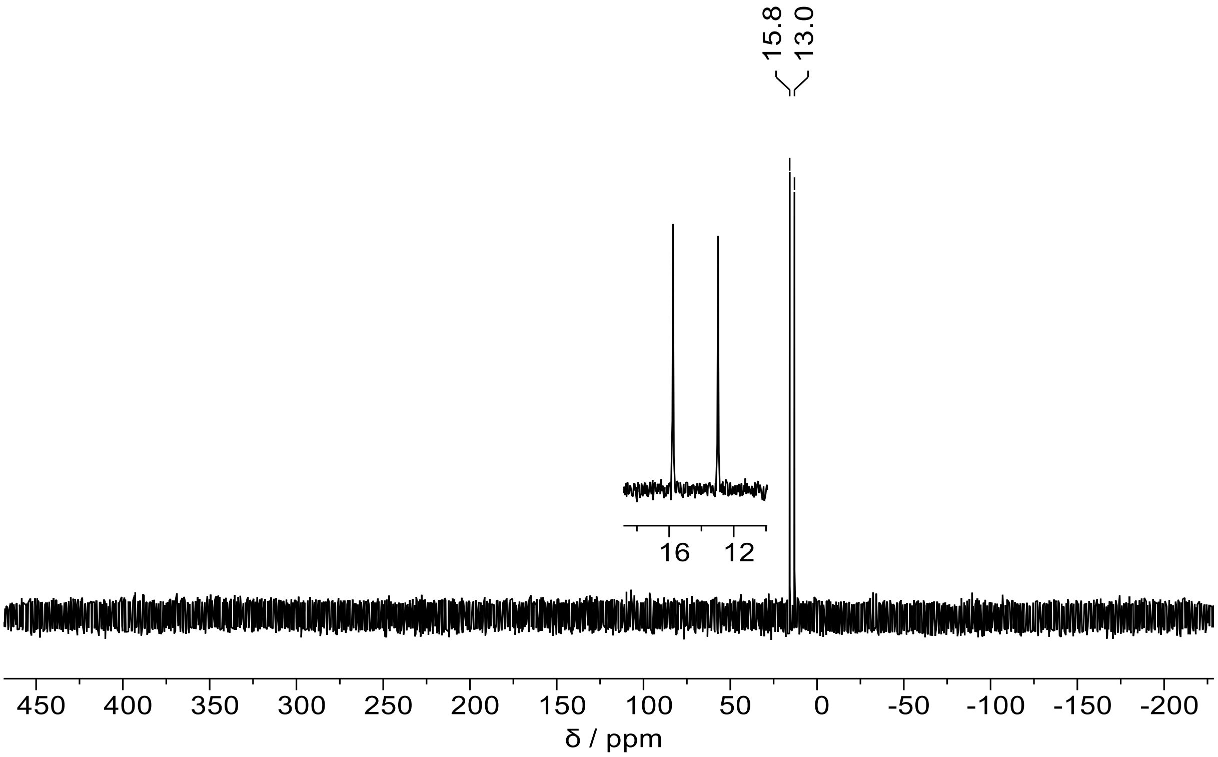

Figure 66: ^{31}P NMR spectrum (121.51 MHz, C_6D_6, 299 K) of complex **11^{Cr}a**.

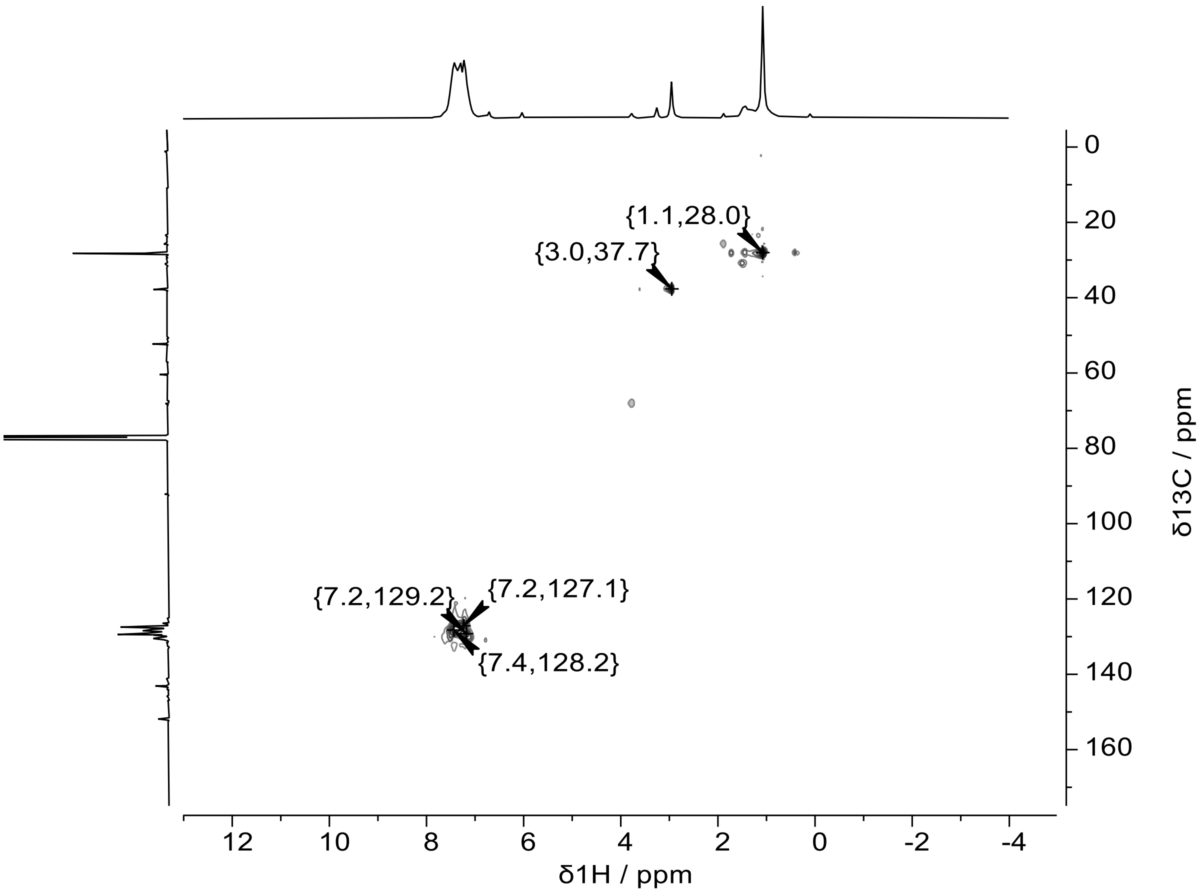

Figure 67: $^{1}H,^{13}C$ HSQC NMR spectrum (500.14 MHz, 125.77 MHz, $CDCl_3$, 297 K) of complex **11^{Cr}a**.

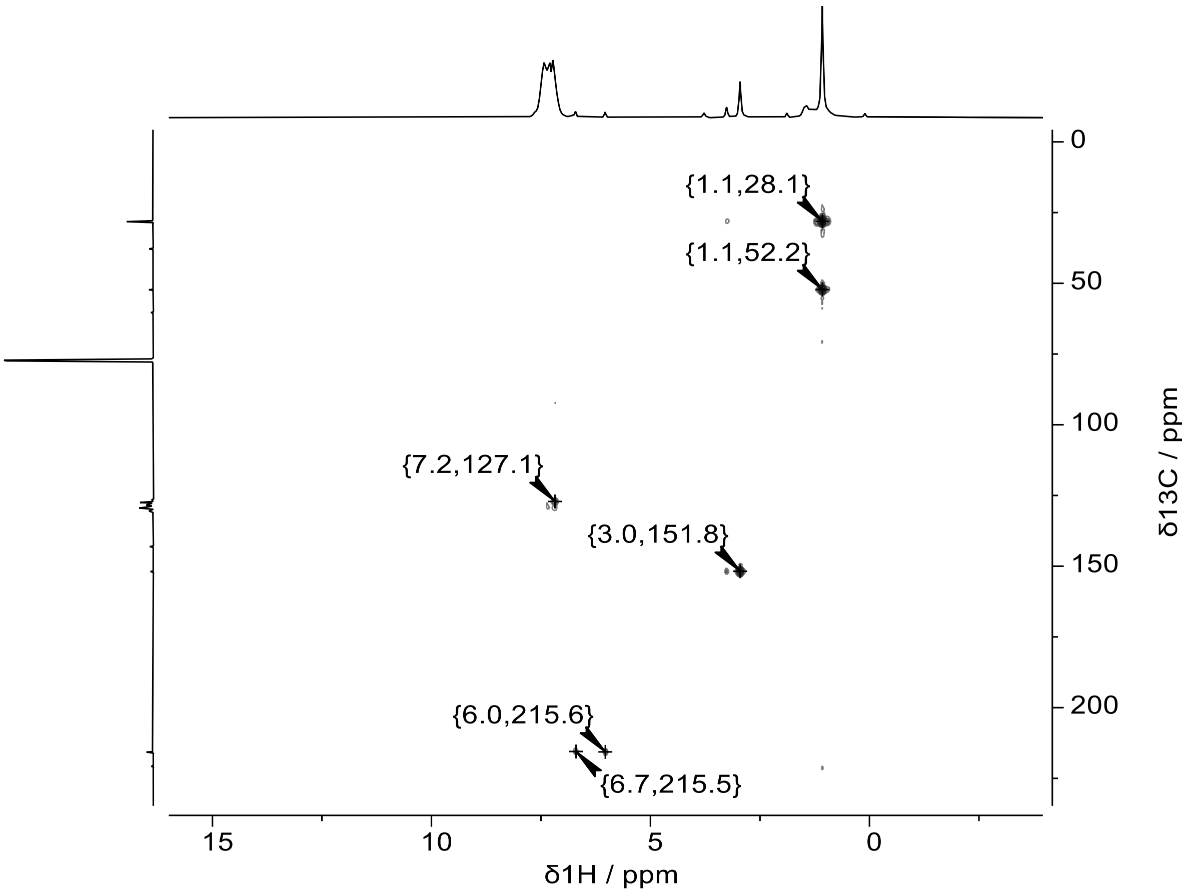

Figure 68: $^{1}H,^{13}C$ HMBC NMR spectrum (500.14 MHz, 125.77 MHz, $CDCl_3$, 297 K) of complex **11^{Cr}a**.

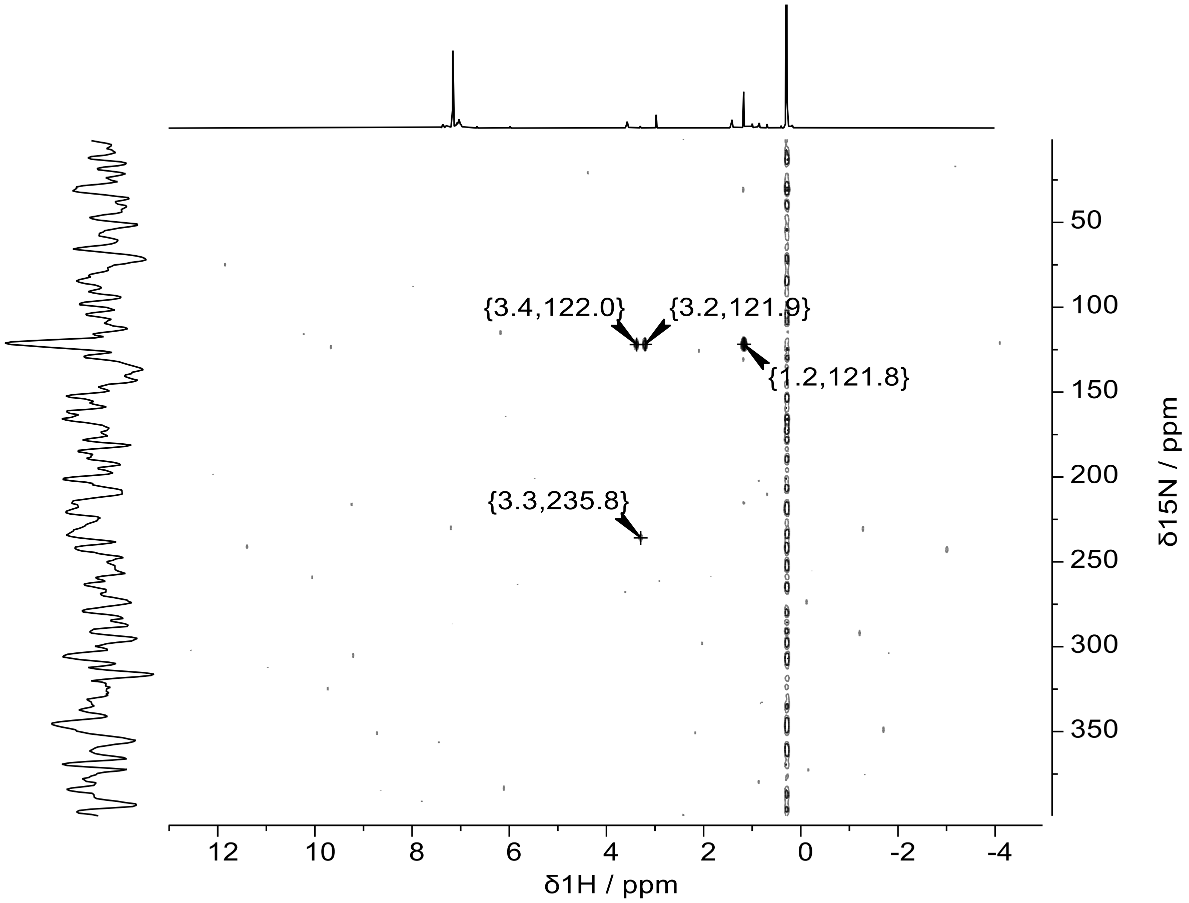

Figure 69: $^{1}H,^{15}N$ HMBC NMR spectrum (500.14 MHz, 50.69 MHz, C_6D_6, 298 K) of complex **11^{Cr}a**.

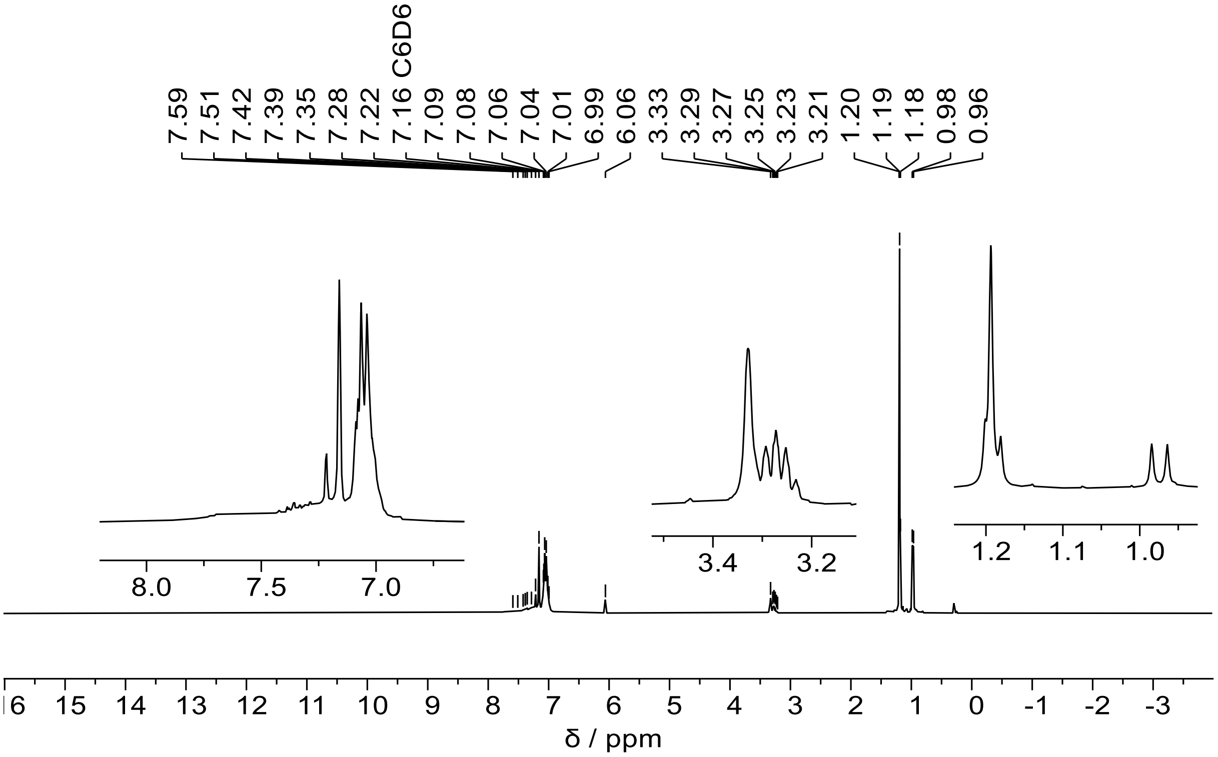

Figure 70: ^{1}H NMR spectrum (300.13 MHz, C_6D_6, 298 K) of complex **11b**.

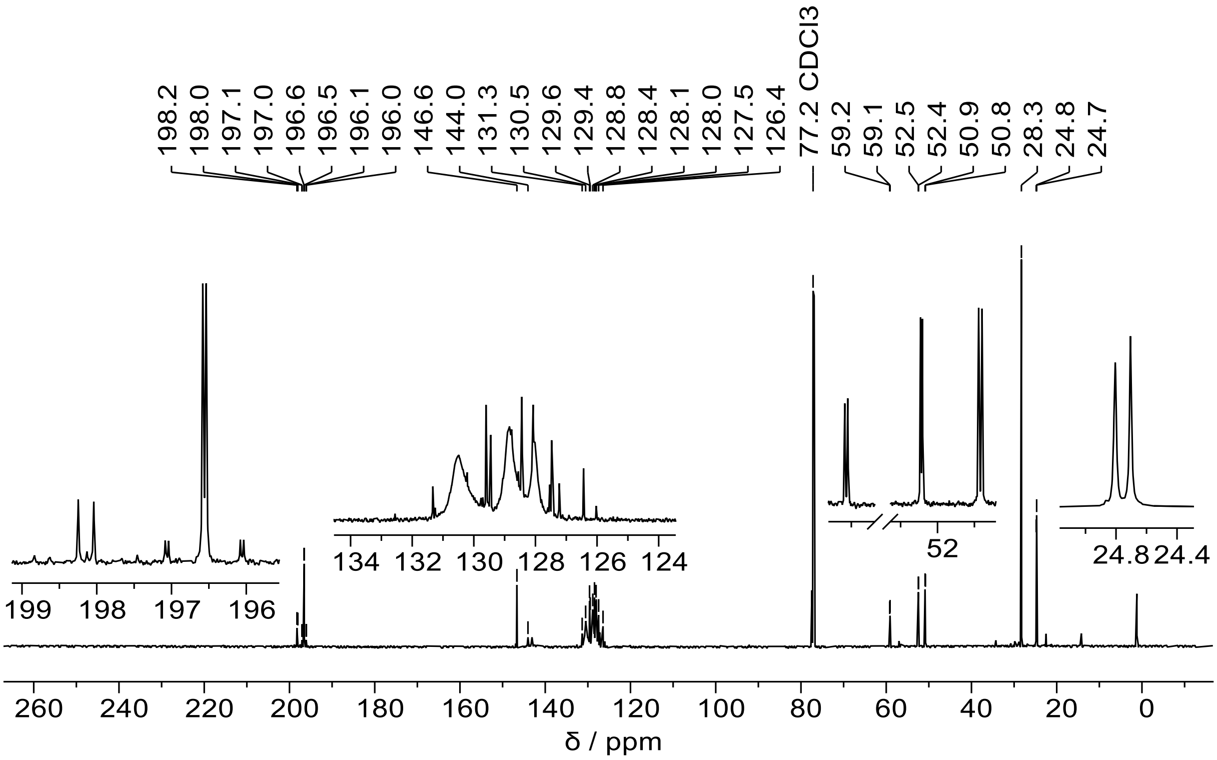

Figure 71: $^{13}C\{^{1}H\}$ NMR spectrum (125.78 MHz, $CDCl_3$, 298 K) of complex **11b**.

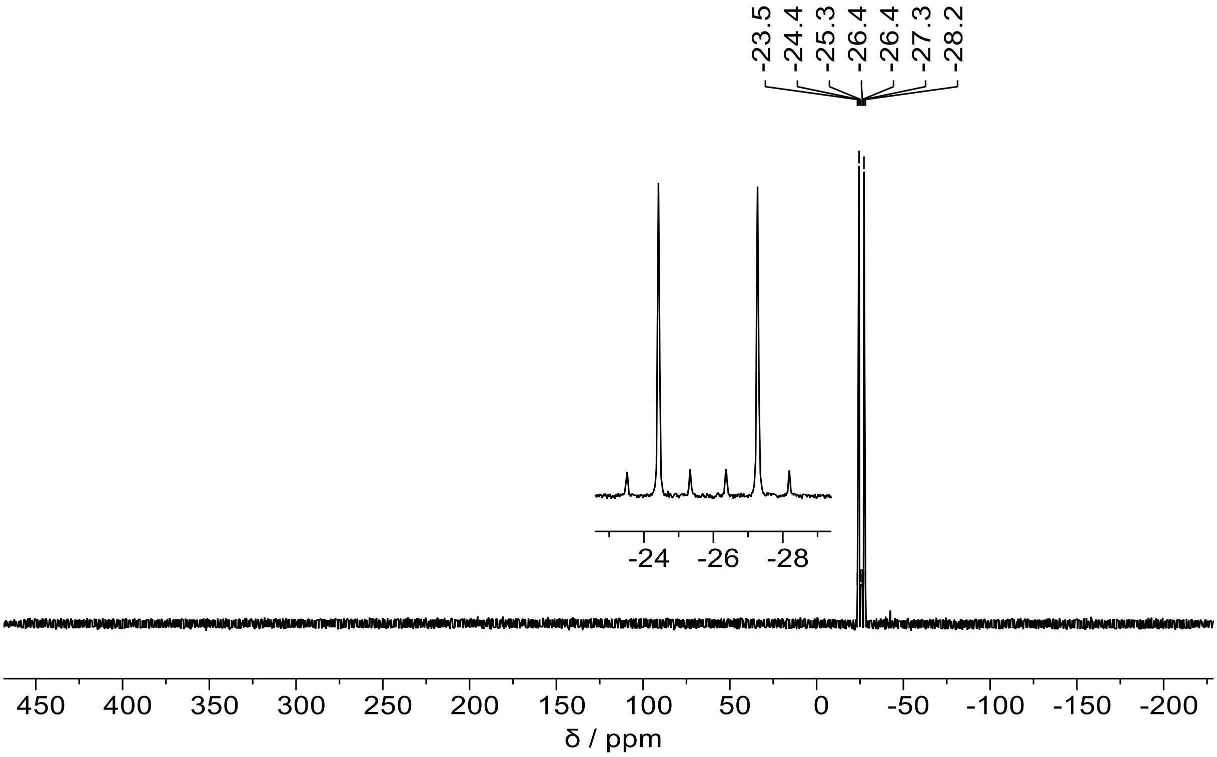

Figure 72: ^{31}P NMR spectrum (121.51 MHz, C_6D_6, 298 K) of complex **11b**.

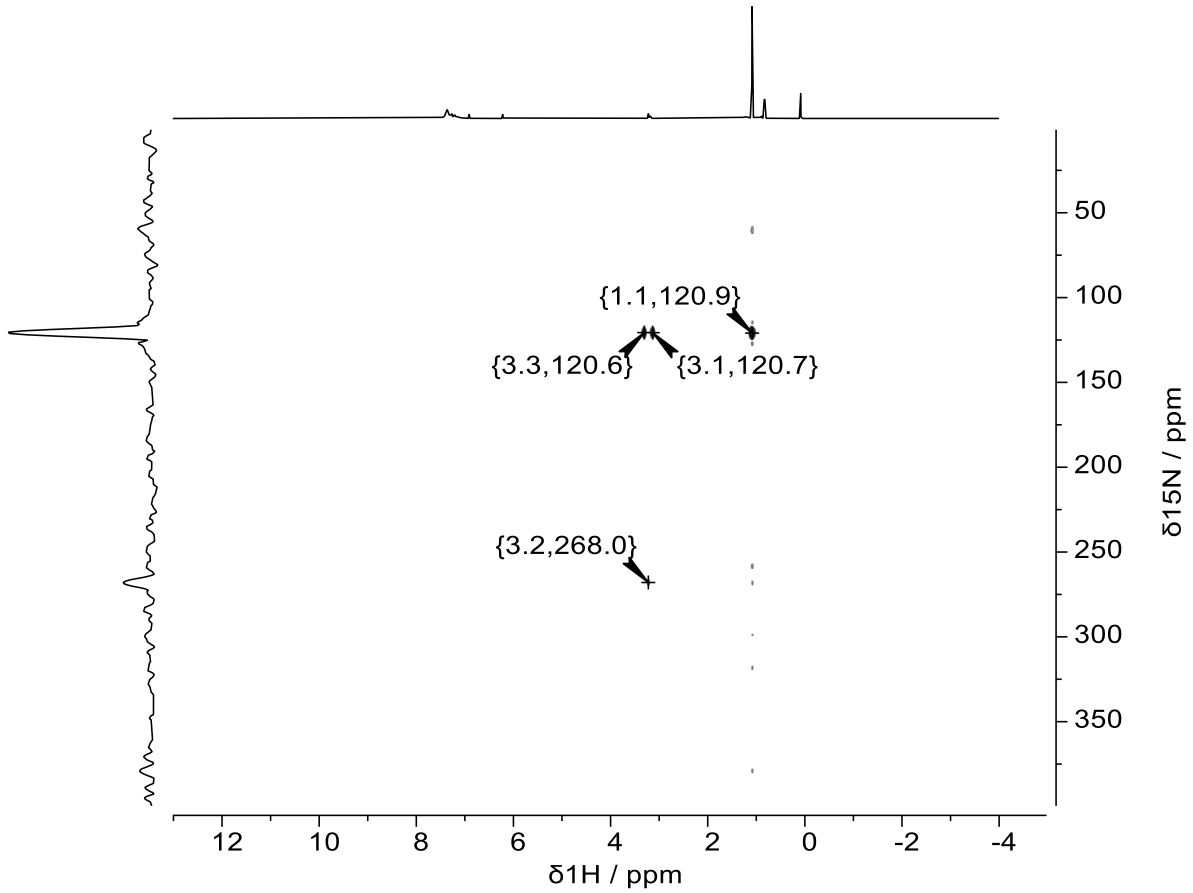

Figure 73: $^{1}H,^{15}N$ HMBC NMR spectrum (500.14 MHz, 50.69 MHz, $CDCl_3$, 298 K) of complex **11b**.

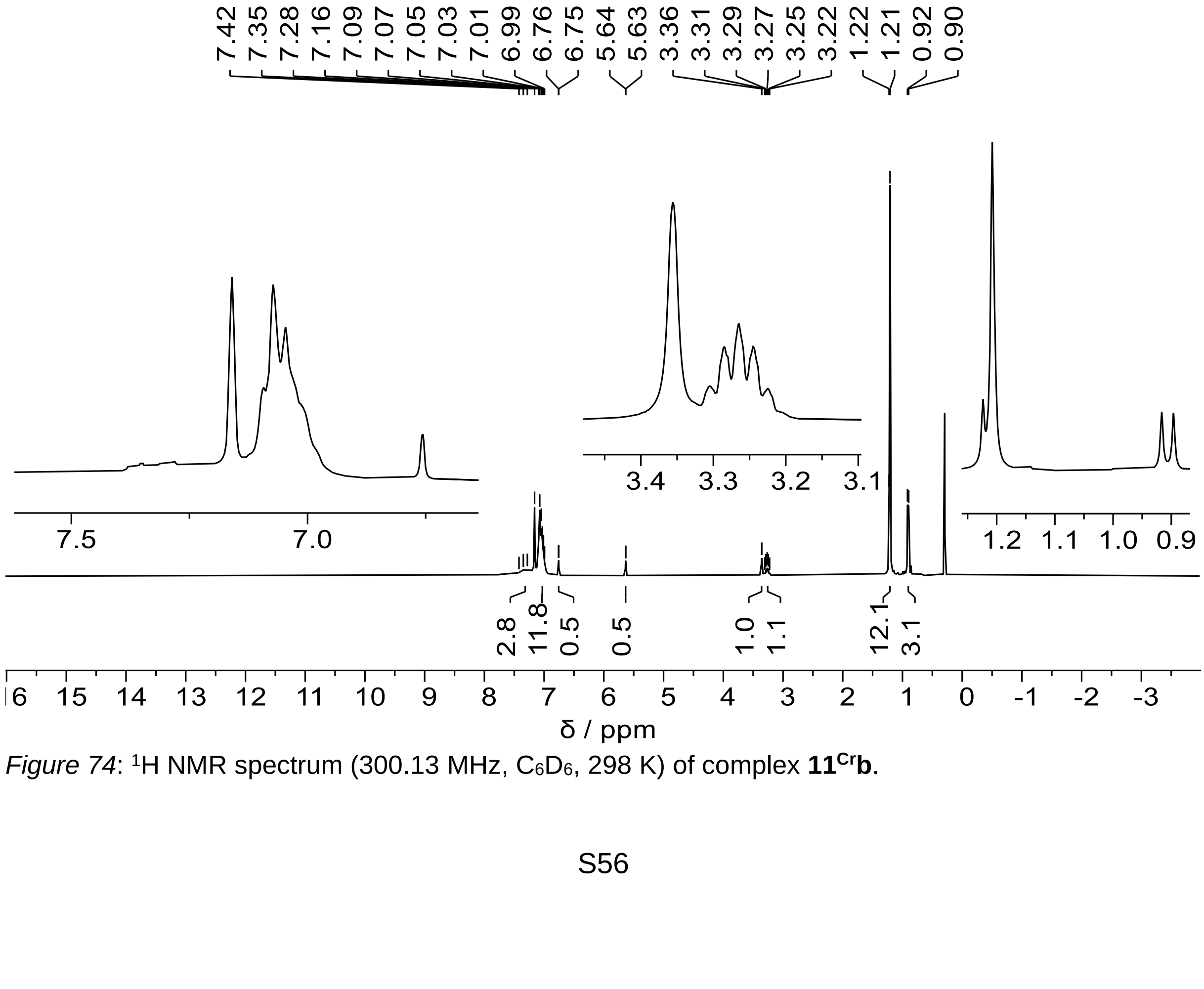

Figure 74: ^{1}H NMR spectrum (300.13 MHz, C_6D_6, 298 K) of complex **$11^{Cr}b$**.

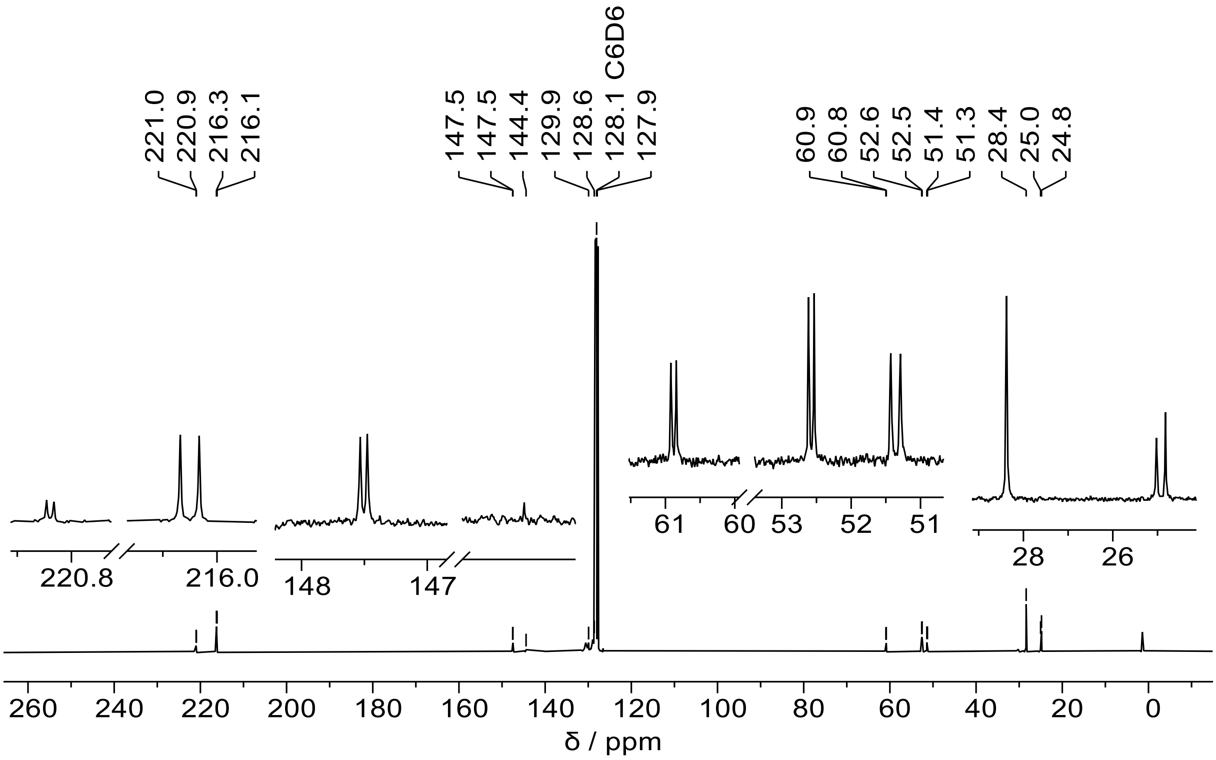

Figure 75: $^{13}C\{^1H\}$ NMR spectrum (75.48 MHz, C_6D_6, 298 K) of complex **11^{Cr}b**.

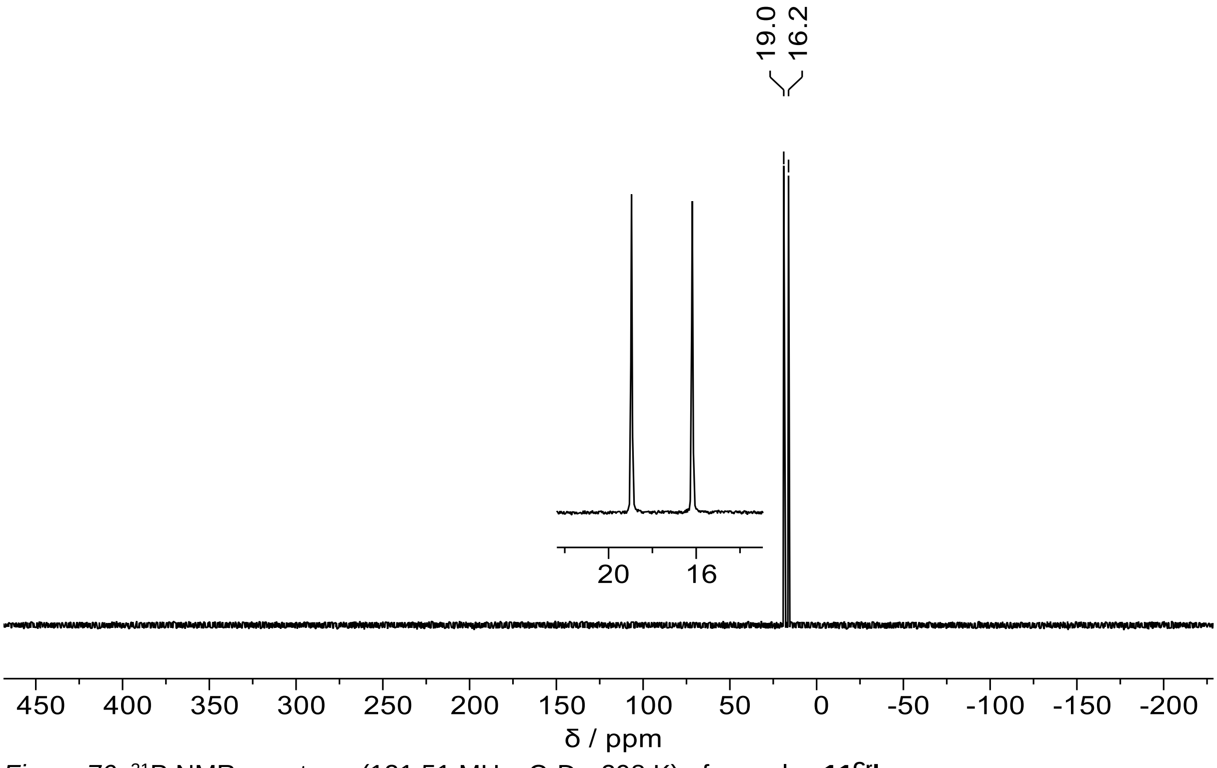

Figure 76: ^{31}P NMR spectrum (121.51 MHz, C_6D_6, 298 K) of complex **11^{Cr}b**.

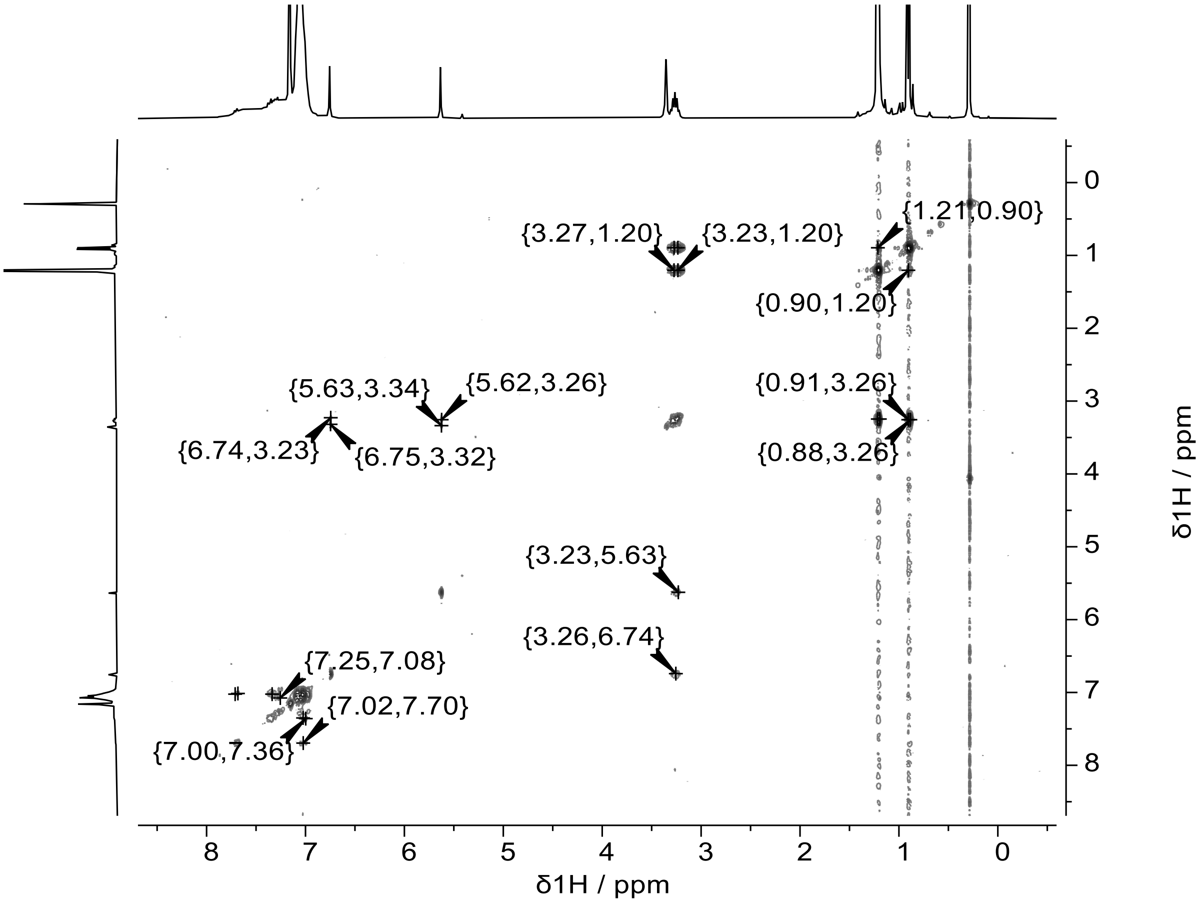

Figure 77: ^{1}H,^{1}H COSY NMR spectrum (300.13 MHz, 300.13 MHz, C_6D_6, 298 K) of complex **11^{Cr}b**.

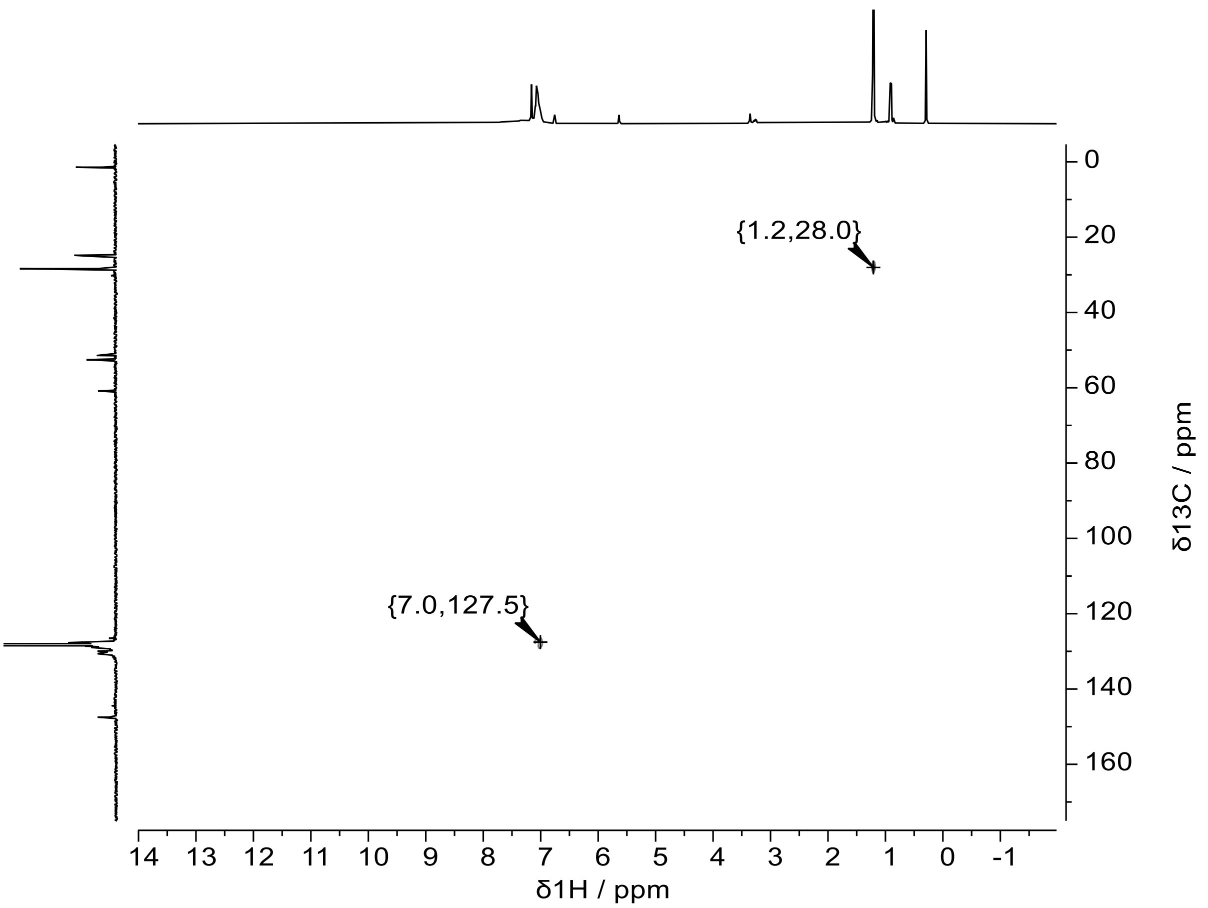

Figure 78: $^{1}H,^{13}C$ HSQC NMR spectrum (300.13 MHz, 75.47 MHz, C_6D_6, 298 K) of complex **11^{Cr}b**.

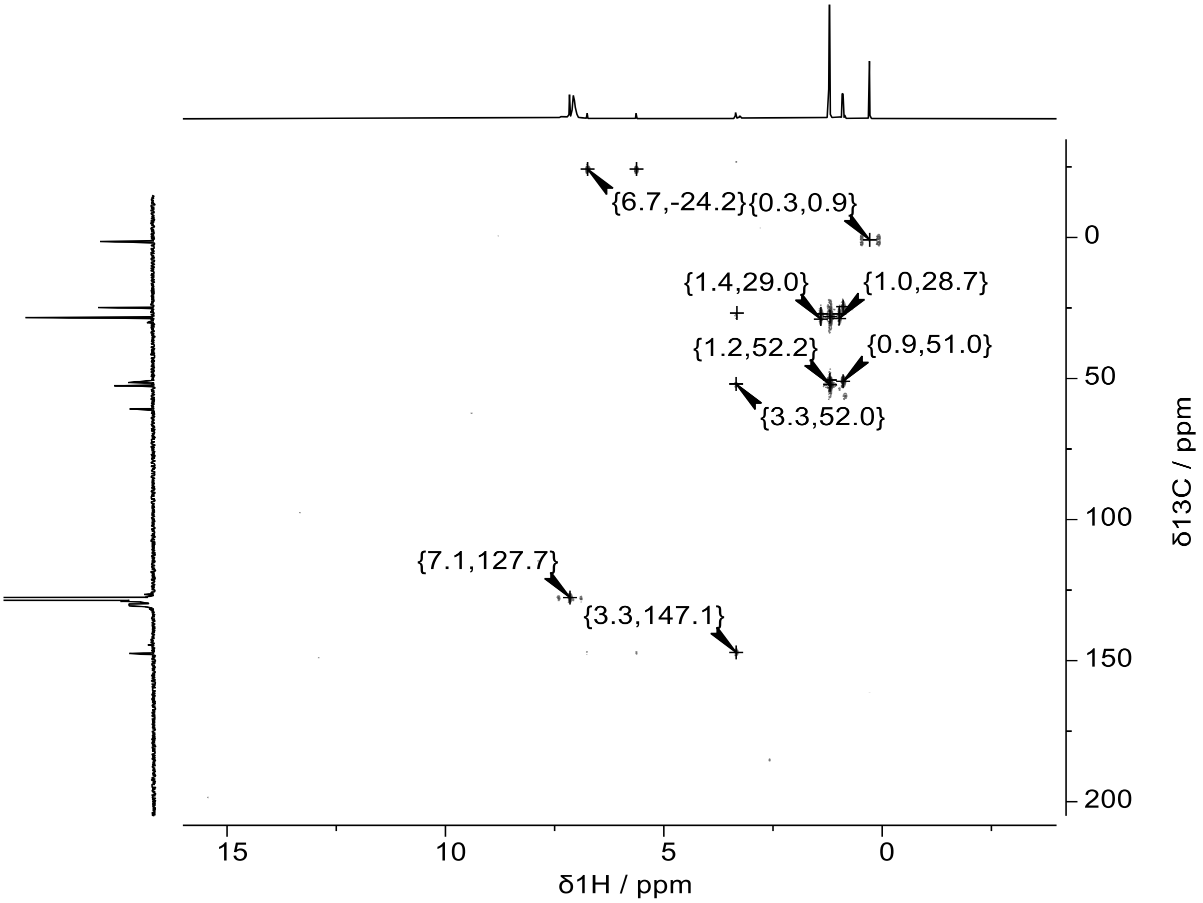

Figure 79: $^{1}H,^{13}C$ HMBC NMR spectrum (300.13 MHz, 75.47 MHz, C_6D_6, 298 K) of complex **11^{Cr}b**.

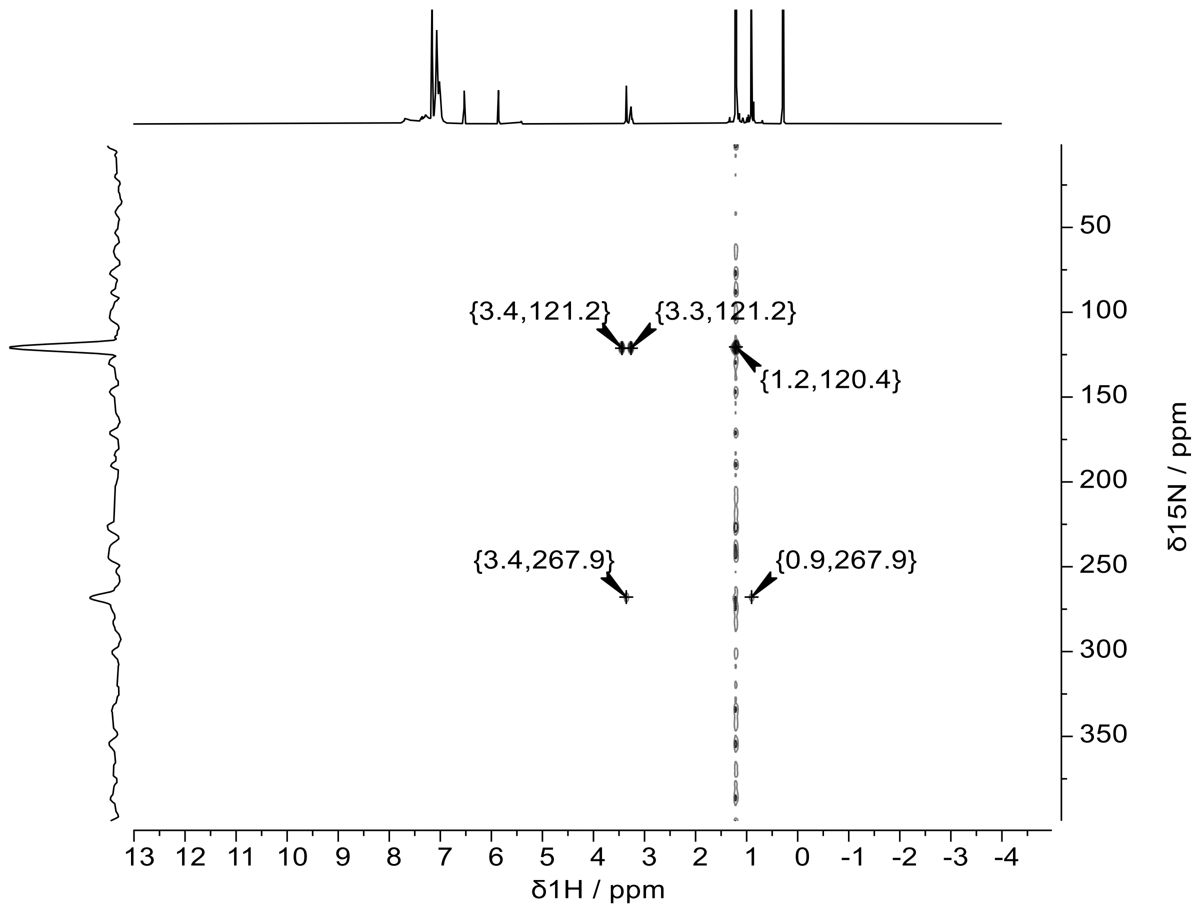

Figure 80: $^{1}H,^{15}N$ HMBC NMR spectrum (500.04 MHz, 50.68 MHz, C_6D_6, 298 K) of complex **11^{Cr}b**.

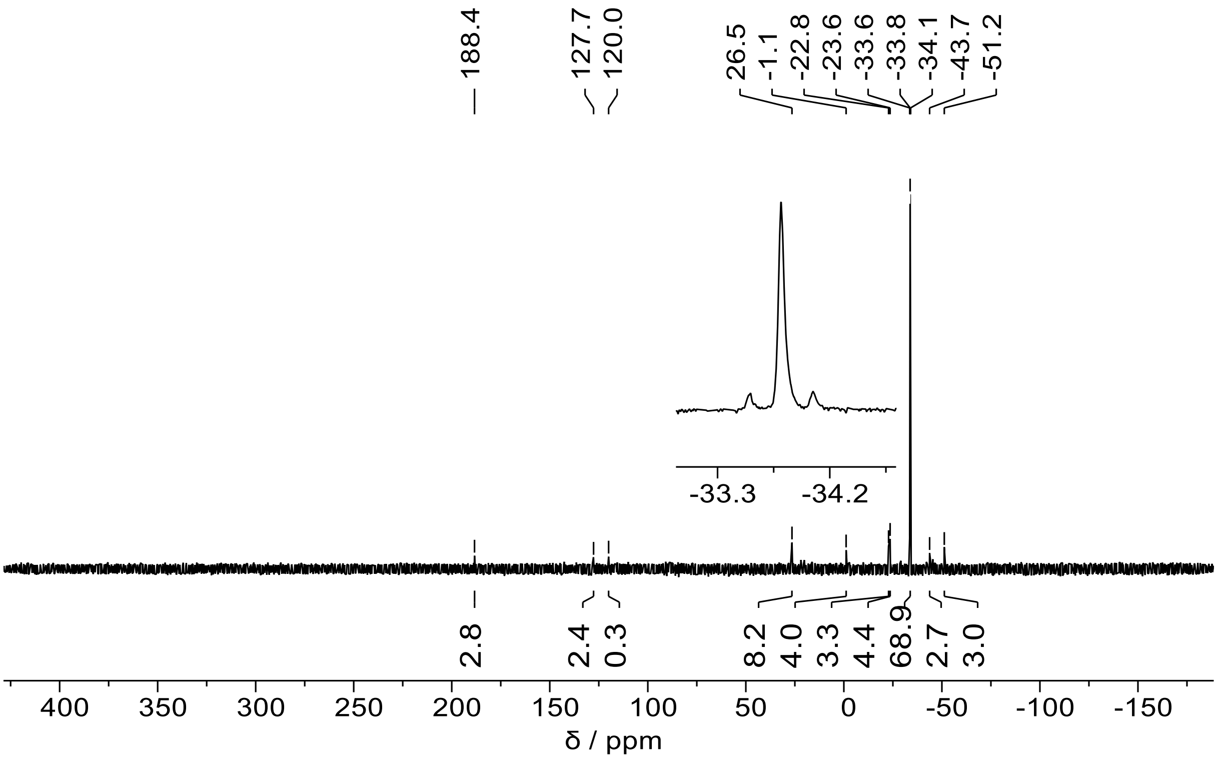

Figure 81: ^{31}P NMR spectrum (202.48 MHz, THF, 298 K) of the reaction mixture of **3d** with *tert*-butylamine under formation of the bisaminocarbene-to-phosphinidene complex adduct **12**.

4 NMR spectra of thermally treated complexes

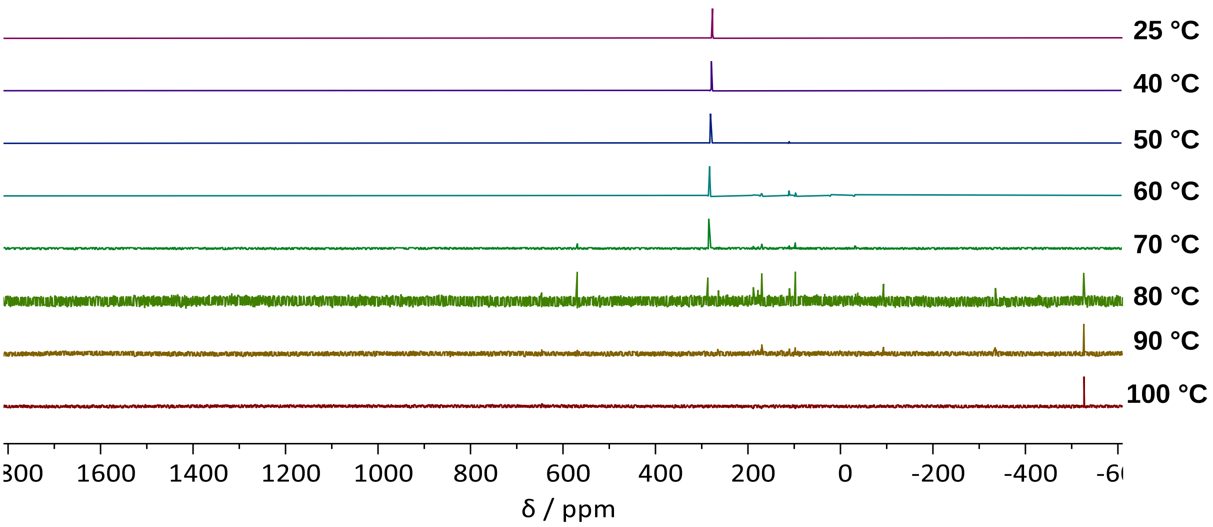

Figure 82: $^{31}P\{^1H\}$ VT NMR spectra (121.57 MHz, chlorobenzene) of a solution of **3^{Cr}b** at 25–100 °C.

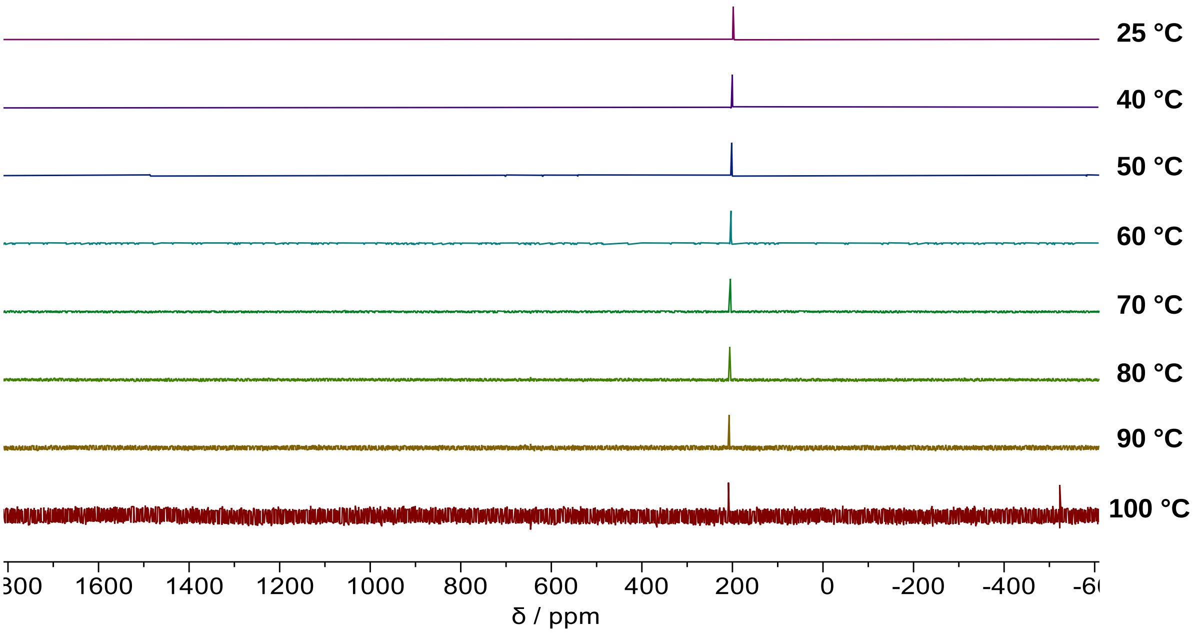

Figure 83: $^{31}P\{^1H\}$ VT NMR spectra (121.57 MHz, chlorobenzene) of a solution of **3c** at 25–100 °C.

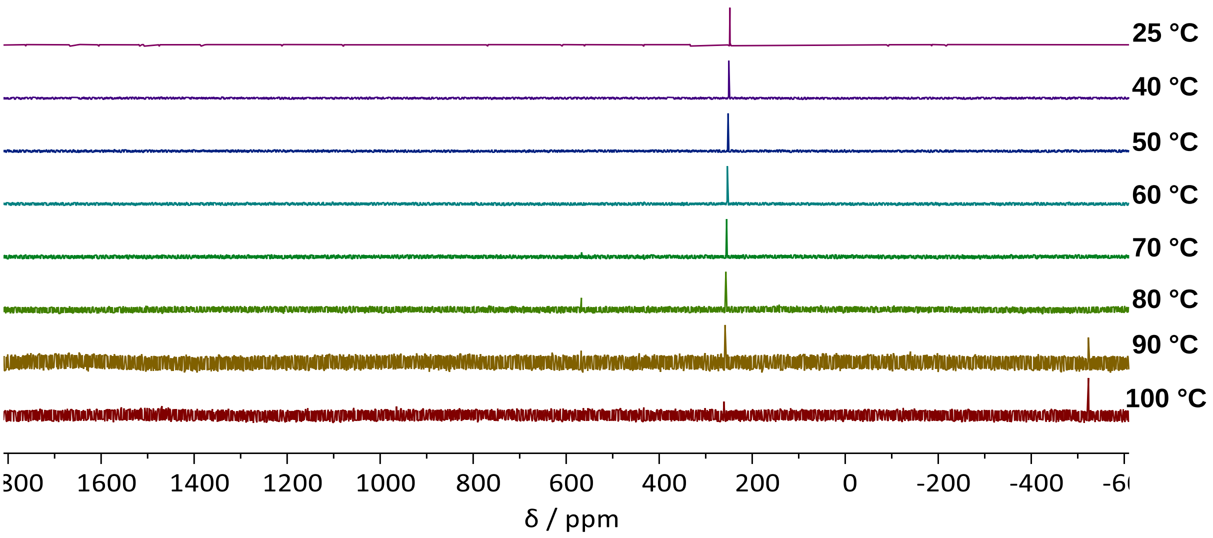

Figure 84: $^{31}P\{^1H\}$ VT NMR spectra (121.57 MHz, chlorobenzene) of a solution of **3^{Cr}c** at 25–100 °C.

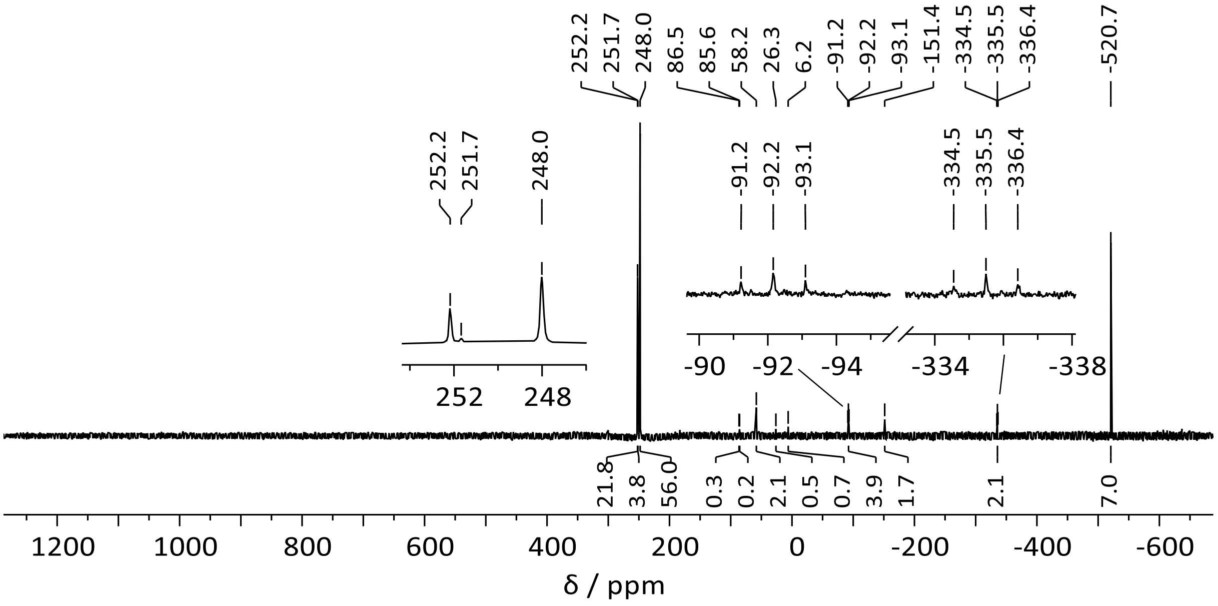

Figure 85: $^{31}P\{^1H\}$ NMR spectrum (202.48 MHz, 298 K, chlorobenzene) of a solution of **3^{Cr}c** after heating at 70 °C for 48 hours.

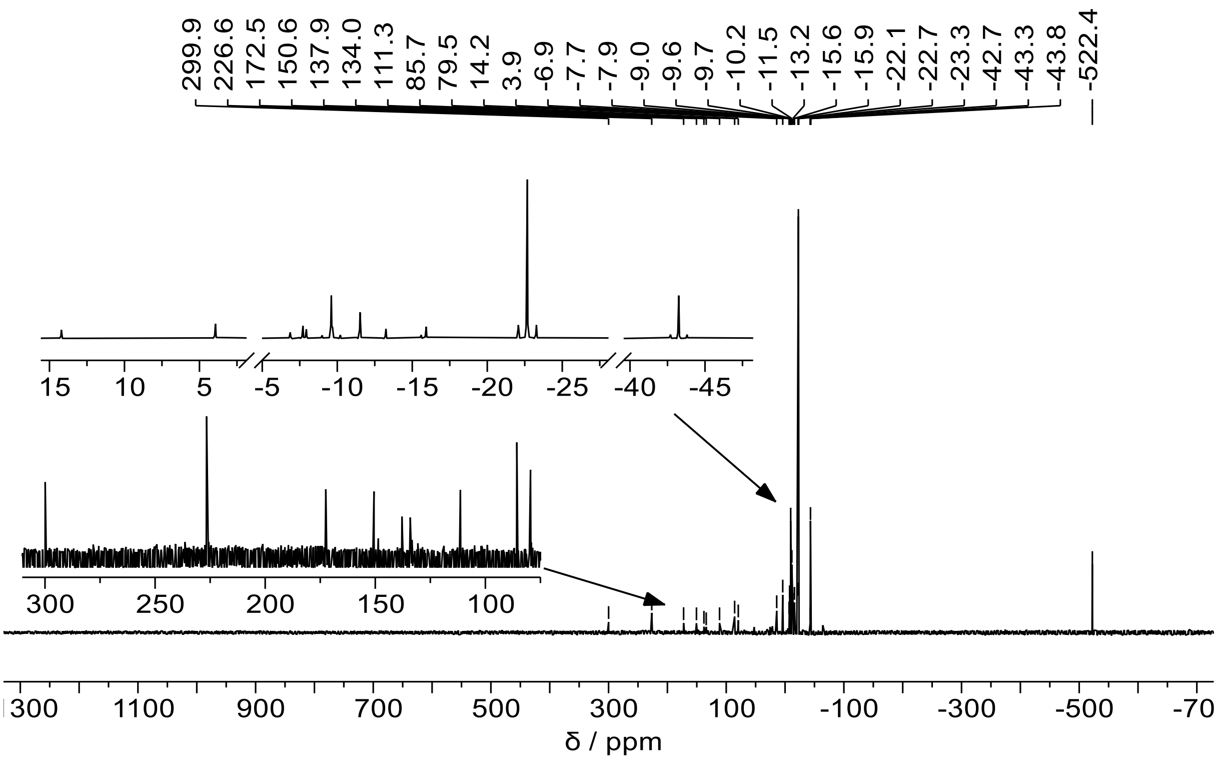

Figure 86: $^{31}P\{^{1}H\}$ NMR spectrum (202.52 MHz, chlorobenzene, 298 K) of the reaction solution of **3d** after heating at 100 °C for 17 h.

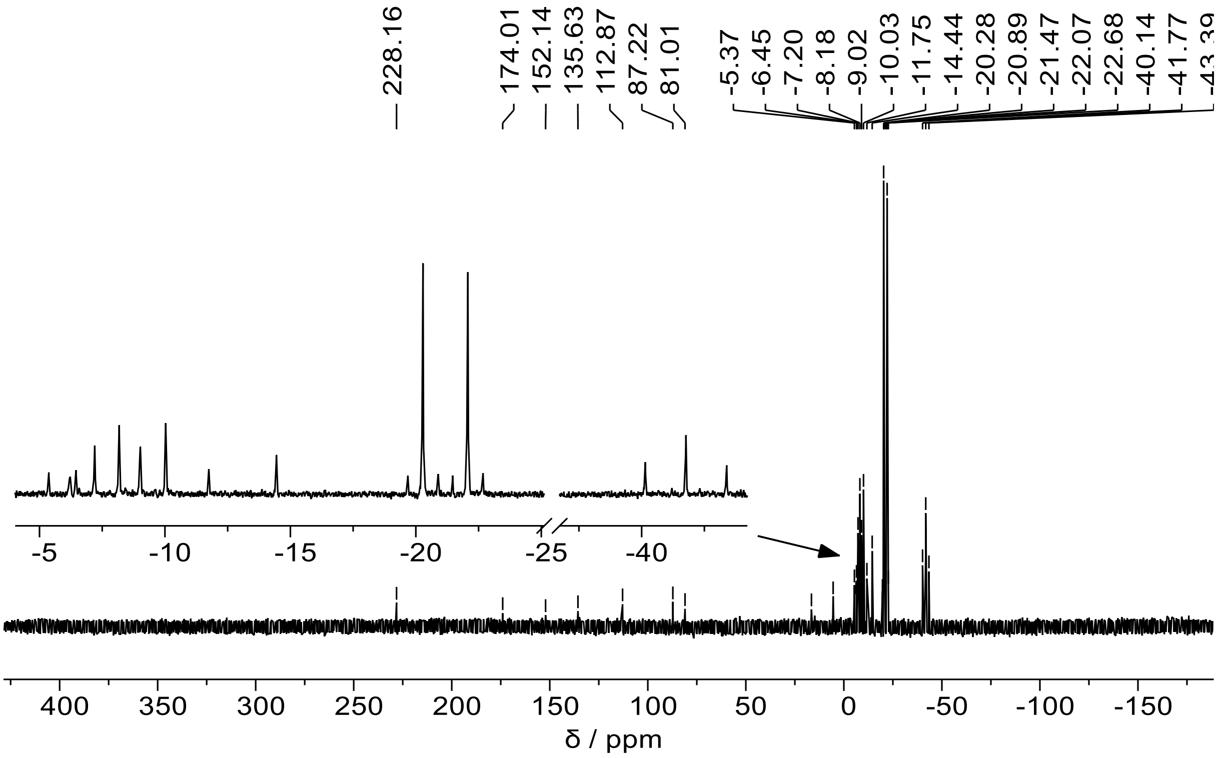

Figure 87: ^{31}P NMR spectrum (202.48 MHz, chlorobenzene, 298 K) of the reaction solution of **3d** after heating at 100 °C for 17 h.

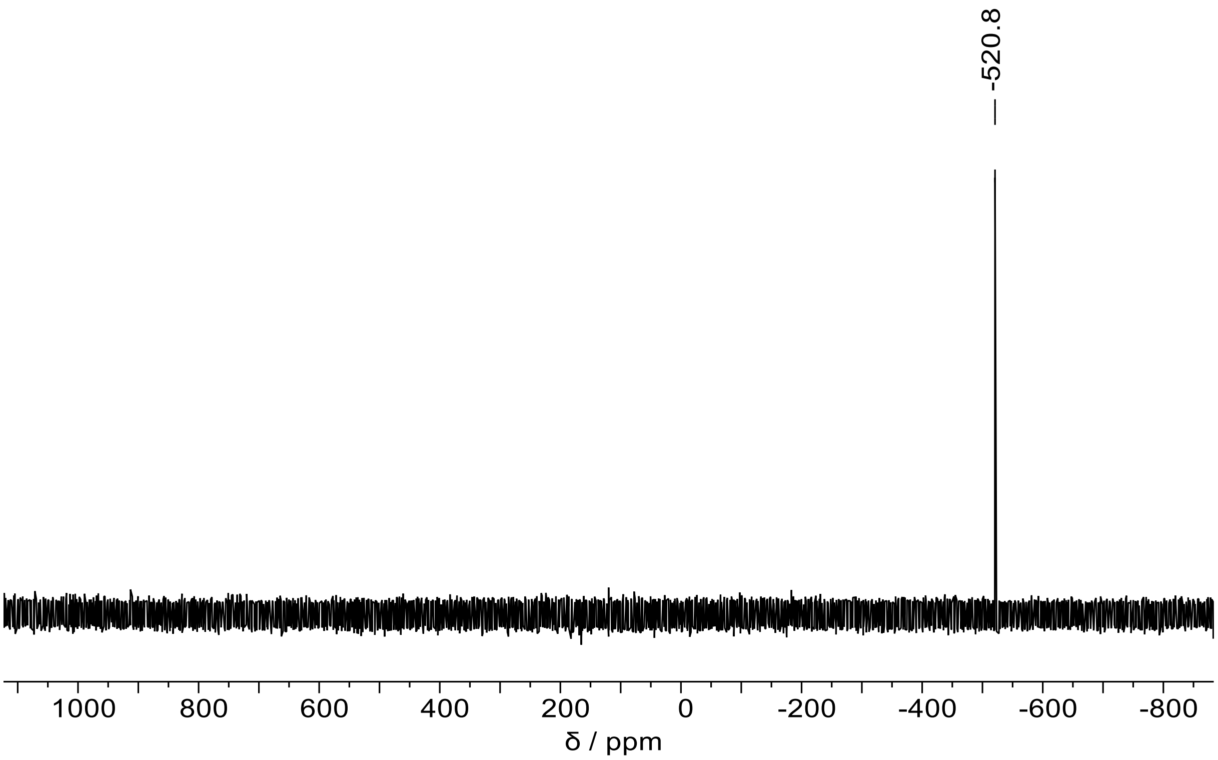

Figure 88: $^{31}P\{^1H\}$ NMR spectrum (121.51 MHz, chlorobenzene, 298 K) of a solution of $\mathbf{3^{Cr}d}$ after heating at 95 °C for 18 h.

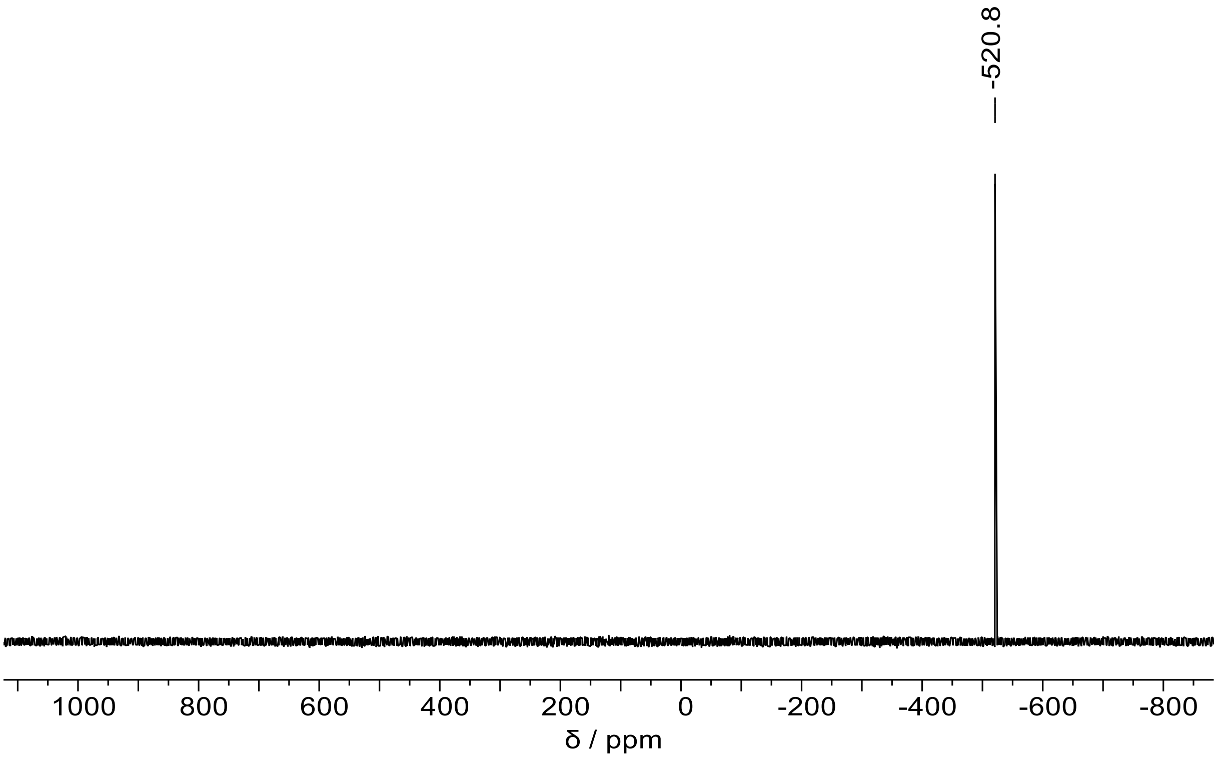

Figure 89: $^{31}P\{^1H\}$ NMR spectrum (121.51 MHz, chlorobenzene, 298 K) of a solution of $\mathbf{3^{Cr}d}$ after heating at 95 °C for 18 h with addition of P_4 as authentic sample.

5 Electrochemical experiments

The measurements of cyclic voltammograms were performed using the potentiostat and galvanostat system WaveNowXV® of Pine Research with scan rates of 20–10000 mV/s. For all CV measurements Pine research ceramic screen-printed platinum electrodes containing an Ag/AgCl reference electrode were used. These electrodes combine working, counter and reference electrodes on one ceramic plate. A low volume glass cell with a special PTFE insert at the bottom that features a narrow slit for the ceramic screen-printed electrodes was used. The internal volume of the slit is approximately 1 mL. For the experiments a 0.2 M electrolyte solution of $[^nBu_4N]PF_6$ in tetrahydrofuran was freshly prepared and used. The electrolyte was dried in vacuo (<0.02 mbar) at 80 °C for 24 h. Tetrahydrofuran was freshly purified by drying over a potassium mirror, trap-to-trap recondensation and degassing by three freeze-pump-thaw cycles. The used analyte was prepared with a concentration of 1 mM if not stated otherwise. All sample preparations and measurements were performed in a glovebox under argon atmosphere at ambient temperature. After background scans on the electrolyte solution were measured, the analyte was added. Background scans established an electrochemical window from −3.2 V to 0.06 V (3.8 V wide; potentials referenced to $Fc^{+/0}$) and identified the anodic and cathodic limits with respect to the nominal voltage of the solid silver reference. Next, open circuit potential measurements were performed to establish the starting potential of the cyclic voltammetry experiments. Careful cyclic voltammetry scans were then measured in the anodic and cathodic directions to encounter the most accessible processes, and only after these were investigated thoroughly, further scans to higher positive and negative potentials were measured. After all measurements were completed, cobaltocenium hexafluorophosphate $[(\eta^5\text{-}C_5H_5)_2Co]PF_6$ was added to a concentration of 1 mM and served as internal reference using the cobaltocenium/cobaltocene ($Cc^{+/0}$) redox couple, set to −1.35 V.[8] See Figs. 86, 91, 97, 103 for examples of referencing; sometimes showing both Cc waves. Thus, the cyclic voltammograms could be indirectly referenced to the ferrocene/ferrocenium ($Fc^{+/0}$) redox couple, set to 0 V, according to IUPAC recommendations.[9] A thorough investigation was undertaken for each sample, and scan rate dependences were measured, which show increased chemical reversibility with scan rates for the first anodic processes. Plots of the peak currents against the square root of the scan rate $\nu^{1/2}$ for these are linear, indicative of diffusion-based voltammetric behavior (i.e. occurring in solution at the interface between the solid electrode and the bulk solutions). For measurement and data processing the program Aftermath of Pine Research was used. All plots of the cyclic voltammograms were obtained using the program OriginPro 8G of OrginLab.

Table 1: Cyclic voltammetry measurement details for donor-to-phosphinidene complex adducts **3b–d**.

Compound	*m* / mg	*M* / g mol^{-1}	*n* / µmol	*c* / mmol L^{-1}
**3b**	1	720.364	1	0.5
**3c**	2.0	680.299	3	1.0
**3^{Cr}c**	1.6	548.455	3	1.0
**3d**	2.4	681.327	4	1.2

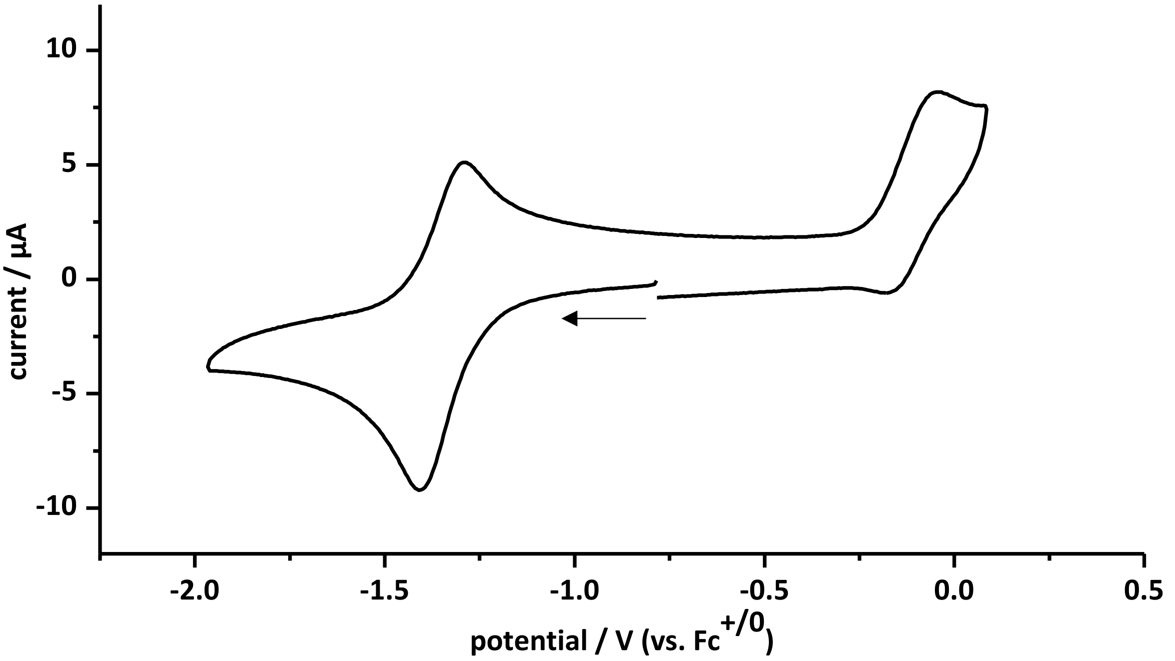

Figure 90: Cyclic voltammogram of complex **3b** (0.5 mM) at a Pt electrode in a 0.2 M $^nBu_4PF_6$/THF solution with cobaltocenium hexafluorophosphate as internal reference; measurement with cathodic initial scan direction (denoted with an arrow); scan rate: 200 mV s^{-1}; potentials are referenced against $Fc^{+/0}$.

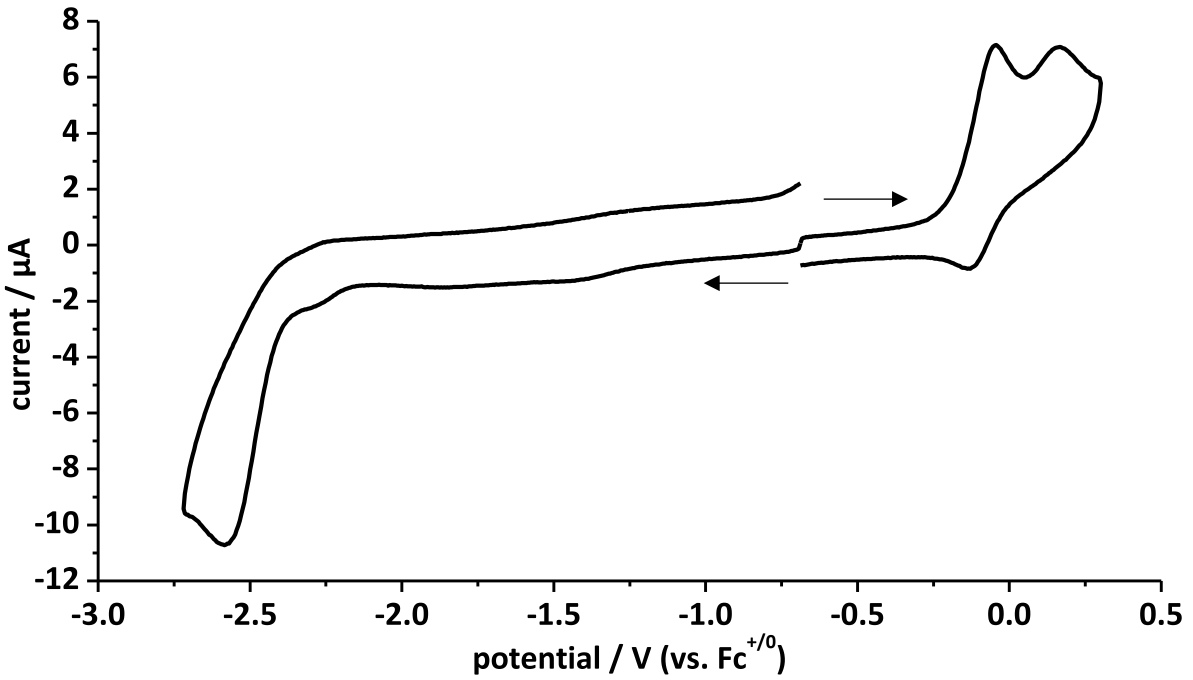

Figure 91: Overlay of cyclic voltammograms of **3b** (0.5 mM) at a Pt electrode in a 0.2 M $^nBu_4PF_6$/THF solution; oxidation parts with anodic initial scan direction and reduction parts with cathodic initial scan direction as denoted with arrows; scan rate: 200 mV s^{-1}; potentials are referenced against $Fc^{+/0}$.

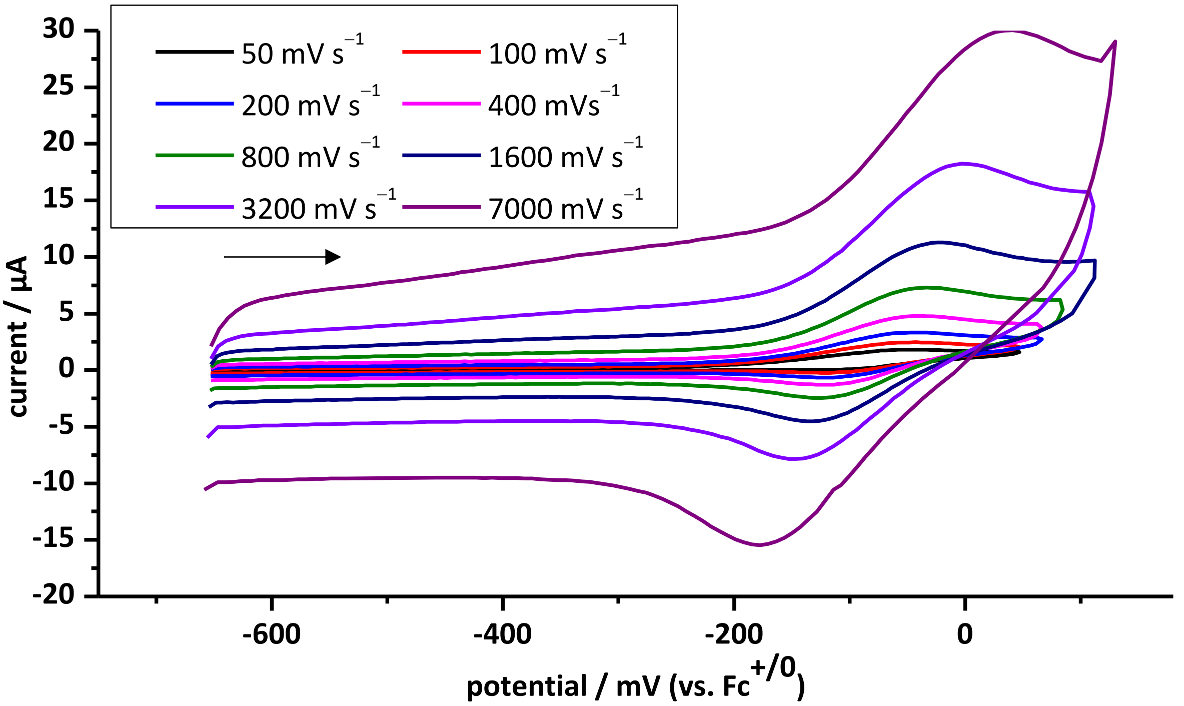

Figure 92: Cyclic voltammogram of complex **3b** (0.5 mM) at a Pt electrode in a 0.2 M $^nBu_4PF_6$/THF solution at various scan rates; measurement with anodic initial scan direction (denoted with an arrow) of the first redox process; potentials are referenced against $Fc^{+/0}$.

Table 2: Selected results of the cyclic voltametric studies of **3b** in 0.2 M $^{n}Bu_4NPF_6$/THF solution at ambient temperature. Potentials are referenced against $Fc^{+/0}$.

ν / mV s^{-1}	E_p^{Ia} / V	i_p^{Ia} / µA	E_p^{Ic} / V	i_p^{Ic} / µA	$E_{1/2}^{I}$ / V	ΔE_p^{I} / mV	$\lvert i_p^c/i_p^a \rvert$
50	−0.04	0.87	−0.13	−0.67	−0.09	85	0.77
100	−0.04	1.34	−0.12	−1.10	−0.08	76	0.82
200	−0.04	2.17	−0.12	−1.62	−0.08	75	0.75
400	−0.04	3.08	−0.12	−2.74	−0.08	81	0.89
800	−0.03	5.12	−0.13	−4.46	−0.08	92	0.87
1600	−0.02	7.28	−0.13	−8.12	−0.08	108	1.11
3200	0.00	10.9	−0.15	−14.2	−0.07	143	1.30
7000	0.04	15.8	−0.18	−27.1	−0.07	219	1.72

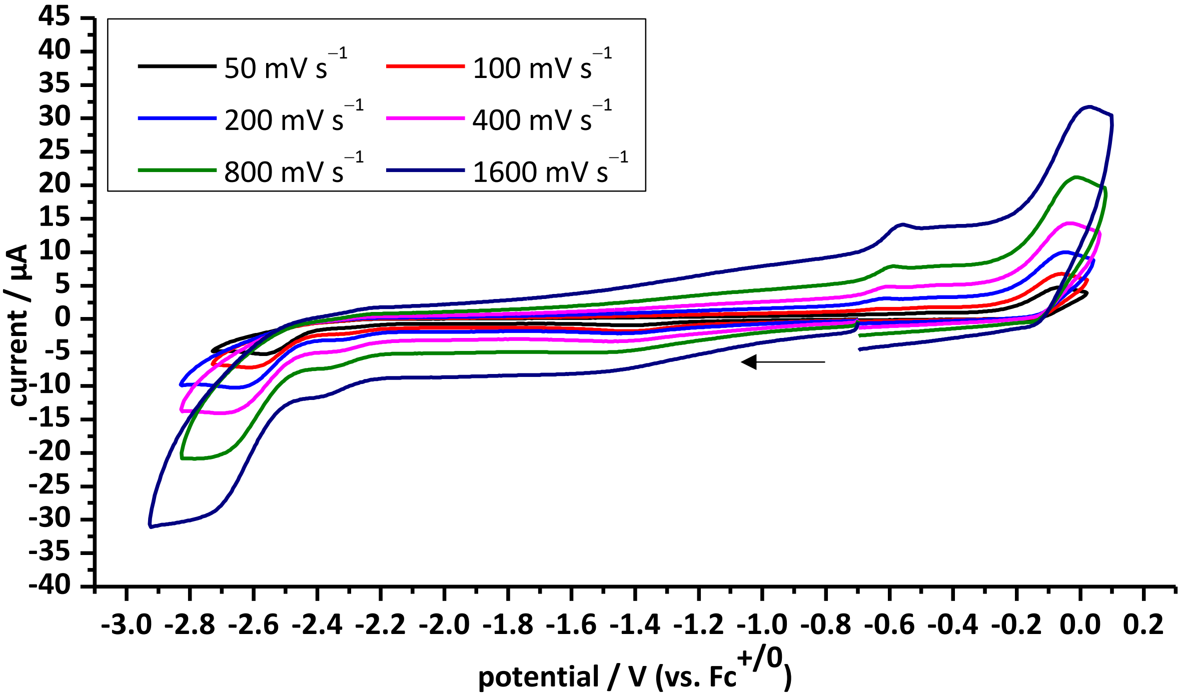

Figure 93: Cyclic voltammogram of complex **3b** (0.5 mM) at a Pt electrode in a 0.2 M $^{n}Bu_4PF_6$/THF solution at various scan rates; measurement with cathodic initial scan direction (denoted with an arrow) of the first and third redox process; potentials are referenced against $Fc^{+/0}$.

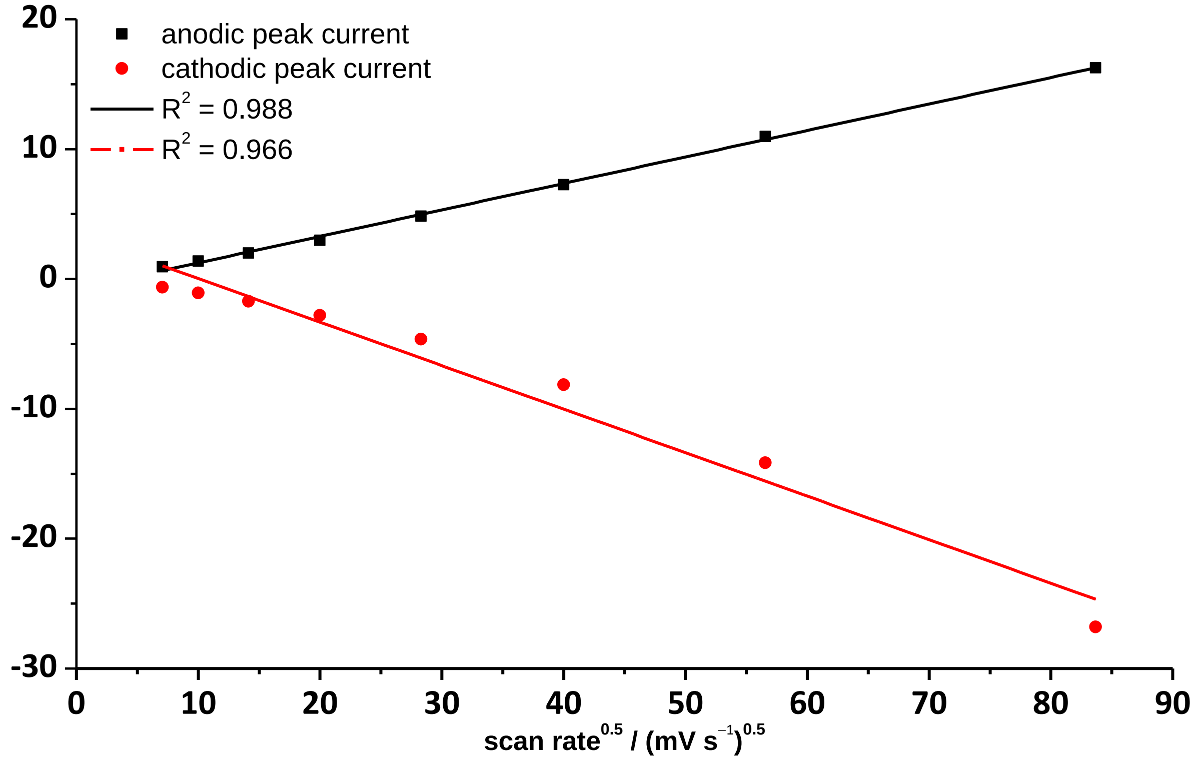

Figure 94: Plot of the peak currents against the square root of the scan rate $\nu^{0.5}$ for the first redox process of complex **3b**.

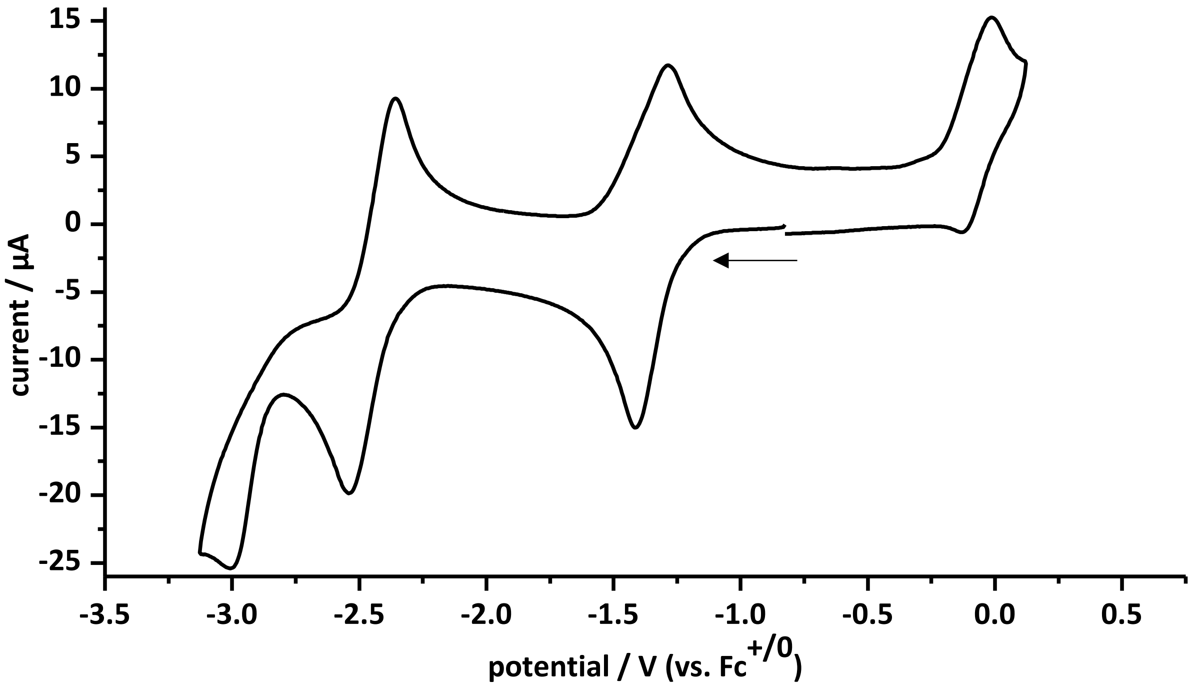

Figure 95: Cyclic voltammogram of complex **3c** (1 mM) at a Pt electrode in a 0.2 M $^{n}Bu_4PF_6$/THF solution with cobaltocenium hexafluorophosphate as internal reference; measurement with cathodic initial scan direction (denoted with an arrow); scan rate: 200 mV s^{-1}; potentials are referenced against Fc$^{+/0}$.

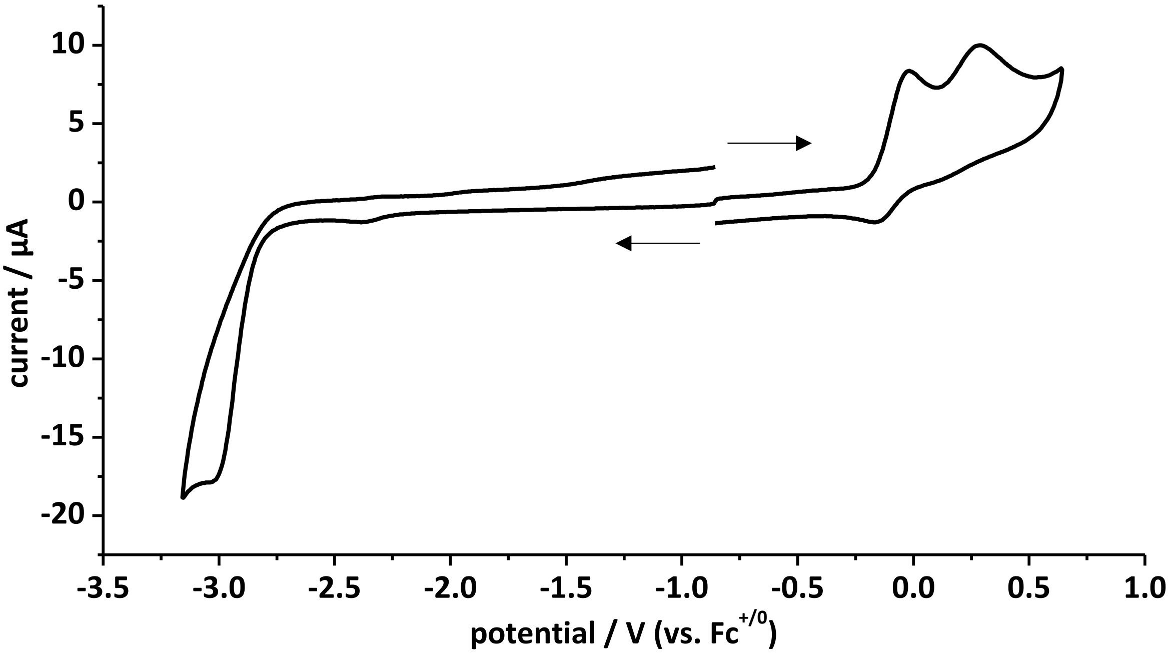

Figure 96: Overlay of cyclic voltammograms of **3c** (1 mM) at a Pt electrode in a 0.2 M $^{n}Bu_4PF_6$/THF solution; oxidation parts with anodic initial scan direction and reduction parts with cathodic initial scan direction as denoted with arrows; scan rate: 200 mV s^{-1}; potentials are referenced against $Fc^{+/0}$.

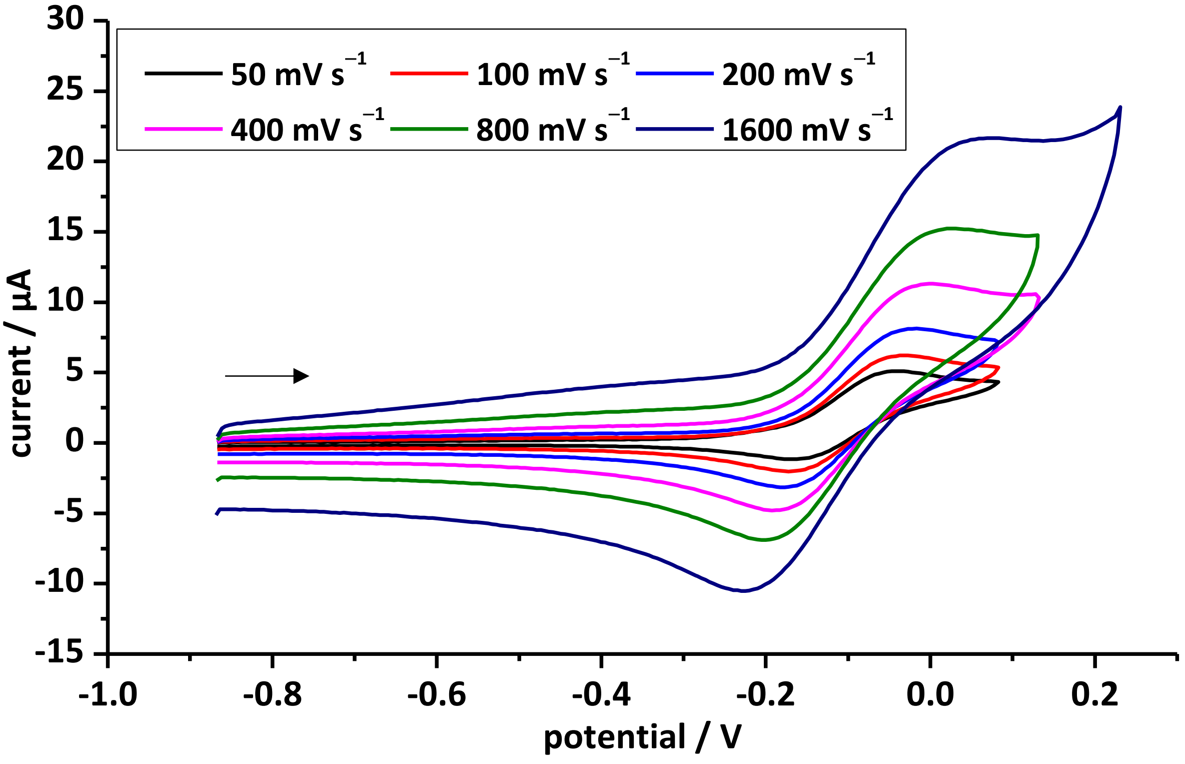

Figure 97: Cyclic voltammogram of complex **3c** (1 mM) at a Pt electrode in a 0.2 M $^{n}Bu_4PF_6$/THF solution at various scan rates; measurement with anodic initial scan direction (denoted with an arrow) of the first redox process; potentials are referenced against $Fc^{+/0}$.

Table 3: Selected results of the cyclic voltametric studies of **3c** in 0.2 M $^{n}Bu_4NPF_6$/THF solution at ambient temperature. Potentials are referenced against $Fc^{+/0}$.

ν / mV s^{-1}	E_p^{Ia} / V	i_p^{Ia} / µA	E_p^{Ic} / V	i_p^{Ic} / µA	$E_{1/2}^{I}$ / V	ΔE_p^{I} / mV	$\lvert i_p^c/i_p^a \rvert$
50	−0.04	2.69	−0.16	−2.46	−0.10	125	0.91
100	−0.03	3.39	−0.17	−3.33	−0.10	140	0.98
200	−0.02	4.71	−0.18	−4.69	−0.10	166	1.00
400	0.00	7.03	−0.19	−6.98	−0.10	191	0.99
800	0.03	9.80	−0.21	−10.0	−0.09	230	1.02
1600	0.07	15.4	−0.23	−15.4	−0.08	294	1.00

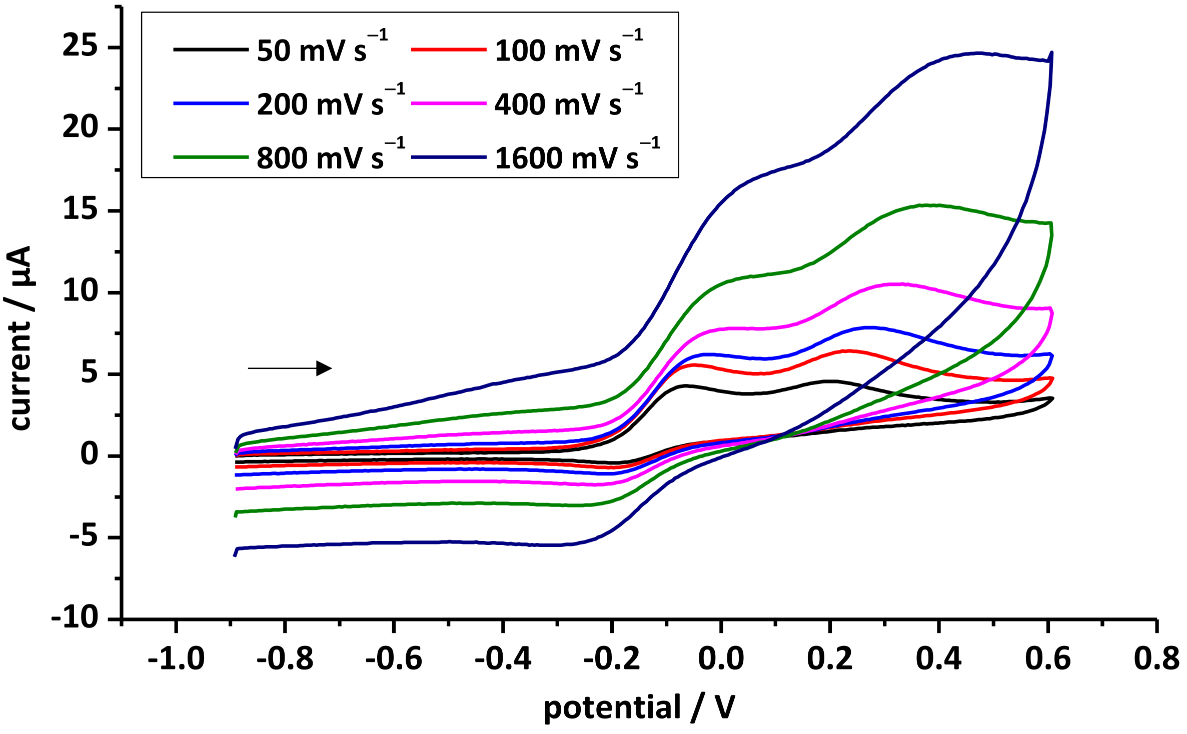

Figure 98: Cyclic voltammogram of complex **3c** (1 mM) at a Pt electrode in a 0.2 M $^{n}Bu_4PF_6$/THF solution at various scan rates; measurement with anodic initial scan direction (denoted with an arrow) of the first and second redox processes; potentials are referenced against $Fc^{+/0}$.

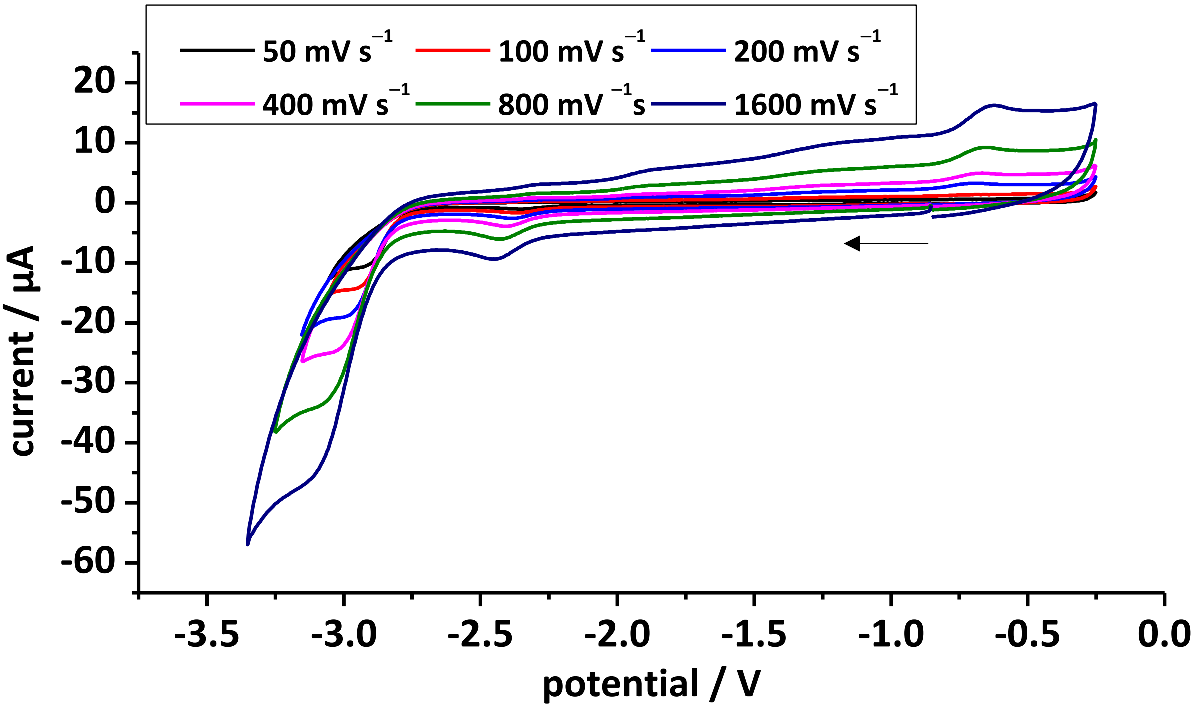

Figure 99: Cyclic voltammogram of complex **3c** (1 mM) at a Pt electrode in a 0.2 M $^{n}Bu_4PF_6$/THF solution at various scan rates; measurement with cathodic initial scan direction (denoted with an arrow) of the third redox process; potentials are referenced against $Fc^{+/0}$.

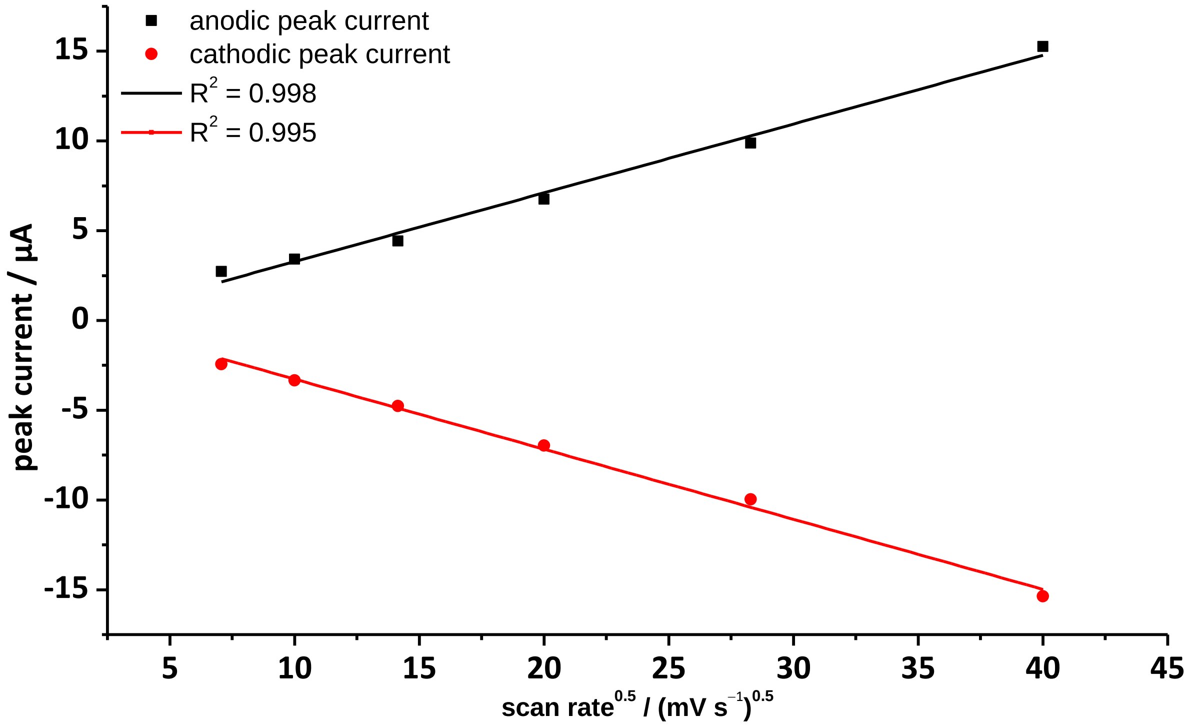

Figure 100: Plot of the peak currents against the square root of the scan rate $\nu^{0.5}$ for the first redox process of complex **3c**.

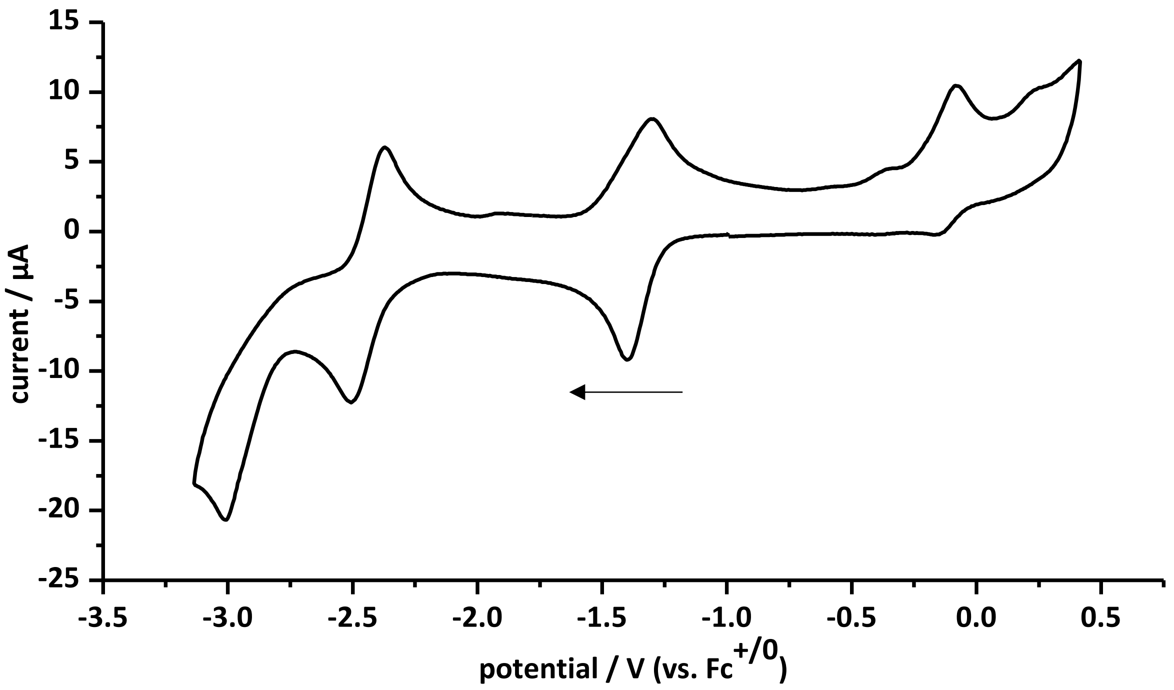

Figure 101: Cyclic voltammogram of complex **3^{Cr}c** (1 mM) at a Pt electrode in a 0.2 M $^{n}Bu_4PF_6$/THF solution with cobaltocenium hexafluorophosphate as internal reference; measurement with cathodic initial scan direction (denoted with an arrow); scan rate: 200 mV s^{-1}; potentials are referenced against Fc$^{+/0}$.

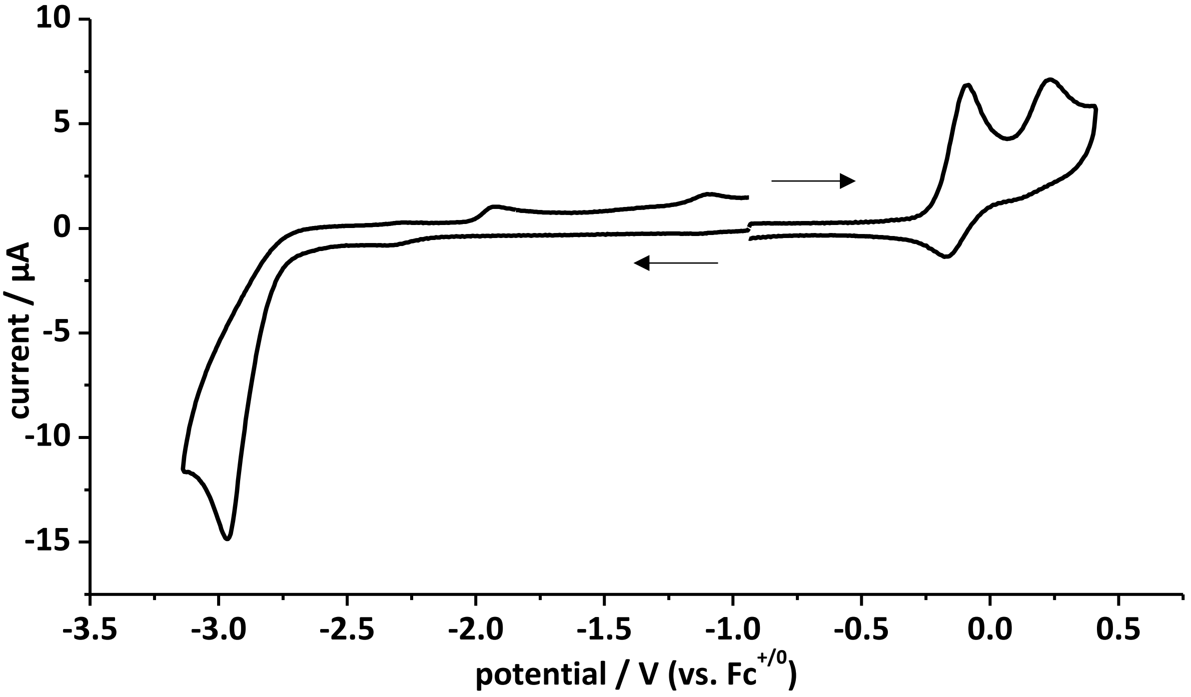

Figure 102: Overlay of cyclic voltammograms of **3^{Cr}c** (1 mM) at a Pt electrode in a 0.2 M $^{n}Bu_4PF_6$/THF solution; oxidation parts with anodic initial scan direction and reduction parts with cathodic initial scan direction as denoted with arrows; scan rate: 200 mV s^{-1}; potentials are referenced against Fc$^{+/0}$.

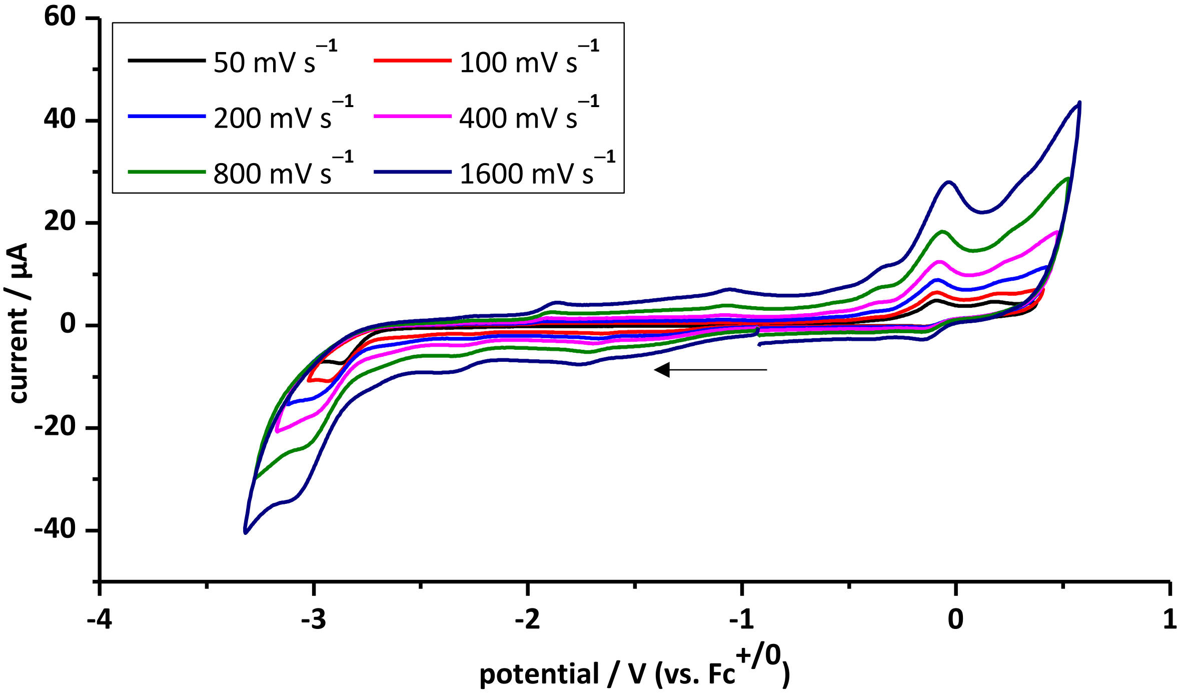

Figure 103: Cyclic voltammogram of complex **3^{Cr}c** (1 mM) at a Pt electrode in a 0.2 M $^{n}Bu_4PF_6$/THF solution at various scan rates; measurement with cathodic initial scan direction (denoted with an arrow); potentials are referenced against $Fc^{+/0}$.

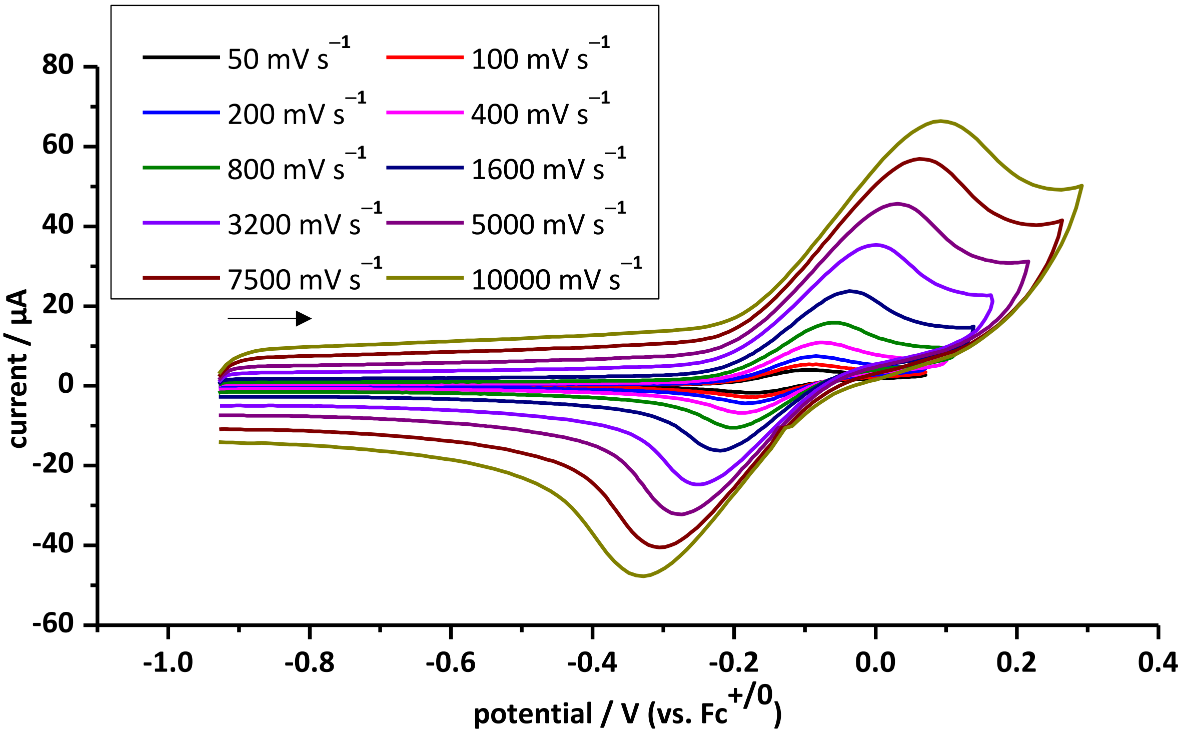

Figure 104: Cyclic voltammogram of complex **3^{Cr}c** (1 mM) at a Pt electrode in a 0.2 M $^{n}Bu_4PF_6$/THF solution at various scan rates; measurement with anodic initial scan direction (denoted with an arrow) of the first redox process; potentials are referenced against $Fc^{+/0}$.

Table 4: Selected results of the cyclic voltametric studies of **3^{Cr}c** in 0.2 M $^{n}Bu_4NPF_6$/THF solution at ambient temperature. Potentials are referenced against $Fc^{+/0}$.

ν / mV s^{-1}	E_p^{Ia} / V	i_p^{Ia} / µA	E_p^{Ic} / V	i_p^{Ic} / µA	$E_{1/2}^{I}$ / V	ΔE_p^{I} / mV	$\lvert i_p^c/i_p^a \rvert$
50	−0.10	2.69	−0.17	−2.62	0.14	75	0.97
100	−0.09	3.81	−0.18	−3.85	0.14	85	1.01
200	−0.08	5.38	−0.18	−5.63	0.13	100	1.05
400	−0.07	8.23	−0.18	−8.52	0.13	110	1.03
800	−0.06	12.4	−0.20	−12.8	0.13	139	1.03
1600	−0.04	18.8	−0.22	−19.6	0.13	183	1.04
3200	0.00	29.7	−0.25	−29.6	0.12	248	1.00
5000	0.03	36.9	−0.27	−39.5	0.12	305	1.07
7500	0.06	44.7	−0.31	−50.7	0.12	368	1.13
10000	0.09	49.90	−0.33	−61.0	0.12	420	1.22

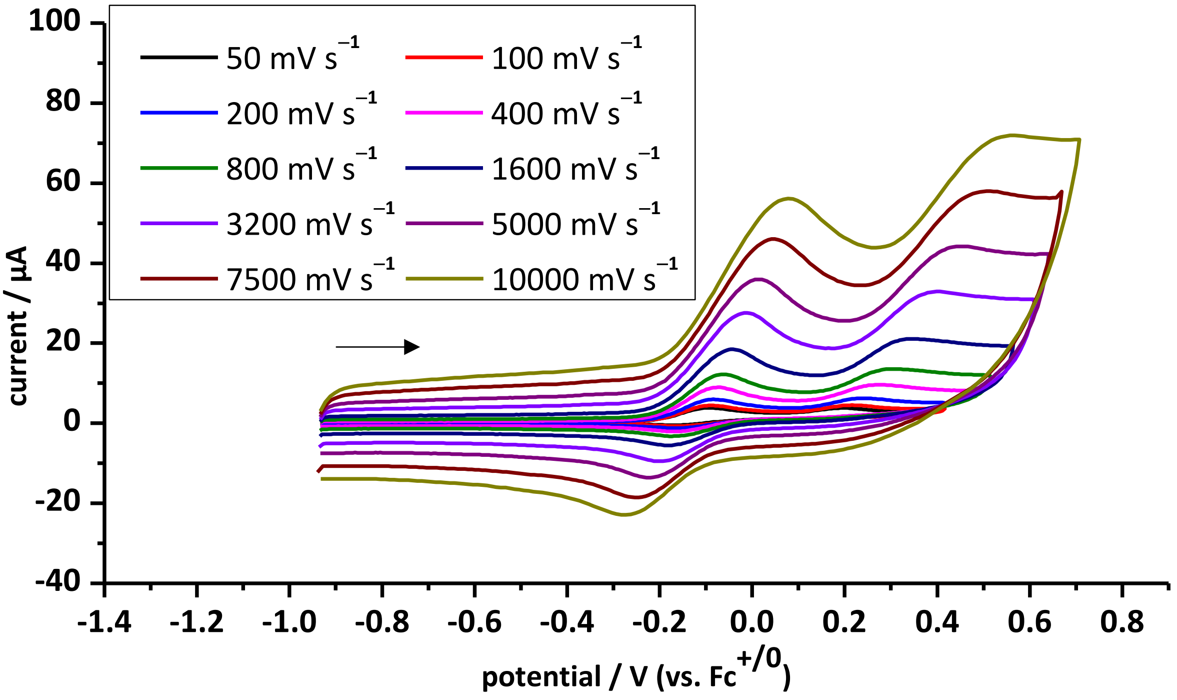

Figure 105: Cyclic voltammogram of complex **3^{Cr}c** (1 mM) at a Pt electrode in a 0.2 M $^{n}Bu_4PF_6$/THF solution at various scan rates; measurement with anodic initial scan direction (denoted with an arrow) of the first and second redox processes; potentials are referenced against $Fc^{+/0}$.

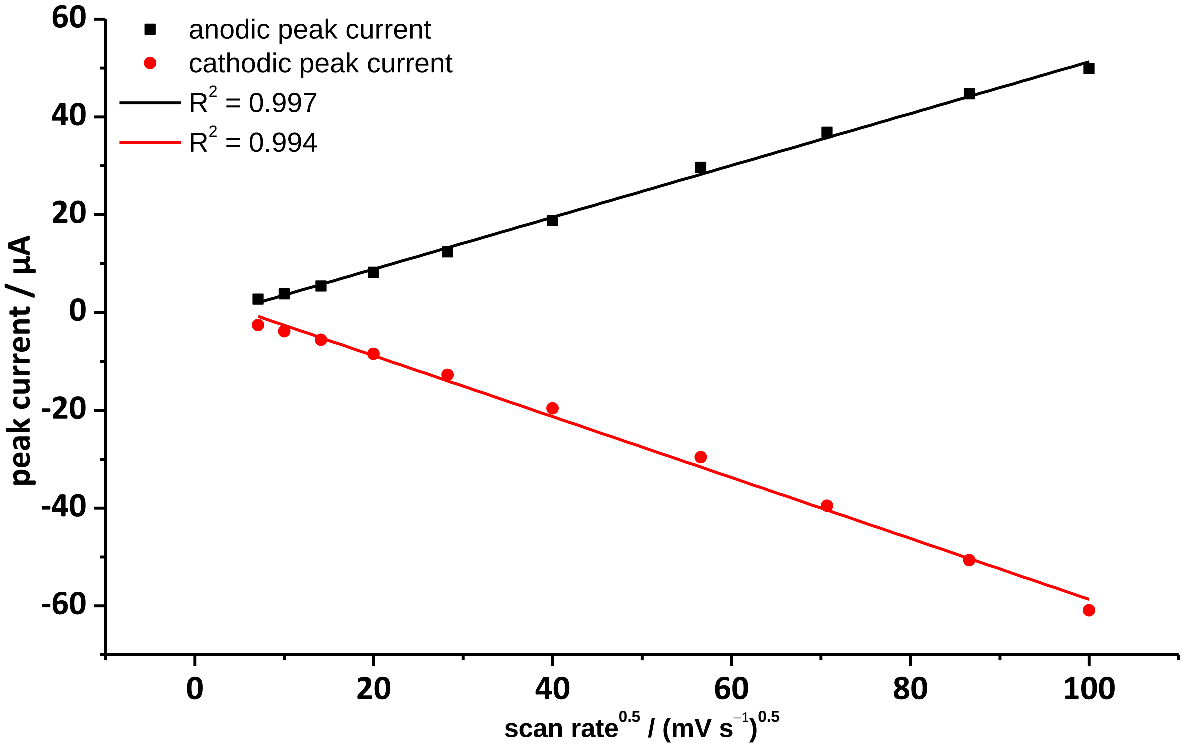

Figure 106: Plot of the peak currents against the square root of the scan rate $v^{0.5}$ for the first redox process of complex **3**^{Cr}**c**.

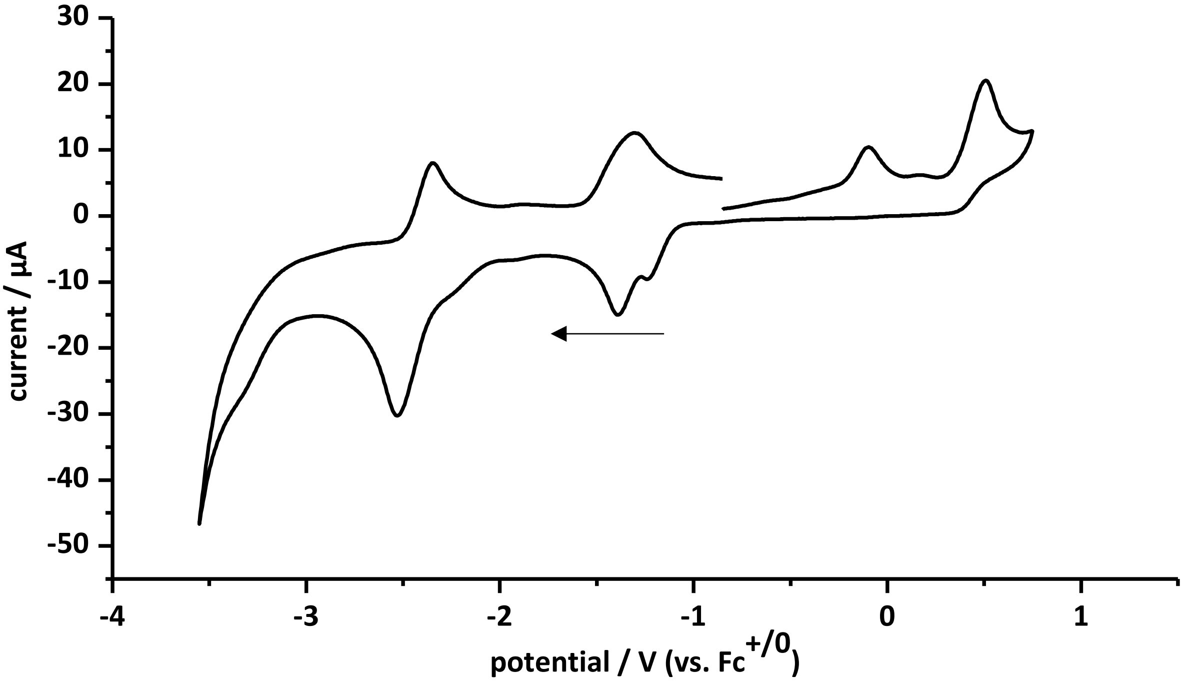

Figure 107: Cyclic voltammogram of complex **3d** (1 mM) at a Pt electrode in a 0.2 M $^{n}Bu_4PF_6$/THF solution with cobaltocenium hexafluorophosphate as internal reference; measurement with cathodic

initial scan direction (denoted with an arrow); scan rate: 200 mV s^{-1}; potentials are referenced against Fc$^{+/0}$.

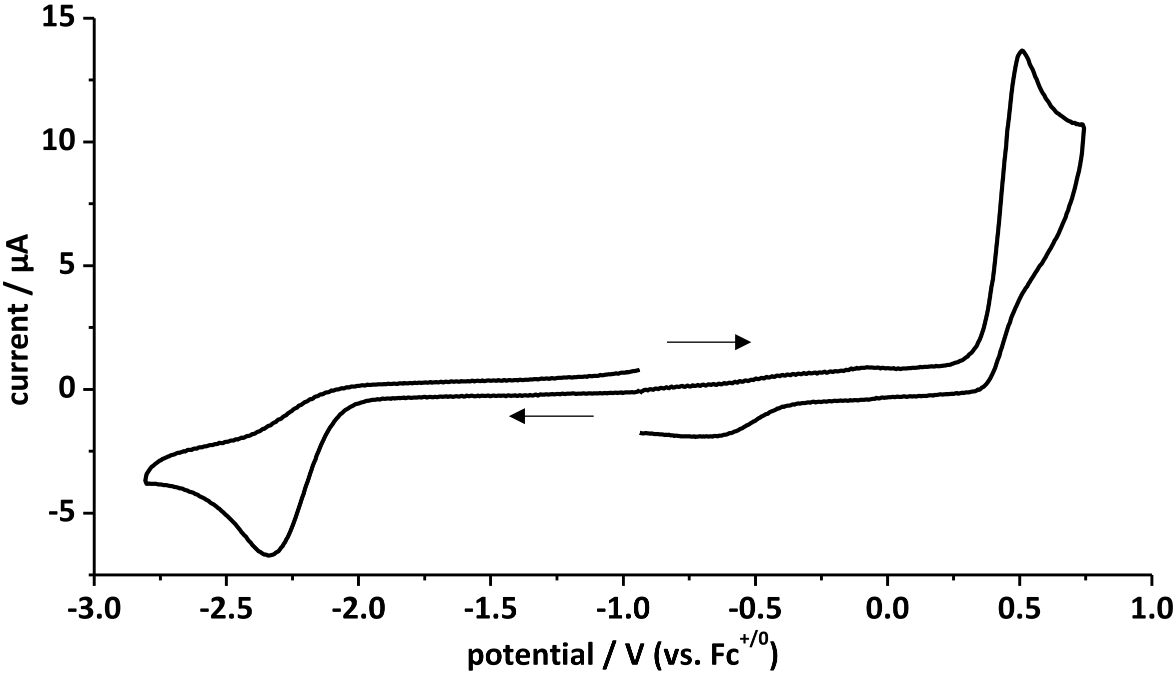

Figure 108: Overlay of cyclic voltammograms of **3d** (1 mM) at a Pt electrode in a 0.2 M $^{n}Bu_4PF_6$/THF solution; oxidation parts with anodic initial scan direction and reduction parts with cathodic initial scan direction as denoted with arrows; scan rate: 200 mV s^{-1}; potentials are referenced against Fc$^{+/0}$.

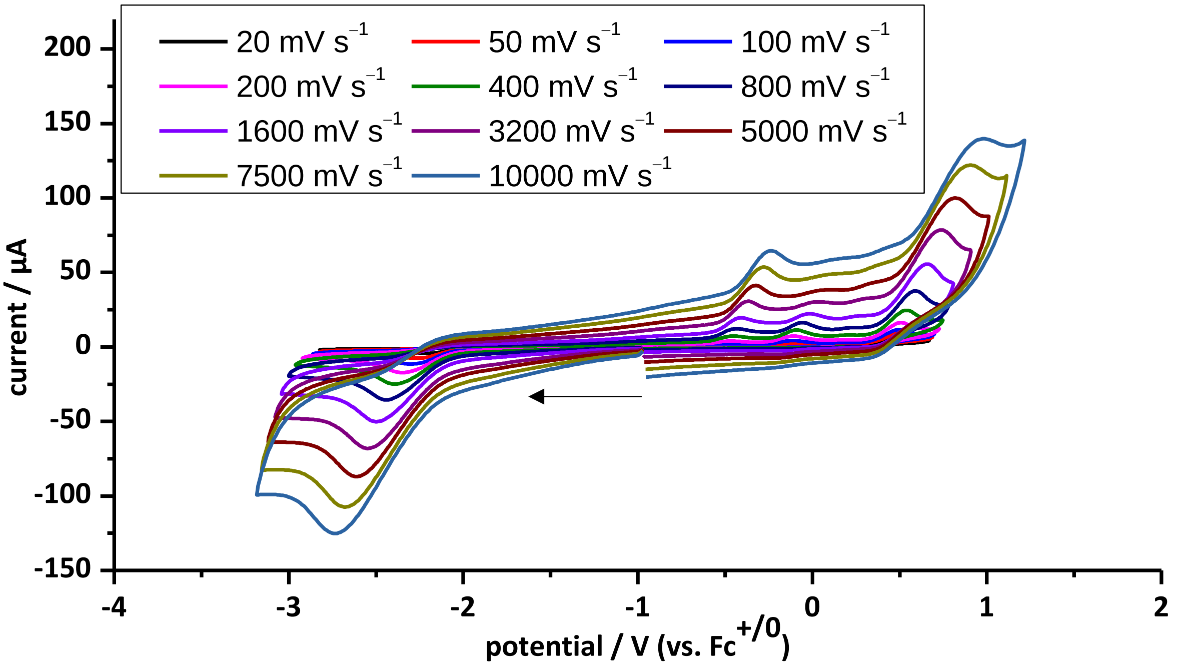

Figure 109: Cyclic voltammogram of complex **3d** (1 mM) at a Pt electrode in a 0.2 M $^{n}Bu_4PF_6$/THF solution at various scan rates; measurement with cathodic initial scan direction (denoted with an arrow); potentials are referenced against $Fc^{+/0}$.

Table 5: Selected results of the cyclic voltametric studies of **3d** in 0.2 M $^{n}Bu_4NPF_6$/THF solution at ambient temperature. Potentials are referenced against $Fc^{+/0}$.

ν / mV s^{-1}	E_p^{Ia} / V	i_p^{Ia} / μA	E_p^{IIc} / V	i_p^{IIc} / μA	E_p^{IIIa} / V	i_p^{IIIa} / μA	$\lvert i_p^{IIIa}/i_p^{IIc}\rvert$
20	0.47	7.28	−2.20	−4.28	−0.15	0.68	0.16
50	0.48	8.72	−2.26	−7.50	−0.13	2.51	0.33
100	0.49	11.6	−2.30	−11.4	−0.11	4.33	0.38
200	0.51	16.4	−2.35	−17.2	−0.11	7.31	0.42
400	0.54	24.6	−2.39	−24.9	−0.09	11.4	0.46
800	0.59	37.6	−2.44	−35.6	−0.05	16.4	0.46
1600	0.66	55.6	−2.50	−50.1	−0.01	22.4	0.45
3200	0.74	78.6	−2.55	−68.1	0.03	30.3	0.44
5000	0.82	100	−2.61	−87.0	0.11	38.4	0.44
7500	0.90	122	−2.68	−107	0.14	49.2	0.46
10000	0.98	140	−2.73	−125	0.21	59.9	0.48

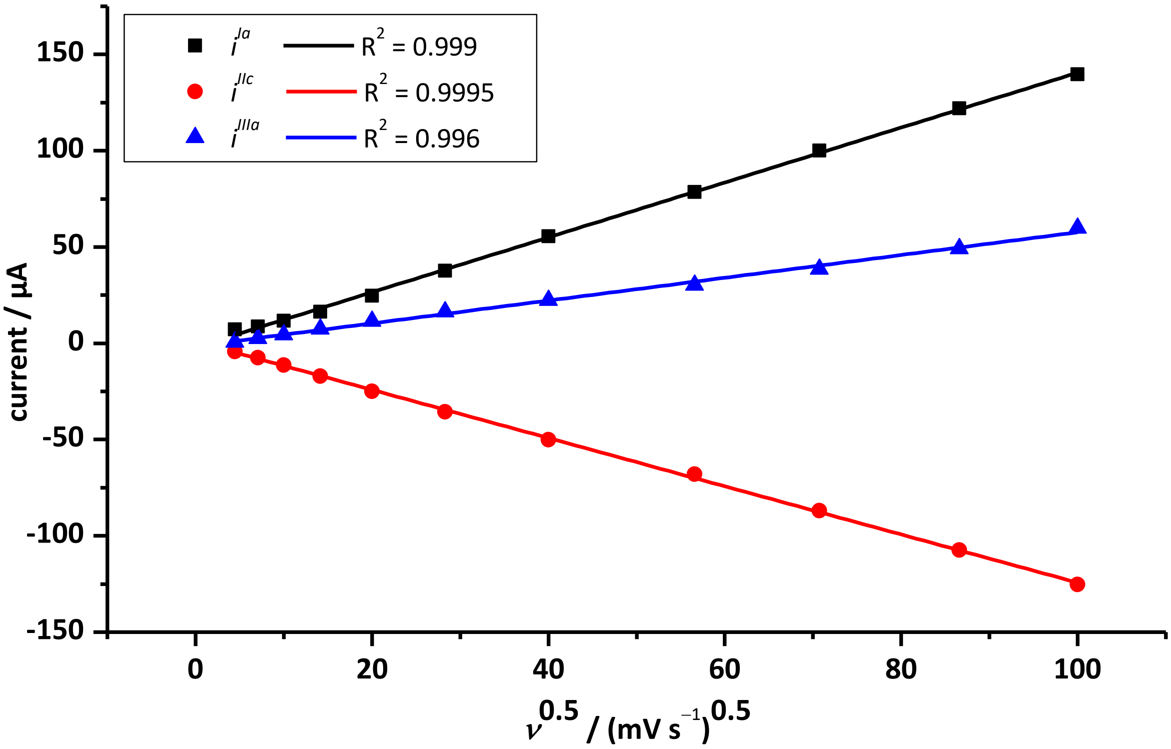

Figure 110: Plot of the peak currents against the square root of the scan rate $\nu^{0.5}$ of **3d**.

6 X-ray diffraction studies

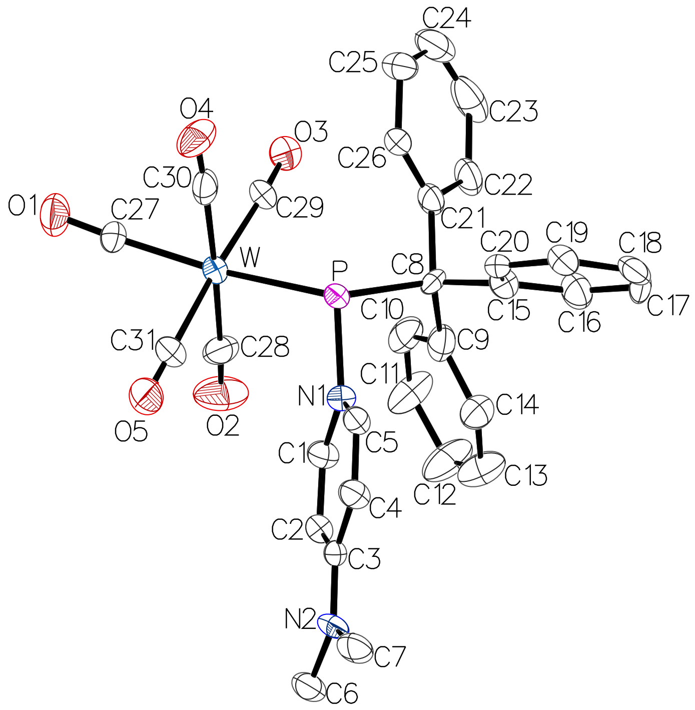

Figure 111: Molecular structures of **3b** in the single crystal lattice at 123(2) K. Thermal ellipsoids are set at 50% probability. Hydrogen atoms and solvent molecules were omitted for clarity. Suitable single crystals were obtained as clear yellow planks by slow evaporation of a solution of 1.4 mg of **3b** in 1.5 mL of diethyl ether at ambient temperature in a glovebox. CCDC 2250849.

Alert level B

PLAT972_ALERT_2_B Check Calcd Resid. Dens. 0.90Ang From W -3.42 eA-3

Author Response: Peaks of this height close to W (AN = 74) are often encountered and not noteworthy.

PLAT972_ALERT_2_B Check Calcd Resid. Dens. 0.93Ang From W -2.95 eA-3

Author Response: Peaks of this height close to W (AN = 74) are often encountered and not noteworthy.

PLAT972_ALERT_2_B Check Calcd Resid. Dens. 1.00Ang From W -2.89 eA-3

Author Response: Peaks of this height close to W (AN = 74) are often encountered and not noteworthy.

PLAT972_ALERT_2_B Check Calcd Resid. Dens. 1.01Ang From W -2.59 eA-3

Author Response: Peaks of this height close to W (AN = 74) are often encountered and not noteworthy.

Table 6: Crystal data and structure refinements for **3b**.

Identification code	GSTR741, DB-425 // GXray6737
Crystal habitus	clear yellow plank
Device type	STOE IPDS-2T
Empirical formula	$C_{66}H_{60}N_4O_{11}P_2W_2$
Moiety formula	2 ($C_{31}H_{25}N_2O_5PW$), $C_4H_{10}O$
Formula weight / g/mol	1514.82
T / K	123
Crystal system	monoclinic
Space group	$P2_1/c$
a / Å	15.8124(12)
b / Å	9.6562(6)
c / Å	20.8092(16)
α / °	90
β / °	93.151(6)
γ / °	90
V / Å^3	3172.5(4)
Z	2
ρ_{calc} / g/cm^3	1.586
μ / mm^{-1}	3.735
F(000)	1500.0
Crystal size / mm^3	0.35 × 0.12 × 0.04
Absorption correction	integration
Min. and max. transmission	0.4355 and 0.7479
Radiation	Mo-K$_\alpha$ (λ = 0.71073 Å)
2Θ range for data collection / °	5.372 to 55.992
Completeness to Θ	0.994
Index ranges	$-20 \leq h \leq 20$, $-12 \leq k \leq 12$, $-24 \leq l \leq 27$
Reflections collected	23804
Independent reflections	7604 (R_{int} = 0.1071, R_σ = 0.1868)
Data / restraints / parameters	7604 / 35 / 410
Goodness-of-fit on F^2	0.784
Final *R* indexes ($I \geq 2\sigma(I)$)	R_1 = 0.0540, ωR_2 = 0.1099
Final *R* indexes (all data)	R_1 = 0.1299, ωR_2 = 0.1311
Largest diff. peak and hole / e/Å^3	2.25 and −4.25

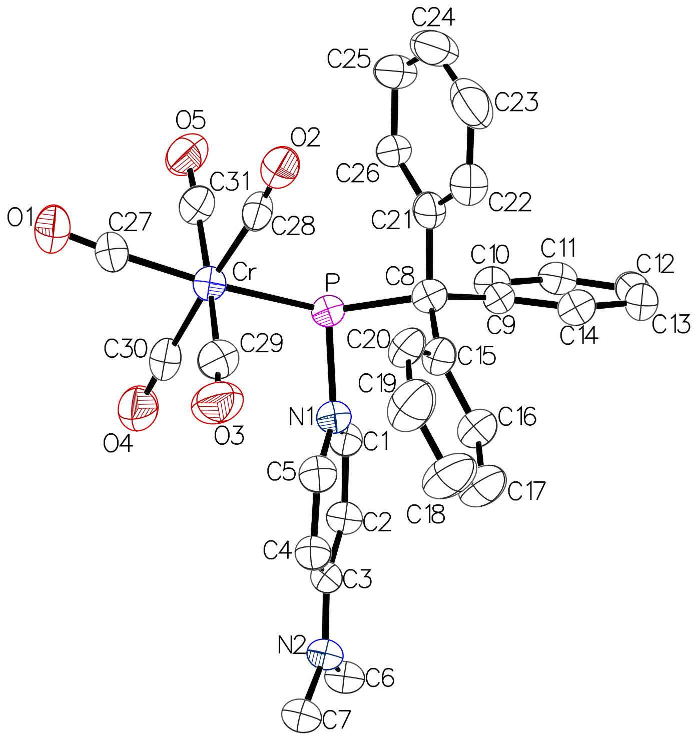

Figure 112: Molecular structures of **3**^{Cr}**b** in the single crystal lattice at 123(2) K. Thermal ellipsoids are set at 50% probability. Hydrogen atoms and solvent molecules were omitted for clarity. Suitable single crystals were obtained as clear yellow plates by slow evaporation of a solution of 4 mg of **3**^{Cr}**b** in 1.5 mL of diethyl ether at ambient temperature in a glovebox. CCDC 2250850.

Table 7: Crystal data and structure refinements for **3**^{Cr}**b**.

Identification code	GSTR740, DB-426 // GXray6736
Crystal habitus	clear yellow plate
Device type	STOE IPDS-2T
Empirical formula	$C_{66}H_{60}Cr_2N_4O_{11}P_2$
Moiety formula	2 ($C_{31}H_{25}CrN_2O_5P$), $C_4H_{10}O$
Formula weight / g/mol	1251.12
T / K	123
Crystal system	monoclinic
Space group	$P2_1/c$
a / Å	15.7633(15)
b / Å	9.6245(7)
c / Å	20.6670(19)
α / °	90
β / °	93.619(8)
γ / °	90
V / Å^3	3129.2(5)
Z	2
ρ_{calc} / g/cm^3	1.328

μ / mm^{-1}	0.461
F(000)	1300.0
Crystal size / mm^3	0.12 × 0.06 × 0.06
Absorption correction	integration
Min. and max. transmission	0.7408 and 0.9613
Radiation	Mo-K$_\alpha$ (λ = 0.71073 Å)
2Θ range for data collection / °	5.178 to 56
Completeness to Θ	0.997
Index ranges	$-20 \le h \le 20$, $-12 \le k \le 12$, $-27 \le l \le 27$
Reflections collected	29406
Independent reflections	7540 (R_{int} = 0.1380, R_σ = 0.1215)
Data / restraints / parameters	7540 / 32 / 410
Goodness-of-fit on F^2	0.990
Final R indexes ($I \ge 2\sigma(I)$)	R_1 = 0.0870, ωR_2 = 0.2111
Final R indexes (all data)	R_1 = 0.1675, ωR_2 = 0.2512
Largest diff. peak and hole / e/Å^3	1.08 and −0.80

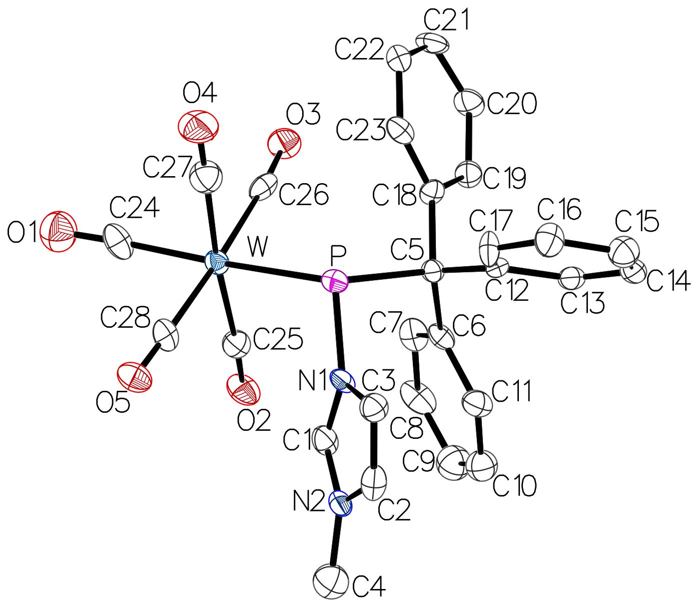

Figure 113: Molecular structures of **3c** in the single crystal lattice at 123(2) K. Thermal ellipsoids are set at 50% probability. Hydrogen atoms and solvent molecules were omitted for clarity. Suitable single crystals were obtained as clear yellow blocks by slow evaporation of a solution of 33 mg of **3c** in 1.0 mL of benzene-d_6 at ambient temperature in a glovebox. CCDC 2250851.

Alert level B

PLAT331_ALERT_2_B Small Aver Phenyl C-C Dist C29 --C34 . 1.36 Ang.

Author Response: These are solvent benzene, accurately modelled, but with less accuracy than the main complex. This is normal.

PLAT973_ALERT_2_B Check Calcd Positive Resid. Density on W 1.98 eA-3

Author Response: A peak of this size near W (AN = 74) is not considered to be unusual.

Table 8: Crystal data and structure refinements for **3c**.

Identification code	GSTR723, DB-362 // 6613
Crystal habitus	clear yellow block
Device type	STOE IPDS-2T
Empirical formula	$C_{37}H_{30}N_2O_5PW$
Moiety formula	$C_{28}H_{21}N_2O_5PW$, 1.5 (C_6H_6)
Formula weight / g/mol	797.45
T / K	123(2)
Crystal system	triclinic
Space group	*P*−1
a / Å	10.0209(7)
b / Å	11.6994(9)
c / Å	14.8295(11)
α / °	92.666(6)
β / °	101.738(6)
γ / °	94.449(6)
V / Å^3	1693.6(2)
Z	2
ρ_{calc} / g/cm^3	1.564
μ / mm^{-1}	3.502
F(000)	790.0
Crystal size / mm^3	0.15 × 0.12 × 0.08
Absorption correction	integration
Min. and max. transmission	0.1644 and 0.6078
Radiation	Mo-K$_\alpha$ (λ = 0.71073 Å)
2Θ range for data collection / °	5.492 to 55.996
Completeness to Θ	0.989
Index ranges	$-13 \le h \le 13$, $-15 \le k \le 15$, $-19 \le l \le 17$
Reflections collected	17922
Independent reflections	8113 (R_{int} = 0.1668, R_σ = 0.3153)
Data / restraints / parameters	8113 / 86 / 416
Goodness-of-fit on F^2	0.706
Final *R* indexes ($I \ge 2\sigma(I)$)	R_1 = 0.0633, ωR_2 = 0.1134
Final *R* indexes (all data)	R_1 = 0.1357, ωR_2 = 0.1327
Largest diff. peak and hole / e/Å^3	2.50 and −2.72

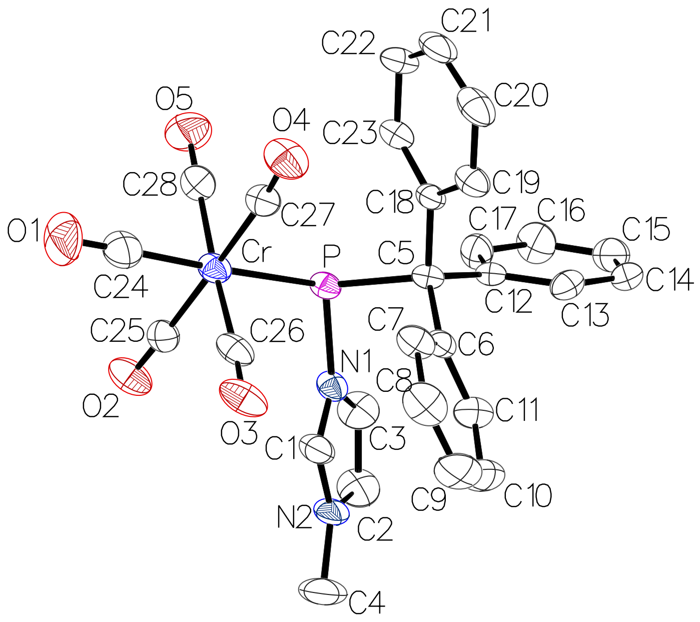

Figure 114: Molecular structures of **3^Cr^c** in the single crystal lattice at 180(2) K. Thermal ellipsoids are set at 50% probability. Hydrogen atoms and solvent molecules were omitted for clarity. Suitable single crystals were obtained as clear yellow blocks from a concentrated solution of 24 mg of **3b** in 0.5 mL of benzene-d_6 at ambient temperature in a J. Young NMR tube. CCDC 2250852.

Alert level B

PLAT331_ALERT_2_B Small Aver Phenyl C-C Dist C35 --C37_a . 1.36 Ang.

Author Response: These are solvent benzene, accurately modelled, but with less accuracy than the main complex. This is normal.

Table 9: Crystal data and structure refinements for **3^{Cr}c**.

Identification code	GSTR730, DB-373 // GXray6654
Crystal habitus	clear yellow block
Device type	STOE IPDS-2T
Empirical formula	$C_{37}H_{30}CrN_2O_5P$
Moiety formula	$C_{28}H_{21}CrN_2O_5P$, 1.5 (C_6H_6)
Formula weight / g/mol	665.60
T / K	180
Crystal system	triclinic
Space group	*P*−1
a / Å	10.0539(7)
b / Å	11.6733(9)
c / Å	14.6554(10)
α / °	90.504(6)
β / °	100.483(5)
γ / °	94.922(6)
V / Å^3	1684.5
Z	2
ρ_{calc} / g/cm^3	1.312

μ / mm^{-1}	0.432
$F(000)$	690.0
Crystal size / mm^3	0.35 × 0.15 × 0.15
Absorption correction	integration
Min. and max. transmission	0.7056 and 0.9405
Radiation	Mo-K_σ (λ = 0.71073 Å)
2Θ range for data collection / °	5.422 to 56
Completeness to Θ	0.982
Index ranges	$-13 \le h \le 13$, $-15 \le k \le 15$, $-18 \le l \le 19$
Reflections collected	14869
Independent reflections	7987 (R_{int} = 0.0638, R_σ = 0.2033)
Data / restraints / parameters	7987 / 0 / 404
Goodness-of-fit on F^2	0.635
Final R indexes ($I \ge 2\sigma(I)$)	R_1 = 0.0398, ωR_2 = 0.0599
Final R indexes (all data)	R_1 = 0.1225, ωR_2 = 0.0691
Largest diff. peak and hole / e/Å^3	0.34 and −0.26

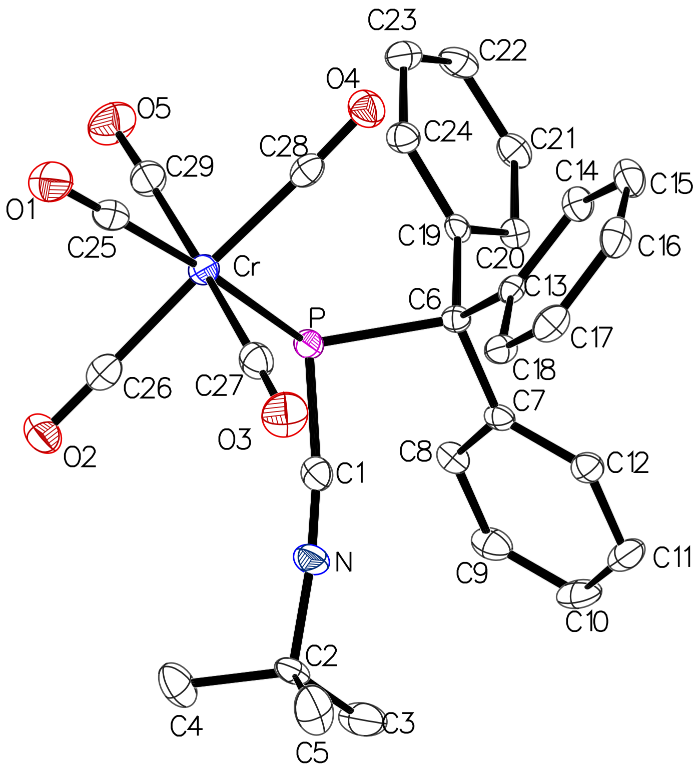

Figure 115: Molecular structures of **3^{Cr}d** in the single crystal lattice at 100(2) K. Thermal ellipsoids are set at 50% probability. Hydrogen atoms were omitted for clarity. Suitable single crystals were obtained as clear yellow plates by slow evaporation of a solution of 7 mg of **3^{Cr}d** in 1.5 mL of diethyl ether at −30 °C in a glovebox. CCDC 2250853.

Table 10: Crystal data and structure refinements for **3^Cr^d**.

Identification code	GSTR649, DB-83 // GXray5842f
Crystal habitus	yellow plate
Device type	Bruker X8-KappaApexII
Empirical formula	$C_{29}H_{24}NO_5PCr$
Moiety formula	$C_{29}H_{24}CrNO_5P$
Formula weight / g/mol	549.46
T / K	100
Crystal system	monoclinic
Space group	$P2_1/n$
a / Å	13.1380(11)
b / Å	13.1116(11)
c / Å	15.6049(13)
α / °	90
β / °	90.806(3)
γ / °	90
V / Å^3	2687.8(4)
Z	4
ρ_{calc} / g/cm^3	1.358
μ / mm^{-1}	0.524
F(000)	1136.0
Crystal size / mm^3	0.26 × 0.24 × 0.04
Absorption correction	empirical
Min. and max. transmission	0.6541 and 0.7462
Radiation	Mo-K$_\alpha$ (λ = 0.71073 Å)
2Θ range for data collection / °	5.084 to 55.99
Completeness to Θ	0.998
Index ranges	$-17 \le h \le 17$, $-17 \le k \le 17$, $-20 \le l \le 20$
Reflections collected	44084
Independent reflections	6480 (R_{int} = 0.0956, R_σ = 0.0631)
Data / restraints / parameters	6480 / 0 / 337
Goodness-of-fit on F^2	1.016
Final *R* indexes ($I \ge 2\sigma(I)$)	R_1 = 0.0399, ωR_2 = 0.0809
Final *R* indexes (all data)	R_1 = 0.0760, ωR_2 = 0.0947
Largest diff. peak and hole / e/Å^3	0.35 and −0.51

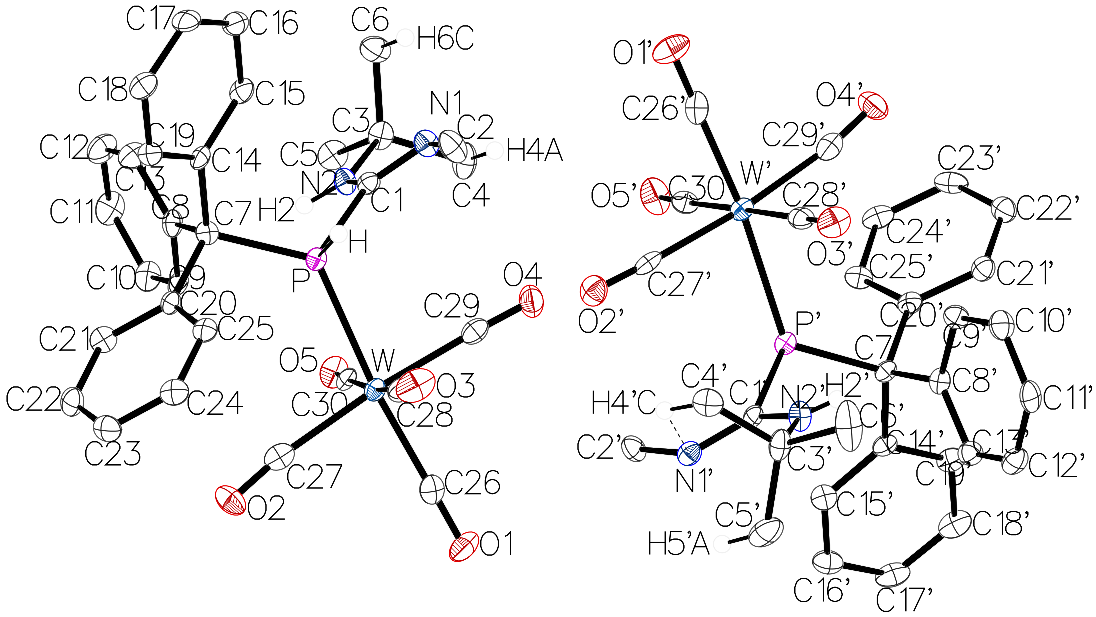

Figure 116: Molecular structures of **11a** in the single crystal lattice at 100(2) K. Thermal ellipsoids are set at 50% probability. Hydrogen atoms and solvent molecules were omitted for clarity except for those bound to phosphorus and nitrogen atoms or that are in close proximity to a nitrogen atom. Suitable single crystals were obtained as clear light yellow plates by slow evaporation of a solution of **11a** in diethyl ether at ambient temperature in a glovebox. CCDC 2250854.

Table 11: Crystal data and structure refinements for **11a**.

Identification code	GSTR672, DB-116.1 // GXray6002f
Crystal habitus	clear light yellow plate
Device type	Bruker X8-KappaApexII
Empirical formula	$C_{30}H_{29}N_2O_5PW$
Moiety formula	$C_{30}H_{29}N_2O_5PW$
Formula weight / g/mol	712.37
T / K	100
Crystal system	triclinic
Space group	$P-1$
a / Å	9.2076(5)
b / Å	17.6788(10)
c / Å	17.9311(9)
α / °	87.109(3)
β / °	82.372(3)
γ / °	78.688(3)
V / Å^3	2835.9(3)
Z	4
ρ_{calc} / g/cm^3	1.669
μ / mm^{-1}	4.172
F(000)	1408.0

Crystal size / mm^3	0.18 × 0.1 × 0.04
Absorption correction	empirical
Min. and max. transmission	0.4487 and 0.7461
Radiation	Mo-K$_\alpha$ (λ = 0.71073 Å)
2Θ range for data collection / °	2.35 to 55.996
Completeness to Θ	0.998
Index ranges	$-12 \le h \le 12$, $-23 \le k \le 23$, $-23 \le l \le 23$
Reflections collected	133248
Independent reflections	13670 (R_{int} = 0.1023, R_σ = 0.0518)
Data / restraints / parameters	13670 / 0 / 723
Goodness-of-fit on F^2	1.113
Final R indexes ($I \ge 2\sigma(I)$)	R_1 = 0.0377, ωR_2 = 0.0689
Final R indexes (all data)	R_1 = 0.0595, ωR_2 = 0.0774
Largest diff. peak and hole / e/Å^3	1.86 and −1.82

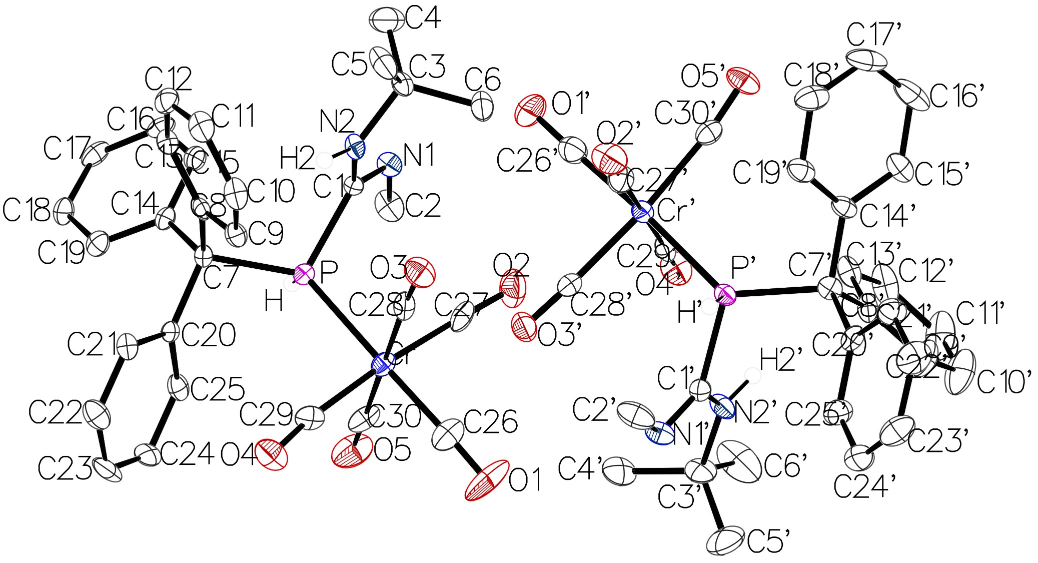

Figure 117: Molecular structures of **11^{Cr}a** in the single crystal lattice at 123(2) K. Thermal ellipsoids are set at 50% probability. Hydrogen atoms and solvent molecules were omitted for clarity except for those bound to phosphorus and nitrogen atoms. Suitable single crystals were obtained as clear light yellow needles by slow evaporation of a solution of 7 mg of **11^{Cr}a** in 1.5 mL of diethyl ether at ambient temperature in a glovebox. CCDC 2250855.

Alert level B

PLAT910_ALERT_3_B Missing # of FCF Reflection(s) Below Theta(Min). 13 Note

Author Response: The data in this structure is somewhat marginal, but a fully model has been generated. Intensities fall off above Theta 80 deg and has lead to some unobserved rfl.

Table 12: Crystal data and structure refinements for **11^{Cr}a**.

Identification code	GSTR734, DB-398 // GXray6704
Crystal habitus	clear light yellow needle
Device type	STOE IPDS-2T
Empirical formula	$C_{30}H_{29}CrN_2O_5P$
Moiety formula	$C_{30}H_{29}CrN_2O_5P$
Formula weight / g/mol	580.52
T / K	123(2)
Crystal system	triclinic
Space group	P−1
a / Å	9.1284(5)
b / Å	13.1778(6)
c / Å	24.2171(13)
α / °	89.744(4)
β / °	81.178(4)
γ / °	84.886(4)
V / Å^3	2867.1(3)
Z	4
ρ_{calc} / g/cm^3	1.345
μ / mm^{-1}	0.496
F(000)	1208.0
Crystal size / mm^3	0.18 × 0.04 × 0.03
Absorption correction	integration
Min. and max. transmission	0.9272 and 0.9909
Radiation	Mo-K$_\alpha$ (λ = 0.71073 Å)
2Θ range for data collection / °	5.32 to 56
Completeness to Θ	0.998
Index ranges	$-9 \leq h \leq 12$, $-16 \leq k \leq 17$, $-31 \leq l \leq 31$
Reflections collected	32389
Independent reflections	13808 (R_{int} = 0.1148, R_σ = 0.2248)
Data / restraints / parameters	13808 / 0 / 717
Goodness-of-fit on F^2	0.725
Final R indexes ($I \geq 2\sigma(I)$)	R_1 = 0.0529, ωR_2 = 0.0624
Final R indexes (all data)	R_1 = 0.1523, ωR_2 = 0.0784
Largest diff. peak and hole / e/Å^3	0.47 and −0.46

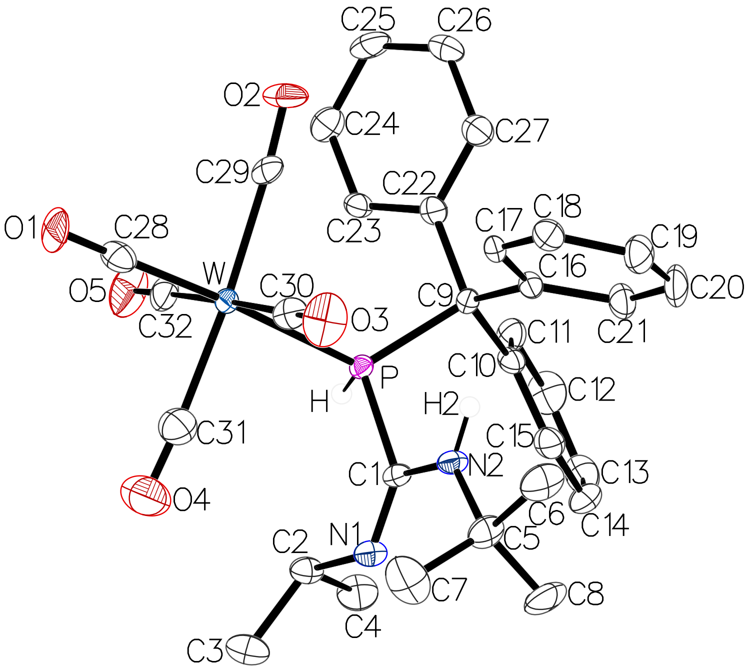

Figure 118: Molecular structures of **11b** in the single crystal lattice at 123(2) K. Thermal ellipsoids are set at 50% probability. Hydrogen atoms and solvent molecules were omitted for clarity except for those bound to phosphorus and nitrogen atoms. Suitable single crystals were obtained as clear light yellow needles by slow evaporation of a solution of 7 mg of **11b** in 1.5 mL of diethyl ether at ambient temperature in a glovebox. CCDC 2250856.

Table 13: Crystal data and structure refinements for **11b**.

Identification code	DB-400 // GXray6705
Crystal habitus	clear colorless plate
Device type	STOE IPDS-2T
Empirical formula	$C_{32}H_{33}N_2O_5PW$
Moiety formula	$C_{32}H_{33}N_2O_5PW$
Formula weight / g/mol	740.42
T / K	123(2)
Crystal system	monoclinic
Space group	$P2_1/n$
a / Å	17.2823(4)
b / Å	10.1835(3)
c / Å	19.4703(4)
α / °	90
β / °	113.249(2)
γ / °	90
V / Å^3	3148.41(14)
Z	4
ρ_{calc} / g/cm^3	1.562
μ / mm^{-1}	3.761

F(000)	1472.0
Crystal size / mm^3	0.15 × 0.11 × 0.04
Absorption correction	multi-scan
Min. and max. transmission	0.2428 and 0.2974
Radiation	Mo-K$_\alpha$ (λ = 0.71073 Å)
2Θ range for data collection / °	5.13 to 58.354
Completeness to Θ	0.994
Index ranges	$-23 \le h \le 23$, $-13 \le k \le 13$, $-26 \le l \le 22$
Reflections collected	20535
Independent reflections	8410 (R_{int} = 0.0318, R_σ = 0.0492)
Data / restraints / parameters	8410 / 305 / 378
Goodness-of-fit on F^2	0.963
Final R indexes ($I \ge 2\sigma(I)$)	R_1 = 0.0287, ωR_2 = 0.0579
Final R indexes (all data)	R_1 = 0.0478, ωR_2 = 0.0615
Largest diff. peak and hole / e/Å^3	0.80 and −1.35

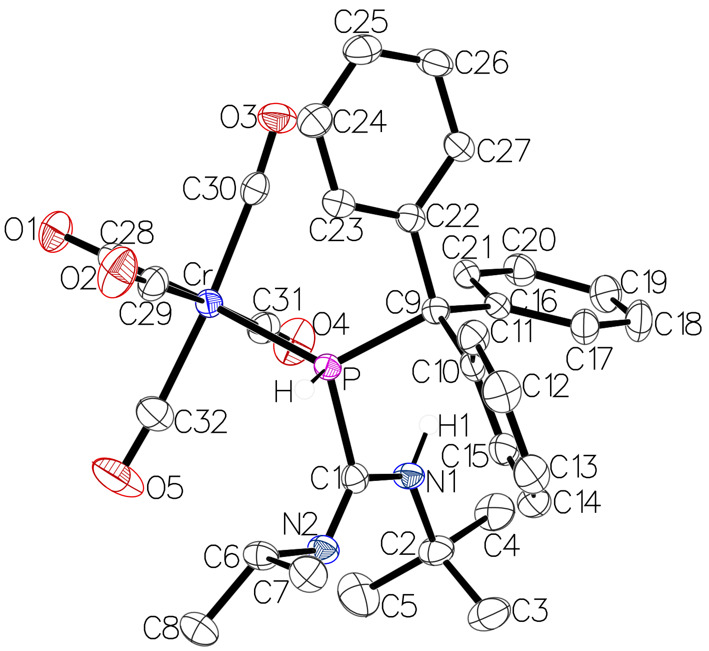

Figure 119: Molecular structures of **11^{Cr}b** in the single crystal lattice at 123(2) K. Thermal ellipsoids are set at 50% probability. Hydrogen atoms and solvent molecules were omitted for clarity except for those bound to phosphorus and nitrogen atoms. Suitable single crystals were obtained as clear light yellow needles by slow evaporation of a solution of 12 mg of **11^{Cr}b** in 0.5 mL of isopropylamine at ambient temperature in a glovebox. CCDC 2250857.

Table 14: Crystal data and structure refinements for **11^{Cr}b**.

Identification code	GSTR714, DB-301 // GXray6529
Crystal habitus	clear light yellow block
Device type	STOE IPDS-2T
Empirical formula	$C_{32}H_{33}CrN_2O_5P$
Moiety formula	$C_{32}H_{33}CrN_2O_5P$
Formula weight / g/mol	608.57
T / K	123
Crystal system	monoclinic
Space group	$P2_1/n$
a / Å	17.2112(9)
b / Å	10.0937(4)
c / Å	19.2780(10)
α / °	90
β / °	113.077(4)
γ / °	90
V / Å^3	3081.1(3)
Z	4
ρ_{calc} / g/cm^3	1.312
μ / mm^{-1}	0.465
F(000)	1272.0
Crystal size / mm^3	0.14 × 0.09 × 0.07
Absorption correction	integration
Min. and max. transmission	0.8669 and 0.9766
Radiation	Mo-K$_\alpha$ (λ = 0.71073 Å)
2Θ range for data collection / °	5.146 to 55.998
Completeness to Θ	0.999
Index ranges	$-22 \le h \le 22$, $-13 \le k \le 13$, $-22 \le l \le 25$
Reflections collected	24207
Independent reflections	7447 (R_{int} = 0.0675, R_σ = 0.1004)
Data / restraints / parameters	7447 / 0 / 378
Goodness-of-fit on F^2	0.840
Final *R* indexes ($I \ge 2\sigma(I)$)	R_1 = 0.0420, ωR_2 = 0.0591
Final *R* indexes (all data)	R_1 = 0.0959, ωR_2 = 0.0691
Largest diff. peak and hole / e/Å^3	0.26 and −0.48

7 Theoretical Investigations

Computational details

Quantum chemical calculations were performed with ORCA (v. 4.2.1).[10] All geometry optimizations were run in redundant internal coordinates with tight convergence criteria, using the B3LYP[11,12] functional together with the powerful speeding up RIJCOSX algorithm[13] and the Ahlrichs' segmented def2-TZVP basis set.[14,15] For W atoms the [SD(60,MWB)] effective core potential (ECP) was used, as obtained from Turbomole basis set library (ftp://ftp.chemie.uni-karlsruhe.de/pub/basen/).[16] In all optimizations and energy evaluations, the 2010 Grimme's semiempirical atom-pair-wise correction (DFT-D3 methods), taking into account the major part of the contribution of dispersion forces to the energy, was included.[17,18] Harmonic frequency calculations verified the nature of the computed species as minima or TS (transition state) structures, featuring none or only one negative eigenvalues, respectively. Moreover, all TS structures were confirmed by intrinsic reaction coordinate (IRC) calculations. From these geometries, all reported electronic data were obtained by means of single-point (SP) calculations using (unless otherwise indicated) the same functional as well as the more polarized def2-TZVPP[14,15] or def2-QZVPP[19] basis set. Basis sets may be obtained from the Basis Set Exchange (BSE) software and the EMSL Basis Set Library (https://bse.pnl.gov/bse/portal).[20] Reported energies were corrected for the Gibbs energy term at the optimization level and obtained in most cases by means of the recently developed near linear scaling domain-based local pair natural orbital (DLPNO) method[21] to achieve the "gold standard" coupled cluster theory with single-double and perturbative triple excitations (CCSD(T)),[22] except for the more demanding trityl-substituted (real) derivatives, for which the computationally less costly double-hybrid-meta-GGA functional PWPB95[23,24] with Grimme's D3 correction (PWPB95-D3) was used. The last level was used to obtain HOMO/LUMO energies and Mulliken electric charges, as well as for TOP and FIA calculations in order to allow the calculation of trityl-substituted derivatives. Reactions depicted in Figure 7 of the main text were also computed using Grimme's fast PBEh-3c composite functional[25] for optimization and frequency calculation, and the same PWPB95-D3/def2-TZVPP(ecp) level for final energy evaluation (see Figure 122). NBO and AIM wavefunction analyses were performed at the B3LYP/def2-TZVPP level with NBO 6.0[26] and Multiwfn 3.7,[27] respectively. Solvent effects (tetrahydrofuran) for the mechanistic study of the reaction of the isocyanide model adduct with amines were considered via the COSMO solvation model[28,29] for both geometry optimizations and energy SP calculations, in analogy with previous reports.[30,31] In all other cases solvent effects (toluene) were included using the Conductor-like Polarizable Continuum Model (CPCM) [32,33] as standard method. For the above-mentioned mechanistic study, selected reactions were

checked using the CPCM(THF) alternative and show negligible energy differences. Isotropic values (σ_{Iso}) for the ^{31}P NMR magnetic shielding tensor were computed using the Gauge Including Atomic Orbital (GIAO) method,[34] using the PBE0[35,36] functional and the def2-TZVP(ecp) basis set. The expected chemical shifts δ^P were estimated through a linear equation $\delta^P = 237.68 - 0.8628 \cdot \sigma_{Iso}$, which in turn was obtained from a linear regression ($R^2 = 0.997$) of nine reference compounds spanning a wide range of chemical shifts, as reported elsewhere.[37] Thermodynamic oxygen transfer potentials (TOP) were computed as zero-point corrected energy values and referred to the H_2O/H_2O_2 redox couple,[38] whereas fluoride ion affinities (FIA) were obtained as enthalpy variations relative to the Tms^+/Tms-F pair,[39] both parameters being computed in the gas phase.

Correlation of the ^{31}P NMR data with the HOMO-LUMO gap

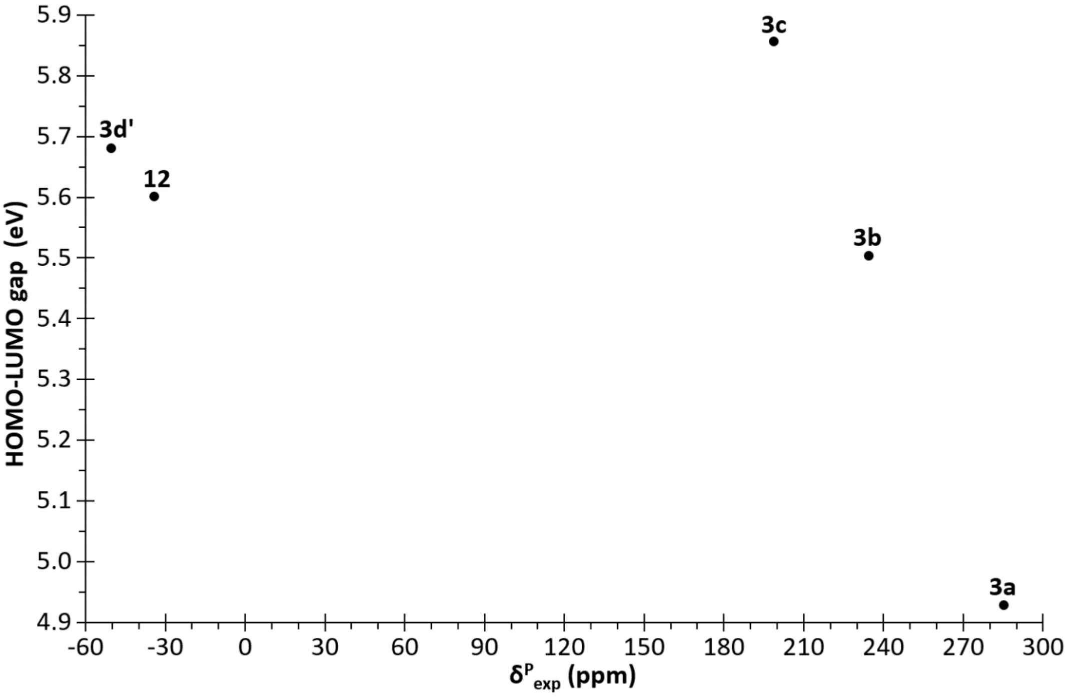

Figure 120: Plot of the computed [CPCM$_{tol}$/PWPB95-D3/def2-QZVPP(ecp)//CPCM$_{tol}$/B3LYP-D3/def2-TZVP(ecp)] HOMO-LUMO gap energies with the experimental ^{31}P chemical shift values.

Fluoride ion affinitiy (FIA) and thermodynamic oxygen atom transfer potentials (TOP)

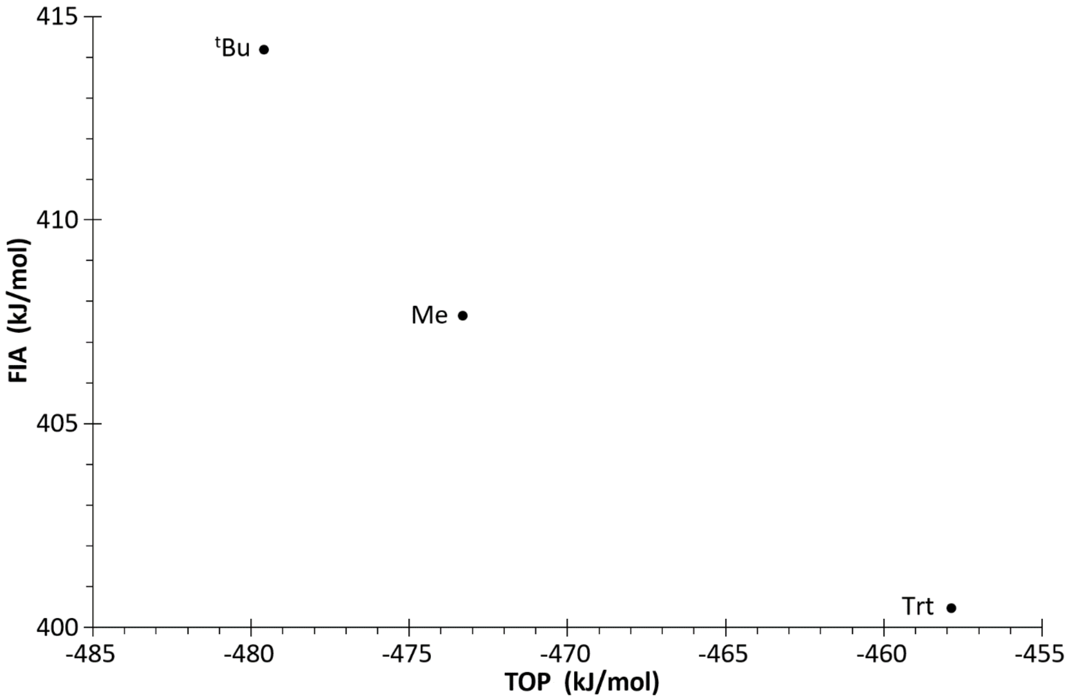

Figure 121: Computed [$CPCM_{tol}$/PWPB95-D3/def2-QZVPP(ecp)//$CPCM_{tol}$/B3LYP-D3/def2-TZVP(ecp)] fluoride ion affinity and thermodynamic oxygen atom transfer potentials for differently *P*-substituted phosphinidene *P*-W(CO)$_5$ complexes.

Computed energy profile at the alternative PBEh-3c optimization level

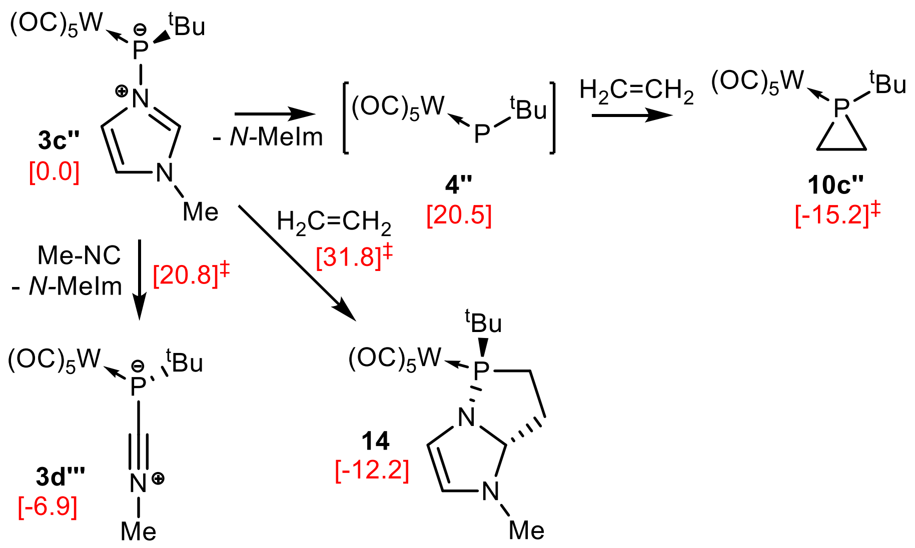

Figure 122: Proposed mechanism for the reactions of model *N*-MeIm adduct complex **3c''** with methyl isocyanide and ethylene. Computed [$CPCM_{toluene}$/CCSD(T)/def2-TZVPP(ecp)//$CPCM_{toluene}$/PBEh-3c] relative Gibbs free energies (kcal mol^{-1}) in red and square brackets.

Computed structures

Cartesian coordinates (in Å) and energies (in hartrees) for all computed species can be found in a separate source data file in the supplementary data. Geometries, zero-point energy correction (ZPE) and Gibbs energy correction (G_{corr}) at the optimization level (vide supra), whereas electronic energies are computed at the COSMO(THF)/CCSD(T)/def2-TZVPP(ecp) level unless otherwise stated.

5. References

1. Armarego, W. *Purification of Laboratory Chemicals* (Elsevier, 2003).
2. Fulmer, R. R. et al. NMR chemical shifts of trace impurities: Common laboratory solvents, organics, and gases in deuterated solvent relevant to the organometallic chemist. *Organometallics* **29**, 2176–2179 (2010).
3. Harris, R. K., Becker, E. D., Cabral de Menezes, S. M., Granger, P., Hoffman, R. E. & Zilm, K. W. Further conventions for NMR shielding and chemical shifts. *Pure Appl. Chem.* **80**, 59–84 (2008).
4. Harris, R. K., Becker, E. D., Cabral de Menezes, S. M., Goodfellow, R. & Granger, P. NMR nomenclature, nuclear spin properties and conventions for chemical shifts. *Pure Appl. Chem.* **73**, 1795–1818 (2001).
5. Blessing, R. H. An empirical correction for absorption anisotropy. *Acta Crystallogr., Sect. A: Found. Crystallogr.* **51**, 33–38 (1995).
6. Sheldrick, G. M. *ShelXS97 and ShelXL97*, University of Göttingen, Germany (1997).
7. Dolomanov, O. V., Bourhis, L. J., Gildea, R. J., Howard, J. A. K. & Puschman, H. OLEX2: a complete structure solution, refinement and analysis program. *J. Appl. Crystallogr.* **42**, 339–341 (2009).
8. Stojanovic, R. S. & Bond, A. M. Examination of conditions under which the reduction of the cobaltocenium cation can be used as standard voltammetric reference process in organic and aqueous solvents. *Anal. Chem.* **65**, 56–64 (1993).
9. Gritzner, G. & Kuta, J. Recommendations on reporting electrode potentials in nonaqueous solvents. *Pure Appl. Chem.* **56**, 461–466 (1984).
10. Neese, F. The ORCA program system. *WIREs Comput. Mol. Sci.* **2**, 73–78 (2012).

11. Becke, A. D. Density-functional thermochemistry. III. The role of exact exchange. *J. Chem. Phys.* **98**, 5648–5652 (1993).

12. Lee, C., Yang, W. & Parr, R. G. Development of the Colle-Salvetti correlation-energy formula into a functional of the electron density. *Phys. Rev. B* **37**, 785–789 (1988).

13. Neese, F., Wennmohs, F., Hansen, A. & Becker, U. Efficient, approximate and parallel Hartree-Fock and hybrid DFT calculations. A 'chain-of-spheres' algorithm for the Hartree-Fock exchange. *Chem. Phys.* **356**, 98–109 (2009).

14. Schäfer, A., Huber, C. & Ahlrichs, R. Fully optimized contracted Gaussian basis sets of triple zeta valence quality for atoms Li to Kr. *J. Chem. Phys.* **100**, 5829–5835 (1994).

15. Weigend, F. & Ahlrichs, R. Balanced basis sets of split valence, triple zeta valence and quadruple zeta valence quality for H to Rn: design and assessment of accuracy. *Phys. Chem. Chem. Phys.* **7**, 3297–3305 (2005).

16. Andrae, D., Häußermann, U., Dolg, M., Stoll, H. & Preuß, H. Energy-adjusted ab initio pseudopotentials for the second and third row transition elements. *Theor. Chim. Acta* **77**, 123–141 (1990).

17. Grimme, S., Antony, J., Ehrlich, S. & Krieg, H. A consistent and accurate ab initio parametrization of density functional dispersion correction (DFT-D) for the 94 elements H-Pu. *J. Chem. Phys.* **132**, 154104.

18. Grimme, S., Ehrlich, S. & Goerigk, L. Effect of the damping function in dispersion corrected density functional theory. *J. Comput. Chem.* **32**, 1456–1465 (2011).

19. Weigend, F., Furche, F. & Ahlrichs, R. Gaussian basis sets of quadruple zeta valence quality for atoms H-Kr. *J. Chem. Phys.*, **119**, 12753–12762 (2003).

20. Feller, D. The role of databases in support of computational chemistry calculations. *J. Comput. Chem.* **17**, 1571–1586 (1996).

21. Riplinger, C., Sandhoefer, B., Hansen, A. & Neese, F. Natural triple excitations in local coupled cluster calculations with pair natural orbitals. *J. Chem. Phys.* **139**, 134101 (2013).

22. Pople, J. A., Head-Gordon, M. & Raghavachari, K. Quadratic configuration interaction. A general technique for determining electron correlation energies. *J. Chem. Phys.* **87**, 5968–5975 (1987).

23. Goerigk, L. & Grimme, S. Efficient and accurate double-hybrid-meta-GGA density functionals - Evaluation with the extended GMTKN30 database for general main group

thermochemistry, kinetics, and noncovalent interactions. *J. Chem. Theory Comput*. **7**, 291–309 (2011).

24. Goerigk, L. & Grimme, S. A thorough benchmark of density functional methods for general main group thermochemistry, kinetics, and noncovalent interactions. *Phys. Chem. Chem. Phys.* **13**, 6670–6688 (2011).

25. Grimme, S., Brandenburg, J. G., Bannwarth, C. & Hansen, A. Consistent structures and interactions by density functional theory with small atomic orbital basis sets. *J. Chem. Phys.* **143**, 054107 (2015).

26. Glendening, E. D. *et al.* NBO 6.0. Theoretical Chemistry Institute, University of Wisconsin, Madison (2012).

27. Lu, T. & Chen, F. Multiwfn: a multifunctional wavefunction analyzer. *J. Comput. Chem.* **33**, 580–592. Website: http://sobereva.com/multiwfn/28. Klamt, A. & Schüürmann, G. COSMO: a new approach to dielectric screening in solvents with explicit expressions for the screening energy and its gradient. *J. Chem. Soc., Perkin Trans.* **2**, 799–805 (1993).

29. Klamt, A. Conductor-like screening model for real solvents: a new approach to the quantitative calculations of solvation phenomena. *J. Phys. Chem.* **99**, 2224–2235 (1995).

30. Kunzmann, R. *et al.* A synthetic equivalent for unknown 1,3-zwitterions? – A K/OR phosphinidenoid complex with an additional Si-Cl function. *Chem. Commun.*, **56**, 3899–3902 (2020).

31. Junker, P. *et al.* A case study on the conversion of Li/Cl phosphinidenoid into phosphinidene complexes. *Dalton Trans.*, **50**, 739–745 (2021).

32. Barone, V. & Cossi, M. Quantum calculation of molecular energies and energy gradients in solution by a conductor solvent model. *J. Phys. Chem. A*, **102**, 1995–2001 (1998).

33. Cossi, M., Rega, N., Scalmani, G. & Barone, V. Energies, structures, and electronic properties of molecules in solution with the C-PCM solvation model. *J. Comp. Chem.*, **24**, 669–681 (2003).

34. Ziegler, T. & Schreckenbach, G. Calculation of NMR shielding tensors using gauge-including atomic orbitals and modern density functional theory. *J. Phys. Chem.* **99**, 606–611 (1995).

35. Perdew, J. P., Burke, K. & Ernzerhof, M. Generalized gradient approximation made simple. *Phys. Rev. Lett.* **77**, 3865–3868 (1996).

36. Perdew, J. P., Burke, K. & Ernzerhof, M. Errata: Generalized gradient approximation made simple. *Phys. Rev. Lett.* **78**, 1396 (1997).

37. Gese, A., Kermanshashian, S., Schnakenburg, G., Kelemen, Z., Nyulaszi, L., Espinosa Ferao, A. & Streubel R. Towards a 1,4-diphosphinine-based molecular CPS-ternary compound. *Inorg. Chem.* **60**, 13029–13040, (2021).

38. Schulten, C., von Frantzius, G., Schnakenburg, G., Espinosa, A. & Streubel, R. Deoxygenation of carbon dioxide by electrophilic terminal phosphinidene complexes. *Chem. Sci.* **3**, 3526–3533 (2012).

39. Erdmann, P., Leitner, J., Schwarz, J. & Greb, L. An extensive set of accurate fluoride ion affinities for p-block element Lewis acids and basic design principles for strong fluoride ion acceptors. *ChemPhysChem* **21**, 987–994 (2020).
